# An Accurate Approach for Computational pKa Determination of Phenolic Compounds

**DOI:** 10.3390/molecules27238590

**Published:** 2022-12-06

**Authors:** Silvia Pezzola, Samuele Tarallo, Alessandro Iannini, Mariano Venanzi, Pierluca Galloni, Valeria Conte, Federica Sabuzi

**Affiliations:** Department of Chemical Science and Technologies, University of Rome Tor Vergata, 00133 Rome, Italy

**Keywords:** pKa, computational pKa, phenol, thymol, direct approach, DFT, CAM-B3LYP, solvation model based on density (SMD)

## Abstract

Computational chemistry is a valuable tool, as it allows for in silico prediction of key parameters of novel compounds, such as pKa. In the framework of computational pKa determination, the literature offers several approaches based on different level of theories, functionals and continuum solvation models. However, correction factors are often used to provide reliable models that adequately predict pKa. In this work, an accurate protocol based on a direct approach is proposed for computing phenols pKa. Importantly, this methodology does not require the use of correction factors or mathematical fitting, making it highly practical, easy to use and fast. Above all, DFT calculations performed in the presence two explicit water molecules using CAM-B3LYP functional with 6-311G+dp basis set and a solvation model based on density (SMD) led to accurate pKa values. In particular, calculations performed on a series of 13 differently substituted phenols provided reliable results, with a mean absolute error of 0.3. Furthermore, the model achieves accurate results with -CN and -NO_2_ substituents, which are usually excluded from computational pKa studies, enabling easy and reliable pKa determination in a wide range of phenols.

## 1. Introduction

Phenols are a class of odorous compounds mainly present in plant essential oils that is attracting increasing scientific and applicative interest. Their use is extremely widespread, ranging from antimicrobials and antivirals to cancer treatments and antioxidants also adopted in fuels [1,2]. Native compounds are exploited in the biomedical field for their ability to interact with cell membranes, provoking cell death without triggering a specific pathway [3,4,5]. This behavior plays a fundamental role, as it often minimizes pharmacological resistance. Because antibiotic resistance is major cause of death every year [6], the possibility of developing new compounds combining the potentially flawless natural properties of phenols with tailored structural modification is compelling. To this end, computational chemistry is pivotal, offering the possibility of designing molecules and predicting their physicochemical properties [7]. It prevents time- and cost-consuming experiments, suggesting easily screenable sets of data. Computational calculation of acid–base dissociation constants (pKa) is among the most embraced methods [8,9].

The literature includes many strategies proposed to calculate pKa through computational models. The most common methodologies are the thermodynamic cycles theory and the direct approach [10]. Considering the dissociation equilibrium of a generic acid, HA:

(1) $${\mathrm{HA}}_{\left(\mathrm{sol}\right)}⇌A^{-}{}_{\left(\mathrm{sol}\right)}+H^{+}{}_{\left(\mathrm{sol}\right)}$$

using the thermodynamic cycle theory, pKa determination requires the estimation of the Gibbs free energy of the acid–base equilibrium in the gas phase (ΔG_g_) and the solvation free energy for each species involved in the equilibrium (ΔΔG_solv_) [11]. Although accurate, this model is time-consuming and laborious. Conversely, the direct approach is based on the calculation of the Gibbs free energy of the acid–base equilibrium in solution (ΔG_sol_) [12] without requiring gas-phase calculations [11]. Although the direct method depends on different variables, such as the level of theory, the molecules involved in the equilibrium and the adopted solvation model, it has shown to be as accurate as the thermodynamic cycle theory [13].

However, both models require the free energy of the solvated proton, which cannot be calculated using DFT methods because H^+^ has no electrons [13,14]. Therefore, a wide range of experimental values (between −252.6 and −271.7 kcal⋅mol^−1^) [11] is usually adopted, although this method is highly questioned [12]. Alternatively, correction factors and linear regressions are commonly used to obtain reliable results, leading to relatively accurate data [15,16,17,18].

To further improve precision in pKa calculation, in the last two decades, the inclusion of explicit water molecules in the first solvation shell has been proposed. Using water as the solvent in a continuum model, ΔpKa (i.e., pKa_calc_—pKa_exp_) values were still higher than 1 unit [17]. Thus, the use of correction factors can be used to reduce the gap between the experimental pKa and the calculated value [17].

In order to overcome issues related to the use of experimental values for proton solvation free energy, the acid–base dissociation equilibrium can be rewritten as Equation (2) [18,19,20]:

(2) $${\mathrm{HA}(H_{2}O)}_{n(\mathrm{sol})}+{\mathrm{OH}}^{-}(H_{2}O){}_{n(\mathrm{sol})}\;⇌\;A^{-}(H_{2}O){}_{n(\mathrm{sol})}+H_{2}O(H_{2}O){}_{n(\mathrm{sol})}$$

where n is the number of explicit water molecules.

In this model, the number of charged species is conserved on both sides of the equation; thus, errors in calculations are reduced [11]. This equation [20] allows for the use of continuum solvation theories with zero to several (n) explicit water molecules to shape a solvation cage around the compounds involved in the equilibrium. In this case, correction factors ensure reliable pKa values.

Taking into account the numerous models proposed in the literature, the aim of this work is to offer a practical, accurate and ready-to-use protocol for computational pKa determination of phenol derivatives through a direct approach that does not require correction factors or mathematical fitting [18,19]. A screening of functional and solvation models is thus proposed to accurately compute pKa. A series of differently substituted phenol derivatives was selected on the basis of their biological, pharmaceutical, industrial and synthetic interest in order to validate the proposed methodology.

## 2. Results and Discussion

In silico pKa determination requires investigation of a wide set of conditions [21]; in general, DFT calculations are preferred to ab initio calculations, owing to their lower computational and time costs. In this work, we screened multiple functionals in order to identify the most suitable model in terms of reliability and computational cost. B3PW91 was selected, owing to its reliability based on exchange- and gradient-corrected correlation functionals [22]. B3LYP was chosen because it is a versatile functional in modelling small and medium-sized organic molecules [23], and it uses 20% Hartree–Fock (HF) exchange. CAM-B3LYP is based on B3LYP but it better depicts long-range interactions fundamental in describing hydrogen bonds [24]. wB97XD includes a long-range correction based on the empirical dispersion term of van der Waals interactions [25,26]. Hence, such theories adequately describe hydrogen bonds and electrostatic interactions in water solutions [25]. Likewise, 6-311G+dp was selected as a basis set for its accuracy and reliability in describing the behavior of first- and second- row elements, with an acceptable computational cost [24]. Solvation continuum models were chosen based on their isotropic properties. In particular, SMD, CPCM and IEFPCM consider water a continuum dielectric field, but they differ in terms of solvent cavity description. Truhlar and colleagues defined SMD as a continuum solvation model effective for charged and neutral compounds in any solvent in which just a few parameters are known or required [23]. CPCM and IEFPCM are similar from a common end-user point of view, although IEFPCM has a higher computational cost [27].

Thus, pKa was calculated according to the dissociation equilibrium reported in Equation (2) [19,20]. Reaction energy (ΔE_dep_) was estimated as follows:

ΔE_dep_ = E_A−_ + E_H2O_ − E_OH−_ − E_HA_ (3)

where E_A−_, E_H2O_, E_OH−_ and E_HA_ are the electronic energies of each species (eventually calculated in the presence of explicit water molecules). After obtaining ΔE_dep_, it is possible to estimate the pKa according to the following equation: [28]

pKa = ΔE_dep_/2.302RT + 15.74 (4)

Preliminary studies were performed on phenol and thymol as leading compounds. pKa values were calculated according to equation 4, without adding explicit water molecules. The geometry was optimized for each functional in the vacuum, followed by the calculation of electronic energy in a continuum solvent using the specific solvation models. Table 1 lists ΔpKa values as pKa_calc_—pKa_exp_.

Results show that with all analyzed functionals and solvation models, pKa values are largely under- or overestimated; thus, ΔpKa values are considerably higher than 1 pKa unit, which is the acceptable threshold value for studies of this sort. The accuracy of data seems not to be affected by the complexity of the theory or by the dielectric polarizability of the models. It is not possible to highlight a general trend. The worst values were obtained by applying the SMD model with B3LYP and B3PW91, whereas ΔpKa values with CPCM and IEFPCM are slightly better. CAM-B3LYP and wB97XD exhibited an opposite trend, as ΔpKa values obtained with SMD are lower than those obtained with the other solvation models.

To improve the model, one and two water molecules were made explicit for each species involved in the equilibrium. A general improvement in ΔpKa values was observed, including one explicit water molecule (Table 2).

In particular, all ΔpKa values were less than 4 units and, with B3LYP and CAM-B3LYP in SMD, ΔpKa values of less than 1 were successfully obtained. Furthermore, for each functional, the SMD solvation model led to satisfactory values for acid dissociation constant, with a ΔpKa slightly higher than 1.

The addition of two explicit water molecules led to a further improvement in the system. Specifically, ΔpKa values lower than 1 were obtained with SMD (Table 3). B3LYP and CAM-B3LYP showed improved results, leading to a pKa_calc_ value close to the experimental value for phenol, with a ΔpKa slightly higher than zero.

**Figure 1:**
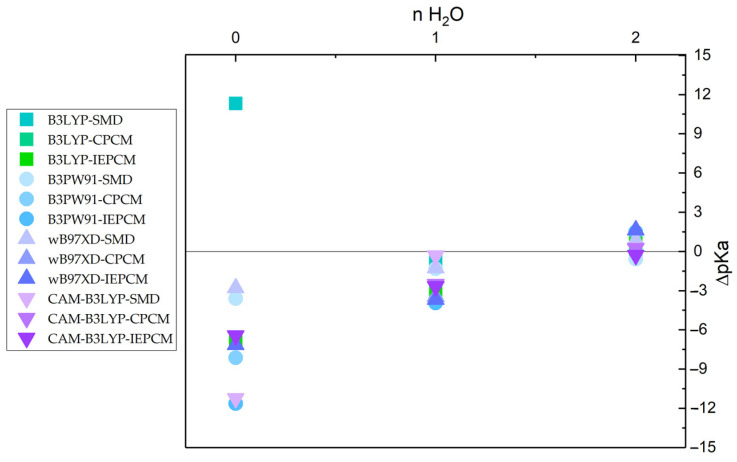
ΔpKa vs. number of explicit water molecules.

Figure 1 depicts the dependence of ΔpKa on the number of explicit water molecules for phenol; an increase in the number of water molecules results in a decrease in ΔpKa values to approximately zero. Therefore, no further investigations were performed with additional explicit water molecules.

**Figure 2:**
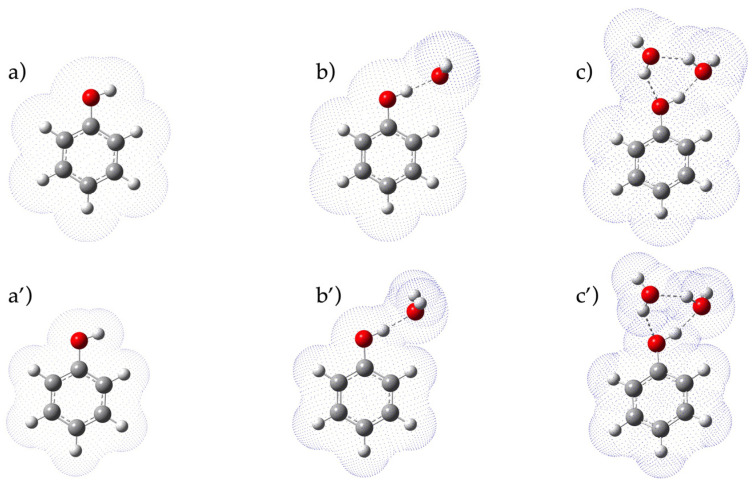
Solvation cavity of phenol with different models: IEFPCM in the presence of 0 (**a**), 1 (**b**) and 2 (**c**) explicit water molecules; SMD in the presence of 0 (**a’**), 1 (**b’**) and 2 (**c’**) explicit water molecules. Geometry optimization performed with CAM-B3LYP 6-311G+dp (C: grey; H: white; O: red).

Figure 2 compares the solvation volume obtained with SMD and IEFPCM for phenol. A well-finished cavity was obtained with SMD, which is recommended for calculation of the ΔG of solvation. In all cases, an ordered H-bonded closed network was observed around the −OH group [29,30]. As previously reported [12,17], the position of water molecules influences pKa; however, in this work, the optimized geometry with the minimum of energy (thus the most stable one) was selected to compute pKa.

**Figure 3:**
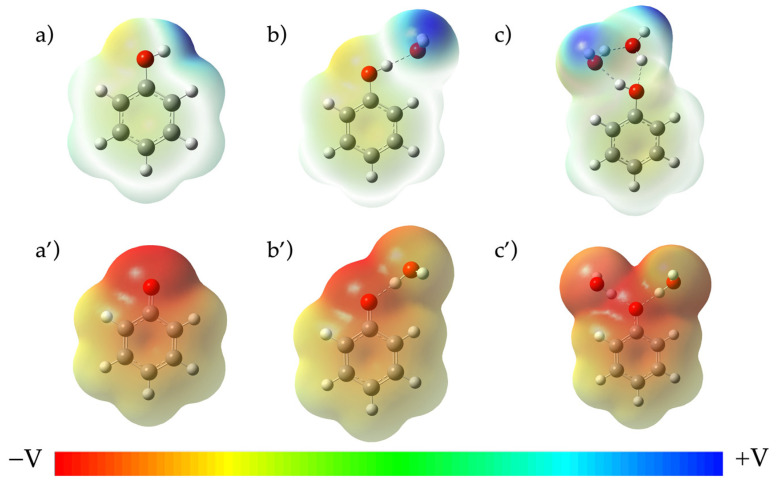
Isosurface of electrostatic potential plotted at a value of 0.0004 of phenol (top) and phenate (bottom) with: 0 (**a**,**a’**), 1 (**b**,**b’**), 2 (**c**,**c’**) explicit water molecules. Calculations were performed with CAM-B3LYP/6-311G+dp/SMD level of theory.

Figure 3 shows the influence of water molecules in the electronic distribution on the potential electronic map (PEM). As expected, the presence of explicit solvent affects the electronic distribution, especially for phenate, for which the negative charge is delocalized. Such representation realistically describes what happens in solution, where water molecules stabilize the negative charge through H-bond interactions.

To substantiate such methodology, a set of differently substituted phenols was selected. Theoretical pKa was determined using the best-performing of the previously tested computational methods, i.e., 1H_2_O/CAM-B3LYP/SMD, 2H_2_O/CAM-B3LYP/SMD, 2H_2_O/CAM-B3LYP/PCM, 2H_2_O/CAM-B3LYP/CPCM and 2H_2_O/B3LYP/SMD. With such functionals and solvation models, ΔpKa values lower than 0.3 were obtained for phenol, in addition to reliable results for thymol. Thus, an assorted range of phenolic compounds bearing at least two alkyl substituents with varying steric hindrance and phenols substituted with electron-donating or electron-withdrawing groups was selected (i.e., halogens, methoxy, cyano and nitro groups). Importantly, halogens, methoxy, cyano and nitro substituents were exclusively included at position 4 of the phenols to avoid intramolecular hydrogen bonding at the reaction center [16].

Table 4 shows the experimental and calculated pKa values of the selected compounds, and the ΔpKa as the difference between experimental and calculated pKa, the mean absolute error (MAE) and standard deviation (std. dev.) for each computational method are reported in Table 5.

**Figure 4:**
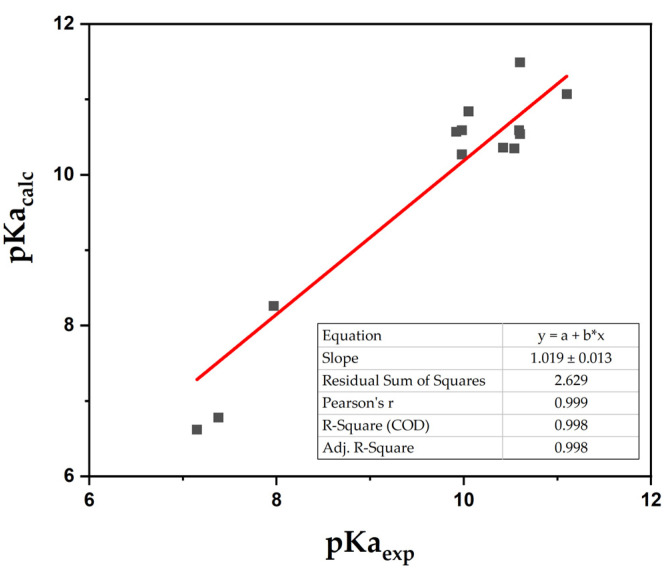
Calculated pKa vs. experimental pKa plot. pKa_calc_ was obtained using the 2H_2_O/CAM-B3LYP/SMD method.

Calculations performed with 1H_2_O/CAM-B3LYP/SMD led to satisfactory results. In particular, ΔpKa < 1.1 units (MAE = 0.72) was obtained for all dialkyl-substituted phenols, which is considered acceptable for theoretical pKa determinations. Satisfactory results were also achieved with halogens and methoxy substituents. However, considerable ΔpKa values were obtained with strong electron-withdrawing groups, such as -CN and -NO_2_, which significantly affect the electron density on the phenol ring. Conversely, good to excellent results were obtained with two explicit water molecules. In particular, the SMD solvation model achieved ΔpKa < 0.2 units for all dialkyl-substituted phenols, except for thymol, the pKa of which was overestimated, at 0.9 units. 4-Bromo- and 4-chlorothymol (4-bromo-2-isopropyl-5-methylphenol and 4-chloro-2-isopropyl-5-methylphenol, respectively) pKa were satisfactorily calculated with all solvation models, and SMD was suitable for the accurate determination of the pKa of phenols with complex substituents, such as -CN and -NO_2_ groups. In particular, the pKa of -NO_2_-substituted phenols was slightly underestimated (0.6 units), whereas with 4-hydroxybenzonitrile, a ΔpKa = 0.3 was obtained. Such remarkable results strengthen the applicability of this method [31,32,33]. Cyano and nitro substituents are rarely reported in computational studies on pKa determination and are usually associated with a high ΔpKa [34,35]. Thus, the simulation of their electronic effect is challenging. With all the tested computational methods, with the exception of 2H_2_O/CAM-B3LYP/SMD, pKa_calc_ values for phenols bearing -CN and -NO_2_ groups were largely underestimated (Table 4 and Table 5). On the contrary, 2H_2_O/CAM-B3LYP/SMD led to accurate results without the introduction of correction factors or mathematical regressions, as typically observed in the literature [16,28,29]. In addition, for all analyzed compounds, 2H_2_O/CAM-B3LYP/SMD led to the lowest MAE (0.39) and std. dev. (0.29), with maximum positive and negative deviations of 0.89 and −0.60, respectively, which is in the range of acceptable values for theoretical pKa determinations. Figure 4 shows the relationship obtained between pKa_calc_ and pKa_exp_ using such a computational method; the reported slope (1.019) is close to the ideal value (i.e., 1.000 for pKa_calc_ = pKa_exp_) [16], highlighting the accuracy of such a model, without requiring correction factors. 

**Figure 5:**
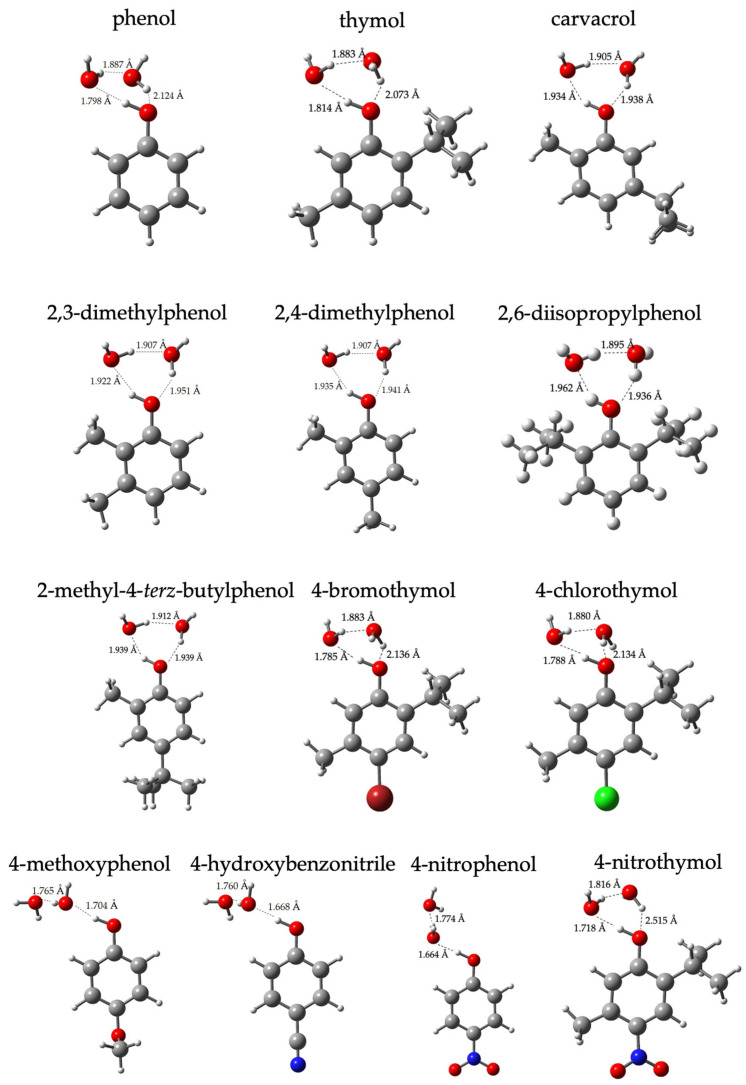
Optimized geometry of phenol derivatives in the presence of two explicit water molecules. Geometry optimization performed with CAM-B3LYP functional, 6-311G+dp basis set and SMD solvation model (C: grey; H: white; O: red; N: blue; Br: dark red; Cl: green).

The optimized geometries of the analyzed phenols in the presence of two explicit water molecules obtained with 2H_2_O/CAM-B3LYP/SMD computational model are reported in Figure 5. For phenol, dialkyl phenol derivatives and phenols bearing halogen substituents, an ordered H-bonded cage can be observed around the reaction center (i.e., −OH group), with H-bond lengths in the range of 1.765–2.136 Å. Conversely, in the case of 4-nitrophenol and 4-hydroxybenzonitrile, an open-cage was obtained, likely owing to the presence of the electron-withdrawing substituent, strengthening the H bond between the −OH group and the first water molecule (H-bond length < 1.67 Å) but reducing the availability of the oxygen lone pair. Therefore, the second water molecule is more than 4 Å from the −OH group, preventing an H bond. Unexpectedly, an open cage is also energetically favored in the case of 4-methoxyphenol, being ca. 1.24 kcal⋅mol^−1^ more stable than the ordered H-bonded cage.

## 3. Materials and Methods

### 3.1. Chemicals and Synthesis

Solvents and chemical reagents were purchased from Sigma Aldrich/Merck KgaA, Darmstadt, Germany, and were used without further purification. 4-Bromo-2-isopropyl-5-methylphenol (4-bromothymol) and 2-isopropyl-5-methyl-4-nitrophenol (4-nitrothymol) were synthesized and fully characterized (Supp. Info). ^1^H NMR experiments were carried out using a Bruker400 MHz Avance III spectrometer (Billerica, MA, USA). GC-MS analyses were performed with a GCMS QP2010 Ultra system (Shimadzu, Kyoto, Japan) at 70 eV ionization energy. The absorption spectra were recorded with a UV−Vis 2450 spectrophotometer (Shimadzu, Kyoto, Japan). For known compounds, experimental pKa values were obtained from the NIH (National Library of Medicine) through PubChem or ChemID*plus*.

### 3.2. Computational Method and Data Analysis

DFT calculations were performed using Gaussian 16 rev. A.03. [36]. All compounds were geometrically optimized in vacuum and in continuum solvent. Calculations were performed using wB97XD, B3PW91, B3LYP and CAM-B3LYP functionals with 6-311G+dp basis set. For calculations in solution, the following continuum solvation models were used: SMD (solvation model based on density), CPCM (conductor-like polarizable continuum model) and IEFPCM (integral equation formalism polarizable continuum model). For each functional and basis set, electronic energy was collected in the presence of zero, one or two explicit water molecules [37]. The starting geometries for the hydrated molecules were based on chemical intuition, as proposed by Cunningham and colleagues [17], and drawn using GaussView 6.0 software. All calculations parameters were set up for geometry optimization (true minima) of each species, avoiding negative frequencies. pKa values were computed at 298.15 K. Optimized cartesian coordinates are reported in Supp. Info.

### 3.3. Experimental pKa Determination

Buffer solutions with different pH values were prepared using varying KH_2_PO_4_, K_2_HPO_4_ and K_3_PO_4_ concentrations. The pH of each solution was checked with a glass electrode. A 10^−2^ M stock solution was prepared in methanol for the analyzed phenols (4-bromothymol and 4-nitrothymol). A volume of 30 μL of the stock solution was diluted in 3 mL of the buffer solutions at varying pH values, and the UV-vis absorption spectra were recorded. For all the tested compounds, the final concentration in the cuvette was 10^−4^ M. For data analysis, the absorbance values were reported as a function of pH at the appropriate wavelength; then, sigmoidal fitting was used to obtain the inflection point (Figures S1–S2), pKa_exp_ (4-bromothymol) = 9.92 ± 0.04; pKa_exp_ (4-nitrothymol) = 7.38 ± 0.06.

## 4. Conclusions

In this work, we developed an easy-to-use method based on the direct approach for computing pKa without introducing correction factors. Phenol and thymol were used as leading compounds of a set of molecules with various substituents on the aromatic ring. Four DFT functionals, three solvation models and a range of explicit water molecules (from zero to two) were compared.

For all analyzed phenols, 2H_2_O/CAM-B3LYP/6-311G+dp/SMD led to accurate results, with a mean absolute error of 0.37, which is an acceptable value for theoretical pKa determinations. In addition, this methodology achieved reliable results with nitro and cyano substituents, which are usually associated with very high ΔpKa values, proving that this method can be applied to a wide range of substituted phenols. Results were obtained without the introduction of any correction factors or mathematical regressions, making this approach a valid and accurate method for the direct calculation of the pKa of natural and synthetic phenols.

## Figures and Tables

**Table 1:** ΔpKa values calculated with no explicit water molecules.

Compound	pKa_(ref)_	B3LYP 6-311G^+^dp			B3PW91 6-311G^+^dp			WB97XD 6-311G^+^dp			CAM-B3LYP 6-311G^+^dp		
		SMD	CPCM	PCM	SMD	CPCM	PCM	SMD	CPCM	PCM	SMD	CPCM	PCM
Phenol	9.98	−11.24	−6.43	−6.45	11.33	−6.93	−6.95	−2.78	−7.13	−7.15	−3.60	−11.63	−8.12
Thymol	10.60	−10.16	−4.79	−4.84	12.43	−5.36	−5.40	−1.77	−5.55	−5.60	−2.63	−6.69	−6.71

**Table 2:** ΔpKa values calculated with one explicit water molecule.

Compound	pKa_(ref)_	B3LYP			B3PW91			WB97XD			CAM-B3LYP		
		SMD	CPCM	PCM	SMD	CPCM	PCM	SMD	CPCM	PCM	SMD	CPCM	PCM
Phenol	9.98	−0.69	−3.04	−3.26	−1.33	−3.70	−3.93	−1.26	−3.46	−3.67	−0.32	−2.50	−2.67
Thymol	10.60	−0.07	−1.97	−2.11	−1.17	−3.28	−3.49	−1.06	−2.96	−3.16	−0.06	−1.96	−1.97

**Table 3:** ΔpKa values calculated with two explicit water molecules.

Compound	pKa_(ref)_	B3LYP			B3PW91			WB97XD			CAM-B3LYP		
		SMD	PCM	CPCM	SMD	PCM	CPCM	SMD	PCM	CPCM	SMD	CPCM	PCM
Phenol	9,98	0.02	0.91	0.96	0.58	1.48	1.49	0.86	1.62	1.65	0.29	0.24	0.28
Thymol	10,60	−0.72	0.13	0.16	−0.27	1.08	1.10	0.44	1.14	1.17	0.89	−0.37	−0.18

**Table 4:** Calculated pKa for phenol derivatives with 6-311G+dp basis set.

Compound	pKa_ref_	CAM-B3LYP				B3LYP
		1H_2_O	2H_2_O	2H_2_O	2H_2_O	2H_2_O
		SMD	SMD	PCM	CPCM	SMD
		pKa_calc_	pKa_calc_	pKa_calc_	pKa_calc_	pKa_calc_
Phenol	**9.98**	9.35	10.27	10.23	10.15	9.95
2-isopropyl-5-methylphenol (thymol)	**10.60**	10.23	11.49	11.80	11.78	11.39
5-isopropyl-2-methylphenol (carvacrol)	**10.42**	9.59	10.36	11.19	11.15	9.89
2,3-dimethylphenol	**10.54**	9.45	10.35	11.04	11.01	9.87
2,4-dimethylphenol	**10.60**	9.74	10.54	11.32	11.29	9.99
2,6-diisopropylphenol	**11.10**	10.02	11.07	11.58	11.53	10.26
2-methyl-4-*terz*-butylphenol	**10.59**	10.41	10.59	11.31	11.27	10.03
4-bromo-2-isopropyl-5-methylphenol	**9.92**	9.31	10.57	10.42	10.38	9.90
4-chloro-2-isopropyl-5-methylphenol	**9.98**	9.34	10.59	10.61	10.58	10.08
4-methoxyphenol	**10.05**	10.84	10.84	11.02	10.97	11.14
4-hydroxybenzonitrile	**7.97**	6.25	8.26	6.55	6.54	7.73
4-nitrophenol	**7.15**	4.11	6.62	6.22	6.22	5.33
4-nitro-2-isopropyl-5-methylphenol	**7.38**	4.85	6.78	5.98	5.97	6.39

**Table 5:** ΔpKa for phenol derivatives with 6-311G+dp basis set.

Compound	pKa_ref_	CAM-B3LYP				B3LYP
		1H_2_O	2H_2_O	2H_2_O	2H_2_O	2H_2_O
		SMD	SMD	PCM	CPCM	SMD
		pKa_calc_	pKa_calc_	pKa_calc_	pKa_calc_	pKa_calc_
Phenol	**9.98**	−0.63	0.29	0.25	0.17	−0.03
2-isopropyl-5-methylphenol (thymol)	**10.60**	−0.37	0.89	1.20	1.18	0.79
5-isopropyl-2-methylphenol (carvacrol)	**10.42**	−0.83	0.06	0.77	0.73	−0.53
2,3-dimethylphenol	**10.54**	−1.09	−0.19	0.50	0.47	−0.67
2,4-dimethylphenol	**10.60**	−0.86	−0.06	0.72	0.69	−0.61
2,6-diisopropylphenol	**11.10**	−1.08	−0,03	0.48	0.43	−0.84
2-methyl-4-*terz*-butylphenol	**10.59**	−0.18	0.00	0.72	0.68	−0.56
4-bromo-2-isopropyl-5-methylphenol	**9.92**	−0.61	0.65	0.50	0.46	−0.02
4-chloro-2-isopropyl-5-methylphenol	**9.98**	−0.64	0.61	0.63	0.60	−0.10
4-methoxyphenol	**10.05**	0.79	0.79	0.97	0.92	1.09
4-hydroxybenzonitrile	**7.97**	−1.72	0.29	−1.42	−1.43	−0.24
4-nitrophenol	**7.15**	−3.04	−0.53	−0.93	−0.93	−1.82
4-nitro-2-isopropyl-5-methylphenol	**7.38**	−2.53	−0.60	−1.40	−1.41	−0.99
MAE		1.25	0.39	0.88	0.85	0.70
Std. dev.		0.97	0.30	0.45	0.47	0.53

## Data Availability

The data presented in this study are available upon request from the corresponding author.

## Supplementary Materials

The following supporting information can be downloaded at: https://www.mdpi.com/article/10.3390/molecules27238590/s1, Synthesis of 4-bromo-2-isopropyl-5-methylphenol (4-bromothymol); Synthesis of 2-isopropyl-5-methyl-4-nitrophenol (4-nitrothymol); Experimental pKa determination; Figure S1: UV-vis absorption spectra of 4-bromothymol at different pHs (left); sigmoidal fitting for 4-bromothymol pKa determination (right); Figure S2: UV-vis absorption spectra of 4-nitrothymol at different pHs (left); sigmoidal fitting for 4-nitrothymol pKa determination (right); Optimized Cartesian coordinates (in Angstroms). References [38,39,40] are cited in Supplementary Materials.

## Sample Availability

Samples of the compounds are available from the authors.
