# Supplementary material for: An Accurate Approach for Computational pKa Determination of Phenolic Compounds
Source: Molecules. 2022 Dec 6;27(23):8590. doi: 10.3390/molecules27238590 (PMC9736058; doi:10.3390/molecules27238590)
Supplement: Supplementary file 1 [file molecules-27-08590-s001.zip › molecules-2068170-supplementary.pdf]

Supporting Information

An Accurate Approach for Computational pKa Determination of Phenolic Compounds

Silvia Pezzola , Samuele Tarallo , Alessandro Iannini , Mariano Venanzi , Pierluca Galloni , Valeria Conte and Federica Sabuzi *

Department of Chemical Science and Technologies, University of Rome Tor Vergata

Contents

Synthetic procedures S2

Synthesis of 4-bromo-2-isopropyl-5-methylphenol (4-bromothymol). S2

Synthesis of 2-isopropyl-5-methyl-4-nitrophenol (4-nitrothymol). S2

Experimental pKa determination S3

Optimized cartesian coordinates (in Angstroms) S4

References S170

Synthetic procedures

Synthesis of 4-bromo-2-isopropyl-5-methylphenol (4-bromothymol). 4-bromothymol was synthesized according to a previously reported literature procedure [1,2]. Thymol (7.5 g, 50 mmol), and KBr (6.0 g, 50 mmol) were dissolved in 85 mL of a 25 mM aqueous solution of NH_4VO_3 (294 mg, 2.5 mmol). 10 mL of 10.4 mM aqueous solution of H_2O_2 (100 mmol) and 4 mL of $HClO_4$ 11.7 M were added, to obtain pH = 1. The mixture was stirred at room temperature for 24 h. Afterwards, reaction products were extracted with three portions of ethyl acetate; the organic phase was dried over anhydrous Na_2SO_4 and the solvent was evaporated. The product was crystallized by pentane[patent] and isolated as a white powder. Yield: 62%. MS (EI, 70 eV): *m/z* = 230 $[M^{Br81}]^{+\cdot}$; 228 $[M^{Br79}]^{+\cdot}$; 215 $[M^{Br81}\text{-}CH_3]^{+}$; 213 $[M^{Br79}\text{-}CH_3]^{+}$; 134 $[M\text{-}CH_3Br]^{+\cdot}$. ^{1}H-NMR (400 MHz, $CDCl_3$): d = 7.29 (s, 1 H), 6.64 (s, 1 H), 3.18–3.07 (m, 1 H, J = 6.9 Hz), 2.30 (s, 3 H), 1.23 (d, 6 H, J = 6.9 Hz) ppm.

Synthesis of 2-isopropyl-5-methyl-4-nitrophenol (4-nitrothymol). 4-nitrothymol was synthesized readjusting a previously reported literature procedure [3]. Thymol (1.5 g, 10 mmol) was dissolved in 25 mL of CH_2Cl_2. Afterwards, 2.3 mL of H_2O, 280 mg of tetrabutylammonium chloride (1 mmol) and 0.7 mL of HNO_3 65 % (10 mmol, 1 eq., 15% solution) were added and the mixture was stirred at room temperature for 30 mins. Organic phase was then separated from the aqueous phase and washed with water until neutrality. The organic phase was died over anhydrous Na_2SO_4 and the solvent was evaporated. The product was purified by chromatography column (SiO_2, ethyl acetate : petroleum ether 1 : 9 v :v), crystallized by pentane and isolated as a pale yellow solid. Yield: 19 %. MS (EI, 70 eV): *m/z* = 195 $[M]^{+\cdot}$; 180 $[M\text{-}CH_3]^{+}$; 134 $[M\text{-}CH_3NO_2]^{+\cdot}$. ^{1}H-NMR (400 MHz, $CDCl_3$): d = 8.06 (s, 1 H), 6.76 (s, 1 H), 3.22–3.32 (m, 1 H, J = 7.1 Hz), 2.62 (s, 3 H), 1.32 (d, 6 H, J = 6.8 Hz) ppm.

Experimental pKa determination

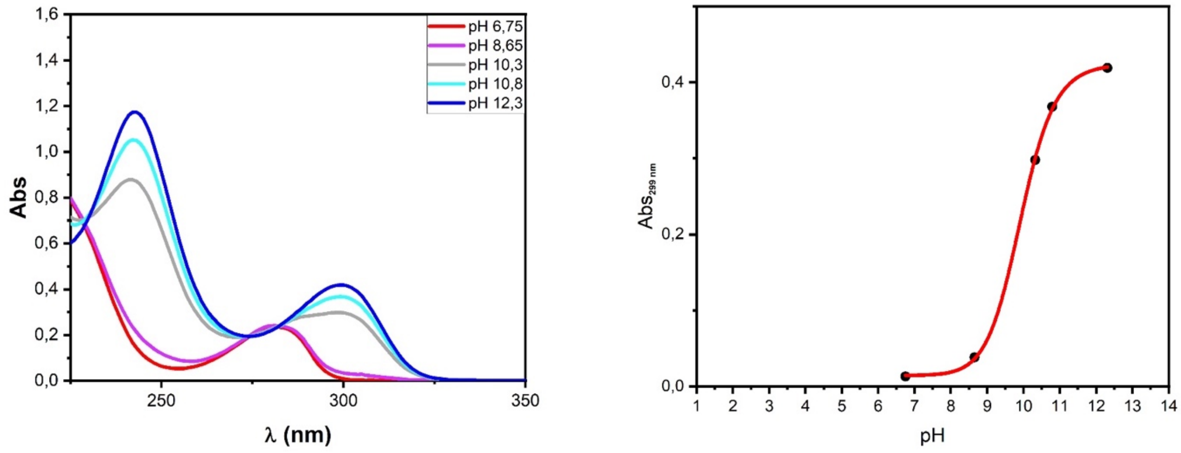

Figure. S1. UV-vis absorption spectra of 4-bromothymol at different pHs (left); sigmoidal fitting for 4-bromothymol pKa determination (right). $pKa_{exp} = 9.92 \pm 0.04$

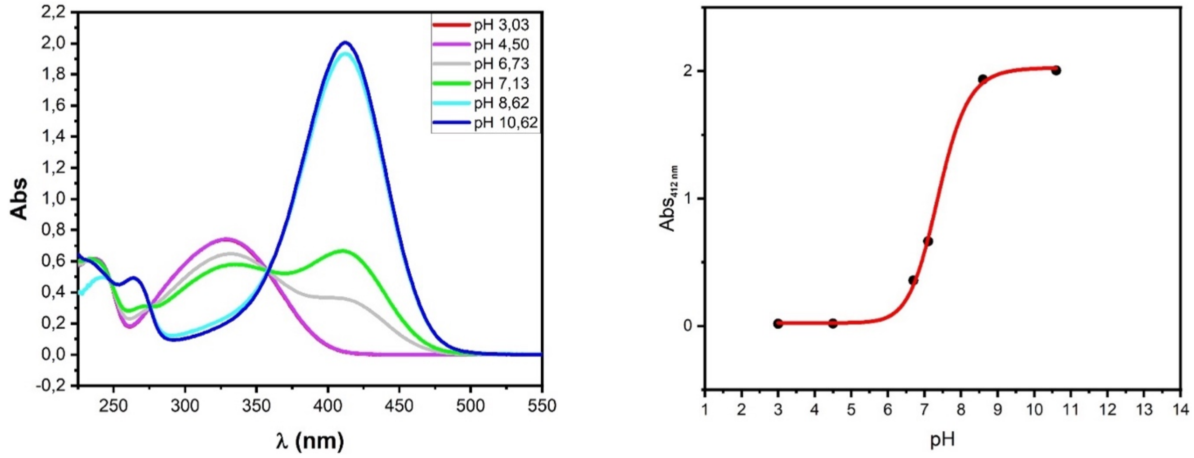

Figure. S2. UV-vis absorption spectra of 4-nitrothymol at different pHs (left); sigmoidal fitting for 4-nitrothymol pKa determination (right). $pKa_{exp} = 7.38 \pm 0.06$.

Optimized cartesian coordinates (in Angstroms)

- B3LYP 6-311G+dp/SMD

H_2O

Electronic Energy -76.471996
Stoichiometry H2O
Framework group CS[SG(H2O)]
Deg. of freedom 3
Full point group CS NOp 2
Largest Abelian subgroup CS NOp 2
Largest concise Abelian subgroup C1 NOp 1
Standard orientation:

Center Number	Atomic Number	Atomic Type	Coordinates (Angstroms) X	Y	Z
1	8	0	0.000000	0.118386	0.000000
2	1	0	0.762387	-0.473545	0.000000
3	1	0	-0.762387	-0.473545	0.000000

Rotational constants (GHZ): 805.7582180 431.3711005 280.9575394

OH^-

Electronic Energy -75.978626
Stoichiometry HO(1-)
Framework group C*V[C*(HO)]
Deg. of freedom 1
Full point group C*V NOp 4
Largest Abelian subgroup C2V NOp 4
Largest concise Abelian subgroup C1 NOp 1
Standard orientation:

Center Number	Atomic Number	Atomic Type	Coordinates (Angstroms) X	Y	Z
1	8	0	0.000000	0.000000	0.106682
2	1	0	0.000000	0.000000	-0.853456

Rotational constants (GHZ): 0.0000000 578.2316725 578.2316725

Phenol

Electronic Energy -307.568264
Stoichiometry C6H6O
Framework group C1[X(C6H6O)]
Deg. of freedom 33
Full point group C1 NOp 1
Largest Abelian subgroup C1 NOp 1
Largest concise Abelian subgroup C1 NOp 1
Standard orientation:

Center Number	Atomic Number	Atomic Type	Coordinates (Angstroms) X	Y	Z
1	6	0	1.179045	-1.186678	0.000140
2	6	0	-0.213063	-1.224759	0.000066
3	6	0	-0.936320	-0.028699	-0.000050
4	6	0	-0.269423	1.197857	-0.000094
5	6	0	1.125581	1.222247	-0.000019
6	6	0	1.857417	0.034302	0.000098
7	1	0	1.735819	-2.117310	0.000232
8	1	0	-0.748305	-2.167526	0.000099
9	1	0	-0.840118	2.121137	-0.000184
10	1	0	1.637968	2.177827	-0.000053
11	1	0	2.940851	0.057964	0.000156
12	8	0	-2.310733	-0.115848	-0.000112
13	1	0	-2.699761	0.769071	-0.000203

Rotational constants (GHZ): 5.6531192 2.6090086 1.7851378

Phenolate
Electronic Energy -307.093078
Stoichiometry C6H5O(1-)
Framework group C1[X(C6H5O)]
Deg. of freedom 30
Full point group C1 NOp 1
Largest Abelian subgroup C1 NOp 1
Largest concise Abelian subgroup C1 NOp 1
Standard orientation:

Center Number	Atomic Number	Atomic Type	Coordinates (Angstroms) X	Y	Z
1	6	0	1.100582	-1.200399	0.000004
2	6	0	-0.288737	-1.209534	0.000000
3	6	0	-1.061767	0.000025	-0.000051
4	6	0	-0.288740	1.209539	0.000000
5	6	0	1.100613	1.200376	0.000004
6	6	0	1.822011	-0.000001	-0.000010
7	1	0	1.635124	-2.146977	0.000016
8	1	0	-0.829749	-2.152940	0.000023
9	1	0	-0.829677	2.152988	0.000023
10	1	0	1.635129	2.146968	0.000016
11	1	0	2.905516	-0.000033	-0.000003
12	8	0	-2.352514	-0.000006	0.000031

Rotational constants (GHZ): 5.7216165 2.6877790 1.8287213

Thymol
Electronic Energy -464.866778
Stoichiometry C10H14O
Framework group C1[X(C10H14O)]
Deg. of freedom 69
Full point group C1 NOp 1
Largest Abelian subgroup C1 NOp 1
Largest concise Abelian subgroup C1 NOp 1
Standard orientation:

Center Number	Atomic Number	Atomic Type	Coordinates (Angstroms) X	Y	Z
1	6	0	-2.107781	-0.021454	-0.187754
2	1	0	-2.330293	0.790697	-0.887009
3	6	0	-0.591289	-0.134523	-0.117509
4	6	0	0.082994	-1.342778	0.056621
5	6	0	0.203688	1.022628	-0.199970
6	6	0	1.474561	-1.414470	0.145178
7	1	0	-0.488081	-2.261457	0.120563
8	6	0	1.592261	0.960132	-0.116994
9	6	0	2.252007	-0.260774	0.058190
10	1	0	1.953923	-2.378254	0.278937
11	1	0	2.170042	1.879284	-0.190230
12	6	0	-2.688318	0.387177	1.181902
13	1	0	-3.771361	0.530488	1.114376
14	1	0	-2.494853	-0.388161	1.929998
15	1	0	-2.244641	1.320169	1.535074
16	6	0	-2.797510	-1.287372	-0.714567
17	1	0	-3.866133	-1.099279	-0.850843
18	1	0	-2.384745	-1.600540	-1.677317
19	1	0	-2.704762	-2.125319	-0.016925
20	6	0	3.758372	-0.311220	0.145996
21	1	0	4.223821	0.082994	-0.762945
22	1	0	4.128464	0.285502	0.985718
23	1	0	4.110783	-1.335196	0.283795
24	8	0	-0.447127	2.222964	-0.366657
25	1	0	0.200934	2.931279	-0.436494

Rotational constants (GHZ): 2.0317868 0.7327491 0.5894444

Thymolate
Electronic Energy-464.389191
Stoichiometry C10H13O(1-)
Framework group C1[X(C10H13O)]
Deg. of freedom 66
Full point group C1 NOp 1
Largest Abelian subgroup C1 NOp 1
Largest concise Abelian subgroup C1 NOp 1
Standard orientation:

Center Number	Atomic Number	Atomic Type	Coordinates (Angstroms) X	Y	Z
1	6	0	-2.094908	-0.022872	-0.209917
2	1	0	-2.295056	0.750589	-0.961864
3	6	0	-0.582297	-0.124388	-0.120879
4	6	0	0.095202	-1.320008	0.077991
5	6	0	0.160141	1.124495	-0.227321
6	6	0	1.493530	-1.392901	0.179378
7	1	0	-0.470515	-2.246291	0.160098
8	6	0	1.590270	0.998644	-0.126821
9	6	0	2.243462	-0.212688	0.070076
10	1	0	1.984836	-2.349371	0.336542
11	1	0	2.161919	1.921138	-0.207851
12	6	0	-2.694270	0.502475	1.111187
13	1	0	-3.775901	0.673850	1.019208
14	1	0	-2.530798	-0.216820	1.922924
15	1	0	-2.216379	1.447113	1.376807
16	6	0	-2.801172	-1.314164	-0.645799
17	1	0	-3.874859	-1.136668	-0.782657
18	1	0	-2.395514	-1.691977	-1.589681
19	1	0	-2.697664	-2.109332	0.101916
20	6	0	3.757364	-0.265144	0.123502
21	1	0	4.190224	-0.422710	-0.873204
22	1	0	4.174547	0.668486	0.514179
23	1	0	4.106727	-1.084582	0.761533
24	8	0	-0.420687	2.244485	-0.395791

Rotational constants (GHZ): 2.0872081 0.7370443 0.5919865

- B3LYP 6-311G+dp/PCM

H_2O
Electronic Energy -76.466413
Stoichiometry H2O
Framework group CS[SG(H2O)]
Deg. of freedom 3
Full point group CS NOp 2
Largest Abelian subgroup CS NOp 2
Largest concise Abelian subgroup C1 NOp 1
Standard orientation:

Center Number	Atomic Number	Atomic Type	Coordinates (Angstroms) X	Y	Z
1	8	0	0.000000	0.115552	0.000000
2	1	0	0.768344	-0.462208	0.000000
3	1	0	-0.768344	-0.462205	0.000000

Rotational constants (GHZ): 845.7772941 424.7084360 282.7334013

OH^-
Electronic Energy -75.959302
Stoichiometry HO(1-)
Framework group C*V[C*(HO)]
Deg. of freedom 1
Full point group C*V NOp 4
Largest Abelian subgroup C2V NOp 4
Largest concise Abelian subgroup C1 NOp 1

Standard orientation:

Center Number	Atomic Number	Atomic Type	Coordinates (Angstroms) X	Y	Z
1	8	0	0.000000	0.000000	0.106682
2	1	0	0.000000	0.000000	-0.853456

Rotational constants (GHZ): 0.0000000 578.2316725 578.2316725

Phenol
Electronic Energy -307.565572
Stoichiometry C6H6O
Framework group C1[X(C6H6O)]
Deg. of freedom 33
Full point group C1 NOp 1
Largest Abelian subgroup C1 NOp 1
Largest concise Abelian subgroup C1 NOp 1
Standard orientation:

Center Number	Atomic Number	Atomic Type	Coordinates (Angstroms) X	Y	Z
1	6	0	1.169725	-1.188837	0.000140
2	6	0	-0.220965	-1.221569	0.000068
3	6	0	-0.938050	-0.024019	-0.000048
4	6	0	-0.262697	1.197631	-0.000091
5	6	0	1.131378	1.217225	-0.000020
6	6	0	1.855157	0.027156	0.000096
7	1	0	1.721963	-2.121987	0.000229
8	1	0	-0.764029	-2.158909	0.000100
9	1	0	-0.822750	2.128459	-0.000179
10	1	0	1.649034	2.169921	-0.000055
11	1	0	2.938359	0.045552	0.000151
12	8	0	-2.305367	-0.110272	-0.000112
13	1	0	-2.686933	0.773622	-0.000213

Rotational constants (GHZ): 5.6675172 2.6180498 1.7908059

Phenolate
Electronic Energy -307.086020
Stoichiometry C6H5O(1-)
Framework group C1[X(C6H5O)]
Deg. of freedom 30
Full point group C1 NOp 1
Largest Abelian subgroup C1 NOp 1
Largest concise Abelian subgroup C1 NOp 1
Standard orientation:

Center Number	Atomic Number	Atomic Type	Coordinates (Angstroms) X	Y	Z
1	6	0	1.101013	-1.200385	0.000003
2	6	0	-0.288354	-1.210289	-0.000005
3	6	0	-1.069337	0.000025	-0.000062
4	6	0	-0.288358	1.210294	-0.000005
5	6	0	1.101041	1.200362	0.000003
6	6	0	1.824402	0.000000	-0.000011
7	1	0	1.636379	-2.147279	0.000019
8	1	0	-0.828219	-2.154432	0.000022
9	1	0	-0.828150	2.154479	0.000022
10	1	0	1.636380	2.147271	0.000020
11	1	0	2.908177	-0.000030	0.000002
12	8	0	-2.350877	-0.000007	0.000046

Rotational constants (GHZ): 5.7178249 2.6847752 1.8269434

Thymol
Electronic Energy -464.866380
Stoichiometry C10H14O
Framework group C1[X(C10H14O)]

Deg. of freedom 69
Full point group C1 NOp 1
Largest Abelian subgroup C1 NOp 1
Largest concise Abelian subgroup C1 NOp 1
Standard orientation:

Center Number	Atomic Number	Atomic Type	Coordinates (Angstroms) X	Y	Z
1	6	0	-2.110727	-0.013677	-0.182925
2	1	0	-2.337036	0.812554	-0.864077
3	6	0	-0.593203	-0.127329	-0.116249
4	6	0	0.076045	-1.340530	0.053663
5	6	0	0.208145	1.028414	-0.196405
6	6	0	1.468316	-1.418700	0.141952
7	1	0	-0.499014	-2.256978	0.116899
8	6	0	1.597494	0.959127	-0.114527
9	6	0	2.251397	-0.266445	0.057957
10	1	0	1.943097	-2.385242	0.273381
11	1	0	2.178418	1.875197	-0.186816
12	6	0	-2.691902	0.360619	1.196540
13	1	0	-3.773261	0.515373	1.129456
14	1	0	-2.508830	-0.439405	1.920967
15	1	0	-2.240933	1.278135	1.580785
16	6	0	-2.800366	-1.267978	-0.736872
17	1	0	-3.870043	-1.078245	-0.861945
18	1	0	-2.392767	-1.555650	-1.709852
19	1	0	-2.699889	-2.122481	-0.060984
20	6	0	3.757644	-0.323398	0.145540
21	1	0	4.223308	0.073335	-0.761833
22	1	0	4.129037	0.272067	0.985197
23	1	0	4.105994	-1.349125	0.281035
24	8	0	-0.431888	2.234662	-0.363136
25	1	0	0.219957	2.942556	-0.429162

Rotational constants (GHZ): 2.0231982 0.7313600 0.5890827

Thymolate
Electronic Energy -464.383413
Stoichiometry C10H13O(1-)
Framework group C1[X(C10H13O)]
Deg. of freedom 66
Full point group C1 NOp 1
Largest Abelian subgroup C1 NOp 1
Largest concise Abelian subgroup C1 NOp 1
Standard orientation:

Center Number	Atomic Number	Atomic Type	Coordinates (Angstroms) X	Y	Z
1	6	0	-2.094908	-0.022872	-0.209917
2	1	0	-2.295056	0.750589	-0.961864
3	6	0	-0.582297	-0.124388	-0.120879
4	6	0	0.095202	-1.320008	0.077991
5	6	0	0.160141	1.124495	-0.227321
6	6	0	1.493530	-1.392901	0.179378
7	1	0	-0.470515	-2.246291	0.160098
8	6	0	1.590270	0.998644	-0.126821
9	6	0	2.243462	-0.212688	0.070076
10	1	0	1.984836	-2.349371	0.336542
11	1	0	2.161919	1.921138	-0.207851
12	6	0	-2.694270	0.502475	1.111187
13	1	0	-3.775901	0.673850	1.019208
14	1	0	-2.530798	-0.216820	1.922924
15	1	0	-2.216379	1.447113	1.376807
16	6	0	-2.801172	-1.314164	-0.645799
17	1	0	-3.874859	-1.136668	-0.782657
18	1	0	-2.395514	-1.691977	-1.589681
19	1	0	-2.697664	-2.109332	0.101916
20	6	0	3.757364	-0.265144	0.123502
21	1	0	4.190224	-0.422710	-0.873204
22	1	0	4.174547	0.668486	0.514179

23	1	0	4.106727	-1.084582	0.761533
24	8	0	-0.420687	2.244485	-0.395791

Rotational constants (GHZ): 2.0872081 0.7370443 0.5919865

- B3LYP 6-311G+dp/CPCM

H_2O

Electronic Energy -76.466413
Stoichiometry H2O
Framework group CS[SG(H2O)]
Deg. of freedom 3
Full point group CS NOp 2
Largest Abelian subgroup CS NOp 2
Largest concise Abelian subgroup C1 NOp 1
Standard orientation:

Center Number	Atomic Number	Atomic Type	Coordinates (Angstroms) X	Y	Z
1	8	0	0.000000	0.117985	0.000000
2	1	0	0.762188	-0.471941	0.000000
3	1	0	-0.762188	-0.471941	0.000000

Rotational constants (GHZ): 811.2464689 431.5960605 281.7177332

OH^-

Electronic Energy -75.959356
Stoichiometry HO(1-)
Framework group C*V[C*(HO)]
Deg. of freedom 1
Full point group C*V NOp 4
Largest Abelian subgroup C2V NOp 4
Largest concise Abelian subgroup C1 NOp 1
Standard orientation:

Center Number	Atomic Number	Atomic Type	Coordinates (Angstroms) X	Y	Z
1	8	0	0.000000	0.000000	0.107067
2	1	0	0.000000	0.000000	-0.856533

Rotational constants (GHZ): 0.0000000 574.0845324 574.0845324

Phenol

Electronic Energy -307.565635
Stoichiometry C6H6O
Framework group C1[X(C6H6O)]
Deg. of freedom 33
Full point group C1 NOp 1
Largest Abelian subgroup C1 NOp 1
Largest concise Abelian subgroup C1 NOp 1
Standard orientation:

Center Number	Atomic Number	Atomic Type	Coordinates (Angstroms) X	Y	Z
1	6	0	1.169725	-1.188837	0.000140
2	6	0	-0.220965	-1.221569	0.000068
3	6	0	-0.938050	-0.024019	-0.000048
4	6	0	-0.262697	1.197631	-0.000091
5	6	0	1.131378	1.217225	-0.000020
6	6	0	1.855157	0.027156	0.000096
7	1	0	1.721963	-2.121987	0.000229
8	1	0	-0.764029	-2.158909	0.000100
9	1	0	-0.822750	2.128459	-0.000179
10	1	0	1.649034	2.169921	-0.000055
11	1	0	2.938359	0.045552	0.000151
12	8	0	-2.305367	-0.110272	-0.000112
13	1	0	-2.686933	0.773622	-0.000213

Rotational constants (GHZ): 5.6675172 2.6180498 1.7908059

Phenolate
Electronic Energy -307.086129
Stoichiometry C6H5O(1-)
Framework group C1[X(C6H5O)]
Deg. of freedom 30
Full point group C1 NOp 1
Largest Abelian subgroup C1 NOp 1
Largest concise Abelian subgroup C1 NOp 1
Standard orientation:

Center Number	Atomic Number	Atomic Type	Coordinates (Angstroms) X	Y	Z
1	6	0	1.101592	-1.200744	-0.000018
2	6	0	-0.289697	-1.208834	0.000019
3	6	0	-1.062832	0.000002	0.000214
4	6	0	-0.289698	1.208837	0.000019
5	6	0	1.101595	1.200740	-0.000017
6	6	0	1.822963	0.000000	0.000021
7	1	0	1.635932	-2.147529	-0.000083
8	1	0	-0.827076	-2.153844	-0.000072
9	1	0	-0.827069	2.153853	-0.000071
10	1	0	1.635931	2.147528	-0.000083
11	1	0	2.907122	-0.000003	-0.000020
12	8	0	-2.353548	-0.000002	-0.000138

Rotational constants (GHZ): 5.7221218 2.6846711 1.8273336

Thymol
Electronic Energy -464.866446
Stoichiometry C10H14O
Framework group C1[X(C10H14O)]
Deg. of freedom 69
Full point group C1 NOp 1
Largest Abelian subgroup C1 NOp 1
Largest concise Abelian subgroup C1 NOp 1
Standard orientation:

Center Number	Atomic Number	Atomic Type	Coordinates (Angstroms) X	Y	Z
1	6	0	-2.110842	-0.006333	-0.176082
2	1	0	-2.338244	0.839387	-0.831946
3	6	0	-0.593605	-0.121771	-0.114357
4	6	0	0.072619	-1.336780	0.056315
5	6	0	0.209734	1.031333	-0.194530
6	6	0	1.464525	-1.418997	0.142203
7	1	0	-0.505177	-2.251382	0.121395
8	6	0	1.599556	0.958266	-0.114999
9	6	0	2.250472	-0.268628	0.056187
10	1	0	1.937116	-2.386600	0.273288
11	1	0	2.182354	1.873038	-0.187449
12	6	0	-2.689883	0.326998	1.214627
13	1	0	-3.771788	0.480747	1.154797
14	1	0	-2.502484	-0.492453	1.915902
15	1	0	-2.240426	1.234445	1.624595
16	6	0	-2.800983	-1.244057	-0.767381
17	1	0	-3.870553	-1.050119	-0.886622
18	1	0	-2.393072	-1.501507	-1.748685
19	1	0	-2.700426	-2.118775	-0.118208
20	6	0	3.756941	-0.329671	0.140692
21	1	0	4.221414	0.065686	-0.767920
22	1	0	4.131413	0.266111	0.978773
23	1	0	4.102609	-1.356110	0.276254
24	8	0	-0.428558	2.238644	-0.356048
25	1	0	0.224535	2.946227	-0.411836

Rotational constants (GHZ): 2.0214315 0.7304210 0.5900148

Thymolate
Electronic Energy-464.383580
Stoichiometry C10H13O(1-)
Framework group C1[X(C10H13O)]
Deg. of freedom 66
Full point group C1 NOp 1
Largest Abelian subgroup C1 NOp 1
Largest concise Abelian subgroup C1 NOp 1
Standard orientation:

Center Number	Atomic Number	Atomic Type	Coordinates (Angstroms) X	Y	Z
1	6	0	-2.094908	-0.022872	-0.209917
2	1	0	-2.295056	0.750589	-0.961864
3	6	0	-0.582297	-0.124388	-0.120879
4	6	0	0.095202	-1.320008	0.077991
5	6	0	0.160141	1.124495	-0.227321
6	6	0	1.493530	-1.392901	0.179378
7	1	0	-0.470515	-2.246291	0.160098
8	6	0	1.590270	0.998644	-0.126821
9	6	0	2.243462	-0.212688	0.070076
10	1	0	1.984836	-2.349371	0.336542
11	1	0	2.161919	1.921138	-0.207851
12	6	0	-2.694270	0.502475	1.111187
13	1	0	-3.775901	0.673850	1.019208
14	1	0	-2.530798	-0.216820	1.922924
15	1	0	-2.216379	1.447113	1.376807
16	6	0	-2.801172	-1.314164	-0.645799
17	1	0	-3.874859	-1.136668	-0.782657
18	1	0	-2.395514	-1.691977	-1.589681
19	1	0	-2.697664	-2.109332	0.101916
20	6	0	3.757364	-0.265144	0.123502
21	1	0	4.190224	-0.422710	-0.873204
22	1	0	4.174547	0.668486	0.514179
23	1	0	4.106727	-1.084582	0.761533
24	8	0	-0.420687	2.244485	-0.395791

Rotational constants (GHZ): 2.0872081 0.7370443 0.5919865

- B3PW91 6-311G+dp/SMD

H_2O
Electronic Energy -76.441720
Stoichiometry H2O
Framework group CS[SG(H2O)]
Deg. of freedom 3
Full point group CS NOp 2
Largest Abelian subgroup CS NOp 2
Largest concise Abelian subgroup C1 NOp 1
Standard orientation:

Center Number	Atomic Number	Atomic Type	Coordinates (Angstroms) X	Y	Z
1	8	0	0.000000	0.115552	0.000000
2	1	0	0.768344	-0.462208	0.000000
3	1	0	-0.768344	-0.462205	0.000000

Rotational constants (GHZ): 845.7772941 424.7084360 282.7334013

OH^-
Electronic Energy -75.944505
Stoichiometry HO(1-)
Framework group C*V[C*(HO)]
Deg. of freedom 1
Full point group C*V NOp 4
Largest Abelian subgroup C2V NOp 4
Largest concise Abelian subgroup C1 NOp 1

Standard orientation:

Center Number	Atomic Number	Atomic Type	Coordinates (Angstroms) X	Y	Z
1	8	0	0.000000	0.000000	0.106682
2	1	0	0.000000	0.000000	-0.853456

Rotational constants (GHZ): 0.0000000 578.2316725 578.2316725

Phenol
Electronic Energy -307.444461
Stoichiometry C6H6O
Framework group C1[X(C6H6O)]
Deg. of freedom 33
Full point group C1 NOp 1
Largest Abelian subgroup C1 NOp 1
Largest concise Abelian subgroup C1 NOp 1
Standard orientation:

Center Number	Atomic Number	Atomic Type	Coordinates (Angstroms) X	Y	Z
1	6	0	1.168101	-1.186116	0.000138
2	6	0	-0.220169	-1.219976	0.000067
3	6	0	-0.939066	-0.025077	-0.000047
4	6	0	-0.263529	1.195022	-0.000091
5	6	0	1.127883	1.215468	-0.000021
6	6	0	1.851976	0.028107	0.000095
7	1	0	1.721218	-2.119945	0.000227
8	1	0	-0.762720	-2.158768	0.000098
9	1	0	-0.824807	2.126490	-0.000180
10	1	0	1.645039	2.169605	-0.000056
11	1	0	2.936100	0.047458	0.000150
12	8	0	-2.298757	-0.110357	-0.000108
13	1	0	-2.675950	0.773447	-0.000219

Rotational constants (GHZ): 5.6846147 2.6293820 1.7978145

Phenolate
Electronic Energy -306.967570
Stoichiometry C6H5O(1-)
Framework group C1[X(C6H5O)]
Deg. of freedom 30
Full point group C1 NOp 1
Largest Abelian subgroup C1 NOp 1
Largest concise Abelian subgroup C1 NOp 1
Standard orientation:

Center Number	Atomic Number	Atomic Type	Coordinates (Angstroms) X	Y	Z
1	6	0	-1.098429	-1.197480	0.000008
2	6	0	0.287144	-1.208693	-0.000006
3	6	0	1.077535	0.000050	-0.000058
4	6	0	0.287157	1.208713	-0.000005
5	6	0	-1.098480	1.197438	0.000006
6	6	0	-1.825642	-0.000005	0.000004
7	1	0	-1.635179	-2.147169	0.000029
8	1	0	0.831125	-2.151400	0.000005
9	1	0	0.830978	2.151511	0.000009
10	1	0	-1.635190	2.147147	0.000025
11	1	0	-2.912018	-0.000060	-0.000008
12	8	0	2.343072	-0.000021	0.000031

Rotational constants (GHZ): 5.7364512 2.6911837 1.8318121

Thymol
Electronic Energy -464.685962
Stoichiometry C10H14O
Framework group C1[X(C10H14O)]
Deg. of freedom 69

Full point group C1 NOp 1
Largest Abelian subgroup C1 NOp 1
Largest concise Abelian subgroup C1 NOp 1
Standard orientation:

Center Number	Atomic Number	Atomic Type	Coordinates (Angstroms) X	Y	Z
1	6	0	-2.102627	-0.021835	-0.188280
2	1	0	-2.325004	0.788879	-0.891143
3	6	0	-0.591866	-0.133928	-0.118531
4	6	0	0.081206	-1.340220	0.054001
5	6	0	0.200576	1.022486	-0.199677
6	6	0	1.470189	-1.412258	0.143141
7	1	0	-0.492049	-2.259159	0.115705
8	6	0	1.587258	0.959079	-0.115885
9	6	0	2.246232	-0.259857	0.058395
10	1	0	1.950590	-2.376760	0.275871
11	1	0	2.166149	1.878778	-0.188297
12	6	0	-2.676731	0.389226	1.176345
13	1	0	-3.760682	0.531026	1.113273
14	1	0	-2.478978	-0.383735	1.926394
15	1	0	-2.232574	1.324108	1.525527
16	6	0	-2.787632	-1.286353	-0.706741
17	1	0	-3.856712	-1.100949	-0.846755
18	1	0	-2.372168	-1.606254	-1.666669
19	1	0	-2.696380	-2.119637	-0.002397
20	6	0	3.747152	-0.310223	0.146704
21	1	0	4.213197	0.085536	-0.761648
22	1	0	4.116762	0.285339	0.987886
23	1	0	4.099810	-1.334714	0.283149
24	8	0	-0.447195	2.216267	-0.366631
25	1	0	0.203048	2.920706	-0.434673

Rotational constants (GHZ): 2.0412376 0.7375072 0.5927874

Thymolate
Electronic Energy -464.206974
Stoichiometry C10H13O(1-)
Framework group C1[X(C10H13O)]
Deg. of freedom 66
Full point group C1 NOp 1
Largest Abelian subgroup C1 NOp 1
Largest concise Abelian subgroup C1 NOp 1
Standard orientation:

Center Number	Atomic Number	Atomic Type	Coordinates (Angstroms) X	Y	Z
1	6	0	-2.088528	-0.026439	-0.210929
2	1	0	-2.286134	0.742931	-0.969841
3	6	0	-0.581453	-0.127125	-0.122350
4	6	0	0.096100	-1.320625	0.074286
5	6	0	0.154670	1.121654	-0.227461
6	6	0	1.491883	-1.391276	0.176099
7	1	0	-0.470759	-2.247901	0.153827
8	6	0	1.582076	0.998057	-0.125332
9	6	0	2.237320	-0.210475	0.069956
10	1	0	1.986495	-2.347424	0.331395
11	1	0	2.153402	1.922127	-0.204809
12	6	0	-2.678407	0.510484	1.102160
13	1	0	-3.760794	0.682143	1.016151
14	1	0	-2.509106	-0.200139	1.920808
15	1	0	-2.196291	1.458040	1.352831
16	6	0	-2.790980	-1.317269	-0.631066
17	1	0	-3.865312	-1.143590	-0.770900
18	1	0	-2.383401	-1.706671	-1.570023
19	1	0	-2.687137	-2.103273	0.127165
20	6	0	3.746001	-0.258514	0.124620
21	1	0	4.179481	-0.418377	-0.871747
22	1	0	4.160468	0.677979	0.512290

23	1	0	4.097583	-1.075040	0.765739
24	8	0	-0.428825	2.236046	-0.396598

Rotational constants (GHZ): 2.0970161 0.7426100 0.5955077

- B3PW91 6-311G+dp/PCM

H_2O
Electronic Energy -76.436075
Stoichiometry H2O
Framework group CS[SG(H2O)]
Deg. of freedom 3
Full point group CS NOp 2
Largest Abelian subgroup CS NOp 2
Largest concise Abelian subgroup C1 NOp 1
Standard orientation:

Center Number	Atomic Number	Atomic Type	Coordinates (Angstroms) X	Y	Z
1	8	0	0.000000	0.115552	0.000000
2	1	0	0.768344	-0.462208	0.000000
3	1	0	-0.768344	-0.462205	0.000000

Rotational constants (GHZ): 845.7772941 424.7084360 282.7334013

OH^-
Electronic Energy -75.924215
Stoichiometry HO(1-)
Framework group C*V[C*(HO)]
Deg. of freedom 1
Full point group C*V NOp 4
Largest Abelian subgroup C2V NOp 4
Largest concise Abelian subgroup C1 NOp 1
Standard orientation:

Center Number	Atomic Number	Atomic Type	Coordinates (Angstroms) X	Y	Z
1	8	0	0.000000	0.000000	0.106682
2	1	0	0.000000	0.000000	-0.853456

Rotational constants (GHZ): 0.0000000 578.2316725 578.2316725

Phenol
Electronic Energy -307.441667
Stoichiometry C6H6O
Framework group C1[X(C6H6O)]
Deg. of freedom 33
Full point group C1 NOp 1
Largest Abelian subgroup C1 NOp 1
Largest concise Abelian subgroup C1 NOp 1
Standard orientation:

Center Number	Atomic Number	Atomic Type	Coordinates (Angstroms) X	Y	Z
1	6	0	1.168101	-1.186116	0.000138
2	6	0	-0.220169	-1.219976	0.000067
3	6	0	-0.939066	-0.025077	-0.000047
4	6	0	-0.263529	1.195022	-0.000091
5	6	0	1.127883	1.215468	-0.000021
6	6	0	1.851976	0.028107	0.000095
7	1	0	1.721218	-2.119945	0.000227
8	1	0	-0.762720	-2.158768	0.000098
9	1	0	-0.824807	2.126490	-0.000180
10	1	0	1.645039	2.169605	-0.000056
11	1	0	2.936100	0.047458	0.000150
12	8	0	-2.298757	-0.110357	-0.000108

13	1	0	-2.675950	0.773447	-0.000219

Rotational constants (GHZ): 5.6846147 2.6293820 1.7978145

Phenolate

Electronic Energy -306.959968
Stoichiometry C6H5O(1-)
Framework group C1[X(C6H5O)]
Deg. of freedom 30
Full point group C1 NOp 1
Largest Abelian subgroup C1 NOp 1
Largest concise Abelian subgroup C1 NOp 1
Standard orientation:

Center Number	Atomic Number	Atomic Type	Coordinates (Angstroms) X	Y	Z
1	6	0	1.099816	-1.198446	0.000037
2	6	0	-0.289275	-1.206479	-0.000063
3	6	0	-1.063417	0.000001	-0.000597
4	6	0	-0.289276	1.206480	-0.000063
5	6	0	1.099817	1.198445	0.000037
6	6	0	1.820982	0.000000	-0.000060
7	1	0	1.634270	-2.146549	0.000217
8	1	0	-0.826676	-2.152720	0.000174
9	1	0	-0.826673	2.152723	0.000174
10	1	0	1.634269	2.146549	0.000217
11	1	0	2.906145	-0.000001	0.000032
12	8	0	-2.349152	-0.000001	0.000429

Rotational constants (GHZ): 5.7407805 2.6921047 1.8326803

Thymol

Electronic Energy -464.685322
Stoichiometry C10H14O
Framework group C1[X(C10H14O)]
Deg. of freedom 69
Full point group C1 NOp 1
Largest Abelian subgroup C1 NOp 1
Largest concise Abelian subgroup C1 NOp 1
Standard orientation:

Center Number	Atomic Number	Atomic Type	Coordinates (Angstroms) X	Y	Z
1	6	0	-2.105545	-0.007747	-0.177563
2	1	0	-2.332779	0.834326	-0.840281
3	6	0	-0.593988	-0.122067	-0.116110
4	6	0	0.071516	-1.334790	0.053361
5	6	0	0.206270	1.031008	-0.195069
6	6	0	1.461005	-1.416737	0.140333
7	1	0	-0.507996	-2.250000	0.116166
8	6	0	1.594341	0.957485	-0.114336
9	6	0	2.244989	-0.267222	0.056552
10	1	0	1.935066	-2.384870	0.270793
11	1	0	2.177845	1.873116	-0.186056
12	6	0	-2.677571	0.334008	1.207143
13	1	0	-3.760227	0.487222	1.151145
14	1	0	-2.487271	-0.481198	1.913348
15	1	0	-2.226466	1.244385	1.610024
16	6	0	-2.791635	-1.246582	-0.755063
17	1	0	-3.861724	-1.055188	-0.878054
18	1	0	-2.382163	-1.515228	-1.733268
19	1	0	-2.692470	-2.114538	-0.095573
20	6	0	3.746041	-0.327421	0.142170
21	1	0	4.211391	0.069735	-0.765629
22	1	0	4.119501	0.267728	0.981616
23	1	0	4.092530	-1.354159	0.276729
24	8	0	-0.430124	2.230403	-0.357220
25	1	0	0.223226	2.935829	-0.411709

Rotational constants (GHZ): 2.0314549 0.7352444 0.5932521

Thymolate
Electronic Energy -464.200492
Stoichiometry C10H13O(1-)
Framework group C1[X(C10H13O)]
Deg. of freedom 66
Full point group C1 NOp 1
Largest Abelian subgroup C1 NOp 1
Largest concise Abelian subgroup C1 NOp 1
Standard orientation:

Center Number	Atomic Number	Atomic Type	Coordinates (Angstroms) X	Y	Z
1	6	0	-2.088528	-0.026439	-0.210929
2	1	0	-2.286134	0.742931	-0.969841
3	6	0	-0.581453	-0.127125	-0.122350
4	6	0	0.096100	-1.320625	0.074286
5	6	0	0.154670	1.121654	-0.227461
6	6	0	1.491883	-1.391276	0.176099
7	1	0	-0.470759	-2.247901	0.153827
8	6	0	1.582076	0.998057	-0.125332
9	6	0	2.237320	-0.210475	0.069956
10	1	0	1.986495	-2.347424	0.331395
11	1	0	2.153402	1.922127	-0.204809
12	6	0	-2.678407	0.510484	1.102160
13	1	0	-3.760794	0.682143	1.016151
14	1	0	-2.509106	-0.200139	1.920808
15	1	0	-2.196291	1.458040	1.352831
16	6	0	-2.790980	-1.317269	-0.631066
17	1	0	-3.865312	-1.143590	-0.770900
18	1	0	-2.383401	-1.706671	-1.570023
19	1	0	-2.687137	-2.103273	0.127165
20	6	0	3.746001	-0.258514	0.124620
21	1	0	4.179481	-0.418377	-0.871747
22	1	0	4.160468	0.677979	0.512290
23	1	0	4.097583	-1.075040	0.765739
24	8	0	-0.428825	2.236046	-0.396598

Rotational constants (GHZ): 2.0970161 0.7426100 0.5955077

- B3PW91 6-311G+dp/CPCM

H_2O
Electronic Energy -76.466463
Stoichiometry H2O
Framework group CS[SG(H2O)]
Deg. of freedom 3
Full point group CS NOp 2
Largest Abelian subgroup CS NOp 2
Largest concise Abelian subgroup C1 NOp 1
Standard orientation:

Center Number	Atomic Number	Atomic Type	Coordinates (Angstroms) X	Y	Z
1	8	0	0.000000	0.115552	0.000000
2	1	0	0.768344	-0.462208	0.000000
3	1	0	-0.768344	-0.462205	0.000000

Rotational constants (GHZ): 845.7772941 424.7084360 282.7334013

OH^-
Electronic Energy -75.924270
Stoichiometry HO(1-)
Framework group C*V[C*(HO)]
Deg. of freedom 1
Full point group C*V NOp 4
Largest Abelian subgroup C2V NOp 4
Largest concise Abelian subgroup C1 NOp 1

Standard orientation:

Center Number	Atomic Number	Atomic Type	Coordinates (Angstroms) X	Y	Z
1	8	0	0.000000	0.000000	0.106682
2	1	0	0.000000	0.000000	-0.853456

Rotational constants (GHZ): 0.0000000 578.2316725 578.2316725

Phenol
Electronic Energy -307.441733
Stoichiometry C6H6O
Framework group C1[X(C6H6O)]
Deg. of freedom 33
Full point group C1 NOp 1
Largest Abelian subgroup C1 NOp 1
Largest concise Abelian subgroup C1 NOp 1
Standard orientation:

Center Number	Atomic Number	Atomic Type	Coordinates (Angstroms) X	Y	Z
1	6	0	1.168101	-1.186116	0.000138
2	6	0	-0.220169	-1.219976	0.000067
3	6	0	-0.939066	-0.025077	-0.000047
4	6	0	-0.263529	1.195022	-0.000091
5	6	0	1.127883	1.215468	-0.000021
6	6	0	1.851976	0.028107	0.000095
7	1	0	1.721218	-2.119945	0.000227
8	1	0	-0.762720	-2.158768	0.000098
9	1	0	-0.824807	2.126490	-0.000180
10	1	0	1.645039	2.169605	-0.000056
11	1	0	2.936100	0.047458	0.000150
12	8	0	-2.298757	-0.110357	-0.000108
13	1	0	-2.675950	0.773447	-0.000219

Rotational constants (GHZ): 5.6846147 2.6293820 1.7978145

Phenolate
Electronic Energy -306.960080
Stoichiometry C6H5O(1-)
Framework group C1[X(C6H5O)]
Deg. of freedom 30
Full point group C1 NOp 1
Largest Abelian subgroup C1 NOp 1
Largest concise Abelian subgroup C1 NOp 1
Standard orientation:

Center Number	Atomic Number	Atomic Type	Coordinates (Angstroms) X	Y	Z
1	6	0	-1.098429	-1.197480	0.000008
2	6	0	0.287144	-1.208693	-0.000006
3	6	0	1.077535	0.000050	-0.000058
4	6	0	0.287157	1.208713	-0.000005
5	6	0	-1.098480	1.197438	0.000006
6	6	0	-1.825642	-0.000005	0.000004
7	1	0	-1.635179	-2.147169	0.000029
8	1	0	0.831125	-2.151400	0.000005
9	1	0	0.830978	2.151511	0.000009
10	1	0	-1.635190	2.147147	0.000025
11	1	0	-2.912018	-0.000060	-0.000008
12	8	0	2.343072	-0.000021	0.000031

Rotational constants (GHZ): 5.7364512 2.6911837 1.8318121

Thymol
Electronic Energy -464.685388
Stoichiometry C10H14O
Framework group C1[X(C10H14O)]

Deg. of freedom 69
Full point group C1 NOp 1
Largest Abelian subgroup C1 NOp 1
Largest concise Abelian subgroup C1 NOp 1
Standard orientation:

Center Number	Atomic Number	Atomic Type	Coordinates (Angstroms) X	Y	Z
1	6	0	-2.102627	-0.021835	-0.188280
2	1	0	-2.325004	0.788879	-0.891143
3	6	0	-0.591866	-0.133928	-0.118531
4	6	0	0.081206	-1.340220	0.054001
5	6	0	0.200576	1.022486	-0.199677
6	6	0	1.470189	-1.412258	0.143141
7	1	0	-0.492049	-2.259159	0.115705
8	6	0	1.587258	0.959079	-0.115885
9	6	0	2.246232	-0.259857	0.058395
10	1	0	1.950590	-2.376760	0.275871
11	1	0	2.166149	1.878778	-0.188297
12	6	0	-2.676731	0.389226	1.176345
13	1	0	-3.760682	0.531026	1.113273
14	1	0	-2.478978	-0.383735	1.926394
15	1	0	-2.232574	1.324108	1.525527
16	6	0	-2.787632	-1.286353	-0.706741
17	1	0	-3.856712	-1.100949	-0.846755
18	1	0	-2.372168	-1.606254	-1.666669
19	1	0	-2.696380	-2.119637	-0.002397
20	6	0	3.747152	-0.310223	0.146704
21	1	0	4.213197	0.085536	-0.761648
22	1	0	4.116762	0.285339	0.987886
23	1	0	4.099810	-1.334714	0.283149
24	8	0	-0.447195	2.216267	-0.366631
25	1	0	0.203048	2.920706	-0.434673

Rotational constants (GHZ): 2.0412376 0.7375072 0.5927874

Thymolate

Electronic Energy -464.200663
Stoichiometry C10H13O(1-)
Framework group C1[X(C10H13O)]
Deg. of freedom 66
Full point group C1 NOp 1
Largest Abelian subgroup C1 NOp 1
Largest concise Abelian subgroup C1 NOp 1
Standard orientation:

Center Number	Atomic Number	Atomic Type	Coordinates (Angstroms) X	Y	Z
1	6	0	-2.088528	-0.026439	-0.210929
2	1	0	-2.286134	0.742931	-0.969841
3	6	0	-0.581453	-0.127125	-0.122350
4	6	0	0.096100	-1.320625	0.074286
5	6	0	0.154670	1.121654	-0.227461
6	6	0	1.491883	-1.391276	0.176099
7	1	0	-0.470759	-2.247901	0.153827
8	6	0	1.582076	0.998057	-0.125332
9	6	0	2.237320	-0.210475	0.069956
10	1	0	1.986495	-2.347424	0.331395
11	1	0	2.153402	1.922127	-0.204809
12	6	0	-2.678407	0.510484	1.102160
13	1	0	-3.760794	0.682143	1.016151
14	1	0	-2.509106	-0.200139	1.920808
15	1	0	-2.196291	1.458040	1.352831
16	6	0	-2.790980	-1.317269	-0.631066
17	1	0	-3.865312	-1.143590	-0.770900
18	1	0	-2.383401	-1.706671	-1.570023
19	1	0	-2.687137	-2.103273	0.127165
20	6	0	3.746001	-0.258514	0.124620
21	1	0	4.179481	-0.418377	-0.871747

22	1	0	4.160468	0.677979	0.512290
23	1	0	4.097583	-1.075040	0.765739
24	8	0	-0.428825	2.236046	-0.396598

Rotational constants (GHZ): 2.0970161 0.7426100 0.5955077

- wB97XD 6-311G+dp/SMD

H_2O

Electronic Energy -76.446101
Stoichiometry H2O
Framework group CS[SG(H2O)]
Deg. of freedom 3
Full point group CS NOp 2
Largest Abelian subgroup CS NOp 2
Largest concise Abelian subgroup C1 NOp 1
Standard orientation:

Center Number	Atomic Number	Atomic Type	Coordinates (Angstroms) X	Y	Z
1	8	0	0.000000	0.115552	0.000000
2	1	0	0.768344	-0.462208	0.000000
3	1	0	-0.768344	-0.462205	0.000000

Rotational constants (GHZ): 845.7772941 424.7084360 282.7334013

OH^-

Electronic Energy -75.949093
Stoichiometry HO(1-)
Framework group C*V[C*(HO)]
Deg. of freedom 1
Full point group C*V NOp 4
Largest Abelian subgroup C2V NOp 4
Largest concise Abelian subgroup C1 NOp 1
Standard orientation:

Center Number	Atomic Number	Atomic Type	Coordinates (Angstroms) X	Y	Z
1	8	0	0.000000	0.000000	0.106682
2	1	0	0.000000	0.000000	-0.853456

Rotational constants (GHZ): 0.0000000 578.2316725 578.2316725

Phenol

Electronic Energy -307.456324
Stoichiometry C6H6O
Framework group C1[X(C6H6O)]
Deg. of freedom 33
Full point group C1 NOp 1
Largest Abelian subgroup C1 NOp 1
Largest concise Abelian subgroup C1 NOp 1
Standard orientation:

Center Number	Atomic Number	Atomic Type	Coordinates (Angstroms) X	Y	Z
1	6	0	1.166365	-1.185110	0.000138
2	6	0	-0.220317	-1.218357	0.000067
3	6	0	-0.937315	-0.024576	-0.000047
4	6	0	-0.263434	1.194125	-0.000090
5	6	0	1.126691	1.213840	-0.000021
6	6	0	1.849484	0.027865	0.000094
7	1	0	1.719159	-2.117999	0.000227
8	1	0	-0.762921	-2.156095	0.000099
9	1	0	-0.823336	2.125277	-0.000179
10	1	0	1.643936	2.166813	-0.000056
11	1	0	2.932644	0.047041	0.000150
12	8	0	-2.295224	-0.110072	-0.000109

13	1	0	-2.676535	0.768815	-0.000219

Rotational constants (GHZ): 5.6970930 2.6365260 1.8024023

Phenolate
Electronic Energy -306.977872
Stoichiometry C6H5O(1-)
Framework group C1[X(C6H5O)]
Deg. of freedom 30
Full point group C1 NOp 1
Largest Abelian subgroup C1 NOp 1
Largest concise Abelian subgroup C1 NOp 1
Standard orientation:

Center Number	Atomic Number	Atomic Type	Coordinates (Angstroms) X	Y	Z
1	6	0	-1.096527	-1.196063	0.000004
2	6	0	0.287292	-1.208230	0.000009
3	6	0	1.075469	0.000060	-0.000011
4	6	0	0.287314	1.208277	0.000006
5	6	0	-1.096562	1.196021	0.000009
6	6	0	-1.822882	-0.000008	-0.000012
7	1	0	-1.632640	-2.144860	0.000012
8	1	0	0.830704	-2.150205	0.000015
9	1	0	0.830590	2.150337	0.000004
10	1	0	-1.632630	2.144832	0.000013
11	1	0	-2.908351	-0.000073	-0.000040
12	8	0	2.338462	-0.000047	-0.000005

Rotational constants (GHZ): 5.7454342 2.7006297 1.8371031

Thymol
Electronic Energy -464.711087
Stoichiometry C10H14O
Framework group C1[X(C10H14O)]
Deg. of freedom 69
Full point group C1 NOp 1
Largest Abelian subgroup C1 NOp 1
Largest concise Abelian subgroup C1 NOp 1
Standard orientation:

Center Number	Atomic Number	Atomic Type	Coordinates (Angstroms) X	Y	Z
1	6	0	-2.104768	-0.011752	-0.189272
2	1	0	-2.341862	0.800275	-0.883431
3	6	0	-0.592586	-0.125482	-0.129420
4	6	0	0.069405	-1.337938	0.041312
5	6	0	0.206772	1.025236	-0.201078
6	6	0	1.457510	-1.418360	0.134779
7	1	0	-0.509127	-2.253136	0.101438
8	6	0	1.591632	0.955713	-0.112739
9	6	0	2.238478	-0.268973	0.058306
10	1	0	1.932446	-2.385195	0.265989
11	1	0	2.172072	1.872640	-0.177571
12	6	0	-2.662454	0.374973	1.187586
13	1	0	-3.745805	0.520762	1.137373
14	1	0	-2.459451	-0.418201	1.914935
15	1	0	-2.211909	1.299350	1.558240
16	6	0	-2.792749	-1.274335	-0.706938
17	1	0	-3.861832	-1.084203	-0.836365
18	1	0	-2.385078	-1.589338	-1.671685
19	1	0	-2.691550	-2.107796	-0.005189
20	6	0	3.739662	-0.325415	0.152259
21	1	0	4.204089	0.076145	-0.753075
22	1	0	4.101925	0.270620	0.994897
23	1	0	4.087013	-1.351663	0.286664
24	8	0	-0.427916	2.231647	-0.364852
25	1	0	0.226978	2.934565	-0.422173

Rotational constants (GHZ): 2.0315235 0.7398440 0.5940136

Thymolate
Electronic Energy -464.230438
Stoichiometry C10H13O(1-)
Framework group C1[X(C10H13O)]
Deg. of freedom 66
Full point group C1 NOp 1
Largest Abelian subgroup C1 NOp 1
Largest concise Abelian subgroup C1 NOp 1
Standard orientation:

Center Number	Atomic Number	Atomic Type	Coordinates (Angstroms) X	Y	Z
1	6	0	-2.088450	-0.028244	-0.218154
2	1	0	-2.295055	0.718734	-0.994536
3	6	0	-0.580140	-0.127172	-0.131349
4	6	0	0.094628	-1.319375	0.063536
5	6	0	0.155691	1.120017	-0.230419
6	6	0	1.488834	-1.390864	0.169535
7	1	0	-0.472125	-2.245955	0.138074
8	6	0	1.580544	0.997369	-0.122445
9	6	0	2.231853	-0.211371	0.070850
10	1	0	1.982824	-2.346686	0.323521
11	1	0	2.152717	1.920573	-0.195534
12	6	0	-2.670018	0.532747	1.087818
13	1	0	-3.753535	0.693614	1.006431
14	1	0	-2.487062	-0.163436	1.915526
15	1	0	-2.190996	1.487118	1.315292
16	6	0	-2.790259	-1.331958	-0.599539
17	1	0	-3.862727	-1.159525	-0.748263
18	1	0	-2.378180	-1.751713	-1.522603
19	1	0	-2.688990	-2.089613	0.186790
20	6	0	3.741995	-0.259180	0.132124
21	1	0	4.174704	-0.388658	-0.867370
22	1	0	4.151345	0.666156	0.549020
23	1	0	4.088899	-1.093853	0.750538
24	8	0	-0.426237	2.232679	-0.398578

Rotational constants (GHZ): 2.1009789 0.7457645 0.5964070

- wB97XD 6-311G+dp/PCM

H_2O
Electronic Energy -76.440343
Stoichiometry H2O
Framework group CS[SG(H2O)]
Deg. of freedom 3
Full point group CS NOp 2
Largest Abelian subgroup CS NOp 2
Largest concise Abelian subgroup C1 NOp 1
Standard orientation:

Center Number	Atomic Number	Atomic Type	Coordinates (Angstroms) X	Y	Z
1	8	0	0.000000	0.115552	0.000000
2	1	0	0.768344	-0.462208	0.000000
3	1	0	-0.768344	-0.462205	0.000000

Rotational constants (GHZ): 845.7772941 424.7084360 282.7334013

OH^-
Electronic Energy -75.928811
Stoichiometry HO(1-)
Framework group C*V[C*(HO)]
Deg. of freedom 1
Full point group C*V NOp 4

Largest Abelian subgroup C2V NOp 4
Largest concise Abelian subgroup C1 NOp 1
Standard orientation:

Center Number	Atomic Number	Atomic Type	Coordinates (Angstroms) X	Y	Z
1	8	0	0.000000	0.000000	0.106682
2	1	0	0.000000	0.000000	-0.853456

Rotational constants (GHZ): 0.0000000 578.2316725 578.2316725

Phenol
Electronic Energy -307.453422
Stoichiometry C6H6O
Framework group C1[X(C6H6O)]
Deg. of freedom 33
Full point group C1 NOp 1
Largest Abelian subgroup C1 NOp 1
Largest concise Abelian subgroup C1 NOp 1
Standard orientation:

Center Number	Atomic Number	Atomic Type	Coordinates (Angstroms) X	Y	Z
1	6	0	1.166365	-1.185110	0.000138
2	6	0	-0.220317	-1.218357	0.000067
3	6	0	-0.937315	-0.024576	-0.000047
4	6	0	-0.263434	1.194125	-0.000090
5	6	0	1.126691	1.213840	-0.000021
6	6	0	1.849484	0.027865	0.000094
7	1	0	1.719159	-2.117999	0.000227
8	1	0	-0.762921	-2.156095	0.000099
9	1	0	-0.823336	2.125277	-0.000179
10	1	0	1.643936	2.166813	-0.000056
11	1	0	2.932644	0.047041	0.000150
12	8	0	-2.295224	-0.110072	-0.000109
13	1	0	-2.676535	0.768815	-0.000219

Rotational constants (GHZ): 5.6970930 2.6365260 1.8024023

Phenolate
Electronic Energy -306.969889
Stoichiometry C6H5O(1-)
Framework group C1[X(C6H5O)]
Deg. of freedom 30
Full point group C1 NOp 1
Largest Abelian subgroup C1 NOp 1
Largest concise Abelian subgroup C1 NOp 1
Standard orientation:

Center Number	Atomic Number	Atomic Type	Coordinates (Angstroms) X	Y	Z
1	6	0	-1.096527	-1.196063	0.000004
2	6	0	0.287292	-1.208230	0.000009
3	6	0	1.075469	0.000060	-0.000011
4	6	0	0.287314	1.208277	0.000006
5	6	0	-1.096562	1.196021	0.000009
6	6	0	-1.822882	-0.000008	-0.000012
7	1	0	-1.632640	-2.144860	0.000012
8	1	0	0.830704	-2.150205	0.000015
9	1	0	0.830590	2.150337	0.000004
10	1	0	-1.632630	2.144832	0.000013
11	1	0	-2.908351	-0.000073	-0.000040
12	8	0	2.338462	-0.000047	-0.000005

Rotational constants (GHZ): 5.7454342 2.7006297 1.8371031

Thymol
Electronic Energy -464.710368
Stoichiometry C10H14O

Framework group C1[X(C10H14O)]
Deg. of freedom 69
Full point group C1 NOp 1
Largest Abelian subgroup C1 NOp 1
Largest concise Abelian subgroup C1 NOp 1
Standard orientation:

Center Number	Atomic Number	Atomic Type	Coordinates (Angstroms) X	Y	Z
1	6	0	-2.102725	-0.016373	-0.189920
2	1	0	-2.333888	0.792054	-0.890455
3	6	0	-0.590812	-0.130265	-0.127556
4	6	0	0.074656	-1.339406	0.044846
5	6	0	0.203223	1.024471	-0.202947
6	6	0	1.463020	-1.415894	0.138008
7	1	0	-0.500858	-2.256293	0.105805
8	6	0	1.588691	0.957142	-0.115561
9	6	0	2.239898	-0.264936	0.058137
10	1	0	1.939713	-2.381464	0.271039
11	1	0	2.171566	1.872489	-0.183455
12	6	0	-2.662395	0.387782	1.182133
13	1	0	-3.745305	0.534637	1.129268
14	1	0	-2.460764	-0.395984	1.919698
15	1	0	-2.208072	1.315935	1.536370
16	6	0	-2.792626	-1.282160	-0.699928
17	1	0	-3.860167	-1.091137	-0.837743
18	1	0	-2.378739	-1.608134	-1.657957
19	1	0	-2.699839	-2.109051	0.010848
20	6	0	3.742761	-0.315961	0.151255
21	1	0	4.205612	0.083417	-0.755551
22	1	0	4.103987	0.282925	0.991997
23	1	0	4.094486	-1.340031	0.288575
24	8	0	-0.439174	2.218060	-0.367857
25	1	0	0.203521	2.929763	-0.416391

Rotational constants (GHZ): 2.0395055 0.7396510 0.5941310

Thymolate
Electronic Energy -464.223394
Stoichiometry C10H13O(1-)
Framework group C1[X(C10H13O)]
Deg. of freedom 66
Full point group C1 NOp 1
Largest Abelian subgroup C1 NOp 1
Largest concise Abelian subgroup C1 NOp 1
Standard orientation:

Center Number	Atomic Number	Atomic Type	Coordinates (Angstroms) X	Y	Z
1	6	0	-2.088450	-0.028244	-0.218154
2	1	0	-2.295055	0.718734	-0.994536
3	6	0	-0.580140	-0.127172	-0.131349
4	6	0	0.094628	-1.319375	0.063536
5	6	0	0.155691	1.120017	-0.230419
6	6	0	1.488834	-1.390864	0.169535
7	1	0	-0.472125	-2.245955	0.138074
8	6	0	1.580544	0.997369	-0.122445
9	6	0	2.231853	-0.211371	0.070850
10	1	0	1.982824	-2.346686	0.323521
11	1	0	2.152717	1.920573	-0.195534
12	6	0	-2.670018	0.532747	1.087818
13	1	0	-3.753535	0.693614	1.006431
14	1	0	-2.487062	-0.163436	1.915526
15	1	0	-2.190996	1.487118	1.315292
16	6	0	-2.790259	-1.331958	-0.599539
17	1	0	-3.862727	-1.159525	-0.748263
18	1	0	-2.378180	-1.751713	-1.522603
19	1	0	-2.688990	-2.089613	0.186790
20	6	0	3.741995	-0.259180	0.132124

21	1	0	4.174704	-0.388658	-0.867370
22	1	0	4.151345	0.666156	0.549020
23	1	0	4.088899	-1.093853	0.750538
24	8	0	-0.426237	2.232679	-0.398578

Rotational constants (GHZ): 2.1009789 0.7457645 0.5964070

- wB97XD 6-311G+dp/CPCM

H_2O
Electronic Energy -76.440394
Stoichiometry H2O
Framework group CS[SG(H2O)]
Deg. of freedom 3
Full point group CS NOp 2
Largest Abelian subgroup CS NOp 2
Largest concise Abelian subgroup C1 NOp 1
Standard orientation:

Center Number	Atomic Number	Atomic Type	Coordinates (Angstroms) X	Y	Z
1	8	0	0.000000	0.115552	0.000000
2	1	0	0.768344	-0.462208	0.000000
3	1	0	-0.768344	-0.462205	0.000000

Rotational constants (GHZ): 845.7772941 424.7084360 282.7334013

OH^-
Electronic Energy -75.928866
Stoichiometry HO(1-)
Framework group C*V[C*(HO)]
Deg. of freedom 1
Full point group C*V NOp 4
Largest Abelian subgroup C2V NOp 4
Largest concise Abelian subgroup C1 NOp 1
Standard orientation:

Center Number	Atomic Number	Atomic Type	Coordinates (Angstroms) X	Y	Z
1	8	0	0.000000	0.000000	0.106682
2	1	0	0.000000	0.000000	-0.853456

Rotational constants (GHZ): 0.0000000 578.2316725 578.2316725

Phenol
Electronic Energy -307.453490
Stoichiometry C6H6O
Framework group C1[X(C6H6O)]
Deg. of freedom 33
Full point group C1 NOp 1
Largest Abelian subgroup C1 NOp 1
Largest concise Abelian subgroup C1 NOp 1
Standard orientation:

Center Number	Atomic Number	Atomic Type	Coordinates (Angstroms) X	Y	Z
1	6	0	1.171139	-1.184735	0.000139
2	6	0	-0.217023	-1.220032	0.000067
3	6	0	-0.937342	-0.026375	-0.000049
4	6	0	-0.267572	1.195306	-0.000092
5	6	0	1.123568	1.216774	-0.000020
6	6	0	1.850798	0.031227	0.000096
7	1	0	1.726025	-2.116478	0.000230
8	1	0	-0.754361	-2.161488	0.000099
9	1	0	-0.830092	2.123794	-0.000181
10	1	0	1.637803	2.171349	-0.000055
11	1	0	2.934097	0.052986	0.000153

12	8	0	-2.296200	-0.113491	-0.000111
13	1	0	-2.685280	0.764770	-0.000210

Rotational constants (GHZ): 5.6844468 2.6331193 1.7995440

Phenolate
Electronic Energy -306.970004
Stoichiometry C6H5O(1-)
Framework group C1[X(C6H5O)]
Deg. of freedom 30
Full point group C1 NOp 1
Largest Abelian subgroup C1 NOp 1
Largest concise Abelian subgroup C1 NOp 1
Standard orientation:

Center Number	Atomic Number	Atomic Type	Coordinates (Angstroms) X	Y	Z
1	6	0	-1.096527	-1.196063	0.000004
2	6	0	0.287292	-1.208230	0.000009
3	6	0	1.075469	0.000060	-0.000011
4	6	0	0.287314	1.208277	0.000006
5	6	0	-1.096562	1.196021	0.000009
6	6	0	-1.822882	-0.000008	-0.000012
7	1	0	-1.632640	-2.144860	0.000012
8	1	0	0.830704	-2.150205	0.000015
9	1	0	0.830590	2.150337	0.000004
10	1	0	-1.632630	2.144832	0.000013
11	1	0	-2.908351	-0.000073	-0.000040
12	8	0	2.338462	-0.000047	-0.000005

Rotational constants (GHZ): 5.7454342 2.7006297 1.8371031

Thymol
Electronic Energy -464.710436
Stoichiometry C10H14O
Framework group C1[X(C10H14O)]
Deg. of freedom 69
Full point group C1 NOp 1
Largest Abelian subgroup C1 NOp 1
Largest concise Abelian subgroup C1 NOp 1
Standard orientation:

Center Number	Atomic Number	Atomic Type	Coordinates (Angstroms) X	Y	Z
1	6	0	-2.102786	-0.015829	-0.189468
2	1	0	-2.334056	0.793880	-0.888471
3	6	0	-0.590867	-0.129875	-0.127452
4	6	0	0.074357	-1.339194	0.044821
5	6	0	0.203394	1.024724	-0.202801
6	6	0	1.462716	-1.415987	0.137872
7	1	0	-0.501365	-2.255952	0.105814
8	6	0	1.588882	0.957095	-0.115513
9	6	0	2.239830	-0.265148	0.058057
10	1	0	1.939207	-2.381671	0.270802
11	1	0	2.171945	1.872317	-0.183350
12	6	0	-2.662304	0.385699	1.183427
13	1	0	-3.745206	0.532723	1.130955
14	1	0	-2.460678	-0.399546	1.919423
15	1	0	-2.207915	1.313116	1.539548
16	6	0	-2.792724	-1.280685	-0.701773
17	1	0	-3.860275	-1.089399	-0.839138
18	1	0	-2.378908	-1.604843	-1.660450
19	1	0	-2.699849	-2.108911	0.007422
20	6	0	3.742694	-0.316484	0.151084
21	1	0	4.205555	0.082814	-0.755751
22	1	0	4.104083	0.282413	0.991744
23	1	0	4.094215	-1.340618	0.288426
24	8	0	-0.438745	2.218461	-0.367576
25	1	0	0.204054	2.930098	-0.415893

Rotational constants (GHZ): 2.0392503 0.7395820 0.5941750

Thymolate
Electronic Energy -464.223575
Stoichiometry C10H13O(1-)
Framework group C1[X(C10H13O)]
Deg. of freedom 66
Full point group C1 NOp 1
Largest Abelian subgroup C1 NOp 1
Largest concise Abelian subgroup C1 NOp 1
Standard orientation:

Center Number	Atomic Number	Atomic Type	Coordinates (Angstroms) X	Y	Z
1	6	0	-2.088450	-0.028244	-0.218154
2	1	0	-2.295055	0.718734	-0.994536
3	6	0	-0.580140	-0.127172	-0.131349
4	6	0	0.094628	-1.319375	0.063536
5	6	0	0.155691	1.120017	-0.230419
6	6	0	1.488834	-1.390864	0.169535
7	1	0	-0.472125	-2.245955	0.138074
8	6	0	1.580544	0.997369	-0.122445
9	6	0	2.231853	-0.211371	0.070850
10	1	0	1.982824	-2.346686	0.323521
11	1	0	2.152717	1.920573	-0.195534
12	6	0	-2.670018	0.532747	1.087818
13	1	0	-3.753535	0.693614	1.006431
14	1	0	-2.487062	-0.163436	1.915526
15	1	0	-2.190996	1.487118	1.315292
16	6	0	-2.790259	-1.331958	-0.599539
17	1	0	-3.862727	-1.159525	-0.748263
18	1	0	-2.378180	-1.751713	-1.522603
19	1	0	-2.688990	-2.089613	0.186790
20	6	0	3.741995	-0.259180	0.132124
21	1	0	4.174704	-0.388658	-0.867370
22	1	0	4.151345	0.666156	0.549020
23	1	0	4.088899	-1.093853	0.750538
24	8	0	-0.426237	2.232679	-0.398578

Rotational constants (GHZ): 2.1009789 0.7457645 0.5964070

- CAM-B3LYP 6-311G+dp/SMD

H_2O
Electronic Energy -76.444219
Stoichiometry H2O
Framework group CS[SG(H2O)]
Deg. of freedom 3
Full point group CS NOp 2
Largest Abelian subgroup CS NOp 2
Largest concise Abelian subgroup C1 NOp 1
Standard orientation:

Center Number	Atomic Number	Atomic Type	Coordinates (Angstroms) X	Y	Z
1	8	0	0.000000	0.115552	0.000000
2	1	0	0.768344	-0.462208	0.000000
3	1	0	-0.768344	-0.462205	0.000000

Rotational constants (GHZ): 845.7772941 424.7084360 282.7334013

OH^-
Electronic Energy -75.952738
Stoichiometry HO(1-)
Framework group C*V[C*(HO)]
Deg. of freedom 1

Full point group C*V NOp 4
Largest Abelian subgroup C2V NOp 4
Largest concise Abelian subgroup C1 NOp 1

Standard orientation:

Center Number	Atomic Number	Atomic Type	Coordinates (Angstroms) X	Y	Z
1	8	0	0.000000	0.000000	0.106682
2	1	0	0.000000	0.000000	-0.853456

Rotational constants (GHZ): 0.0000000 578.2316725 578.2316725

Phenol

Electronic Energy -307.405415
Stoichiometry C6H6O
Framework group C1[X(C6H6O)]
Deg. of freedom 33
Full point group C1 NOp 1
Largest Abelian subgroup C1 NOp 1
Largest concise Abelian subgroup C1 NOp 1

Standard orientation:

Center Number	Atomic Number	Atomic Type	Coordinates (Angstroms) X	Y	Z
1	6	0	1.174028	-1.181615	0.000141
2	6	0	-0.212885	-1.220510	0.000066
3	6	0	-0.929440	-0.029669	-0.000050
4	6	0	-0.268651	1.192815	-0.000093
5	6	0	1.120360	1.217716	-0.000018
6	6	0	1.848803	0.034670	0.000098
7	1	0	1.731002	-2.111206	0.000231
8	1	0	-0.747446	-2.162822	0.000099
9	1	0	-0.843187	2.112738	-0.000184
10	1	0	1.633350	2.172262	-0.000053
11	1	0	2.931507	0.059120	0.000156
12	8	0	-2.301431	-0.115083	-0.000114
13	1	0	-2.687068	0.770121	-0.000199

Rotational constants (GHZ): 5.6947073 2.6316085 1.7998645

Phenolate

Electronic Energy -306.931240
Stoichiometry C6H5O(1-)
Framework group C1[X(C6H5O)]
Deg. of freedom 30
Full point group C1 NOp 1
Largest Abelian subgroup C1 NOp 1
Largest concise Abelian subgroup C1 NOp 1

Standard orientation:

Center Number	Atomic Number	Atomic Type	Coordinates (Angstroms) X	Y	Z
1	6	0	1.094882	-1.194362	-0.000004
2	6	0	-0.286655	-1.206551	-0.000027
3	6	0	-1.072024	0.000030	0.000007
4	6	0	-0.286757	1.206578	-0.000011
5	6	0	1.094832	1.194355	-0.000027
6	6	0	1.820539	0.000032	0.000027
7	1	0	1.630268	-2.142343	0.000006
8	1	0	-0.831695	-2.146101	-0.000033
9	1	0	-0.831723	2.146187	0.000004
10	1	0	1.630109	2.142382	-0.000011
11	1	0	2.904951	-0.000001	0.000085
12	8	0	-2.336352	-0.000077	0.000020

Rotational constants (GHZ): 5.7619198 2.7075373 1.8419850

Thymol
Electronic Energy -464.604855
Stoichiometry C10H14O
Framework group C1[X(C10H14O)]
Deg. of freedom 69
Full point group C1 NOp 1
Largest Abelian subgroup C1 NOp 1
Largest concise Abelian subgroup C1 NOp 1
Standard orientation:

Center Number	Atomic Number	Atomic Type	Coordinates (Angstroms) X	Y	Z
1	6	0	-2.097266	-0.033915	-0.199878
2	1	0	-2.317402	0.740233	-0.940400
3	6	0	-0.586793	-0.144617	-0.122883
4	6	0	0.088737	-1.344260	0.050100
5	6	0	0.197855	1.011909	-0.202754
6	6	0	1.475713	-1.407747	0.142432
7	1	0	-0.477680	-2.265177	0.110220
8	6	0	1.579725	0.958579	-0.115796
9	6	0	2.242134	-0.254556	0.059736
10	1	0	1.960644	-2.367847	0.276269
11	1	0	2.152878	1.879909	-0.186558
12	6	0	-2.672631	0.441741	1.140880
13	1	0	-3.754599	0.580559	1.068518
14	1	0	-2.477453	-0.295211	1.924893
15	1	0	-2.228464	1.390310	1.444522
16	6	0	-2.783249	-1.320994	-0.653599
17	1	0	-3.850579	-1.141236	-0.800172
18	1	0	-2.370600	-1.688812	-1.595534
19	1	0	-2.690194	-2.115571	0.091447
20	6	0	3.743862	-0.295176	0.152079
21	1	0	4.207153	0.103104	-0.754176
22	1	0	4.104879	0.301743	0.993438
23	1	0	4.101507	-1.316162	0.289057
24	8	0	-0.458457	2.201434	-0.372728
25	1	0	0.179036	2.916899	-0.441607

Rotational constants (GHZ): 2.0552348 0.7417849 0.5935965

Thymolate
Electronic Energy -464.128329
Stoichiometry C10H13O(1-)
Framework group C1[X(C10H13O)]
Deg. of freedom 66
Full point group C1 NOp 1
Largest Abelian subgroup C1 NOp 1
Largest concise Abelian subgroup C1 NOp 1
Standard orientation:

Center Number	Atomic Number	Atomic Type	Coordinates (Angstroms) X	Y	Z
1	6	0	-2.085488	-0.034325	-0.220176
2	1	0	-2.286260	0.700279	-1.008192
3	6	0	-0.578375	-0.132365	-0.126126
4	6	0	0.099893	-1.320371	0.067256
5	6	0	0.155059	1.113030	-0.228058
6	6	0	1.493277	-1.387660	0.171473
7	1	0	-0.462922	-2.247590	0.142234
8	6	0	1.577922	0.996592	-0.122168
9	6	0	2.232555	-0.208042	0.070542
10	1	0	1.989369	-2.341008	0.325584
11	1	0	2.144182	1.921641	-0.197626
12	6	0	-2.676829	0.549692	1.069876
13	1	0	-3.757133	0.716109	0.973575
14	1	0	-2.510130	-0.132092	1.911183
15	1	0	-2.195806	1.503849	1.286599
16	6	0	-2.786765	-1.341406	-0.585991
17	1	0	-3.858960	-1.171641	-0.731008
18	1	0	-2.381292	-1.769178	-1.506874

19	1	0	-2.681341	-2.092084	0.204262
20	6	0	3.741612	-0.251093	0.130105
21	1	0	4.176051	-0.368740	-0.868991
22	1	0	4.147835	0.670297	0.555490
23	1	0	4.091598	-1.089622	0.739197
24	8	0	-0.431545	2.224433	-0.398229

Rotational constants (GHZ): 2.1110515 0.7464748 0.5964394

- CAM-B3LYP 6-311G+dp/PCM

H_2O

Electronic Energy -76.438457
Stoichiometry H2O
Framework group CS[SG(H2O)]
Deg. of freedom 3
Full point group CS NOp 2
Largest Abelian subgroup CS NOp 2
Largest concise Abelian subgroup C1 NOp 1
Standard orientation:

Center Number	Atomic Number	Atomic Type	Coordinates (Angstroms) X	Y	Z
1	8	0	0.000000	0.115552	0.000000
2	1	0	0.768344	-0.462208	0.000000
3	1	0	-0.768344	-0.462205	0.000000

Rotational constants (GHZ): 845.7772941 424.7084360 282.7334013

OH^-

Electronic Energy -75.952738
Stoichiometry HO(1-)
Framework group C*V[C*(HO)]
Deg. of freedom 1
Full point group C*V NOp 4
Largest Abelian subgroup C2V NOp 4
Largest concise Abelian subgroup C1 NOp 1
Standard orientation:

Center Number	Atomic Number	Atomic Type	Coordinates (Angstroms) X	Y	Z
1	8	0	0.000000	0.000000	0.106682
2	1	0	0.000000	0.000000	-0.853456

Rotational constants (GHZ): 0.0000000 578.2316725 578.2316725

Phenol

Electronic Energy -307.402499
Stoichiometry C6H6O
Framework group C1[X(C6H6O)]
Deg. of freedom 33
Full point group C1 NOp 1
Largest Abelian subgroup C1 NOp 1
Largest concise Abelian subgroup C1 NOp 1
Standard orientation:

Center Number	Atomic Number	Atomic Type	Coordinates (Angstroms) X	Y	Z
1	6	0	1.170045	-1.182949	0.000139
2	6	0	-0.216361	-1.218393	0.000067
3	6	0	-0.933741	-0.026748	-0.000049
4	6	0	-0.266744	1.193145	-0.000092
5	6	0	1.122501	1.215255	-0.000020
6	6	0	1.848549	0.031457	0.000097
7	1	0	1.724690	-2.113821	0.000230
8	1	0	-0.753954	-2.158736	0.000099

9	1	0	-0.830906	2.119516	-0.000181
10	1	0	1.636618	2.169023	-0.000054
11	1	0	2.931067	0.053482	0.000154
12	8	0	-2.295778	-0.113390	-0.000113
13	1	0	-2.686782	0.767057	-0.000206

Rotational constants (GHZ): 5.7006956 2.6371994 1.8030775

Phenolate
Electronic Energy -306.923585
Stoichiometry C6H5O(1-)
Framework group C1[X(C6H5O)]
Deg. of freedom 30
Full point group C1 NOp 1
Largest Abelian subgroup C1 NOp 1
Largest concise Abelian subgroup C1 NOp 1
Standard orientation:

Center Number	Atomic Number	Atomic Type	Coordinates (Angstroms) X	Y	Z
1	6	0	1.094882	-1.194362	-0.000004
2	6	0	-0.286655	-1.206551	-0.000027
3	6	0	-1.072024	0.000030	0.000007
4	6	0	-0.286757	1.206578	-0.000011
5	6	0	1.094832	1.194355	-0.000027
6	6	0	1.820539	0.000032	0.000027
7	1	0	1.630268	-2.142343	0.000006
8	1	0	-0.831695	-2.146101	-0.000033
9	1	0	-0.831723	2.146187	0.000004
10	1	0	1.630109	2.142382	-0.000011
11	1	0	2.904951	-0.000001	0.000085
12	8	0	-2.336352	-0.000077	0.000020

Rotational constants (GHZ): 5.7619198 2.7075373 1.8419850

Thymol
Electronic Energy -464.604170
Stoichiometry C10H14O
Framework group C1[X(C10H14O)]
Deg. of freedom 69
Full point group C1 NOp 1
Largest Abelian subgroup C1 NOp 1
Largest concise Abelian subgroup C1 NOp 1
Standard orientation:

Center Number	Atomic Number	Atomic Type	Coordinates (Angstroms) X	Y	Z
1	6	0	-2.097266	-0.033915	-0.199878
2	1	0	-2.317402	0.740233	-0.940400
3	6	0	-0.586793	-0.144617	-0.122883
4	6	0	0.088737	-1.344260	0.050100
5	6	0	0.197855	1.011909	-0.202754
6	6	0	1.475713	-1.407747	0.142432
7	1	0	-0.477680	-2.265177	0.110220
8	6	0	1.579725	0.958579	-0.115796
9	6	0	2.242134	-0.254556	0.059736
10	1	0	1.960644	-2.367847	0.276269
11	1	0	2.152878	1.879909	-0.186558
12	6	0	-2.672631	0.441741	1.140880
13	1	0	-3.754599	0.580559	1.068518
14	1	0	-2.477453	-0.295211	1.924893
15	1	0	-2.228464	1.390310	1.444522
16	6	0	-2.783249	-1.320994	-0.653599
17	1	0	-3.850579	-1.141236	-0.800172
18	1	0	-2.370600	-1.688812	-1.595534
19	1	0	-2.690194	-2.115571	0.091447
20	6	0	3.743862	-0.295176	0.152079
21	1	0	4.207153	0.103104	-0.754176
22	1	0	4.104879	0.301743	0.993438

23	1	0	4.101507	-1.316162	0.289057
24	8	0	-0.458457	2.201434	-0.372728
25	1	0	0.179036	2.916899	-0.441607

Rotational constants (GHZ): 2.0552348 0.7417849 0.5935965

Thymolate
Electronic Energy -464.121695
Stoichiometry C10H13O(1-)
Framework group C1[X(C10H13O)]
Deg. of freedom 66
Full point group C1 NOp 1
Largest Abelian subgroup C1 NOp 1
Largest concise Abelian subgroup C1 NOp 1
Standard orientation:

Center Number	Atomic Number	Atomic Type	Coordinates (Angstroms) X	Y	Z
1	6	0	-2.085488	-0.034325	-0.220176
2	1	0	-2.286260	0.700279	-1.008192
3	6	0	-0.578375	-0.132365	-0.126126
4	6	0	0.099893	-1.320371	0.067256
5	6	0	0.155059	1.113030	-0.228058
6	6	0	1.493277	-1.387660	0.171473
7	1	0	-0.462922	-2.247590	0.142234
8	6	0	1.577922	0.996592	-0.122168
9	6	0	2.232555	-0.208042	0.070542
10	1	0	1.989369	-2.341008	0.325584
11	1	0	2.144182	1.921641	-0.197626
12	6	0	-2.676829	0.549692	1.069876
13	1	0	-3.757133	0.716109	0.973575
14	1	0	-2.510130	-0.132092	1.911183
15	1	0	-2.195806	1.503849	1.286599
16	6	0	-2.786765	-1.341406	-0.585991
17	1	0	-3.858960	-1.171641	-0.731008
18	1	0	-2.381292	-1.769178	-1.506874
19	1	0	-2.681341	-2.092084	0.204262
20	6	0	3.741612	-0.251093	0.130105
21	1	0	4.176051	-0.368740	-0.868991
22	1	0	4.147835	0.670297	0.555490
23	1	0	4.091598	-1.089622	0.739197
24	8	0	-0.431545	2.224433	-0.398229

Rotational constants (GHZ): 2.1110515 0.7464748 0.5964394

- CAM-B3LYP 6-311G+dp/CPCM

H_2O
Electronic Energy -76.438457
Stoichiometry H2O
Framework group CS[SG(H2O)]
Deg. of freedom 3
Full point group CS NOp 2
Largest Abelian subgroup CS NOp 2
Largest concise Abelian subgroup C1 NOp 1
Standard orientation:

Center Number	Atomic Number	Atomic Type	Coordinates (Angstroms) X	Y	Z
1	8	0	0.000000	0.117319	0.000000
2	1	0	0.762910	-0.469278	0.000000
3	1	0	-0.762910	-0.469278	0.000000

Rotational constants (GHZ): 820.4785628 430.7798306 282.4721242

OH^-
Electronic Energy-75.933126
Stoichiometry HO(1-)
Framework group C*V[C*(HO)]
Deg. of freedom 1
Full point group C*V NOp 4
Largest Abelian subgroup C2V NOp 4
Largest concise Abelian subgroup C1 NOp 1
Standard orientation:

Center Number	Atomic Number	Atomic Type	Coordinates (Angstroms) X	Y	Z
1	8	0	0.000000	0.000000	0.106682
2	1	0	0.000000	0.000000	-0.853456

Rotational constants (GHZ): 0.0000000 578.2316725 578.2316725

Phenol
Electronic Energy -307.402564
Stoichiometry C6H6O
Framework group C1[X(C6H6O)]
Deg. of freedom 33
Full point group C1 NOp 1
Largest Abelian subgroup C1 NOp 1
Largest concise Abelian subgroup C1 NOp 1
Standard orientation:

Center Number	Atomic Number	Atomic Type	Coordinates (Angstroms) X	Y	Z
1	6	0	1.170096	-1.182942	0.000139
2	6	0	-0.216325	-1.218421	0.000067
3	6	0	-0.933742	-0.026781	-0.000049
4	6	0	-0.266797	1.193150	-0.000092
5	6	0	1.122456	1.215297	-0.000020
6	6	0	1.848555	0.031503	0.000097
7	1	0	1.724770	-2.113795	0.000230
8	1	0	-0.753848	-2.158810	0.000099
9	1	0	-0.831047	2.119453	-0.000181
10	1	0	1.636533	2.169086	-0.000054
11	1	0	2.931073	0.053565	0.000154
12	8	0	-2.295773	-0.113423	-0.000113
13	1	0	-2.686758	0.767050	-0.000206

Rotational constants (GHZ): 5.7005432 2.6371952 1.8030603

Phenolate
Electronic Energy -306.923698
Stoichiometry C6H5O(1-)
Framework group C1[X(C6H5O)]
Deg. of freedom 30
Full point group C1 NOp 1
Largest Abelian subgroup C1 NOp 1
Largest concise Abelian subgroup C1 NOp 1
Standard orientation:

Center Number	Atomic Number	Atomic Type	Coordinates (Angstroms) X	Y	Z
1	6	0	1.095853	-1.194832	0.000011
2	6	0	-0.287747	-1.204774	-0.000006
3	6	0	-1.063112	0.000027	-0.000028
4	6	0	-0.287738	1.204785	-0.000005
5	6	0	1.095889	1.194802	0.000022
6	6	0	1.816925	-0.000006	0.000008
7	1	0	1.629846	-2.141343	0.000014
8	1	0	-0.828543	-2.147154	-0.000010
9	1	0	-0.828452	2.147212	-0.000011
10	1	0	1.629872	2.141321	0.000025

11	1	0	2.899792	-0.000031	0.000019
12	8	0	-2.340368	-0.000003	-0.000006

Rotational constants (GHZ): 5.7669783 2.7084259 1.8429131

Thymol
Electronic Energy -464.604235
Stoichiometry C10H14O
Framework group C1[X(C10H14O)]
Deg. of freedom 69
Full point group C1 NOp 1
Largest Abelian subgroup C1 NOp 1
Largest concise Abelian subgroup C1 NOp 1
Standard orientation:

Center Number	Atomic Number	Atomic Type	Coordinates (Angstroms) X	Y	Z
1	6	0	-2.100065	-0.022229	-0.192083
2	1	0	-2.325439	0.776480	-0.903950
3	6	0	-0.588899	-0.134566	-0.122312
4	6	0	0.079867	-1.340021	0.049265
5	6	0	0.202866	1.018808	-0.200993
6	6	0	1.467182	-1.411953	0.140870
7	1	0	-0.491986	-2.257732	0.110509
8	6	0	1.586022	0.956957	-0.115506
9	6	0	2.240788	-0.261155	0.058799
10	1	0	1.946531	-2.375139	0.273850
11	1	0	2.162944	1.874741	-0.186385
12	6	0	-2.672610	0.401846	1.167327
13	1	0	-3.753328	0.551732	1.100547
14	1	0	-2.484186	-0.370094	1.918851
15	1	0	-2.220856	1.332608	1.513955
16	6	0	-2.787352	-1.292645	-0.690129
17	1	0	-3.855286	-1.106974	-0.824230
18	1	0	-2.379101	-1.623622	-1.647644
19	1	0	-2.688392	-2.114742	0.023192
20	6	0	3.742564	-0.310531	0.150377
21	1	0	4.206148	0.088153	-0.755274
22	1	0	4.106076	0.287131	0.989837
23	1	0	4.094487	-1.333451	0.287194
24	8	0	-0.442435	2.214246	-0.368821
25	1	0	0.199685	2.929868	-0.423564

Rotational constants (GHZ): 2.0457303 0.7400549 0.5938891

Thymolate
Electronic Energy -464.121875
Stoichiometry C10H13O(1-)
Framework group C1[X(C10H13O)]
Deg. of freedom 66
Full point group C1 NOp 1
Largest Abelian subgroup C1 NOp 1
Largest concise Abelian subgroup C1 NOp 1
Standard orientation:

Center Number	Atomic Number	Atomic Type	Coordinates (Angstroms) X	Y	Z
1	6	0	-2.085488	-0.034325	-0.220176
2	1	0	-2.286260	0.700279	-1.008192
3	6	0	-0.578375	-0.132365	-0.126126
4	6	0	0.099893	-1.320371	0.067256
5	6	0	0.155059	1.113030	-0.228058
6	6	0	1.493277	-1.387660	0.171473
7	1	0	-0.462922	-2.247590	0.142234
8	6	0	1.577922	0.996592	-0.122168
9	6	0	2.232555	-0.208042	0.070542
10	1	0	1.989369	-2.341008	0.325584
11	1	0	2.144182	1.921641	-0.197626
12	6	0	-2.676829	0.549692	1.069876

13	1	0	-3.757133	0.716109	0.973575
14	1	0	-2.510130	-0.132092	1.911183
15	1	0	-2.195806	1.503849	1.286599
16	6	0	-2.786765	-1.341406	-0.585991
17	1	0	-3.858960	-1.171641	-0.731008
18	1	0	-2.381292	-1.769178	-1.506874
19	1	0	-2.681341	-2.092084	0.204262
20	6	0	3.741612	-0.251093	0.130105
21	1	0	4.176051	-0.368740	-0.868991
22	1	0	4.147835	0.670297	0.555490
23	1	0	4.091598	-1.089622	0.739197
24	8	0	-0.431545	2.224433	-0.398229

Rotational constants (GHZ): 2.1110515 0.7464748 0.5964394

- 1H_2O/B3LYP 6-311G+dp/SMD

H_2O
Electronic Energy -152.948642
Stoichiometry H4O2
Framework group C1[X(H4O2)]
Deg. of freedom 12
Full point group C1 NOp 1
Largest Abelian subgroup C1 NOp 1
Largest concise Abelian subgroup C1 NOp 1
Standard orientation:

Center Number	Atomic Number	Atomic Type	Coordinates (Angstroms) X	Y	Z
1	8	0	1.351337	0.000190	0.097379
2	1	0	1.788612	0.765301	-0.295658
3	1	0	1.789643	-0.765826	-0.292737
4	8	0	-1.500404	-0.000257	-0.119531
5	1	0	-1.853959	0.002155	0.777237
6	1	0	-0.531755	-0.001099	-0.011622

Rotational constants (GHZ): 223.3589304 6.6026795 6.5940576

OH^-
Electronic Energy -152.462455
Stoichiometry H3O2(1-)
Framework group C1[X(H3O2)]
Deg. of freedom 9
Full point group C1 NOp 1
Largest Abelian subgroup C1 NOp 1
Largest concise Abelian subgroup C1 NOp 1
Standard orientation:

Center Number	Atomic Number	Atomic Type	Coordinates (Angstroms) X	Y	Z
1	8	0	1.286943	0.084111	-0.074999
2	1	0	1.649047	-0.511239	0.590619
3	8	0	-1.265979	-0.096278	-0.055426
4	1	0	-1.583273	0.634103	0.487062
5	1	0	-0.233481	-0.025523	-0.034279

Rotational constants (GHZ): 321.6601960 8.6825052 8.6569125

Phenol
Electronic Energy -384.045841
Stoichiometry C6H8O2
Framework group C1[X(C6H8O2)]
Deg. of freedom 42
Full point group C1 NOp 1
Largest Abelian subgroup C1 NOp 1
Largest concise Abelian subgroup C1 NOp 1

Standard orientation:

Center Number	Atomic Number	Atomic Type	Coordinates (Angstroms) X	Y	Z
1	6	0	0.017629	0.852135	-0.013131
2	6	0	0.080231	-0.541883	-0.007240
3	6	0	-1.091622	-1.323582	0.009901
4	6	0	-2.306420	-0.639177	0.013842
5	6	0	-2.375793	0.755394	0.002848
6	6	0	-1.210463	1.520893	-0.008032
7	1	0	0.945006	1.418120	-0.023272
8	1	0	-3.231584	-1.203625	0.029163
9	1	0	-3.344070	1.244736	0.005838
10	8	0	1.278017	-1.201331	-0.019232
11	1	0	2.017150	-0.570381	-0.005623
12	8	0	3.581293	0.486939	0.013293
13	1	0	4.177502	0.427315	-0.740617
14	1	0	4.141374	0.456849	0.796140
15	1	0	-1.244342	2.590324	0.000341
16	1	0	-1.016884	-2.390883	-0.003594

Rotational constants (GHZ): 4.2437161 1.0980493 0.8759908

Phenolate
Electronic Energy -383.574413
Stoichiometry C6H7O2(1-)
Framework group C1[X(C6H7O2)]
Deg. of freedom 39
Full point group C1 NOp 1
Largest Abelian subgroup C1 NOp 1
Largest concise Abelian subgroup C1 NOp 1
Standard orientation:

Center Number	Atomic Number	Atomic Type	Coordinates (Angstroms) X	Y	Z
1	6	0	0.061117	0.955713	-0.079658
2	6	0	-0.233249	-0.442900	-0.073693
3	6	0	0.901151	-1.335862	0.014026
4	6	0	2.185324	-0.808433	0.079679
5	6	0	2.436318	0.570893	0.064114
6	6	0	1.357190	1.459759	-0.019130
7	1	0	-0.785990	1.634950	-0.135871
8	1	0	3.035425	-1.482594	0.154208
9	1	0	3.455308	0.943432	0.122331
10	8	0	-1.435510	-0.900061	-0.140253
11	8	0	-3.729383	0.437692	0.040778
12	1	0	-4.013116	0.083105	0.887373
13	1	0	-2.839888	-0.004886	-0.097329
14	1	0	1.527130	2.515373	-0.060341
15	1	0	0.693169	-2.385454	0.013399

Rotational constants (GHZ): 4.7036232 1.0819473 0.8841047

Thymol
Electronic Energy -541.343987
Stoichiometry C10H16O2
Framework group C1[X(C10H16O2)]
Deg. of freedom 78
Full point group C1 NOp 1
Largest Abelian subgroup C1 NOp 1
Largest concise Abelian subgroup C1 NOp 1
Standard orientation:

Center Number	Atomic Number	Atomic Type	Coordinates (Angstroms) X	Y	Z
1	6	0	1.513297	-0.212673	0.001548
2	6	0	0.290876	0.454289	-0.088394
3	6	0	-0.926000	-0.255129	-0.111320
4	6	0	-0.847417	-1.645064	-0.033449

5	6	0	0.372323	-2.318053	0.061849
6	6	0	1.572032	-1.607739	0.077800
7	1	0	2.429229	0.371625	0.013183
8	1	0	-1.760800	-2.228344	-0.050236
9	1	0	0.384573	-3.401290	0.120767
10	8	0	0.226875	1.818479	-0.156184
11	1	0	1.119005	2.204097	-0.157983
12	6	0	-2.241653	0.507004	-0.185037
13	1	0	-2.061890	1.380429	-0.819866
14	6	0	-2.646019	1.035795	1.206578
15	1	0	-1.856185	1.656825	1.633648
16	1	0	-3.556713	1.640210	1.141501
17	1	0	-2.838472	0.205728	1.893979
18	6	0	-3.388636	-0.295850	-0.814150
19	1	0	-4.265856	0.344818	-0.942729
20	1	0	-3.112666	-0.691312	-1.795477
21	1	0	-3.694021	-1.136971	-0.184091
22	6	0	2.905283	-2.313111	0.156358
23	1	0	3.446534	-2.244943	-0.793523
24	1	0	3.545764	-1.873155	0.926541
25	1	0	2.778552	-3.372470	0.389379
26	8	0	2.757321	3.142523	-0.168261
27	1	0	2.938176	3.746129	0.559991
28	1	0	3.006688	3.613796	-0.970236

Rotational constants (GHZ): 0.9273184 0.6662872 0.4149415

Thymolate

Electronic Energy -540.870574

Stoichiometry C10H15O2(1-)

Framework group C1[X(C10H15O2)]

Deg. of freedom 75

Full point group C1 NOp 1

Largest Abelian subgroup C1 NOp 1

Largest concise Abelian subgroup C1 NOp 1

Standard orientation:

Center Number	Atomic Number	Atomic Type	Coordinates (Angstroms) X	Y	Z
1	6	0	-1.604135	0.085514	0.048441
2	6	0	-0.263055	0.574629	-0.022799
3	6	0	0.779506	-0.424364	-0.106575
4	6	0	0.439307	-1.771901	-0.107148
5	6	0	-0.888573	-2.215003	-0.028573
6	6	0	-1.919327	-1.270277	0.052474
7	1	0	-2.395266	0.829378	0.102212
8	1	0	1.225221	-2.520173	-0.178906
9	1	0	-1.113253	-3.278000	-0.036953
10	8	0	0.019391	1.831225	-0.015731
11	6	0	2.211407	0.078961	-0.174619
12	1	0	2.181415	0.977900	-0.802002
13	6	0	3.208327	-0.908347	-0.797003
14	1	0	2.880069	-1.237885	-1.787790
15	1	0	4.193605	-0.439393	-0.903326
16	1	0	3.340994	-1.801857	-0.176023
17	6	0	2.701361	0.541126	1.213236
18	1	0	3.700003	0.994122	1.151609
19	1	0	2.010859	1.283021	1.617729
20	1	0	2.753961	-0.306781	1.906647
21	6	0	-3.361189	-1.717726	0.177957
22	1	0	-3.673418	-1.773969	1.228637
23	1	0	-4.040057	-1.020899	-0.323370
24	1	0	-3.510143	-2.710364	-0.259893
25	8	0	-1.638189	3.903375	-0.222697
26	1	0	-1.356947	4.200673	-1.091879
27	1	0	-1.068445	3.091760	-0.071623

Rotational constants (GHZ): 0.8770655 0.7143073 0.4215868

- 1H_2O/B3LYP 6-311G+dp/PCM

H_2O

Electronic Energy -152.948642
Stoichiometry H4O2
Framework group C1[X(H4O2)]
Deg. of freedom 12
Full point group C1 NOp 1
Largest Abelian subgroup C1 NOp 1
Largest concise Abelian subgroup C1 NOp 1
Standard orientation:

Center Number	Atomic Number	Atomic Type	Coordinates (Angstroms) X	Y	Z
1	8	0	1.354996	-0.000069	0.095350
2	1	0	1.785425	0.769030	-0.288545
3	1	0	1.787533	-0.767656	-0.289197
4	8	0	-1.498925	0.000064	-0.119702
5	1	0	-1.882099	0.000036	0.759773
6	1	0	-0.539428	-0.001371	0.012785

Rotational constants (GHZ): 226.2594154 6.5900974 6.5775524

OH^-

Electronic Energy -152.462455
Stoichiometry H3O2(1-)
Framework group C1[X(H3O2)]
Deg. of freedom 9
Full point group C1 NOp 1
Largest Abelian subgroup C1 NOp 1
Largest concise Abelian subgroup C1 NOp 1
Standard orientation:

Center Number	Atomic Number	Atomic Type	Coordinates (Angstroms) X	Y	Z
1	8	0	1.286943	0.084111	-0.074999
2	1	0	1.649047	-0.511239	0.590619
3	8	0	-1.265979	-0.096278	-0.055426
4	1	0	-1.583273	0.634103	0.487062
5	1	0	-0.233481	-0.025523	-0.034279

Rotational constants (GHZ): 321.6601960 8.6825052 8.6569125

Phenol

Electronic Energy -384.045841
Stoichiometry C6H8O2
Framework group C1[X(C6H8O2)]
Deg. of freedom 42
Full point group C1 NOp 1
Largest Abelian subgroup C1 NOp 1
Largest concise Abelian subgroup C1 NOp 1
Standard orientation:

Center Number	Atomic Number	Atomic Type	Coordinates (Angstroms) X	Y	Z
1	6	0	2.299681	-0.636070	0.000103
2	6	0	1.074936	-1.283268	0.000036
3	6	0	-0.101809	-0.539616	-0.000071
4	6	0	-0.041477	0.851868	-0.000125
5	6	0	1.192748	1.488022	-0.000058
6	6	0	2.369476	0.752629	0.000063
7	1	0	3.210615	-1.223439	0.000176
8	1	0	1.007148	-2.364014	0.000051
9	1	0	-0.960991	1.426349	-0.000233
10	1	0	1.230377	2.571314	-0.000094
11	1	0	3.329412	1.253567	0.000127
12	8	0	-1.276510	-1.217316	-0.000126
13	1	0	-2.029068	-0.603808	-0.000262

14	8	0	-3.518559	0.486473	0.000071
15	1	0	-4.094700	0.492744	-0.769827
16	1	0	-4.093570	0.492634	0.770816

Rotational constants (GHZ): 4.3158479 1.1226857 0.8947008
Standard basis: 6-311+G(d,p) (5D, 7F)

Phenolate
Electronic Energy -383.574575
Stoichiometry C6H7O2(1-)
Framework group C1[X(C6H7O2)]
Deg. of freedom 39
Full point group C1 NOp 1
Largest Abelian subgroup C1 NOp 1
Largest concise Abelian subgroup C1 NOp 1
Standard orientation:

Center Number	Atomic Number	Atomic Type	Coordinates (Angstroms) X	Y	Z
1	6	0	2.211717	-0.759237	0.053555
2	6	0	0.931431	-1.304689	0.014124
3	6	0	-0.227497	-0.482554	-0.043222
4	6	0	-0.004295	0.921976	-0.059414
5	6	0	1.281708	1.455034	-0.019265
6	6	0	2.406546	0.625927	0.037688
7	1	0	3.069701	-1.423838	0.097404
8	1	0	0.796763	-2.382443	0.027412
9	1	0	-0.864577	1.582608	-0.104799
10	1	0	1.407828	2.533795	-0.033217
11	1	0	3.405193	1.046345	0.069005
12	8	0	-1.438051	-1.003400	-0.080390
13	8	0	-3.649138	0.443109	-0.021830
14	1	0	-3.932333	0.451598	0.879969
15	1	0	-2.782720	-0.064481	-0.018819

Rotational constants (GHZ): 4.6476062 1.1131429 0.9013112

Thymol
Electronic Energy -541.341186
Stoichiometry C10H16O2
Framework group C1[X(C10H16O2)]
Deg. of freedom 78
Full point group C1 NOp 1
Largest Abelian subgroup C1 NOp 1
Largest concise Abelian subgroup C1 NOp 1
Standard orientation:

Center Number	Atomic Number	Atomic Type	Coordinates (Angstroms) X	Y	Z
1	6	0	-2.241832	0.506419	-0.183581
2	1	0	-2.063079	1.379046	-0.819851
3	6	0	-0.926202	-0.255893	-0.111356
4	6	0	-0.847102	-1.645812	-0.037857
5	6	0	0.290811	0.453828	-0.088650
6	6	0	0.373232	-2.318737	0.054089
7	1	0	-1.760087	-2.229583	-0.057336
8	6	0	1.513325	-0.212798	-0.001993
9	6	0	1.572502	-1.608236	0.072006
10	1	0	0.385960	-3.402212	0.107767
11	1	0	2.429211	0.371673	0.007682
12	6	0	-2.643365	1.037131	1.208166
13	1	0	-3.553891	1.641885	1.143940
14	1	0	-2.835009	0.207942	1.896846
15	1	0	-1.852483	1.658301	1.633036
16	6	0	-3.390446	-0.296724	-0.809285
17	1	0	-4.267719	0.344128	-0.936535
18	1	0	-3.116900	-0.693316	-1.790834
19	1	0	-3.694638	-1.137032	-0.177569
20	6	0	2.905900	-2.311533	0.165550

21	1	0	3.533903	-2.092322	-0.704011
22	1	0	3.461581	-1.995424	1.054240
23	1	0	2.777869	-3.394598	0.219917
24	8	0	0.226114	1.818075	-0.155711
25	1	0	1.118115	2.204058	-0.158380
26	8	0	2.755820	3.143125	-0.170666
27	1	0	2.939674	3.742420	0.560436
28	1	0	3.001088	3.619554	-0.970865

Rotational constants (GHZ): 0.9272535 0.6663954 0.4149521
Standard basis: 6-311+G(d,p) (5D, 7F)

Thymolate
Electronic Energy -540.864600
Stoichiometry C10H15O2(1-)
Framework group C1[X(C10H15O2)]
Deg. of freedom 75
Full point group C1 NOp 1
Largest Abelian subgroup C1 NOp 1
Largest concise Abelian subgroup C1 NOp 1
Standard orientation:

Center Number	Atomic Number	Atomic Type	Coordinates (Angstroms) X	Y	Z
1	6	0	-2.211037	0.080903	-0.184138
2	1	0	-2.177088	0.976012	-0.816695
3	6	0	-0.780029	-0.423735	-0.106937
4	6	0	-0.442832	-1.771809	-0.087467
5	6	0	0.263641	0.574202	-0.023188
6	6	0	0.883529	-2.216540	0.007099
7	1	0	-1.230443	-2.519385	-0.147344
8	6	0	1.602990	0.083383	0.064772
9	6	0	1.915824	-1.272984	0.079989
10	1	0	1.105542	-3.280037	0.021446
11	1	0	2.394577	0.826238	0.125548
12	6	0	-2.707048	0.551464	1.198670
13	1	0	-3.704951	1.005065	1.129834
14	1	0	-2.763754	-0.292429	1.896639
15	1	0	-2.017700	1.294969	1.602194
16	6	0	-3.205755	-0.909188	-0.805697
17	1	0	-4.189842	-0.439754	-0.920661
18	1	0	-2.872198	-1.245599	-1.792402
19	1	0	-3.343132	-1.798483	-0.179728
20	6	0	3.361737	-1.721355	0.137123
21	1	0	3.815867	-1.739362	-0.861887
22	1	0	3.964420	-1.046589	0.753010
23	1	0	3.449961	-2.730169	0.553828
24	8	0	-0.017179	1.831225	-0.025175
25	8	0	1.645887	3.900937	-0.211246
26	1	0	1.380124	4.195923	-1.086056
27	1	0	1.072831	3.090259	-0.067720

Rotational constants (GHZ): 0.8776610 0.7144013 0.4213500

- 1H₂O/B3LYP 6-311G+dp/CPCM

H_2O
Electronic Energy -152.939271
Stoichiometry H4O2
Framework group CS[SG(H4O2)]
Deg. of freedom 9
Full point group CS NOp 2
Largest Abelian subgroup CS NOp 2
Largest concise Abelian subgroup C1 NOp 1

Standard orientation:

Center Number	Atomic Number	Atomic Type	Coordinates (Angstroms) X	Y	Z
1	8	0	0.043084	-1.322998	0.000000
2	1	0	0.863967	-1.830672	0.000000
3	1	0	-0.663929	-1.980160	0.000000
4	8	0	0.043084	1.506358	0.000000
5	1	0	-0.017438	0.533042	0.000000
6	1	0	-0.871948	1.810910	0.000000

Rotational constants (GHZ): 256.9766853 6.7208756 6.5495802

OH^-
Electronic Energy -152.446206
Stoichiometry H3O2(1-)
Framework group C1[X(H3O2)]
Deg. of freedom 9
Full point group C1 NOp 1
Largest Abelian subgroup C1 NOp 1
Largest concise Abelian subgroup C1 NOp 1
Standard orientation:

Center Number	Atomic Number	Atomic Type	Coordinates (Angstroms) X	Y	Z
1	8	0	-1.265642	-0.095902	-0.055845
2	1	0	-0.233004	-0.024905	-0.035014
3	1	0	-1.582945	0.632042	0.490001
4	8	0	1.287035	0.083728	-0.075323
5	1	0	1.644805	-0.509741	0.594352

Rotational constants (GHZ): 321.1116234 8.6850650 8.6616058s

Phenol
Electronic Energy -307.093078
Stoichiometry C6H8O2
Framework group C1[X(C6H8O2)]
Deg. of freedom 42
Full point group C1 NOp 1
Largest Abelian subgroup C1 NOp 1
Largest concise Abelian subgroup C1 NOp 1
Standard orientation:

Center Number	Atomic Number	Atomic Type	Coordinates (Angstroms) X	Y	Z
1	6	0	-0.012594	0.902009	0.003906
2	6	0	0.115152	-0.490796	0.007013
3	6	0	-1.026996	-1.297642	0.002636
4	6	0	-2.289777	-0.709773	-0.004235
5	6	0	-2.427730	0.680024	-0.007152
6	6	0	-1.283113	1.477825	-0.003105
7	1	0	0.875100	1.524337	0.006930
8	1	0	-3.170102	-1.343193	-0.007621
9	1	0	-3.412097	1.133213	-0.012575
10	8	0	1.337089	-1.117632	0.014882
11	1	0	2.077163	-0.467969	0.006611
12	8	0	3.569165	0.494075	-0.036885
13	1	0	4.209970	0.087283	-0.634936
14	1	0	4.008049	0.502275	0.823971
15	1	0	-1.373862	2.558574	-0.005288
16	1	0	-0.913908	-2.375947	0.004547

Rotational constants (GHZ): 4.4388152 1.0905213 0.8788403

Phenolate
Electronic Energy -383.566995
Stoichiometry C6H7O2(1-)
Framework group C1[X(C6H7O2)]
Deg. of freedom 39

Full point group C1 NOp 1
Largest Abelian subgroup C1 NOp 1
Largest concise Abelian subgroup C1 NOp 1
Standard orientation:

Center Number	Atomic Number	Atomic Type	Coordinates (Angstroms) X	Y	Z
1	6	0	-0.001738	0.923763	-0.040080
2	6	0	-0.227116	-0.480495	-0.031117
3	6	0	0.930970	-1.304913	0.008169
4	6	0	2.212522	-0.761822	0.037600
5	6	0	2.409527	0.623107	0.028979
6	6	0	1.285528	1.454419	-0.010131
7	1	0	-0.861344	1.586088	-0.071410
8	1	0	3.069819	-1.428082	0.067651
9	1	0	3.409180	1.041645	0.052247
10	8	0	-1.438865	-0.999195	-0.058998
11	8	0	-3.644452	0.456651	-0.042325
12	1	0	-4.034996	0.323718	0.828890
13	1	0	-2.782220	-0.057897	-0.024777
14	1	0	1.413363	2.533044	-0.018105
15	1	0	0.794584	-2.382519	0.015572

Rotational constants (GHZ): 4.6564646 1.1119832 0.9002697

Thymol

Electronic Energy -541.341260
Stoichiometry C10H16O2
Framework group C1[X(C10H16O2)]
Deg. of freedom 78
Full point group C1 NOp 1
Largest Abelian subgroup C1 NOp 1
Largest concise Abelian subgroup C1 NOp 1
Standard orientation:

Center Number	Atomic Number	Atomic Type	Coordinates (Angstroms) X	Y	Z
1	6	0	1.536889	-0.193111	0.001718
2	6	0	0.305894	0.460410	-0.081382
3	6	0	-0.904606	-0.261363	-0.107661
4	6	0	-0.809738	-1.652963	-0.034062
5	6	0	0.418179	-2.313000	0.056726
6	6	0	1.612188	-1.588601	0.072550
7	1	0	2.446085	0.399685	0.014264
8	1	0	-1.717023	-2.245986	-0.048425
9	1	0	0.443472	-3.396224	0.112983
10	8	0	0.235816	1.833487	-0.139783
11	1	0	1.131967	2.237611	-0.133441
12	6	0	-2.237846	0.472379	-0.171396
13	1	0	-2.074973	1.382433	-0.756348
14	6	0	-2.689935	0.907799	1.236773
15	1	0	-1.929088	1.515500	1.733305
16	1	0	-3.610141	1.497992	1.180470
17	1	0	-2.887317	0.032835	1.864761
18	6	0	-3.348728	-0.324984	-0.867700
19	1	0	-4.239508	0.300508	-0.976579
20	1	0	-3.044278	-0.653402	-1.865689
21	1	0	-3.639671	-1.209540	-0.293614
22	6	0	2.952408	-2.278034	0.146847
23	1	0	3.490656	-2.196930	-0.803612
24	1	0	3.588705	-1.829894	0.915427
25	1	0	2.837286	-3.339763	0.374790
26	8	0	2.628463	3.229702	-0.154151
27	1	0	2.581616	3.927080	0.513249
28	1	0	2.699759	3.701391	-0.994542

Rotational constants (GHZ): 0.9155426 0.6726760 0.4165496

Thymolate
Electronic Energy -541.341260
Stoichiometry C10H16O2
Framework group C1[X(C10H16O2)]
Deg. of freedom 78
Full point group C1 NOp 1
Largest Abelian subgroup C1 NOp 1
Largest concise Abelian subgroup C1 NOp 1
Standard orientation:

Center Number	Atomic Number	Atomic Type	Coordinates (Angstroms) X	Y	Z
1	6	0	1.536889	-0.193111	0.001718
2	6	0	0.305894	0.460410	-0.081382
3	6	0	-0.904606	-0.261363	-0.107661
4	6	0	-0.809738	-1.652963	-0.034062
5	6	0	0.418179	-2.313000	0.056726
6	6	0	1.612188	-1.588601	0.072550
7	1	0	2.446085	0.399685	0.014264
8	1	0	-1.717023	-2.245986	-0.048425
9	1	0	0.443472	-3.396224	0.112983
10	8	0	0.235816	1.833487	-0.139783
11	1	0	1.131967	2.237611	-0.133441
12	6	0	-2.237846	0.472379	-0.171396
13	1	0	-2.074973	1.382433	-0.756348
14	6	0	-2.689935	0.907799	1.236773
15	1	0	-1.929088	1.515500	1.733305
16	1	0	-3.610141	1.497992	1.180470
17	1	0	-2.887317	0.032835	1.864761
18	6	0	-3.348728	-0.324984	-0.867700
19	1	0	-4.239508	0.300508	-0.976579
20	1	0	-3.044278	-0.653402	-1.865689
21	1	0	-3.639671	-1.209540	-0.293614
22	6	0	2.952408	-2.278034	0.146847
23	1	0	3.490656	-2.196930	-0.803612
24	1	0	3.588705	-1.829894	0.915427
25	1	0	2.837286	-3.339763	0.374790
26	8	0	2.628463	3.229702	-0.154151
27	1	0	2.581616	3.927080	0.513249
28	1	0	2.699759	3.701391	-0.994542

Rotational constants (GHZ): 0.9155426 0.6726760 0.4165496

- 1H_2O/B3PW91 6-311G+dp/SMD

H_2O
Electronic Energy -152.886443
Stoichiometry H4O2
Framework group CS[SG(H4O2)]
Deg. of freedom 9
Full point group CS NOp 2
Largest Abelian subgroup CS NOp 2
Largest concise Abelian subgroup C1 NOp 1
Standard orientation:

Center Number	Atomic Number	Atomic Type	Coordinates (Angstroms) X	Y	Z
1	8	0	0.042979	-1.310810	0.000000
2	1	0	0.856448	-1.822131	0.000000
3	1	0	-0.668011	-1.957162	0.000000
4	8	0	0.042979	1.494760	0.000000
5	1	0	-0.003287	0.524076	0.000000
6	1	0	-0.872813	1.783614	0.000000

Rotational constants (GHZ): 257.6563606 6.8393964 6.6625416

OH⁻

Electronic Energy -152.398046
Stoichiometry H3O2(1-)
Framework group C1[X(H3O2)]
Deg. of freedom 9
Full point group C1 NOp 1
Largest Abelian subgroup C1 NOp 1
Largest concise Abelian subgroup C1 NOp 1
Standard orientation:

Center Number	Atomic Number	Atomic Type	Coordinates (Angstroms) X	Y	Z
1	8	0	-1.272942	-0.097719	-0.051169
2	1	0	-0.255045	-0.030517	-0.034728
3	1	0	-1.584111	0.651130	0.461892
4	8	0	1.294469	0.080563	-0.077675
5	1	0	1.666939	-0.483366	0.603582

Rotational constants (GHZ): 325.4595069 8.5856481 8.5606474

Phenol

Electronic Energy -383.909558
Stoichiometry C6H8O2
Framework group C1[X(C6H8O2)]
Deg. of freedom 42
Full point group C1 NOp 1
Largest Abelian subgroup C1 NOp 1
Largest concise Abelian subgroup C1 NOp 1
Standard orientation:

Center Number	Atomic Number	Atomic Type	Coordinates (Angstroms) X	Y	Z
1	6	0	0.000762	0.891143	0.001746
2	6	0	0.117993	-0.496199	0.005679
3	6	0	-1.023895	-1.292530	0.003289
4	6	0	-2.277735	-0.698911	-0.002436
5	6	0	-2.405945	0.686156	-0.006136
6	6	0	-1.260849	1.473063	-0.004055
7	1	0	0.892903	1.506109	0.003079
8	1	0	-3.161897	-1.325828	-0.004248
9	1	0	-3.386658	1.145616	-0.010633
10	8	0	1.331806	-1.125248	0.012348
11	1	0	2.075401	-0.480402	0.005527
12	8	0	3.518483	0.492134	-0.033283
13	1	0	4.175614	0.124055	-0.636968
14	1	0	3.961400	0.536118	0.823222
15	1	0	-1.344687	2.553692	-0.006907
16	1	0	-0.916373	-2.370780	0.005891

Rotational constants (GHZ): 4.4550873 1.1135613 0.8943871

Phenolate

Electronic Energy -383.435157
Stoichiometry C6H7O2(1-)
Framework group C1[X(C6H7O2)]
Deg. of freedom 39
Full point group C1 NOp 1
Largest Abelian subgroup C1 NOp 1
Largest concise Abelian subgroup C1 NOp 1
Standard orientation:

Center Number	Atomic Number	Atomic Type	Coordinates (Angstroms) X	Y	Z
1	6	0	-0.013052	0.914708	-0.040474
2	6	0	-0.228561	-0.483276	-0.030988
3	6	0	0.925250	-1.299963	0.008502
4	6	0	2.199333	-0.753455	0.037758
5	6	0	2.389484	0.626336	0.028802

6	6	0	1.266612	1.449092	-0.010624
7	1	0	-0.876279	1.570981	-0.072233
8	1	0	3.058722	-1.415614	0.068087
9	1	0	3.386592	1.048991	0.052078
10	8	0	-1.435476	-1.004689	-0.058785
11	8	0	-3.595381	0.462367	-0.041306
12	1	0	-3.996254	0.331757	0.823968
13	1	0	-2.741546	-0.068583	-0.026226
14	1	0	1.389348	2.527439	-0.018933
15	1	0	0.791876	-2.377050	0.016133

Rotational constants (GHZ): 4.6770234 1.1349725 0.9160416

Thymol
Electronic Energy -541.164144
Stoichiometry C10H16O2
Framework group C1[X(C10H16O2)]
Deg. of freedom 78
Full point group C1 NOp 1
Largest Abelian subgroup C1 NOp 1
Largest concise Abelian subgroup C1 NOp 1
Standard orientation:

Center Number	Atomic Number	Atomic Type	Coordinates (Angstroms) X	Y	Z
1	6	0	1.529590	-0.172414	0.004903
2	6	0	0.296197	0.461624	-0.083415
3	6	0	-0.899135	-0.272445	-0.113123
4	6	0	-0.789493	-1.656185	-0.040685
5	6	0	0.442036	-2.298182	0.054712
6	6	0	1.620468	-1.561330	0.075766
7	1	0	2.430896	0.431300	0.020466
8	1	0	-1.688897	-2.259727	-0.060229
9	1	0	0.482024	-3.380314	0.110309
10	8	0	0.208465	1.826618	-0.145735
11	1	0	1.097135	2.245634	-0.140078
12	6	0	-2.232878	0.447369	-0.183926
13	1	0	-2.086837	1.329643	-0.812635
14	6	0	-2.657137	0.937784	1.206034
15	1	0	-1.891769	1.569691	1.660835
16	1	0	-3.581944	1.517777	1.144179
17	1	0	-2.835318	0.088669	1.872278
18	6	0	-3.346561	-0.385913	-0.814273
19	1	0	-4.241974	0.228107	-0.936848
20	1	0	-3.060473	-0.764596	-1.798466
21	1	0	-3.620073	-1.239297	-0.188710
22	6	0	2.965153	-2.230897	0.156717
23	1	0	3.510617	-2.129723	-0.785686
24	1	0	3.584096	-1.783367	0.937693
25	1	0	2.861754	-3.295610	0.370414
26	8	0	2.570413	3.205243	-0.164455
27	1	0	2.571173	3.893423	0.512434
28	1	0	2.669118	3.677038	-1.000703

Rotational constants (GHZ): 0.9316196 0.6818526 0.4217409

Thymolate
Electronic Energy -540.687949
Stoichiometry C10H15O2(1-)
Framework group C1[X(C10H15O2)]
Deg. of freedom 75
Full point group C1 NOp 1
Largest Abelian subgroup C1 NOp 1
Largest concise Abelian subgroup C1 NOp 1
Standard orientation:

Center Number	Atomic Number	Atomic Type	Coordinates (Angstroms) X	Y	Z
1	6	0	-1.572919	-0.004228	0.042850
2	6	0	-0.287717	0.570611	-0.037248

3	6	0	0.830926	-0.312396	-0.109413
4	6	0	0.599679	-1.680433	-0.090243
5	6	0	-0.682281	-2.224992	-0.003555
6	6	0	-1.785515	-1.379691	0.064679
7	1	0	-2.424547	0.668428	0.091218
8	1	0	1.443299	-2.359699	-0.148756
9	1	0	-0.817552	-3.301061	0.005321
10	8	0	-0.128784	1.878335	-0.049286
11	6	0	2.228170	0.274503	-0.173860
12	1	0	2.158785	1.188161	-0.771518
13	6	0	3.258603	-0.634510	-0.841338
14	1	0	2.935086	-0.950849	-1.836226
15	1	0	4.208476	-0.104489	-0.949590
16	1	0	3.454082	-1.532657	-0.249400
17	6	0	2.714266	0.682208	1.223088
18	1	0	3.683737	1.186229	1.168228
19	1	0	2.009394	1.359018	1.710160
20	1	0	2.829898	-0.200730	1.859172
21	6	0	-3.183290	-1.931286	0.174822
22	1	0	-3.599752	-1.754574	1.170834
23	1	0	-3.856699	-1.457405	-0.543459
24	1	0	-3.198169	-3.007739	-0.004472
25	8	0	-2.118852	3.549618	-0.171352
26	1	0	-2.184873	3.820153	-1.092708
27	1	0	-1.379610	2.864871	-0.142401

Rotational constants (GHZ): 0.9341078 0.6990864 0.4295606

- 1H_2O/B3PW91 6-311G+dp/PCM

H_2O
Electronic Energy -152.878150
Stoichiometry H4O2
Framework group C1[X(H4O2)]
Deg. of freedom 12
Full point group C1 NOp 1
Largest Abelian subgroup C1 NOp 1
Largest concise Abelian subgroup C1 NOp 1
Standard orientation:

Center Number	Atomic Number	Atomic Type	Coordinates (Angstroms) X	Y	Z
1	8	0	1.334098	-0.000066	0.089003
2	1	0	1.802929	0.766671	-0.260945
3	1	0	1.805153	-0.765474	-0.260862
4	8	0	-1.492632	-0.000059	-0.117740
5	1	0	-1.818990	0.000437	0.788019
6	1	0	-0.520820	-0.000636	-0.036318

Rotational constants (GHZ): 227.6237525 6.7080289 6.6950899

OH^-
Electronic Energy -152.380924
Stoichiometry H3O2(1-)
Framework group C1[X(H3O2)]
Deg. of freedom 9
Full point group C1 NOp 1
Largest Abelian subgroup C1 NOp 1
Largest concise Abelian subgroup C1 NOp 1
Standard orientation:

Center Number	Atomic Number	Atomic Type	Coordinates (Angstroms) X	Y	Z
1	8	0	1.260773	0.034490	-0.105350
2	1	0	1.669783	-0.137266	0.749182
3	8	0	-1.248911	-0.110104	-0.006106
4	1	0	-1.570442	0.780839	0.161116

5	1	0	-0.194238	-0.038664	-0.018653

Rotational constants (GHZ): 330.2884982 8.9463552 8.9345926

Phenol

Electronic Energy -383.885897
Stoichiometry C6H8O2
Framework group C1[X(C6H8O2)]
Deg. of freedom 42
Full point group C1 NOp 1
Largest Abelian subgroup C1 NOp 1
Largest concise Abelian subgroup C1 NOp 1
Standard orientation:

Center Number	Atomic Number	Atomic Type	Coordinates (Angstroms) X	Y	Z
1	6	0	-2.305940	-0.657742	0.000397
2	6	0	-1.068674	-1.288109	0.000151
3	6	0	0.101481	-0.525797	-0.000195
4	6	0	0.017373	0.869010	-0.000283
5	6	0	-1.229030	1.487799	-0.000036
6	6	0	-2.397611	0.732904	0.000306
7	1	0	-3.208856	-1.260501	0.000663
8	1	0	-0.986813	-2.369531	0.000216
9	1	0	0.927948	1.460676	-0.000537
10	1	0	-1.282413	2.572170	-0.000109
11	1	0	-3.366556	1.219969	0.000497
12	8	0	1.284496	-1.187186	-0.000426
13	1	0	2.026815	-0.559706	-0.000697
14	8	0	3.575561	0.485053	0.000057
15	1	0	4.151302	0.421785	0.767053
16	1	0	4.152531	0.423819	-0.766176

Rotational constants (GHZ): 4.3498922 1.0963247 0.8792435

Phenolate

Electronic Energy -383.409709
Stoichiometry C6H7O2(1-)
Framework group C1[X(C6H7O2)]
Deg. of freedom 39
Full point group C1 NOp 1
Largest Abelian subgroup C1 NOp 1
Largest concise Abelian subgroup C1 NOp 1
Standard orientation:

Center Number	Atomic Number	Atomic Type	Coordinates (Angstroms) X	Y	Z
1	6	0	2.193046	-0.779378	0.092862
2	6	0	0.910938	-1.302351	0.022639
3	6	0	-0.251447	-0.469672	-0.079583
4	6	0	0.019451	0.938229	-0.105316
5	6	0	1.309407	1.442613	-0.034423
6	6	0	2.421796	0.600591	0.066969
7	1	0	3.039715	-1.461411	0.169516
8	1	0	0.749100	-2.377541	0.043092
9	1	0	-0.831079	1.610210	-0.189121
10	1	0	1.456022	2.522237	-0.059862
11	1	0	3.428707	1.004177	0.121588
12	8	0	-1.432997	-0.959144	-0.141818
13	8	0	-3.680683	0.425442	0.046481
14	1	0	-3.730861	0.464492	1.003205
15	1	0	-2.821308	-0.072740	-0.104610

Rotational constants (GHZ): 4.6935869 1.1049544 0.9002358

Thymol

Electronic Energy -541.129084
Stoichiometry C10H16O2
Framework group C1[X(C10H16O2)]

Deg. of freedom 78
Full point group C1 NOp 1
Largest Abelian subgroup C1 NOp 1
Largest concise Abelian subgroup C1 NOp 1
Standard orientation:

Center Number	Atomic Number	Atomic Type	Coordinates (Angstroms) X	Y	Z
1	6	0	-2.233195	0.503821	-0.183742
2	1	0	-2.053842	1.375569	-0.823186
3	6	0	-0.922875	-0.256146	-0.112223
4	6	0	-0.845028	-1.643774	-0.041152
5	6	0	0.291914	0.453190	-0.087643
6	6	0	0.372302	-2.316848	0.051190
7	1	0	-1.760463	-2.225930	-0.063507
8	6	0	1.511656	-0.214496	-0.000401
9	6	0	1.570109	-1.607809	0.071902
10	1	0	0.385068	-3.401377	0.103069
11	1	0	2.428893	0.369703	0.010716
12	6	0	-2.627255	1.033864	1.203462
13	1	0	-3.538874	1.638240	1.144008
14	1	0	-2.814717	0.204629	1.893902
15	1	0	-1.834232	1.655775	1.624651
16	6	0	-3.377779	-0.299440	-0.801491
17	1	0	-4.256048	0.340066	-0.932475
18	1	0	-3.104948	-0.703246	-1.780845
19	1	0	-3.682137	-1.135480	-0.163154
20	6	0	2.898166	-2.309603	0.165816
21	1	0	3.527620	-2.089224	-0.702797
22	1	0	3.452883	-1.995125	1.056078
23	1	0	2.769538	-3.393082	0.218270
24	8	0	0.227537	1.810308	-0.154785
25	1	0	1.120304	2.193731	-0.156193
26	8	0	2.733772	3.153163	-0.170881
27	1	0	2.889274	3.764438	0.554323
28	1	0	2.953126	3.636993	-0.971846

Rotational constants (GHZ): 0.9273236 0.6725540 0.4170877

Thymolate
Electronic Energy -540.650595
Stoichiometry C10H15O2(1-)
Framework group C1[X(C10H15O2)]
Deg. of freedom 75
Full point group C1 NOp 1
Largest Abelian subgroup C1 NOp 1
Largest concise Abelian subgroup C1 NOp 1
Standard orientation:

Center Number	Atomic Number	Atomic Type	Coordinates (Angstroms) X	Y	Z
1	6	0	-2.209121	0.115018	-0.185339
2	1	0	-2.159902	1.011017	-0.818013
3	6	0	-0.790274	-0.406684	-0.108010
4	6	0	-0.470176	-1.756754	-0.097435
5	6	0	0.260689	0.577980	-0.014910
6	6	0	0.848103	-2.216797	-0.001182
7	1	0	-1.268680	-2.493727	-0.166336
8	6	0	1.591235	0.071849	0.076392
9	6	0	1.888426	-1.286318	0.083508
10	1	0	1.059256	-3.283544	0.005911
11	1	0	2.391764	0.805584	0.147529
12	6	0	-2.691195	0.590266	1.193611
13	1	0	-3.683860	1.056772	1.130970
14	1	0	-2.753623	-0.252863	1.892577
15	1	0	-1.988646	1.325433	1.591802
16	6	0	-3.211616	-0.859958	-0.802351
17	1	0	-4.190595	-0.379730	-0.920020
18	1	0	-2.880906	-1.204843	-1.787675

19	1	0	-3.360312	-1.745326	-0.172311
20	6	0	3.324007	-1.749220	0.144921
21	1	0	3.781611	-1.768296	-0.852799
22	1	0	3.931687	-1.083075	0.765841
23	1	0	3.399696	-2.760874	0.558068
24	8	0	-0.008101	1.833856	-0.013344
25	8	0	1.726483	3.824295	-0.221994
26	1	0	1.627050	3.982204	-1.162434
27	1	0	1.107950	3.049768	-0.065628

Rotational constants (GHZ): 0.8943994 0.7159769 0.4257734

- 1H_2O/B3PW91 6-311G+dp/CPCM

H_2O
Electronic Energy -152.877702
Stoichiometry H4O2
Framework group CS[SG(H4O2)]
Deg. of freedom 9
Full point group CS NOp 2
Largest Abelian subgroup CS NOp 2
Largest concise Abelian subgroup C1 NOp 1
Standard orientation:

Center Number	Atomic Number	Atomic Type	Coordinates (Angstroms) X	Y	Z
1	8	0	0.045584	-1.314418	0.000000
2	1	0	0.829499	-1.867307	0.000000
3	1	0	-0.702108	-1.915371	0.000000
4	8	0	0.045584	1.499140	0.000000
5	1	0	0.015429	0.529285	0.000000
6	1	0	-0.872162	1.775611	0.000000

Rotational constants (GHZ): 256.3755065 6.8081813 6.6320635

OH^-
Electronic Energy -152.381017
Stoichiometry H3O2(1-)
Framework group C1[X(H3O2)]
Deg. of freedom 9
Full point group C1 NOp 1
Largest Abelian subgroup C1 NOp 1
Largest concise Abelian subgroup C1 NOp 1
Standard orientation:

Center Number	Atomic Number	Atomic Type	Coordinates (Angstroms) X	Y	Z
1	8	0	-1.256784	-0.104035	0.032085
2	1	0	-0.221801	-0.047540	-0.000815
3	1	0	-1.569288	0.788654	-0.117705
4	8	0	1.268793	-0.007788	-0.109062
5	1	0	1.695009	0.153470	0.734339

Rotational constants (GHZ): 335.7120513 8.8339780 8.8226331

Phenol
Electronic Energy -383.885969
Stoichiometry C6H8O2
Framework group C1[X(C6H8O2)]
Deg. of freedom 42
Full point group C1 NOp 1
Largest Abelian subgroup C1 NOp 1
Largest concise Abelian subgroup C1 NOp 1

Standard orientation:

Center Number	Atomic Number	Atomic Type	Coordinates (Angstroms) X	Y	Z
1	6	0	0.061482	0.850652	-0.007396
2	6	0	0.121125	-0.544696	-0.003013
3	6	0	-1.060190	-1.288230	0.003350
4	6	0	-2.287747	-0.638345	0.005708
5	6	0	-2.355533	0.752912	0.001737
6	6	0	-1.174670	1.488472	-0.004917
7	1	0	0.981180	1.425509	-0.013242
8	1	0	-3.199359	-1.226040	0.010818
9	1	0	-3.315510	1.255645	0.003731
10	8	0	1.294238	-1.221391	-0.004887
11	1	0	2.050503	-0.603454	-0.004518
12	8	0	3.435666	0.500601	-0.000870
13	1	0	4.066478	0.434778	-0.721988
14	1	0	3.962777	0.553636	0.800131
15	1	0	-1.210355	2.572549	-0.008277
16	1	0	-1.001748	-2.370894	0.006580

Rotational constants (GHZ): 4.2914228 1.1538699 0.9132167

Phenolate
Electronic Energy -383.409854
Stoichiometry C6H7O2(1-)
Framework group C1[X(C6H7O2)]
Deg. of freedom 39
Full point group C1 NOp 1
Largest Abelian subgroup C1 NOp 1
Largest concise Abelian subgroup C1 NOp 1
Standard orientation:

Center Number	Atomic Number	Atomic Type	Coordinates (Angstroms) X	Y	Z
1	6	0	-0.037190	0.900741	0.018607
2	6	0	-0.240451	-0.508852	0.021065
3	6	0	0.941603	-1.301860	0.002469
4	6	0	2.206787	-0.731591	-0.016107
5	6	0	2.376047	0.654055	-0.017999
6	6	0	1.234842	1.456843	-0.000245
7	1	0	-0.911611	1.545587	0.031566
8	1	0	3.079089	-1.379692	-0.029903
9	1	0	3.366657	1.094342	-0.032558
10	8	0	-1.421051	-1.042385	0.039367
11	8	0	-3.608882	0.402238	-0.114686
12	1	0	-3.597257	1.003760	0.630907
13	1	0	-2.755437	-0.114664	-0.046295
14	1	0	1.338492	2.538717	-0.001133
15	1	0	0.829705	-2.382888	0.003224

Rotational constants (GHZ): 4.6150800 1.1430330 0.9182562

Thymol
Electronic Energy -541.129162
Stoichiometry C10H16O2
Framework group C1[X(C10H16O2)]
Deg. of freedom 78
Full point group C1 NOp 1
Largest Abelian subgroup C1 NOp 1
Largest concise Abelian subgroup C1 NOp 1
Standard orientation:

Center Number	Atomic Number	Atomic Type	Coordinates (Angstroms) X	Y	Z
1	6	0	1.514706	-0.149226	-0.007248
2	6	0	0.277910	0.484582	-0.100509
3	6	0	-0.915691	-0.258198	-0.120030

4	6	0	-0.802924	-1.641815	-0.035759
5	6	0	0.431762	-2.281292	0.062216
6	6	0	1.608466	-1.539067	0.075918
7	1	0	2.415568	0.457808	0.001212
8	1	0	-1.701744	-2.248566	-0.048442
9	1	0	0.473356	-3.363812	0.125802
10	8	0	0.181142	1.836575	-0.177215
11	1	0	1.066311	2.246778	-0.170027
12	6	0	-2.249713	0.461974	-0.185997
13	1	0	-2.106444	1.338829	-0.825139
14	6	0	-2.651469	0.969033	1.207040
15	1	0	-1.874008	1.606800	1.633753
16	1	0	-3.579320	1.546908	1.155940
17	1	0	-2.813698	0.124964	1.885602
18	6	0	-3.373097	-0.377775	-0.795335
19	1	0	-4.266489	0.238567	-0.927293
20	1	0	-3.090416	-0.780640	-1.771740
21	1	0	-3.649784	-1.215898	-0.148254
22	6	0	2.958771	-2.202487	0.165061
23	1	0	3.525334	-2.061568	-0.760252
24	1	0	3.553772	-1.777873	0.978143
25	1	0	2.860655	-3.275751	0.339065
26	8	0	2.650557	3.060399	-0.156795
27	1	0	2.827037	3.667526	0.566039
28	1	0	2.913953	3.525767	-0.954471

Rotational constants (GHZ): 0.9677892 0.6719645 0.4248944

Thymolate
Electronic Energy -540.650786
Stoichiometry C10H15O2(1-)
Framework group C1[X(C10H15O2)]
Deg. of freedom 75
Full point group C1 NOp 1
Largest Abelian subgroup C1 NOp 1
Largest concise Abelian subgroup C1 NOp 1
Standard orientation:

Center Number	Atomic Number	Atomic Type	Coordinates (Angstroms) X	Y	Z
1	6	0	-1.568571	-0.010475	-0.014161
2	6	0	-0.284269	0.588681	-0.093056
3	6	0	0.843463	-0.298098	-0.112117
4	6	0	0.627658	-1.667605	-0.046890
5	6	0	-0.650797	-2.226865	0.036886
6	6	0	-1.764121	-1.387614	0.056124
7	1	0	-2.432129	0.651642	-0.005710
8	1	0	1.482324	-2.338685	-0.065905
9	1	0	-0.774100	-3.304723	0.082276
10	8	0	-0.134512	1.876687	-0.151672
11	6	0	2.233634	0.304380	-0.165415
12	1	0	2.144936	1.230520	-0.742822
13	6	0	3.276185	-0.582124	-0.849447
14	1	0	2.946431	-0.894905	-1.844453
15	1	0	4.219840	-0.038974	-0.958956
16	1	0	3.488449	-1.484304	-0.266224
17	6	0	2.708588	0.695249	1.241821
18	1	0	3.674274	1.211132	1.204517
19	1	0	1.985642	1.355996	1.726151
20	1	0	2.826488	-0.198522	1.864844
21	6	0	-3.159324	-1.952345	0.176942
22	1	0	-3.526248	-1.874133	1.205890
23	1	0	-3.864810	-1.412158	-0.460232
24	1	0	-3.186240	-3.008390	-0.102656
25	8	0	-2.160909	3.528268	-0.112504
26	1	0	-2.785006	3.224236	-0.772699
27	1	0	-1.411158	2.862530	-0.134740

Rotational constants (GHZ): 0.9440347 0.6947001 0.4302093

- $1H_2O$/wB97XD 6-311G+dp/SMD

H_2O

Electronic Energy -152.896706
Stoichiometry H4O2
Framework group CS[SG(H4O2)]
Deg. of freedom 9
Full point group CS NOp 2
Largest Abelian subgroup CS NOp 2
Largest concise Abelian subgroup C1 NOp 1
Standard orientation:

Center Number	Atomic Number	Atomic Type	Coordinates (Angstroms) X	Y	Z
1	8	0	0.042923	-1.304271	0.000000
2	1	0	0.865162	-1.807645	0.000000
3	1	0	-0.664621	-1.959180	0.000000
4	8	0	0.042923	1.486150	0.000000
5	1	0	-0.019215	0.513336	0.000000
6	1	0	-0.868093	1.798458	0.000000

Rotational constants (GHZ): 257.5224356 6.9032862 6.7230641

OH^-

Electronic Energy -152.407030
Stoichiometry H3O2(1-)
Framework group C1[X(H3O2)]
Deg. of freedom 9
Full point group C1 NOp 1
Largest Abelian subgroup C1 NOp 1
Largest concise Abelian subgroup C1 NOp 1
Standard orientation:

Center Number	Atomic Number	Atomic Type	Coordinates (Angstroms) X	Y	Z
1	8	0	-1.254900	-0.097993	-0.052049
2	1	0	-0.221200	-0.027234	-0.033958
3	1	0	-1.577792	0.645290	0.466560
4	8	0	1.273510	0.081450	-0.077286
5	1	0	1.650111	-0.485711	0.602077

Rotational constants (GHZ): 325.4958404 8.8377839 8.8122087

Phenol

Electronic Energy -383.909558
Stoichiometry C6H8O2
Framework group C1[X(C6H8O2)]
Deg. of freedom 42
Full point group C1 NOp 1
Largest Abelian subgroup C1 NOp 1
Largest concise Abelian subgroup C1 NOp 1
Standard orientation:

Center Number	Atomic Number	Atomic Type	Coordinates (Angstroms) X	Y	Z
1	6	0	0.000762	0.891143	0.001746
2	6	0	0.117993	-0.496199	0.005679
3	6	0	-1.023895	-1.292530	0.003289
4	6	0	-2.277735	-0.698911	-0.002436
5	6	0	-2.405945	0.686156	-0.006136
6	6	0	-1.260849	1.473063	-0.004055
7	1	0	0.892903	1.506109	0.003079
8	1	0	-3.161897	-1.325828	-0.004248
9	1	0	-3.386658	1.145616	-0.010633
10	8	0	1.331806	-1.125248	0.012348
11	1	0	2.075401	-0.480402	0.005527
12	8	0	3.518483	0.492134	-0.033283
13	1	0	4.175614	0.124055	-0.636968

14	1	0	3.961400	0.536118	0.823222
15	1	0	-1.344687	2.553692	-0.006907
16	1	0	-0.916373	-2.370780	0.005891

Rotational constants (GHZ): 4.4550873 1.1135613 0.894387

Phenolate

Electronic Energy -383.435157
Stoichiometry C6H7O2(1-)
Framework group C1[X(C6H7O2)]
Deg. of freedom 39
Full point group C1 NOp 1
Largest Abelian subgroup C1 NOp 1
Largest concise Abelian subgroup C1 NOp 1

Standard orientation:

Center Number	Atomic Number	Atomic Type	Coordinates (Angstroms) X	Y	Z
1	6	0	-0.013052	0.914708	-0.040474
2	6	0	-0.228561	-0.483276	-0.030988
3	6	0	0.925250	-1.299963	0.008502
4	6	0	2.199333	-0.753455	0.037758
5	6	0	2.389484	0.626336	0.028802
6	6	0	1.266612	1.449092	-0.010624
7	1	0	-0.876279	1.570981	-0.072233
8	1	0	3.058722	-1.415614	0.068087
9	1	0	3.386592	1.048991	0.052078
10	8	0	-1.435476	-1.004689	-0.058785
11	8	0	-3.595381	0.462367	-0.041306
12	1	0	-3.996254	0.331757	0.823968
13	1	0	-2.741546	-0.068583	-0.026226
14	1	0	1.389348	2.527439	-0.018933
15	1	0	0.791876	-2.377050	0.016133

Rotational constants (GHZ): 4.6770234 1.1349725 0.9160416

Thymol

Electronic Energy -541.164144
Stoichiometry C10H16O2
Framework group C1[X(C10H16O2)]
Deg. of freedom 78
Full point group C1 NOp 1
Largest Abelian subgroup C1 NOp 1
Largest concise Abelian subgroup C1 NOp 1

Standard orientation:

Center Number	Atomic Number	Atomic Type	Coordinates (Angstroms) X	Y	Z
1	6	0	1.529590	-0.172414	0.004903
2	6	0	0.296197	0.461624	-0.083415
3	6	0	-0.899135	-0.272445	-0.113123
4	6	0	-0.789493	-1.656185	-0.040685
5	6	0	0.442036	-2.298182	0.054712
6	6	0	1.620468	-1.561330	0.075766
7	1	0	2.430896	0.431300	0.020466
8	1	0	-1.688897	-2.259727	-0.060229
9	1	0	0.482024	-3.380314	0.110309
10	8	0	0.208465	1.826618	-0.145735
11	1	0	1.097135	2.245634	-0.140078
12	6	0	-2.232878	0.447369	-0.183926
13	1	0	-2.086837	1.329643	-0.812635
14	6	0	-2.657137	0.937784	1.206034
15	1	0	-1.891769	1.569691	1.660835
16	1	0	-3.581944	1.517777	1.144179
17	1	0	-2.835318	0.088669	1.872278
18	6	0	-3.346561	-0.385913	-0.814273
19	1	0	-4.241974	0.228107	-0.936848
20	1	0	-3.060473	-0.764596	-1.798466
21	1	0	-3.620073	-1.239297	-0.188710

22	6	0	2.965153	-2.230897	0.156717
23	1	0	3.510617	-2.129723	-0.785686
24	1	0	3.584096	-1.783367	0.937693
25	1	0	2.861754	-3.295610	0.370414
26	8	0	2.570413	3.205243	-0.164455
27	1	0	2.571173	3.893423	0.512434
28	1	0	2.669118	3.677038	-1.000703

Rotational constants (GHZ): 0.9316196 0.6818526 0.4217409

Thymolate
Electronic Energy -540.687949
Stoichiometry C10H15O2(1-)
Framework group C1[X(C10H15O2)]
Deg. of freedom 75
Full point group C1 NOp 1
Largest Abelian subgroup C1 NOp 1
Largest concise Abelian subgroup C1 NOp 1
Standard orientation:

Center Number	Atomic Number	Atomic Type	Coordinates (Angstroms) X	Y	Z
1	6	0	-1.572919	-0.004228	0.042850
2	6	0	-0.287717	0.570611	-0.037248
3	6	0	0.830926	-0.312396	-0.109413
4	6	0	0.599679	-1.680433	-0.090243
5	6	0	-0.682281	-2.224992	-0.003555
6	6	0	-1.785515	-1.379691	0.064679
7	1	0	-2.424547	0.668428	0.091218
8	1	0	1.443299	-2.359699	-0.148756
9	1	0	-0.817552	-3.301061	0.005321
10	8	0	-0.128784	1.878335	-0.049286
11	6	0	2.228170	0.274503	-0.173860
12	1	0	2.158785	1.188161	-0.771518
13	6	0	3.258603	-0.634510	-0.841338
14	1	0	2.935086	-0.950849	-1.836226
15	1	0	4.208476	-0.104489	-0.949590
16	1	0	3.454082	-1.532657	-0.249400
17	6	0	2.714266	0.682208	1.223088
18	1	0	3.683737	1.186229	1.168228
19	1	0	2.009394	1.359018	1.710160
20	1	0	2.829898	-0.200730	1.859172
21	6	0	-3.183290	-1.931286	0.174822
22	1	0	-3.599752	-1.754574	1.170834
23	1	0	-3.856699	-1.457405	-0.543459
24	1	0	-3.198169	-3.007739	-0.004472
25	8	0	-2.118852	3.549618	-0.171352
26	1	0	-2.184873	3.820153	-1.092708
27	1	0	-1.379610	2.864871	-0.142401

Rotational constants (GHZ): 0.9341078 0.6990864 0.4295606

- 1H_2O/wB97XD 6-311G+dp/PCM

H_2O
Electronic Energy -152.888235
Stoichiometry H4O2
Framework group C1[X(H4O2)]
Deg. of freedom 12
Full point group C1 NOp 1
Largest Abelian subgroup C1 NOp 1
Largest concise Abelian subgroup C1 NOp 1
Standard orientation:

Center Number	Atomic Number	Atomic Type	Coordinates (Angstroms) X	Y	Z
1	8	0	1.334098	-0.000066	0.089003

2	1	0	1.802929	0.766671	-0.260945
3	1	0	1.805153	-0.765474	-0.260862
4	8	0	-1.492632	-0.000059	-0.117740
5	1	0	-1.818990	0.000437	0.788019
6	1	0	-0.520820	-0.000636	-0.036318

Rotational constants (GHZ): 227.6237525 6.7080289 6.6950899

OH^-
Electronic Energy -152.389464
Stoichiometry H3O2(1-)
Framework group C1[X(H3O2)]
Deg. of freedom 9
Full point group C1 NOp 1
Largest Abelian subgroup C1 NOp 1
Largest concise Abelian subgroup C1 NOp 1
Standard orientation:

Center Number	Atomic Number	Atomic Type	Coordinates (Angstroms) X	Y	Z
1	8	0	1.260773	0.034490	-0.105350
2	1	0	1.669783	-0.137266	0.749182
3	8	0	-1.248911	-0.110104	-0.006106
4	1	0	-1.570442	0.780839	0.161116
5	1	0	-0.194238	-0.038664	-0.018653

Rotational constants (GHZ): 330.2884982 8.9463552 8.9345926

Phenol
Electronic Energy -383.904481
Stoichiometry C6H8O2
Framework group C1[X(C6H8O2)]
Deg. of freedom 42
Full point group C1 NOp 1
Largest Abelian subgroup C1 NOp 1
Largest concise Abelian subgroup C1 NOp 1
Standard orientation:

Center Number	Atomic Number	Atomic Type	Coordinates (Angstroms) X	Y	Z
1	6	0	-2.302075	-0.622886	0.000335
2	6	0	-1.082491	-1.283818	0.000134
3	6	0	0.104201	-0.551461	-0.000158
4	6	0	0.057285	0.843137	-0.000245
5	6	0	-1.171997	1.492388	-0.000043
6	6	0	-2.357823	0.768175	0.000248
7	1	0	-3.219466	-1.201619	0.000560
8	1	0	-1.027758	-2.366098	0.000197
9	1	0	0.982752	1.409687	-0.000472
10	1	0	-1.198347	2.576811	-0.000115
11	1	0	-3.313275	1.279279	0.000404
12	8	0	1.269651	-1.240321	-0.000345
13	1	0	2.021160	-0.629151	-0.000550
14	8	0	3.496479	0.498368	0.000044
15	1	0	4.071196	0.496314	0.767243
16	1	0	4.072101	0.497190	-0.766476

Rotational constants (GHZ): 4.2663683 1.1330328 0.8990482

Phenolate
Electronic Energy -383.426196
Stoichiometry C6H7O2(1-)
Framework group C1[X(C6H7O2)]
Deg. of freedom 39
Full point group C1 NOp 1
Largest Abelian subgroup C1 NOp 1
Largest concise Abelian subgroup C1 NOp 1

Standard orientation:

Center Number	Atomic Number	Atomic Type	Coordinates (Angstroms) X	Y	Z
1	6	0	2.190041	-0.716806	0.140365
2	6	0	0.939050	-1.302597	0.045778
3	6	0	-0.256182	-0.529409	-0.111429
4	6	0	-0.051857	0.888103	-0.168171
5	6	0	1.208874	1.454243	-0.072024
6	6	0	2.354149	0.670076	0.085797
7	1	0	3.064448	-1.354595	0.259901
8	1	0	0.828092	-2.382609	0.089775
9	1	0	-0.928408	1.515873	-0.304422
10	1	0	1.305414	2.537506	-0.123989
11	1	0	3.337963	1.122108	0.160080
12	8	0	-1.409840	-1.071090	-0.192209
13	8	0	-3.566780	0.455057	0.130711
14	1	0	-3.308115	0.845870	0.964963
15	1	0	-2.790883	-0.137553	-0.076220

Rotational constants (GHZ): 4.4720373 1.1643543 0.9337446

Thymol
Electronic Energy -541.161108
Stoichiometry C10H16O2
Framework group C1[X(C10H16O2)]
Deg. of freedom 78
Full point group C1 NOp 1
Largest Abelian subgroup C1 NOp 1
Largest concise Abelian subgroup C1 NOp 1
Standard orientation:

Center Number	Atomic Number	Atomic Type	Coordinates (Angstroms) X	Y	Z
1	6	0	1.514706	-0.149226	-0.007248
2	6	0	0.277910	0.484582	-0.100509
3	6	0	-0.915691	-0.258198	-0.120030
4	6	0	-0.802924	-1.641815	-0.035759
5	6	0	0.431762	-2.281292	0.062216
6	6	0	1.608466	-1.539067	0.075918
7	1	0	2.415568	0.457808	0.001212
8	1	0	-1.701744	-2.248566	-0.048442
9	1	0	0.473356	-3.363812	0.125802
10	8	0	0.181142	1.836575	-0.177215
11	1	0	1.066311	2.246778	-0.170027
12	6	0	-2.249713	0.461974	-0.185997
13	1	0	-2.106444	1.338829	-0.825139
14	6	0	-2.651469	0.969033	1.207040
15	1	0	-1.874008	1.606800	1.633753
16	1	0	-3.579320	1.546908	1.155940
17	1	0	-2.813698	0.124964	1.885602
18	6	0	-3.373097	-0.377775	-0.795335
19	1	0	-4.266489	0.238567	-0.927293
20	1	0	-3.090416	-0.780640	-1.771740
21	1	0	-3.649784	-1.215898	-0.148254
22	6	0	2.958771	-2.202487	0.165061
23	1	0	3.525334	-2.061568	-0.760252
24	1	0	3.553772	-1.777873	0.978143
25	1	0	2.860655	-3.275751	0.339065
26	8	0	2.650557	3.060399	-0.156795
27	1	0	2.827037	3.667526	0.566039
28	1	0	2.913953	3.525767	-0.954471

Rotational constants (GHZ): 0.9677892 0.6719645 0.4248944

Thymolate
Electronic Energy -540.680365
Stoichiometry C10H15O2(1-)
Framework group C1[X(C10H15O2)]

Deg. of freedom 75
Full point group C1 NOp 1
Largest Abelian subgroup C1 NOp 1
Largest concise Abelian subgroup C1 NOp 1
Standard orientation:

Center Number	Atomic Number	Atomic Type	Coordinates (Angstroms) X	Y	Z
1	6	0	-2.230057	0.237290	-0.194177
2	1	0	-2.149248	1.136728	-0.816002
3	6	0	-0.831493	-0.337791	-0.112221
4	6	0	-0.562082	-1.695453	-0.142583
5	6	0	0.250801	0.603149	0.035073
6	6	0	0.735453	-2.207352	-0.037395
7	1	0	-1.384165	-2.399318	-0.253374
8	6	0	1.558401	0.045685	0.137879
9	6	0	1.803354	-1.320867	0.103021
10	1	0	0.907268	-3.280024	-0.063958
11	1	0	2.383398	0.743234	0.266420
12	6	0	-2.703105	0.705170	1.189694
13	1	0	-3.681481	1.199271	1.129123
14	1	0	-2.791608	-0.149965	1.870637
15	1	0	-1.980871	1.412021	1.602333
16	6	0	-3.262467	-0.697059	-0.825498
17	1	0	-4.218318	-0.176511	-0.953535
18	1	0	-2.932241	-1.054122	-1.805937
19	1	0	-3.452411	-1.574072	-0.195464
20	6	0	3.221302	-1.837449	0.183374
21	1	0	3.704633	-1.818824	-0.800604
22	1	0	3.828384	-1.224956	0.856703
23	1	0	3.248119	-2.870413	0.544555
24	8	0	0.031672	1.864921	0.070333
25	8	0	2.082391	3.516736	-0.341467
26	1	0	2.423189	3.089918	-1.127183
27	1	0	1.302217	2.941846	-0.107645

Rotational constants (GHZ): 0.9588905 0.6972141 0.4343389

- 1H_2O/wB97XD 6-311G+dp/CPCM

H_2O
Electronic Energy -152.887822
Stoichiometry H4O2
Framework group CS[SG(H4O2)]
Deg. of freedom 9
Full point group CS NOp 2
Largest Abelian subgroup CS NOp 2
Largest concise Abelian subgroup C1 NOp 1
Standard orientation:

Center Number	Atomic Number	Atomic Type	Coordinates (Angstroms) X	Y	Z
1	8	0	0.045614	-1.308741	0.000000
2	1	0	0.836597	-1.857058	0.000000
3	1	0	-0.701851	-1.915030	0.000000
4	8	0	0.045614	1.491933	0.000000
5	1	0	0.004654	0.519625	0.000000
6	1	0	-0.869217	1.786931	0.000000

Rotational constants (GHZ): 255.7078622 6.8627524 6.6833821

OH^-
Electronic Energy -152.389566
Stoichiometry H3O2(1-)
Framework group C1[X(H3O2)]
Deg. of freedom 9
Full point group C1 NOp 1
Largest Abelian subgroup C1 NOp 1

Largest concise Abelian subgroup C1 NOp 1
Standard orientation:

Center Number	Atomic Number	Atomic Type	Coordinates (Angstroms) X	Y	Z
1	8	0	-1.240695	-0.108949	0.014236
2	1	0	-0.186191	-0.042084	-0.010759
3	1	0	-1.567950	0.793657	0.018536
4	8	0	1.250371	0.013500	-0.109165
5	1	0	1.676734	0.012019	0.751656

Rotational constants (GHZ): 335.5191073 9.0657237 9.0541656

Phenol
Electronic Energy -383.904553
Stoichiometry C6H8O2
Framework group C1[X(C6H8O2)]
Deg. of freedom 42
Full point group C1 NOp 1
Largest Abelian subgroup C1 NOp 1
Largest concise Abelian subgroup C1 NOp 1
Standard orientation:

Center Number	Atomic Number	Atomic Type	Coordinates (Angstroms) X	Y	Z
1	6	0	0.048190	0.857740	-0.006678
2	6	0	0.118916	-0.534356	-0.002806
3	6	0	-1.053405	-1.287215	0.002851
4	6	0	-2.284984	-0.649278	0.005110
5	6	0	-2.364579	0.739516	0.001733
6	6	0	-1.191766	1.484256	-0.004279
7	1	0	0.962674	1.439096	-0.011980
8	1	0	-3.190934	-1.244205	0.009705
9	1	0	-3.328117	1.233654	0.003675
10	8	0	1.300591	-1.201483	-0.004606
11	1	0	2.058774	-0.581514	-0.004040
12	8	0	3.451997	0.488121	-0.000264
13	1	0	4.077665	0.444823	-0.731698
14	1	0	3.985826	0.552266	0.799253
15	1	0	-1.236963	2.567188	-0.007181
16	1	0	-0.983851	-2.368386	0.005640

Rotational constants (GHZ): 4.3366538 1.1458627 0.9102438

Phenolate
Electronic Energy -383.426346
Stoichiometry C6H7O2(1-)
Framework group C1[X(C6H7O2)]
Deg. of freedom 39
Full point group C1 NOp 1
Largest Abelian subgroup C1 NOp 1
Largest concise Abelian subgroup C1 NOp 1
Standard orientation:

Center Number	Atomic Number	Atomic Type	Coordinates (Angstroms) X	Y	Z
1	6	0	-0.004843	0.922468	0.021875
2	6	0	-0.239972	-0.478356	0.024192
3	6	0	0.917886	-1.299790	0.002271
4	6	0	2.194967	-0.762206	-0.018937
5	6	0	2.397916	0.616859	-0.020528
6	6	0	1.278616	1.446366	0.000255
7	1	0	-0.863120	1.586523	0.037821
8	1	0	3.050092	-1.431159	-0.034970
9	1	0	3.398221	1.032200	-0.037043
10	8	0	-1.435759	-0.985532	0.045664
11	8	0	-3.639269	0.376019	-0.122028
12	1	0	-3.680028	0.974916	0.627792
13	1	0	-2.757968	-0.106737	-0.040239

14	1	0	1.408922	2.524355	-0.000246
15	1	0	0.776682	-2.376039	0.003026

Rotational constants (GHZ): 4.7222127 1.1269445 0.9120381

Thymol

Electronic Energy -541.161186

Stoichiometry C10H16O2

Framework group C1[X(C10H16O2)]

Deg. of freedom 78

Full point group C1 NOp 1

Largest Abelian subgroup C1 NOp 1

Largest concise Abelian subgroup C1 NOp 1

Standard orientation:

Center Number	Atomic Number	Atomic Type	Coordinates (Angstroms) X	Y	Z
1	6	0	1.516139	-0.153903	-0.004064
2	6	0	0.279843	0.475766	-0.095003
3	6	0	-0.911743	-0.266261	-0.114725
4	6	0	-0.795832	-1.648069	-0.035164
5	6	0	0.439025	-2.284437	0.060893
6	6	0	1.613289	-1.541996	0.075957
7	1	0	2.413616	0.456249	0.004551
8	1	0	-1.692241	-2.256102	-0.050591
9	1	0	0.482875	-3.366063	0.121393
10	8	0	0.182496	1.830779	-0.169826
11	1	0	1.066677	2.249257	-0.167270
12	6	0	-2.245166	0.453328	-0.186535
13	1	0	-2.098398	1.324411	-0.830816
14	6	0	-2.653670	0.974256	1.198134
15	1	0	-1.877933	1.612036	1.624642
16	1	0	-3.577131	1.556125	1.135330
17	1	0	-2.826201	0.140285	1.884547
18	6	0	-3.366956	-0.388129	-0.793308
19	1	0	-4.262807	0.224057	-0.919515
20	1	0	-3.089027	-0.785971	-1.771917
21	1	0	-3.637566	-1.229421	-0.150184
22	6	0	2.962499	-2.205039	0.164483
23	1	0	3.536398	-2.053584	-0.753524
24	1	0	3.553017	-1.793104	0.986093
25	1	0	2.863443	-3.279393	0.323761
26	8	0	2.618670	3.100304	-0.162901
27	1	0	2.810663	3.702465	0.564266
28	1	0	2.900721	3.556999	-0.962962

Rotational constants (GHZ): 0.9594052 0.6751345 0.4242316

Thymolate

Electronic Energy -540.680535

Stoichiometry C10H15O2(1-)

Framework group C1[X(C10H15O2)]

Deg. of freedom 75

Full point group C1 NOp 1

Largest Abelian subgroup C1 NOp 1

Largest concise Abelian subgroup C1 NOp 1

Standard orientation:

Center Number	Atomic Number	Atomic Type	Coordinates (Angstroms) X	Y	Z
1	6	0	-1.579970	0.004228	-0.012171
2	6	0	-0.288640	0.580797	-0.084031
3	6	0	0.825182	-0.318299	-0.101460
4	6	0	0.588007	-1.682624	-0.042080
5	6	0	-0.697519	-2.221913	0.035633
6	6	0	-1.797087	-1.367978	0.053318
7	1	0	-2.430673	0.680543	-0.006626
8	1	0	1.431083	-2.366052	-0.061198
9	1	0	-0.837125	-3.296833	0.076744
10	8	0	-0.118411	1.870167	-0.138456

11	6	0	2.223536	0.263684	-0.155490
12	1	0	2.137864	1.206336	-0.702541
13	6	0	3.240522	-0.615678	-0.883836
14	1	0	2.898163	-0.878594	-1.887709
15	1	0	4.194399	-0.090039	-0.979190
16	1	0	3.437343	-1.545390	-0.342952
17	6	0	2.730447	0.607036	1.251744
18	1	0	3.700786	1.111374	1.209722
19	1	0	2.028860	1.262583	1.770585
20	1	0	2.850129	-0.302587	1.848473
21	6	0	-3.199524	-1.912865	0.164646
22	1	0	-3.579561	-1.818828	1.186442
23	1	0	-3.890854	-1.373302	-0.486583
24	1	0	-3.236265	-2.970594	-0.102498
25	8	0	-2.010814	3.633496	-0.103881
26	1	0	-2.627788	3.430892	-0.811622
27	1	0	-1.312293	2.902864	-0.130000

- 1H_2O/CAM-B3LYP 6-311G+dp/SMD

H_2O
Electronic Energy -152.948642
Stoichiometry H4O2
Framework group C1[X(H4O2)]
Deg. of freedom 12
Full point group C1 NOp 1
Largest Abelian subgroup C1 NOp 1
Largest concise Abelian subgroup C1 NOp 1
Standard orientation:

Center Number	Atomic Number	Atomic Type	Coordinates (Angstroms) X	Y	Z
1	8	0	1.354996	-0.000069	0.095350
2	1	0	1.785425	0.769030	-0.288545
3	1	0	1.787533	-0.767656	-0.289197
4	8	0	-1.498925	0.000064	-0.119702
5	1	0	-1.882099	0.000036	0.759773
6	1	0	-0.539428	-0.001371	0.012785

Rotational constants (GHZ): 226.2594154 6.5900974 6.5775524

OH^-
Electronic Energy -152.462455
Stoichiometry H3O2(1-)
Framework group C1[X(H3O2)]
Deg. of freedom 9
Full point group C1 NOp 1
Largest Abelian subgroup C1 NOp 1
Largest concise Abelian subgroup C1 NOp 1
Standard orientation:

Center Number	Atomic Number	Atomic Type	Coordinates (Angstroms) X	Y	Z
1	8	0	1.286943	0.084111	-0.074999
2	1	0	1.649047	-0.511239	0.590619
3	8	0	-1.265979	-0.096278	-0.055426
4	1	0	-1.583273	0.634103	0.487062
5	1	0	-0.233481	-0.025523	-0.034279

Rotational constants (GHZ): 321.6601960 8.6825052 8.6569125

Phenol
Electronic Energy -383.856631
Stoichiometry C6H8O2
Framework group C1[X(C6H8O2)]
Deg. of freedom 42

Full point group C1 NOp 1
Largest Abelian subgroup C1 NOp 1
Largest concise Abelian subgroup C1 NOp 1
Standard orientation:

Center Number	Atomic Number	Atomic Type	Coordinates (Angstroms) X	Y	Z
1	6	0	0.000762	0.891143	0.001746
2	6	0	0.117993	-0.496199	0.005679
3	6	0	-1.023895	-1.292530	0.003289
4	6	0	-2.277735	-0.698911	-0.002436
5	6	0	-2.405945	0.686156	-0.006136
6	6	0	-1.260849	1.473063	-0.004055
7	1	0	0.892903	1.506109	0.003079
8	1	0	-3.161897	-1.325828	-0.004248
9	1	0	-3.386658	1.145616	-0.010633
10	8	0	1.331806	-1.125248	0.012348
11	1	0	2.075401	-0.480402	0.005527
12	8	0	3.518483	0.492134	-0.033283
13	1	0	4.175614	0.124055	-0.636968
14	1	0	3.961400	0.536118	0.823222
15	1	0	-1.344687	2.553692	-0.006907
16	1	0	-0.916373	-2.370780	0.005891

Rotational constants (GHZ): 4.4550873 1.1135613 0.8943871

Phenolate
Electronic Energy -383.386583
Stoichiometry C6H7O2(1-)
Framework group C1[X(C6H7O2)]
Deg. of freedom 39
Full point group C1 NOp 1
Largest Abelian subgroup C1 NOp 1
Largest concise Abelian subgroup C1 NOp 1
Standard orientation:

Center Number	Atomic Number	Atomic Type	Coordinates (Angstroms) X	Y	Z
1	6	0	-0.013052	0.914708	-0.040474
2	6	0	-0.228561	-0.483276	-0.030988
3	6	0	0.925250	-1.299963	0.008502
4	6	0	2.199333	-0.753455	0.037758
5	6	0	2.389484	0.626336	0.028802
6	6	0	1.266612	1.449092	-0.010624
7	1	0	-0.876279	1.570981	-0.072233
8	1	0	3.058722	-1.415614	0.068087
9	1	0	3.386592	1.048991	0.052078
10	8	0	-1.435476	-1.004689	-0.058785
11	8	0	-3.595381	0.462367	-0.041306
12	1	0	-3.996254	0.331757	0.823968
13	1	0	-2.741546	-0.068583	-0.026226
14	1	0	1.389348	2.527439	-0.018933
15	1	0	0.791876	-2.377050	0.016133

Rotational constants (GHZ): 4.6770234 1.1349725 0.9160416

Thymol
Electronic Energy -541.055716
Stoichiometry C10H16O2
Framework group C1[X(C10H16O2)]
Deg. of freedom 78
Full point group C1 NOp 1
Largest Abelian subgroup C1 NOp 1
Largest concise Abelian subgroup C1 NOp 1
Standard orientation:

Center Number	Atomic Number	Atomic Type	Coordinates (Angstroms) X	Y	Z
1	6	0	1.529590	-0.172414	0.004903

2	6	0	0.296197	0.461624	-0.083415
3	6	0	-0.899135	-0.272445	-0.113123
4	6	0	-0.789493	-1.656185	-0.040685
5	6	0	0.442036	-2.298182	0.054712
6	6	0	1.620468	-1.561330	0.075766
7	1	0	2.430896	0.431300	0.020466
8	1	0	-1.688897	-2.259727	-0.060229
9	1	0	0.482024	-3.380314	0.110309
10	8	0	0.208465	1.826618	-0.145735
11	1	0	1.097135	2.245634	-0.140078
12	6	0	-2.232878	0.447369	-0.183926
13	1	0	-2.086837	1.329643	-0.812635
14	6	0	-2.657137	0.937784	1.206034
15	1	0	-1.891769	1.569691	1.660835
16	1	0	-3.581944	1.517777	1.144179
17	1	0	-2.835318	0.088669	1.872278
18	6	0	-3.346561	-0.385913	-0.814273
19	1	0	-4.241974	0.228107	-0.936848
20	1	0	-3.060473	-0.764596	-1.798466
21	1	0	-3.620073	-1.239297	-0.188710
22	6	0	2.965153	-2.230897	0.156717
23	1	0	3.510617	-2.129723	-0.785686
24	1	0	3.584096	-1.783367	0.937693
25	1	0	2.861754	-3.295610	0.370414
26	8	0	2.570413	3.205243	-0.164455
27	1	0	2.571173	3.893423	0.512434
28	1	0	2.669118	3.677038	-1.000703

Rotational constants (GHZ): 0.9316196 0.6818526 0.4217409

Thymolate

Electronic Energy -540.583746
Stoichiometry C10H15O2(1-)
Framework group C1[X(C10H15O2)]
Deg. of freedom 75
Full point group C1 NOp 1
Largest Abelian subgroup C1 NOp 1
Largest concise Abelian subgroup C1 NOp 1
Standard orientation:

Center Number	Atomic Number	Atomic Type	Coordinates (Angstroms) X	Y	Z
1	6	0	-1.572919	-0.004228	0.042850
2	6	0	-0.287717	0.570611	-0.037248
3	6	0	0.830926	-0.312396	-0.109413
4	6	0	0.599679	-1.680433	-0.090243
5	6	0	-0.682281	-2.224992	-0.003555
6	6	0	-1.785515	-1.379691	0.064679
7	1	0	-2.424547	0.668428	0.091218
8	1	0	1.443299	-2.359699	-0.148756
9	1	0	-0.817552	-3.301061	0.005321
10	8	0	-0.128784	1.878335	-0.049286
11	6	0	2.228170	0.274503	-0.173860
12	1	0	2.158785	1.188161	-0.771518
13	6	0	3.258603	-0.634510	-0.841338
14	1	0	2.935086	-0.950849	-1.836226
15	1	0	4.208476	-0.104489	-0.949590
16	1	0	3.454082	-1.532657	-0.249400
17	6	0	2.714266	0.682208	1.223088
18	1	0	3.683737	1.186229	1.168228
19	1	0	2.009394	1.359018	1.710160
20	1	0	2.829898	-0.200730	1.859172
21	6	0	-3.183290	-1.931286	0.174822
22	1	0	-3.599752	-1.754574	1.170834
23	1	0	-3.856699	-1.457405	-0.543459
24	1	0	-3.198169	-3.007739	-0.004472
25	8	0	-2.118852	3.549618	-0.171352
26	1	0	-2.184873	3.820153	-1.092708
27	1	0	-1.379610	2.864871	-0.142401

Rotational constants (GHZ): 0.9341078 0.6990864 0.4295606

Carvacrol

Electronic Energy -541.055871
Stoichiometry C10H16O2
Framework group C1[X(C10H16O2)]
Deg. of freedom 78
Full point group C1 NOp 1
Largest Abelian subgroup C1 NOp 1
Largest concise Abelian subgroup C1 NOp 1

Standard orientation:

Center Number	Atomic Number	Atomic Type	Coordinates (Angstroms) X	Y	Z
1	6	0	1.213603	0.951319	-0.002658
2	6	0	0.990774	-0.427489	-0.022962
3	6	0	-0.302571	-0.943528	-0.024318
4	6	0	-1.414906	-0.111772	-0.004329
5	6	0	-1.202776	1.267136	0.017793
6	6	0	0.087949	1.774411	0.017889
7	1	0	-2.045014	1.949058	0.034082
8	8	0	2.007014	-1.349413	-0.043286
9	1	0	2.908509	-0.966997	-0.038436
10	8	0	4.723957	-0.954610	-0.064033
11	1	0	5.101324	-1.658499	0.477464
12	1	0	5.070672	-0.135995	0.310954
13	1	0	-0.426307	-2.021160	-0.041831
14	1	0	0.238710	2.848827	0.033734
15	6	0	2.599241	1.533289	-0.002870
16	1	0	3.166399	1.227088	0.880713
17	1	0	3.170655	1.216668	-0.879748
18	1	0	2.551207	2.622301	-0.009005
19	6	0	-2.812878	-0.699079	-0.005497
20	1	0	-2.702050	-1.786794	-0.036680
21	6	0	-3.604519	-0.272305	-1.244095
22	1	0	-3.082944	-0.547752	-2.163839
23	1	0	-4.586038	-0.753376	-1.254044
24	1	0	-3.762519	0.809399	-1.256004
25	6	0	-3.575410	-0.341704	1.272838
26	1	0	-4.557914	-0.820879	1.277980
27	1	0	-3.034036	-0.669359	2.163563
28	1	0	-3.729461	0.737991	1.348908

Rotational constants (GHZ): 1.9958574 0.4131278 0.3704264

Carvacrolate

Electronic Energy -540.585297
Stoichiometry C10H15O2(1-)
Framework group C1[X(C10H15O2)]
Deg. of freedom 75
Full point group C1 NOp 1
Largest Abelian subgroup C1 NOp 1
Largest concise Abelian subgroup C1 NOp 1

Standard orientation:

Center Number	Atomic Number	Atomic Type	Coordinates (Angstroms) X	Y	Z
1	6	0	-1.299715	1.094494	-0.029167
2	6	0	-1.142101	-0.232042	-0.507320
3	6	0	0.160418	-0.775560	-0.509258
4	6	0	1.275011	-0.071171	-0.067159
5	6	0	1.096914	1.231727	0.400358
6	6	0	-0.178747	1.788982	0.410194
7	1	0	1.941945	1.811842	0.753373
8	8	0	-2.172683	-0.933952	-0.941275
9	8	0	-4.113905	-1.586468	0.694138
10	1	0	-3.944694	-2.507541	0.916875
11	1	0	-3.361765	-1.316859	0.089777
12	1	0	-0.308929	2.805050	0.772541
13	1	0	0.280563	-1.790863	-0.877568
14	6	0	-2.663611	1.725374	-0.017027
15	1	0	-3.108075	1.740651	-1.017047

16	1	0	-3.359733	1.176980	0.624201
17	1	0	-2.612005	2.753860	0.345226
18	6	0	2.648140	-0.718647	-0.093445
19	1	0	2.521685	-1.717710	-0.521395
20	6	0	3.626184	0.050251	-0.985405
21	1	0	3.811520	1.053405	-0.592275
22	1	0	4.586687	-0.469537	-1.039022
23	1	0	3.240248	0.153001	-2.002591
24	6	0	3.224249	-0.884811	1.315340
25	1	0	2.550765	-1.460849	1.954676
26	1	0	4.184583	-1.406496	1.279026
27	1	0	3.389464	0.086845	1.788629

Rotational constants (GHZ): 1.6257901 0.4597504 0.4064813

2,3-dimethylphenol
Electronic Energy -462.456624
Stoichiometry C8H12O2
Framework group C1[X(C8H12O2)]
Deg. of freedom 60
Full point group C1 NOp 1
Largest Abelian subgroup C1 NOp 1
Largest concise Abelian subgroup C1 NOp 1
Standard orientation:

Center Number	Atomic Number	Atomic Type	Coordinates (Angstroms) X	Y	Z
1	6	0	-0.134344	0.442886	0.037416
2	6	0	0.290758	-0.890372	0.039445
3	6	0	-0.618009	-1.942842	0.008611
4	6	0	-1.974519	-1.672870	-0.032874
5	6	0	-2.418779	-0.357742	-0.045642
6	6	0	-1.513855	0.702054	-0.011623
7	1	0	-3.480942	-0.144832	-0.082076
8	8	0	1.615765	-1.246085	0.082822
9	1	0	2.247037	-0.502723	0.004377
10	8	0	3.871202	0.310911	-0.191701
11	1	0	4.590945	-0.208492	0.186873
12	1	0	3.884841	1.146876	0.289708
13	1	0	-2.686640	-2.489508	-0.058200
14	1	0	-0.244227	-2.959776	0.014577
15	6	0	0.883863	1.549902	0.084309
16	1	0	1.577564	1.413734	0.918233
17	1	0	1.481936	1.580259	-0.831240
18	1	0	0.415997	2.523868	0.203589
19	6	0	-2.039399	2.113999	-0.026842
20	1	0	-1.753945	2.660657	0.875583
21	1	0	-1.654585	2.678573	-0.879677
22	1	0	-3.128003	2.112660	-0.087514

Rotational constants (GHZ): 1.9644230 0.8760485 0.6119151

2,3-dimethylphenolate
Electronic Energy -461.986350
Stoichiometry C8H11O2(1-)
Framework group C1[X(C8H11O2)]
Deg. of freedom 57
Full point group C1 NOp 1
Largest Abelian subgroup C1 NOp 1
Largest concise Abelian subgroup C1 NOp 1
Standard orientation:

Center Number	Atomic Number	Atomic Type	Coordinates (Angstroms) X	Y	Z
1	6	0	-0.153722	0.494229	0.255295
2	6	0	0.531760	-0.748354	0.352242
3	6	0	-0.206619	-1.928987	0.123132
4	6	0	-1.549573	-1.885259	-0.210201
5	6	0	-2.204616	-0.664995	-0.326227
6	6	0	-1.509406	0.524463	-0.093393

7	1	0	-2.090639	-2.809374	-0.386654
8	1	0	-3.253986	-0.628412	-0.596655
9	8	0	1.816852	-0.810967	0.646276
10	8	0	3.592872	0.473193	-0.778766
11	1	0	4.067304	-0.221304	-1.246852
12	1	0	2.889414	-0.000681	-0.242743
13	1	0	0.311218	-2.879116	0.206563
14	6	0	0.605510	1.767254	0.519937
15	1	0	1.429463	1.589584	1.211534
16	1	0	1.041305	2.182309	-0.395891
17	1	0	-0.035574	2.539069	0.947854
18	6	0	-2.237977	1.837727	-0.228585
19	1	0	-2.278709	2.377439	0.721871
20	1	0	-1.746686	2.499457	-0.947020
21	1	0	-3.263055	1.676742	-0.565286

Rotational constants (GHZ): 1.8457943 0.9429440 0.6759255

2,4-dimethylphenol
Electronic Energy -462.457765
Stoichiometry C8H12O2
Framework group C1[X(C8H12O2)]
Deg. of freedom 60
Full point group C1 NOp 1
Largest Abelian subgroup C1 NOp 1
Largest concise Abelian subgroup C1 NOp 1
Standard orientation:

Center Number	Atomic Number	Atomic Type	Coordinates (Angstroms) X	Y	Z
1	6	0	-0.163074	0.656721	0.014495
2	6	0	-0.444875	-0.713331	0.018379
3	6	0	0.588368	-1.641541	0.000375
4	6	0	1.910637	-1.221916	-0.019421
5	6	0	2.229077	0.133165	-0.019997
6	6	0	1.175277	1.044062	-0.005774
7	1	0	2.703554	-1.961905	-0.036691
8	8	0	-1.723171	-1.216098	0.034893
9	1	0	-2.429147	-0.537552	0.033976
10	8	0	-4.120464	0.122308	0.055288
11	1	0	-4.736282	-0.449273	-0.419165
12	1	0	-4.164437	0.971763	-0.399951
13	1	0	1.398019	2.106831	-0.011924
14	6	0	3.659012	0.603154	-0.009272
15	1	0	3.999333	0.809260	1.010066
16	1	0	4.325692	-0.151444	-0.430269
17	1	0	3.778738	1.523833	-0.583704
18	1	0	0.342386	-2.697207	-0.001662
19	6	0	-1.259362	1.684560	0.026279
20	1	0	-1.897339	1.602673	-0.858177
21	1	0	-1.904484	1.574773	0.902072
22	1	0	-0.837312	2.689326	0.043610

Rotational constants (GHZ): 2.9216934 0.6810770 0.5566300

2,4-dimethylphenolate
Electronic Energy -461.986867
Stoichiometry C8H11O2(1-)
Framework group C1[X(C8H11O2)]
Deg. of freedom 57
Full point group C1 NOp 1
Largest Abelian subgroup C1 NOp 1
Largest concise Abelian subgroup C1 NOp 1
Standard orientation:

Center Number	Atomic Number	Atomic Type	Coordinates (Angstroms) X	Y	Z
1	6	0	-0.158734	0.806766	-0.258786
2	6	0	-0.640291	-0.517902	-0.442170
3	6	0	0.293817	-1.560989	-0.313398

4	6	0	1.630681	-1.318562	-0.014831
5	6	0	2.105290	-0.024869	0.170802
6	6	0	1.181657	1.016351	0.039125
7	1	0	2.315254	-2.157089	0.074355
8	8	0	-1.909447	-0.755484	-0.727266
9	8	0	-3.774568	-0.166641	1.011770
10	1	0	-4.066584	-1.014996	1.360522
11	1	0	-3.042648	-0.382399	0.361075
12	1	0	1.524492	2.039717	0.172895
13	1	0	-0.054322	-2.579563	-0.455164
14	6	0	-1.109575	1.961766	-0.400452
15	1	0	-1.583904	1.970036	-1.386506
16	1	0	-1.918936	1.911106	0.333406
17	1	0	-0.590388	2.912359	-0.265622
18	6	0	3.547233	0.253428	0.508415
19	1	0	3.990229	0.978617	-0.179795
20	1	0	3.654832	0.662940	1.517523
21	1	0	4.143646	-0.659662	0.459046

Rotational constants (GHZ): 2.4512130 0.7340184 0.6224800

2,6-diisopropylphenol

Electronic Energy -619.648509

Stoichiometry C12H20O2

Framework group C1[X(C12H20O2)]

Deg. of freedom 96

Full point group C1 NOp 1

Largest Abelian subgroup C1 NOp 1

Largest concise Abelian subgroup C1 NOp 1

Standard orientation:

Center Number	Atomic Number	Atomic Type	Coordinates (Angstroms) X	Y	Z
1	6	0	-0.899862	-0.812955	-0.073847
2	6	0	0.153835	0.107758	-0.143374
3	6	0	1.496011	-0.297394	-0.116360
4	6	0	1.765845	-1.654990	-0.007029
5	6	0	0.741891	-2.587767	0.070422
6	6	0	-0.575220	-2.162828	0.034128
7	1	0	2.793572	-1.994883	0.010378
8	1	0	0.971018	-3.643378	0.151678
9	8	0	-0.055495	1.452860	-0.235334
10	1	0	-0.985977	1.701976	-0.331079
11	8	0	-2.378284	3.011495	-0.609971
12	1	0	-2.004209	3.779438	-1.052026
13	1	0	-2.895961	3.351551	0.125366
14	1	0	-1.367860	-2.898218	0.085107
15	6	0	2.587401	0.755148	-0.180622
16	1	0	2.237212	1.526698	-0.871265
17	6	0	3.921025	0.227908	-0.707436
18	1	0	3.804672	-0.276293	-1.669272
19	1	0	4.620392	1.056273	-0.842776
20	1	0	4.385738	-0.474576	-0.010566
21	6	0	2.775659	1.422306	1.188433
22	1	0	3.518294	2.222921	1.129493
23	1	0	1.839202	1.851741	1.545538
24	1	0	3.122907	0.693082	1.925844
25	6	0	-2.341075	-0.331239	-0.079426
26	1	0	-2.416115	0.503261	-0.784061
27	6	0	-3.343280	-1.381053	-0.558545
28	1	0	-4.333853	-0.931140	-0.656928
29	1	0	-3.060339	-1.792477	-1.529320
30	1	0	-3.432919	-2.209119	0.148628
31	6	0	-2.745058	0.192564	1.306984
32	1	0	-2.059605	0.963058	1.666170
33	1	0	-3.755221	0.611660	1.285070
34	1	0	-2.733738	-0.621160	2.036489

Rotational constants (GHZ): 0.8838550 0.4654856 0.3375062

2,6-diisopropylphenolate
Electronic Energy -619.177001
Stoichiometry C12H19O2(1-)
Framework group C1[X(C12H19O2)]
Deg. of freedom 93
Full point group C1 NOp 1
Largest Abelian subgroup C1 NOp 1
Largest concise Abelian subgroup C1 NOp 1
Standard orientation:

Center Number	Atomic Number	Atomic Type	Coordinates (Angstroms) X	Y	Z
1	6	0	-1.011160	-0.747684	-0.069974
2	6	0	0.089260	0.170344	-0.075883
3	6	0	1.411178	-0.383147	-0.114805
4	6	0	1.593288	-1.756813	-0.133374
5	6	0	0.515861	-2.638253	-0.118781
6	6	0	-0.773471	-2.113513	-0.088177
7	1	0	2.600018	-2.162923	-0.166896
8	1	0	0.676763	-3.710645	-0.138481
9	8	0	-0.081580	1.445275	-0.043327
10	8	0	-1.761904	3.311081	-0.847051
11	1	0	-1.175995	3.740767	-1.472698
12	1	0	-1.205515	2.560977	-0.496586
13	1	0	-1.614405	-2.800394	-0.084603
14	6	0	-2.406345	-0.157672	-0.010415
15	1	0	-2.408337	0.711842	-0.673831
16	6	0	-3.512317	-1.101645	-0.480826
17	1	0	-4.469651	-0.572468	-0.511230
18	1	0	-3.307902	-1.492340	-1.481184
19	1	0	-3.633482	-1.955351	0.193902
20	6	0	-2.717503	0.364899	1.398715
21	1	0	-1.946433	1.068099	1.713913
22	1	0	-3.683429	0.881095	1.423217
23	1	0	-2.750874	-0.463781	2.114572
24	6	0	2.560104	0.603899	-0.114441
25	1	0	2.244771	1.428402	-0.762462
26	6	0	2.762572	1.205509	1.282340
27	1	0	3.532344	1.986460	1.270168
28	1	0	1.827545	1.646172	1.628512
29	1	0	3.073850	0.431567	1.992224
30	6	0	3.876291	0.043785	-0.651621
31	1	0	3.750622	-0.392173	-1.646219
32	1	0	4.626804	0.837920	-0.719506
33	1	0	4.284623	-0.732322	0.003668

Rotational constants (GHZ): 0.8609665 0.4825214 0.3477597

2-methyl-4-*terz*-butylphenol
Electronic Energy -580.353998
Stoichiometry C11H18O2
Framework group C1[X(C11H18O2)]
Deg. of freedom 87
Full point group C1 NOp 1
Largest Abelian subgroup C1 NOp 1
Largest concise Abelian subgroup C1 NOp 1
Standard orientation:

Center Number	Atomic Number	Atomic Type	Coordinates (Angstroms) X	Y	Z
1	6	0	-0.514186	1.126158	-0.004520
2	6	0	0.853310	1.366304	-0.003067
3	6	0	1.715398	0.263942	0.004945
4	6	0	1.200937	-1.022700	0.011326
5	6	0	-0.176336	-1.228400	0.010054
6	6	0	-1.066441	-0.159894	0.002046
7	1	0	-1.172055	1.988415	-0.011032
8	1	0	1.877581	-1.869621	0.018108
9	1	0	-0.538321	-2.247795	0.015469
10	8	0	3.063624	0.513586	0.006087

11	1	0	3.582127	-0.321747	0.012762
12	8	0	4.662098	-1.695962	0.041494
13	1	0	4.846224	-2.040188	-0.841208
14	1	0	5.527199	-1.493220	0.418398
15	6	0	-2.588530	-0.336758	0.000089
16	6	0	-2.997660	-1.811920	0.011550
17	1	0	-4.087817	-1.885455	0.010314
18	1	0	-2.627643	-2.343234	-0.868631
19	1	0	-2.630556	-2.328592	0.901622
20	6	0	-3.181687	0.316438	-1.258565
21	1	0	-4.268659	0.196531	-1.268688
22	1	0	-2.962690	1.385391	-1.299774
23	1	0	-2.781440	-0.146947	-2.164244
24	6	0	-3.187680	0.337367	1.244789
25	1	0	-2.791399	-0.110450	2.159987
26	1	0	-2.969478	1.406980	1.269035
27	1	0	-4.274624	0.217032	1.251917
28	6	0	1.408589	2.761634	-0.010122
29	1	0	2.031473	2.947237	0.868981
30	1	0	2.037699	2.935868	-0.887097
31	1	0	0.602319	3.495778	-0.017700

Rotational constants (GHZ): 1.3312348 0.3976055 0.3291344

2-methyl-4-*terz*-butylphenolate
Electronic Energy -579.881634
Stoichiometry C11H17O2(1-)
Framework group C1[X(C11H17O2)]
Deg. of freedom 84
Full point group C1 NOp 1
Largest Abelian subgroup C1 NOp 1
Largest concise Abelian subgroup C1 NOp 1
Standard orientation:

Center Number	Atomic Number	Atomic Type	Coordinates (Angstroms) X	Y	Z
1	6	0	-0.438592	1.105346	0.020864
2	6	0	0.934079	1.306204	-0.006290
3	6	0	1.809676	0.188742	-0.071352
4	6	0	1.211232	-1.082563	-0.100087
5	6	0	-0.170691	-1.249875	-0.070228
6	6	0	-1.036457	-0.161795	-0.010204
7	1	0	-1.071236	1.986805	0.068365
8	1	0	1.855611	-1.954489	-0.149802
9	1	0	-0.561689	-2.259697	-0.096727
10	8	0	3.120056	0.352534	-0.105793
11	8	0	4.773529	-1.647254	0.033601
12	1	0	5.009536	-1.740592	0.962104
13	1	0	4.095720	-0.900460	-0.000309
14	6	0	-2.563142	-0.293855	0.018485
15	6	0	-3.016103	-1.756127	-0.016945
16	1	0	-4.107921	-1.800054	0.005660
17	1	0	-2.681943	-2.263186	-0.925421
18	1	0	-2.642873	-2.317935	0.842830
19	6	0	-3.170807	0.423352	-1.198201
20	1	0	-4.261531	0.339426	-1.184620
21	1	0	-2.917456	1.485621	-1.206476
22	1	0	-2.809166	-0.017474	-2.131153
23	6	0	-3.119209	0.346791	1.300680
24	1	0	-2.720402	-0.149900	2.189354
25	1	0	-2.864741	1.406930	1.363682
26	1	0	-4.209670	0.262457	1.326402
27	6	0	1.513727	2.692010	0.031523
28	1	0	2.170633	2.829101	0.895943
29	1	0	2.121857	2.897864	-0.854693
30	1	0	0.724309	3.443954	0.082928

Rotational constants (GHZ): 1.3875450 0.3980773 0.3329770

4-bromothymol
Electronic Energy -3114.701617
Stoichiometry C10H15BrO2
Framework group C1[X(C10H15BrO2)]
Deg. of freedom 78
Full point group C1 NOp 1
Largest Abelian subgroup C1 NOp 1
Largest concise Abelian subgroup C1 NOp 1
Standard orientation:

Center Number	Atomic Number	Atomic Type	Coordinates (Angstroms) X	Y	Z
1	6	0	0.739299	-1.528334	-0.017415
2	6	0	1.561410	-0.410320	-0.092043
3	6	0	1.018506	0.880397	-0.132585
4	6	0	-0.365984	0.988363	-0.091627
5	6	0	-1.175382	-0.135381	-0.012895
6	6	0	-0.649433	-1.422341	0.025653
7	1	0	1.191634	-2.513148	0.007213
8	1	0	-0.824119	1.967538	-0.125183
9	8	0	2.920385	-0.533519	-0.131778
10	1	0	3.202794	-1.476461	-0.121549
11	6	0	1.928575	2.092555	-0.181958
12	1	0	2.792068	1.815178	-0.791818
13	6	0	2.443637	2.433148	1.221950
14	1	0	2.943279	1.580252	1.685346
15	1	0	3.156389	3.260847	1.178259
16	1	0	1.615103	2.734258	1.869474
17	6	0	1.284267	3.318774	-0.823977
18	1	0	2.027946	4.112052	-0.929025
19	1	0	0.888109	3.094350	-1.817179
20	1	0	0.468881	3.715816	-0.213994
21	6	0	-1.497842	-2.657712	0.106664
22	1	0	-2.174712	-2.732053	-0.747625
23	1	0	-0.870923	-3.549027	0.123523
24	1	0	-2.115927	-2.657141	1.007499
25	8	0	3.905632	-3.066158	-0.115447
26	1	0	4.577678	-3.177013	0.568531
27	1	0	4.355063	-3.270242	-0.944944
28	35	0	-3.073249	0.129638	0.034819

Rotational constants (GHZ): 0.6825016 0.3388602 0.2355357

4-bromothymolate
Electronic Energy -3114.231650
Stoichiometry C10H14BrO2(1-)
Framework group C1[X(C10H14BrO2)]
Deg. of freedom 75
Full point group C1 NOp 1
Largest Abelian subgroup C1 NOp 1
Largest concise Abelian subgroup C1 NOp 1
Standard orientation:

Center Number	Atomic Number	Atomic Type	Coordinates (Angstroms) X	Y	Z
1	6	0	0.694969	1.592221	0.134296
2	6	0	1.616394	0.528297	0.042134
3	6	0	1.089053	-0.788970	-0.085043
4	6	0	-0.287359	-0.959440	-0.102334
5	6	0	-1.157269	0.119141	-0.000586
6	6	0	-0.686875	1.422977	0.118356
7	1	0	1.092775	2.597647	0.227342
8	1	0	-0.700488	-1.955347	-0.199978
9	8	0	2.910627	0.749485	0.072712
10	6	0	2.043771	-1.964259	-0.163592
11	1	0	2.915231	-1.619728	-0.727764
12	6	0	1.475376	-3.183091	-0.887436
13	1	0	1.106150	-2.926346	-1.883496
14	1	0	2.252182	-3.943270	-1.002267
15	1	0	0.653602	-3.641011	-0.330319

16	6	0	2.534216	-2.362001	1.234476
17	1	0	3.287632	-3.152542	1.172310
18	1	0	2.977958	-1.513634	1.759250
19	1	0	1.702151	-2.736599	1.838319
20	6	0	-1.595655	2.614741	0.225719
21	1	0	-2.244584	2.544710	1.102117
22	1	0	-1.012378	3.532282	0.306164
23	1	0	-2.247182	2.700142	-0.647358
24	8	0	3.902517	3.127106	-0.351712
25	1	0	4.473359	3.024292	-1.119854
26	1	0	3.490360	2.222813	-0.198533
27	35	0	-3.046047	-0.236109	-0.033139

Rotational constants (GHZ): 0.7009606 0.3383014 0.2384508

4-chlorothymol

Electronic Energy -1000.683458

Stoichiometry C10H15ClO2

Framework group C1[X(C10H15ClO2)]

Deg. of freedom 78

Full point group C1 NOp 1

Largest Abelian subgroup C1 NOp 1

Largest concise Abelian subgroup C1 NOp 1

Standard orientation:

Center Number	Atomic Number	Atomic Type	Coordinates (Angstroms) X	Y	Z
1	6	0	-0.060667	1.515350	-0.013219
2	6	0	-0.983489	0.478916	-0.089987
3	6	0	-0.564840	-0.857538	-0.127789
4	6	0	0.802786	-1.095730	-0.079252
5	6	0	1.711249	-0.052231	0.002491
6	6	0	1.310877	1.278078	0.036694
7	1	0	-0.418747	2.538135	0.009335
8	1	0	1.173892	-2.111377	-0.107806
9	8	0	-2.324810	0.731170	-0.134854
10	1	0	-2.514606	1.696797	-0.126583
11	6	0	-1.584168	-1.978821	-0.184998
12	1	0	-2.409559	-1.625121	-0.808066
13	6	0	-2.147947	-2.263548	1.212521
14	1	0	-2.570340	-1.365257	1.666809
15	1	0	-2.935600	-3.019797	1.160849
16	1	0	-1.360696	-2.639828	1.872091
17	6	0	-1.048626	-3.262337	-0.814755
18	1	0	-1.860819	-3.984691	-0.923846
19	1	0	-0.624003	-3.080458	-1.804883
20	1	0	-0.278737	-3.729000	-0.195010
21	6	0	2.282399	2.418473	0.120922
22	1	0	2.969580	2.417540	-0.728454
23	1	0	1.751632	3.370174	0.130858
24	1	0	2.890146	2.355315	1.026696
25	8	0	-3.070962	3.347680	-0.130502
26	1	0	-3.727369	3.519655	0.555965
27	1	0	-3.505444	3.585779	-0.958871
28	17	0	3.428317	-0.449550	0.057529

Rotational constants (GHZ): 0.6843679 0.4990451 0.3033784

4-chlorothymolate

Electronic Energy -1000.213427

Stoichiometry C10H14ClO2(1-)

Framework group C1[X(C10H14ClO2)]

Deg. of freedom 75

Full point group C1 NOp 1

Largest Abelian subgroup C1 NOp 1

Largest concise Abelian subgroup C1 NOp 1

Standard orientation:

Center Atomic Atomic Coordinates (Angstroms)

Number Number Type X Y Z

1	6	0	-0.051353	1.554147	0.107143
2	6	0	1.006391	0.624645	0.017641
3	6	0	0.662994	-0.753729	-0.097197
4	6	0	-0.677498	-1.110387	-0.108094
5	6	0	-1.683045	-0.158476	-0.011669
6	6	0	-1.396328	1.197342	0.097396
7	1	0	0.205918	2.604955	0.191646
8	1	0	-0.957553	-2.152824	-0.195436
9	8	0	2.258453	1.021726	0.038620
10	6	0	1.766379	-1.791373	-0.168067
11	1	0	2.583064	-1.340964	-0.739584
12	6	0	1.364386	-3.082912	-0.877100
13	1	0	0.964533	-2.888913	-1.875515
14	1	0	2.235143	-3.734632	-0.984220
15	1	0	0.610457	-3.639262	-0.313769
16	6	0	2.308183	-2.104442	1.232513
17	1	0	3.163609	-2.783780	1.176271
18	1	0	2.629989	-1.198007	1.748945
19	1	0	1.536154	-2.585000	1.841086
20	6	0	-2.468350	2.244878	0.199859
21	1	0	-3.098274	2.086178	1.078743
22	1	0	-2.024740	3.237923	0.273592
23	1	0	-3.126740	2.228613	-0.672276
24	8	0	2.944134	3.520416	-0.276995
25	1	0	3.245349	3.593327	-1.188300
26	1	0	2.637424	2.568809	-0.169670
27	17	0	-3.365623	-0.708925	-0.036415

Rotational constants (GHZ): 0.7032400 0.4966340 0.3075033

4-methoxyphenol
Electronic Energy -498.368030
Stoichiometry C7H10O3
Framework group C1[X(C7H10O3)]
Deg. of freedom 54
Full point group C1 NOp 1
Largest Abelian subgroup C1 NOp 1
Largest concise Abelian subgroup C1 NOp 1
Standard orientation:

Center Number	Atomic Number	Atomic Type	Coordinates (Angstroms) X	Y	Z
1	6	0	1.269949	1.028952	0.005199
2	6	0	-0.046978	1.479645	0.003503
3	6	0	-1.101315	0.579923	-0.005149
4	6	0	-0.834550	-0.787372	-0.012495
5	6	0	0.474365	-1.238825	-0.010922
6	6	0	1.532925	-0.335058	-0.002045
7	1	0	2.073349	1.752296	0.012256
8	1	0	-0.255415	2.542937	0.009249
9	1	0	-1.653394	-1.496847	-0.019683
10	1	0	0.687131	-2.301238	-0.016671
11	8	0	-2.380916	1.077774	-0.006599
12	1	0	-3.047268	0.354749	-0.006328
13	8	0	-4.377067	-0.779474	-0.003966
14	1	0	-4.850775	-0.784365	0.836912
15	1	0	-5.044862	-0.568165	-0.668017
16	8	0	2.796310	-0.878305	-0.000988
17	6	0	3.903412	0.015864	0.007738
18	1	0	4.793917	-0.609240	0.006157
19	1	0	3.904353	0.651327	-0.881538
20	1	0	3.899497	0.639826	0.905118

Rotational constants (GHZ): 3.8013424 0.5606876 0.4912496

4-methoxyphenolate
Electronic Energy -497.894732
Stoichiometry C7H9O3(1-)
Framework group C1[X(C7H9O3)]
Deg. of freedom 51
Full point group C1 NOp 1

Largest Abelian subgroup C1 NOp 1
Largest concise Abelian subgroup C1 NOp 1
Standard orientation:

Center Number	Atomic Number	Atomic Type	Coordinates (Angstroms) X	Y	Z
1	6	0	-1.117685	1.287800	-0.051521
2	6	0	0.235357	1.573296	0.059363
3	6	0	1.219881	0.569135	-0.076229
4	6	0	0.753344	-0.739446	-0.335191
5	6	0	-0.601260	-1.020739	-0.444437
6	6	0	-1.540796	-0.009657	-0.303374
7	1	0	-1.854649	2.077035	0.053553
8	1	0	0.559378	2.590659	0.252002
9	1	0	1.479716	-1.536042	-0.453687
10	1	0	-0.935233	-2.033025	-0.646423
11	8	0	2.503158	0.846814	0.031223
12	8	0	4.316497	-1.013536	0.233964
13	1	0	4.589373	-1.015422	1.156997
14	1	0	3.585608	-0.324553	0.168686
15	8	0	-2.900896	-0.288516	-0.450412
16	6	0	-3.548231	-0.644616	0.771412
17	1	0	-3.483990	0.172034	1.496234
18	1	0	-4.594136	-0.837638	0.534916
19	1	0	-3.099803	-1.545784	1.199400

Rotational constants (GHZ): 3.3501266 0.5853183 0.5210443

4-hydroxybenzonitrile
Electronic Energy -476.089821
Stoichiometry C7H7NO2
Framework group C1[X(C7H7NO2)]
Deg. of freedom 45
Full point group C1 NOp 1
Largest Abelian subgroup C1 NOp 1
Largest concise Abelian subgroup C1 NOp 1
Standard orientation:

Center Number	Atomic Number	Atomic Type	Coordinates (Angstroms) X	Y	Z
1	6	0	1.340141	1.247216	0.004309
2	6	0	0.003427	1.586637	0.003780
3	6	0	-0.970701	0.586713	-0.001238
4	6	0	-0.595469	-0.758119	-0.006037
5	6	0	0.743252	-1.097491	-0.005656
6	6	0	1.719862	-0.098732	-0.000419
7	1	0	2.097731	2.020832	0.008339
8	1	0	-0.304621	2.624570	0.007358
9	1	0	-1.355773	-1.528874	-0.010397
10	1	0	1.037791	-2.139311	-0.009457
11	8	0	-2.265798	0.973669	-0.001253
12	1	0	-2.886794	0.203988	-0.003877
13	8	0	-4.087012	-1.003595	-0.012494
14	1	0	-4.564533	-1.044034	0.825504
15	1	0	-4.763940	-0.843323	-0.681863
16	6	0	3.099917	-0.450731	0.000179
17	7	0	4.217149	-0.735342	0.000694

Rotational constants (GHZ): 3.6993506 0.5923894 0.5118202

4-cyanophenolate
Electronic Energy -475.626501
Stoichiometry C7H6NO2(1-)
Framework group C1[X(C7H6NO2)]
Deg. of freedom 42
Full point group C1 NOp 1
Largest Abelian subgroup C1 NOp 1
Largest concise Abelian subgroup C1 NOp 1

Standard orientation:

Center Number	Atomic Number	Atomic Type	Coordinates (Angstroms) X	Y	Z
1	6	0	-1.269619	1.255319	0.013737
2	6	0	0.064371	1.587131	0.004769
3	6	0	1.081057	0.592386	-0.017557
4	6	0	0.647649	-0.763044	-0.030438
5	6	0	-0.687541	-1.092066	-0.021336
6	6	0	-1.670209	-0.090237	0.001002
7	1	0	-2.022641	2.034910	0.030927
8	1	0	0.367103	2.628094	0.014758
9	1	0	1.398314	-1.545213	-0.048160
10	1	0	-0.989191	-2.133113	-0.031585
11	8	0	2.335217	0.911546	-0.025713
12	8	0	4.218436	-0.946353	0.045887
13	1	0	3.787475	-1.804683	-0.022997
14	1	0	3.475376	-0.281357	0.014811
15	6	0	-3.045085	-0.431583	0.009934
16	7	0	-4.167058	-0.709659	0.017170

Rotational constants (GHZ): 3.7989011 0.6032874 0.5206940

4-nitrophenol
Electronic Energy -588.365413
Stoichiometry C6H7NO4
Framework group C1[X(C6H7NO4)]
Deg. of freedom 48
Full point group C1 NOp 1
Largest Abelian subgroup C1 NOp 1
Largest concise Abelian subgroup C1 NOp 1
Standard orientation:

Center Number	Atomic Number	Atomic Type	Coordinates (Angstroms) X	Y	Z
1	6	0	-0.918801	1.327207	-0.003145
2	6	0	0.422312	1.636792	-0.002067
3	6	0	1.377827	0.615079	0.002320
4	6	0	0.974513	-0.724816	0.005998
5	6	0	-0.367760	-1.037630	0.005182
6	6	0	-1.304936	-0.010198	0.000541
7	1	0	-1.663806	2.110184	-0.006752
8	1	0	0.752258	2.667577	-0.004802
9	1	0	1.719022	-1.510362	0.009757
10	1	0	-0.689586	-2.069285	0.008092
11	8	0	2.672572	0.975041	0.002955
12	1	0	3.285258	0.195645	0.004551
13	8	0	4.451027	-1.019557	0.009835
14	1	0	4.929262	-1.066045	-0.827529
15	1	0	5.129451	-0.876785	0.681761
16	7	0	-2.710837	-0.338472	-0.000564
17	8	0	-3.529962	0.572781	-0.006022
18	8	0	-3.041754	-1.518292	0.003968

Rotational constants (GHZ): 2.9071771 0.4960908 0.4246034

4-nitrophenolate
Electronic Energy -587.906736
Stoichiometry C6H6NO4(1-)
Framework group C1[X(C6H6NO4)]
Deg. of freedom 45
Full point group C1 NOp 1
Largest Abelian subgroup C1 NOp 1
Largest concise Abelian subgroup C1 NOp 1
Standard orientation:

Center Number	Atomic Number	Atomic Type	Coordinates (Angstroms) X	Y	Z
1	6	0	-0.861535	1.336602	-0.001779

2	6	0	0.468817	1.646668	0.000589
3	6	0	1.476526	0.629902	0.005293
4	6	0	1.022196	-0.728285	0.008001
5	6	0	-0.308861	-1.036527	0.005570
6	6	0	-1.267093	-0.008877	0.000511
7	1	0	-1.607143	2.120019	-0.005530
8	1	0	0.790757	2.681230	-0.001224
9	1	0	1.764765	-1.517608	0.012205
10	1	0	-0.632118	-2.068526	0.007561
11	8	0	2.719506	0.926737	0.006950
12	8	0	4.612981	-0.968633	-0.016390
13	1	0	4.169604	-1.822286	0.030186
14	1	0	3.880796	-0.300401	-0.005609
15	7	0	-2.637758	-0.328853	-0.002250
16	8	0	-3.479391	0.580720	-0.006390
17	8	0	-2.988428	-1.517243	-0.000538

Rotational constants (GHZ): 2.9354748 0.5041569 0.4302673

4-nitrothymol
Electronic Energy -745.562979
Stoichiometry C10H15NO4
Framework group C1[X(C10H15NO4)]
Deg. of freedom 84
Full point group C1 NOp 1
Largest Abelian subgroup C1 NOp 1
Largest concise Abelian subgroup C1 NOp 1
Standard orientation:

Center Number	Atomic Number	Atomic Type	Coordinates (Angstroms) X	Y	Z
1	6	0	-0.663385	-1.020101	-0.082352
2	6	0	0.704922	-0.872297	-0.122307
3	6	0	1.202042	0.445315	-0.105766
4	6	0	0.340155	1.538441	-0.048109
5	6	0	-1.039083	1.398231	0.013459
6	6	0	-1.519394	0.080601	-0.006288
7	1	0	-1.101527	-2.007092	-0.095917
8	1	0	0.764927	2.534811	-0.057686
9	8	0	2.537382	0.610775	-0.157041
10	1	0	2.804717	1.563539	-0.152702
11	8	0	3.453929	3.133998	-0.151224
12	1	0	4.122952	3.258616	0.533569
13	1	0	3.899642	3.344713	-0.981253
14	7	0	-2.932941	-0.208540	0.037824
15	8	0	-3.323681	-1.309923	-0.341428
16	8	0	-3.706647	0.643271	0.458082
17	6	0	1.659608	-2.048469	-0.151405
18	1	0	2.497117	-1.759529	-0.791482
19	6	0	2.217924	-2.318855	1.251224
20	1	0	2.962680	-3.117961	1.216993
21	1	0	2.692187	-1.431784	1.675058
22	1	0	1.416318	-2.631763	1.926193
23	6	0	1.044666	-3.317489	-0.735143
24	1	0	0.255913	-3.718657	-0.093593
25	1	0	0.621282	-3.142662	-1.726989
26	1	0	1.813128	-4.088125	-0.827238
27	6	0	-1.882857	2.639337	0.057870
28	1	0	-2.681760	2.612450	-0.684089
29	1	0	-2.356295	2.760073	1.033258
30	1	0	-1.256145	3.509902	-0.133100

Rotational constants (GHZ): 0.6516378 0.4354997 0.2744360

4-nitrothymolate
Electronic Energy -745.562979
Stoichiometry C10H14NO4(1-)
Framework group C1[X(C10H14NO4)]
Deg. of freedom 81
Full point group C1 NOp 1
Largest Abelian subgroup C1 NOp 1

Largest concise Abelian subgroup C1 NOp 1
Standard orientation:

Center Number	Atomic Number	Atomic Type	Coordinates (Angstroms) X	Y	Z
1	6	0	-0.597335	1.019303	-0.072081
2	6	0	0.749222	0.818633	-0.159285
3	6	0	1.238223	-0.538049	-0.186962
4	6	0	0.276859	-1.587330	-0.117832
5	6	0	-1.080836	-1.391532	-0.025751
6	6	0	-1.527189	-0.042529	-0.002236
7	1	0	-0.989825	2.025142	-0.055958
8	1	0	0.658400	-2.601648	-0.142840
9	8	0	2.487625	-0.793781	-0.278118
10	8	0	3.522983	-3.246816	-0.044039
11	1	0	4.120551	-3.194195	0.709028
12	1	0	3.106980	-2.348942	-0.113478
13	7	0	-2.886162	0.306210	0.084417
14	8	0	-3.214153	1.507257	0.101871
15	8	0	-3.758482	-0.572851	0.144350
16	6	0	1.748415	1.954922	-0.201084
17	1	0	2.548498	1.641249	-0.877713
18	6	0	2.376627	2.171689	1.181629
19	1	0	2.815836	1.252011	1.572519
20	1	0	3.164169	2.928522	1.132579
21	1	0	1.620655	2.516653	1.893109
22	6	0	1.173562	3.265296	-0.732308
23	1	0	0.712233	3.135853	-1.714363
24	1	0	0.421598	3.682495	-0.057144
25	1	0	1.970292	4.006653	-0.829211
26	6	0	-1.968086	-2.602277	0.038414
27	1	0	-2.557358	-2.619778	0.956192
28	1	0	-2.676186	-2.625249	-0.790935
29	1	0	-1.353260	-3.501470	-0.000243

Rotational constants (GHZ): 0.6638475 0.4347930 0.2742302

- 1H_2O/CAM-B3LYP 6-311G+dp/PCM

H_2O

Electronic Energy -152.884990
Stoichiometry H4O2
Framework group C1[X(H4O2)]
Deg. of freedom 12
Full point group C1 NOp 1
Largest Abelian subgroup C1 NOp 1
Largest concise Abelian subgroup C1 NOp 1
Standard orientation:

Center Number	Atomic Number	Atomic Type	Coordinates (Angstroms) X	Y	Z
1	8	0	1.334098	-0.000066	0.089003
2	1	0	1.802929	0.766671	-0.260945
3	1	0	1.805153	-0.765474	-0.260862
4	8	0	-1.492632	-0.000059	-0.117740
5	1	0	-1.818990	0.000437	0.788019
6	1	0	-0.520820	-0.000636	-0.036318

Rotational constants (GHZ): 227.6237525 6.7080289 6.6950899

OH^-

Electronic Energy -152.393650
Stoichiometry H3O2(1-)
Framework group C1[X(H3O2)]
Deg. of freedom 9
Full point group C1 NOp 1
Largest Abelian subgroup C1 NOp 1
Largest concise Abelian subgroup C1 NOp 1

Standard orientation:

Center Number	Atomic Number	Atomic Type	Coordinates (Angstroms) X	Y	Z
1	8	0	1.260773	0.034490	-0.105350
2	1	0	1.669783	-0.137266	0.749182
3	8	0	-1.248911	-0.110104	-0.006106
4	1	0	-1.570442	0.780839	0.161116
5	1	0	-0.194238	-0.038664	-0.018653

Rotational constants (GHZ): 330.2884982 8.9463552 8.9345926

Phenol
Electronic Energy -383.851460
Stoichiometry C6H8O2
Framework group C1[X(C6H8O2)]
Deg. of freedom 42
Full point group C1 NOp 1
Largest Abelian subgroup C1 NOp 1
Largest concise Abelian subgroup C1 NOp 1
Standard orientation:

Center Number	Atomic Number	Atomic Type	Coordinates (Angstroms) X	Y	Z
1	6	0	2.299681	-0.636070	0.000103
2	6	0	1.074936	-1.283268	0.000036
3	6	0	-0.101809	-0.539616	-0.000071
4	6	0	-0.041477	0.851868	-0.000125
5	6	0	1.192748	1.488022	-0.000058
6	6	0	2.369476	0.752629	0.000063
7	1	0	3.210615	-1.223439	0.000176
8	1	0	1.007148	-2.364014	0.000051
9	1	0	-0.960991	1.426349	-0.000233
10	1	0	1.230377	2.571314	-0.000094
11	1	0	3.329412	1.253567	0.000127
12	8	0	-1.276510	-1.217316	-0.000126
13	1	0	-2.029068	-0.603808	-0.000262
14	8	0	-3.518559	0.486473	0.000071
15	1	0	-4.094700	0.492744	-0.769827
16	1	0	-4.093570	0.492634	0.770816

Rotational constants (GHZ): 4.3158479 1.1226857 0.8947008

Phenolate
Electronic Energy -383.378420
Stoichiometry C6H7O2(1-)
Framework group C1[X(C6H7O2)]
Deg. of freedom 39
Full point group C1 NOp 1
Largest Abelian subgroup C1 NOp 1
Largest concise Abelian subgroup C1 NOp 1
Standard orientation:

Center Number	Atomic Number	Atomic Type	Coordinates (Angstroms) X	Y	Z
1	6	0	2.184539	-0.790552	0.081527
2	6	0	0.900642	-1.300021	0.015292
3	6	0	-0.247804	-0.455780	-0.073532
4	6	0	0.031593	0.945308	-0.090793
5	6	0	1.323211	1.435059	-0.023047
6	6	0	2.425391	0.583721	0.064321
7	1	0	3.023522	-1.479787	0.147850
8	1	0	0.726346	-2.371282	0.028705
9	1	0	-0.813943	1.622193	-0.159323
10	1	0	1.479480	2.511349	-0.039811
11	1	0	3.434599	0.976693	0.116114
12	8	0	-1.435271	-0.930860	-0.133712
13	8	0	-3.671391	0.439656	0.037503

14	1	0	-3.901676	0.205579	0.938403
15	1	0	-2.800466	-0.041517	-0.104873

Rotational constants (GHZ): 4.7601566 1.1053605 0.9020238

Thymol
Electronic Energy -541.052650
Stoichiometry C10H16O2
Framework group C1[X(C10H16O2)]
Deg. of freedom 78
Full point group C1 NOp 1
Largest Abelian subgroup C1 NOp 1
Largest concise Abelian subgroup C1 NOp 1
Standard orientation:

Center Number	Atomic Number	Atomic Type	Coordinates (Angstroms) X	Y	Z
1	6	0	-2.239615	0.479480	-0.195256
2	1	0	-2.080173	1.324302	-0.871332
3	6	0	-0.921687	-0.266236	-0.116622
4	6	0	-0.825218	-1.648090	-0.042539
5	6	0	0.279145	0.457872	-0.092441
6	6	0	0.400293	-2.300825	0.053698
7	1	0	-1.729517	-2.243760	-0.065199
8	6	0	1.505483	-0.186664	-0.001132
9	6	0	1.582768	-1.575631	0.074786
10	1	0	0.429466	-3.383136	0.108501
11	1	0	2.411839	0.411237	0.010128
12	6	0	-2.614727	1.060210	1.174736
13	1	0	-3.531754	1.652079	1.106205
14	1	0	-2.783097	0.257484	1.898271
15	1	0	-1.821521	1.704369	1.555458
16	6	0	-3.387350	-0.358097	-0.755822
17	1	0	-4.272530	0.267304	-0.893167
18	1	0	-3.131653	-0.798518	-1.722076
19	1	0	-3.667829	-1.168511	-0.077645
20	6	0	2.922137	-2.256445	0.174045
21	1	0	3.548984	-2.025189	-0.691089
22	1	0	3.465385	-1.933141	1.065778
23	1	0	2.810059	-3.340198	0.225512
24	8	0	0.194137	1.814595	-0.164099
25	1	0	1.078119	2.215518	-0.166679
26	8	0	2.701792	3.107260	-0.174458
27	1	0	2.917968	3.689909	0.559363
28	1	0	2.981440	3.561970	-0.974302

Rotational constants (GHZ): 0.9481365 0.6746986 0.4206987

Thymolate
Electronic Energy -540.577064
Stoichiometry C10H15O2(1-)
Framework group C1[X(C10H15O2)]
Deg. of freedom 75
Full point group C1 NOp 1
Largest Abelian subgroup C1 NOp 1
Largest concise Abelian subgroup C1 NOp 1
Standard orientation:

Center Number	Atomic Number	Atomic Type	Coordinates (Angstroms) X	Y	Z
1	6	0	-2.206984	0.097370	-0.195215
2	1	0	-2.173081	0.963386	-0.865036
3	6	0	-0.785068	-0.415285	-0.111878
4	6	0	-0.455721	-1.758425	-0.095460
5	6	0	0.256897	0.571889	-0.024685
6	6	0	0.863698	-2.206758	0.003269
7	1	0	-1.246182	-2.500773	-0.161889
8	6	0	1.587442	0.081717	0.069967
9	6	0	1.892995	-1.270671	0.083478
10	1	0	1.082821	-3.269879	0.015906

11	1	0	2.379575	0.822357	0.136170
12	6	0	-2.677569	0.624211	1.166839
13	1	0	-3.671760	1.081195	1.093227
14	1	0	-2.730238	-0.191386	1.896182
15	1	0	-1.976002	1.376285	1.528165
16	6	0	-3.210733	-0.908386	-0.756384
17	1	0	-4.191307	-0.437602	-0.880316
18	1	0	-2.892219	-1.293414	-1.728774
19	1	0	-3.344440	-1.763855	-0.086336
20	6	0	3.332709	-1.721040	0.149889
21	1	0	3.800270	-1.702379	-0.840498
22	1	0	3.922682	-1.068950	0.798847
23	1	0	3.411494	-2.742604	0.531531
24	8	0	-0.020309	1.826054	-0.030753
25	8	0	1.699965	3.807958	-0.216702
26	1	0	1.522611	4.058257	-1.125182
27	1	0	1.082525	3.029546	-0.071278

Rotational constants (GHZ): 0.9019192 0.7205045 0.427509

- 1H_2O/CAM-B3LYP 6-311G+dp/CPCM

H_2O
Electronic Energy -152.885058
Stoichiometry H4O2
Framework group C1[X(H4O2)]
Deg. of freedom 12
Full point group C1 NOp 1
Largest Abelian subgroup C1 NOp 1
Largest concise Abelian subgroup C1 NOp 1
Standard orientation:

Center Number	Atomic Number	Atomic Type	Coordinates (Angstroms) X	Y	Z
1	8	0	1.354996	-0.000069	0.095350
2	1	0	1.785425	0.769030	-0.288545
3	1	0	1.787533	-0.767656	-0.289197
4	8	0	-1.498925	0.000064	-0.119702
5	1	0	-1.882099	0.000036	0.759773
6	1	0	-0.539428	-0.001371	0.012785

Rotational constants (GHZ): 226.2594154 6.5900974 6.5775524

OH^-
Electronic Energy -152.393735
Stoichiometry H3O2(1-)
Framework group C1[X(H3O2)]
Deg. of freedom 9
Full point group C1 NOp 1
Largest Abelian subgroup C1 NOp 1
Largest concise Abelian subgroup C1 NOp 1
Standard orientation:

Center Number	Atomic Number	Atomic Type	Coordinates (Angstroms) X	Y	Z
1	8	0	1.248236	0.067572	-0.087812
2	1	0	1.685496	-0.368794	0.647813
3	8	0	-1.239951	-0.105367	-0.035213
4	1	0	-1.567710	0.704533	0.363085
5	1	0	-0.184066	-0.033376	-0.026700

Rotational constants (GHZ): 336.8904690 9.0873788 9.0602910

Phenol
Electronic Energy -383.851531
Stoichiometry C6H8O2
Framework group C1[X(C6H8O2)]
Deg. of freedom 42

Full point group C1 NOp 1
Largest Abelian subgroup C1 NOp 1
Largest concise Abelian subgroup C1 NOp 1
Standard orientation:

Center Number	Atomic Number	Atomic Type	Coordinates (Angstroms) X	Y	Z
1	6	0	2.299681	-0.636070	0.000103
2	6	0	1.074936	-1.283268	0.000036
3	6	0	-0.101809	-0.539616	-0.000071
4	6	0	-0.041477	0.851868	-0.000125
5	6	0	1.192748	1.488022	-0.000058
6	6	0	2.369476	0.752629	0.000063
7	1	0	3.210615	-1.223439	0.000176
8	1	0	1.007148	-2.364014	0.000051
9	1	0	-0.960991	1.426349	-0.000233
10	1	0	1.230377	2.571314	-0.000094
11	1	0	3.329412	1.253567	0.000127
12	8	0	-1.276510	-1.217316	-0.000126
13	1	0	-2.029068	-0.603808	-0.000262
14	8	0	-3.518559	0.486473	0.000071
15	1	0	-4.094700	0.492744	-0.769827
16	1	0	-4.093570	0.492634	0.770816

Rotational constants (GHZ): 4.3158479 1.1226857 0.8947008

Phenolate
Electronic Energy -383.378569
Stoichiometry C6H7O2(1-)
Framework group C1[X(C6H7O2)]
Deg. of freedom 39
Full point group C1 NOp 1
Largest Abelian subgroup C1 NOp 1
Largest concise Abelian subgroup C1 NOp 1
Standard orientation:

Center Number	Atomic Number	Atomic Type	Coordinates (Angstroms) X	Y	Z
1	6	0	2.184539	-0.790552	0.081527
2	6	0	0.900642	-1.300021	0.015292
3	6	0	-0.247804	-0.455780	-0.073532
4	6	0	0.031593	0.945308	-0.090793
5	6	0	1.323211	1.435059	-0.023047
6	6	0	2.425391	0.583721	0.064321
7	1	0	3.023522	-1.479787	0.147850
8	1	0	0.726346	-2.371282	0.028705
9	1	0	-0.813943	1.622193	-0.159323
10	1	0	1.479480	2.511349	-0.039811
11	1	0	3.434599	0.976693	0.116114
12	8	0	-1.435271	-0.930860	-0.133712
13	8	0	-3.671391	0.439656	0.037503
14	1	0	-3.901676	0.205579	0.938403
15	1	0	-2.800466	-0.041517	-0.104873

Rotational constants (GHZ): 4.7601566 1.1053605 0.9020238

Thymol
Electronic Energy -541.052726
Stoichiometry C10H16O2
Framework group C1[X(C10H16O2)]
Deg. of freedom 78
Full point group C1 NOp 1
Largest Abelian subgroup C1 NOp 1
Largest concise Abelian subgroup C1 NOp 1
Standard orientation:

Center Number	Atomic Number	Atomic Type	Coordinates (Angstroms) X	Y	Z

1	6	0	-2.239615	0.479480	-0.195256
2	1	0	-2.080173	1.324302	-0.871332
3	6	0	-0.921687	-0.266236	-0.116622
4	6	0	-0.825218	-1.648090	-0.042539
5	6	0	0.279145	0.457872	-0.092441
6	6	0	0.400293	-2.300825	0.053698
7	1	0	-1.729517	-2.243760	-0.065199
8	6	0	1.505483	-0.186664	-0.001132
9	6	0	1.582768	-1.575631	0.074786
10	1	0	0.429466	-3.383136	0.108501
11	1	0	2.411839	0.411237	0.010128
12	6	0	-2.614727	1.060210	1.174736
13	1	0	-3.531754	1.652079	1.106205
14	1	0	-2.783097	0.257484	1.898271
15	1	0	-1.821521	1.704369	1.555458
16	6	0	-3.387350	-0.358097	-0.755822
17	1	0	-4.272530	0.267304	-0.893167
18	1	0	-3.131653	-0.798518	-1.722076
19	1	0	-3.667829	-1.168511	-0.077645
20	6	0	2.922137	-2.256445	0.174045
21	1	0	3.548984	-2.025189	-0.691089
22	1	0	3.465385	-1.933141	1.065778
23	1	0	2.810059	-3.340198	0.225512
24	8	0	0.194137	1.814595	-0.164099
25	1	0	1.078119	2.215518	-0.166679
26	8	0	2.701792	3.107260	-0.174458
27	1	0	2.917968	3.689909	0.559363
28	1	0	2.981440	3.561970	-0.974302

Rotational constants (GHZ): 0.9481365 0.6746986 0.4206987

Thymolate

Electronic Energy- -540.577261

Stoichiometry C10H15O2(1-)

Framework group C1[X(C10H15O2)]

Deg. of freedom 75

Full point group C1 NOp 1

Largest Abelian subgroup C1 NOp 1

Largest concise Abelian subgroup C1 NOp 1

Standard orientation:

Center Number	Atomic Number	Atomic Type	Coordinates (Angstroms) X	Y	Z
1	6	0	-2.206984	0.097370	-0.195215
2	1	0	-2.173081	0.963386	-0.865036
3	6	0	-0.785068	-0.415285	-0.111878
4	6	0	-0.455721	-1.758425	-0.095460
5	6	0	0.256897	0.571889	-0.024685
6	6	0	0.863698	-2.206758	0.003269
7	1	0	-1.246182	-2.500773	-0.161889
8	6	0	1.587442	0.081717	0.069967
9	6	0	1.892995	-1.270671	0.083478
10	1	0	1.082821	-3.269879	0.015906
11	1	0	2.379575	0.822357	0.136170
12	6	0	-2.677569	0.624211	1.166839
13	1	0	-3.671760	1.081195	1.093227
14	1	0	-2.730238	-0.191386	1.896182
15	1	0	-1.976002	1.376285	1.528165
16	6	0	-3.210733	-0.908386	-0.756384
17	1	0	-4.191307	-0.437602	-0.880316
18	1	0	-2.892219	-1.293414	-1.728774
19	1	0	-3.344440	-1.763855	-0.086336
20	6	0	3.332709	-1.721040	0.149889
21	1	0	3.800270	-1.702379	-0.840498
22	1	0	3.922682	-1.068950	0.798847
23	1	0	3.411494	-2.742604	0.531531
24	8	0	-0.020309	1.826054	-0.030753
25	8	0	1.699965	3.807958	-0.216702
26	1	0	1.522611	4.058257	-1.125182
27	1	0	1.082525	3.029546	-0.071278

Rotational constants (GHZ): 0.9019192 0.7205045 0.4275094

- $2H_2O$/CAM-B3LYP 6-311G+dp/SMD

H_2O

Electronic Energy -229.343629
Stoichiometry H6O3
Framework group C1[X(H6O3)]
Deg. of freedom 21
Full point group C1 NOp 1
Largest Abelian subgroup C1 NOp 1
Largest concise Abelian subgroup C1 NOp 1
Standard orientation:

Center Number	Atomic Number	Atomic Type	Coordinates (Angstroms) X	Y	Z
1	8	0	-0.000187	0.940505	-0.000188
2	1	0	-0.001448	1.649190	-0.656698
3	1	0	0.003182	1.390549	0.853531
4	8	0	2.421254	-0.481734	-0.016958
5	1	0	2.194989	-1.412321	0.079446
6	1	0	1.564450	-0.015387	-0.043160
7	8	0	-2.421563	-0.482556	-0.018046
8	1	0	-2.189798	-1.410520	0.089602
9	1	0	-1.567406	-0.011235	-0.041188

Rotational constants (GHZ): 16.0514252 2.4847890 2.1738153

OH^-

Electronic Energy -228.866593
Stoichiometry H5O3(1-)
Framework group C1[X(H5O3)]
Deg. of freedom 18
Full point group C1 NOp 1
Largest Abelian subgroup C1 NOp 1
Largest concise Abelian subgroup C1 NOp 1
Standard orientation:

Center Number	Atomic Number	Atomic Type	Coordinates (Angstroms) X	Y	Z
1	8	0	-0.000225	0.657416	0.000044
2	1	0	-0.000769	1.616660	-0.000144
3	8	0	-2.351148	-0.340430	0.086619
4	1	0	-2.355030	-0.801681	-0.754038
5	1	0	-1.410034	0.086466	0.103715
6	8	0	2.351414	-0.340291	-0.086624
7	1	0	1.410559	0.086532	-0.103892
8	1	0	2.354945	-0.801536	0.754044

Rotational constants (GHZ): 31.7538037 2.6124257 2.4454439

Phenol

Electronic Energy -460.306017
Stoichiometry C6H10O3
Framework group C1[X(C6H10O3)]
Deg. of freedom 51
Full point group C1 NOp 1
Largest Abelian subgroup C1 NOp 1
Largest concise Abelian subgroup C1 NOp 1
Standard orientation:

Center Number	Atomic Number	Atomic Type	Coordinates (Angstroms) X	Y	Z
1	6	0	2.579260	-1.016676	-0.040705
2	6	0	1.253485	-1.237462	-0.379970
3	6	0	0.346478	-0.185920	-0.328551
4	6	0	0.765128	1.081408	0.061626

5	6	0	2.096200	1.289930	0.395107
6	6	0	3.009913	0.246168	0.347385
7	1	0	3.283009	-1.839665	-0.083224
8	1	0	0.907538	-2.215265	-0.691722
9	1	0	0.046779	1.892161	0.109070
10	1	0	2.417832	2.279297	0.698418
11	1	0	4.046814	0.414137	0.609927
12	8	0	-0.950101	-0.442795	-0.665179
13	1	0	-1.485870	0.376792	-0.670092
14	8	0	-2.896718	1.394032	-0.243515
15	1	0	-3.524160	1.828118	-0.825832
16	1	0	-3.367944	0.667279	0.203110
17	8	0	-3.222605	-1.107456	0.798023
18	1	0	-2.363787	-1.283333	0.378914
19	1	0	-3.707608	-1.934460	0.827446

Rotational constants (GHZ): 2.8958025 0.7313665 0.6242864

Phenolate

Electronic Energy -459.942619
Stoichiometry C6H9O3(1-)
Framework group C1[X(C6H9O3)]
Deg. of freedom 48
Full point group C1 NOp 1
Largest Abelian subgroup C1 NOp 1
Largest concise Abelian subgroup C1 NOp 1
Standard orientation:

Center Number	Atomic Number	Atomic Type	Coordinates (Angstroms) X	Y	Z
1	6	0	-2.253191	-1.199371	-0.094639
2	6	0	-0.859724	-1.208477	-0.096867
3	6	0	-0.101532	-0.000162	-0.001151
4	6	0	-0.859465	1.208197	0.095724
5	6	0	-2.252956	1.199182	0.095533
6	6	0	-2.975542	-0.000052	0.000962
7	1	0	-2.788653	-2.145917	-0.169196
8	1	0	-0.312317	-2.145183	-0.170476
9	1	0	-0.311939	2.144890	0.168570
10	1	0	-2.788203	2.145786	0.170885
11	1	0	-4.062832	-0.000018	0.001751
12	8	0	1.201996	-0.000265	-0.002171
13	8	0	2.836666	2.116821	-0.037236
14	1	0	3.365086	1.868616	-0.804494
15	1	0	2.165962	1.375378	0.030204
16	8	0	2.838341	-2.116195	0.039205
17	1	0	2.167163	-1.375504	-0.030878
18	1	0	3.364165	-1.866836	0.807871

Rotational constants (GHZ): 2.0697234 0.8518192 0.6062176

Thymol

Electronic Energy -617.505458
Stoichiometry C10H18O3
Framework group C1[X(C10H18O3)]
Deg. of freedom 87
Full point group C1 NOp 1
Largest Abelian subgroup C1 NOp 1
Largest concise Abelian subgroup C1 NOp 1
Standard orientation:

Center Number	Atomic Number	Atomic Type	Coordinates (Angstroms) X	Y	Z
1	6	0	-0.785067	-1.492031	0.192251
2	6	0	-0.023341	-0.331594	0.211952
3	6	0	-0.595222	0.923930	-0.037569
4	6	0	-1.960493	0.949725	-0.297058
5	6	0	-2.733301	-0.208301	-0.313709
6	6	0	-2.153045	-1.447675	-0.070821
7	1	0	-0.299290	-2.443054	0.388023

8	1	0	-2.443799	1.898475	-0.496335
9	8	0	1.318334	-0.380488	0.481215
10	1	0	1.638526	-1.305768	0.546185
11	6	0	0.263023	2.173791	0.014961
12	1	0	1.243031	1.905550	-0.389969
13	6	0	0.472178	2.625658	1.465688
14	1	0	0.897199	1.828483	2.078575
15	1	0	1.149506	3.482868	1.507647
16	1	0	-0.480129	2.925708	1.912500
17	6	0	-0.281439	3.326584	-0.825326
18	1	0	0.437386	4.149344	-0.832001
19	1	0	-0.457951	3.024875	-1.860520
20	1	0	-1.218910	3.716500	-0.420969
21	6	0	-2.963188	-2.714843	-0.092664
22	1	0	-4.020238	-2.502196	-0.257446
23	1	0	-2.624908	-3.384024	-0.888174
24	1	0	-2.868235	-3.260058	0.849631
25	8	0	3.850186	-0.375992	-0.938890
26	1	0	2.975222	-0.022834	-0.712474
27	1	0	3.909405	-0.320594	-1.899790
28	8	0	2.809098	-2.666496	0.286197
29	1	0	3.303530	-2.961278	1.059799
30	1	0	3.393668	-2.036962	-0.178432
31	1	0	-3.795582	-0.142687	-0.520664

Rotational constants (GHZ): 0.6703512 0.5670809 0.3399319

Thymolate
Electronic Energy -617.037656
Stoichiometry C10H17O3(1-)
Framework group C1[X(C10H17O3)]
Deg. of freedom 84
Full point group C1 NOp 1
Largest Abelian subgroup C1 NOp 1
Largest concise Abelian subgroup C1 NOp 1
Standard orientation:

Center Number	Atomic Number	Atomic Type	Coordinates (Angstroms) X	Y	Z
1	6	0	-1.548924	0.842635	0.091914
2	6	0	-0.204408	0.432598	0.011707
3	6	0	0.064382	-0.961051	-0.100770
4	6	0	-1.009386	-1.841062	-0.118963
5	6	0	-2.333854	-1.412879	-0.033629
6	6	0	-2.613364	-0.053820	0.070513
7	1	0	-1.747323	1.907254	0.176329
8	1	0	-0.817588	-2.905041	-0.203166
9	1	0	-3.141767	-2.136211	-0.050916
10	8	0	0.766711	1.328000	0.040331
11	6	0	1.503475	-1.436978	-0.160200
12	1	0	2.058575	-0.696962	-0.742837
13	6	0	1.687176	-2.788216	-0.848776
14	1	0	1.247027	-2.796014	-1.849089
15	1	0	2.752331	-3.012212	-0.948738
16	1	0	1.236496	-3.602689	-0.275622
17	6	0	2.123902	-1.474111	1.242512
18	1	0	3.188082	-1.722271	1.193083
19	1	0	2.024917	-0.513367	1.750972
20	1	0	1.629543	-2.233611	1.856050
21	6	0	-4.032239	0.447312	0.140478
22	1	0	-4.160531	1.168525	0.951151
23	1	0	-4.318142	0.951851	-0.787090
24	1	0	-4.733315	-0.373517	0.301096
25	8	0	0.391646	3.915384	-0.182111
26	1	0	0.070527	4.089603	-1.072742
27	1	0	0.469981	2.918819	-0.115766
28	8	0	3.405548	1.570374	-0.087435
29	1	0	2.430055	1.388827	-0.045704
30	1	0	3.479335	2.530381	-0.072009

Rotational constants (GHZ): 0.7419549 0.5837649 0.3467626

Carvacrol
Electronic Energy -617.505759
Stoichiometry C10H18O3
Framework group C1[X(C10H18O3)]
Deg. of freedom 87
Full point group C1 NOp 1
Largest Abelian subgroup C1 NOp 1
Largest concise Abelian subgroup C1 NOp 1
Standard orientation:

Center Number	Atomic Number	Atomic Type	Coordinates (Angstroms) X	Y	Z
1	6	0	-0.594602	1.533464	0.039032
2	6	0	-0.665704	0.175225	-0.271486
3	6	0	0.475397	-0.617570	-0.317376
4	6	0	1.731006	-0.084961	-0.049765
5	6	0	1.814808	1.271215	0.267116
6	6	0	0.670292	2.054255	0.306824
7	1	0	2.776511	1.721986	0.482857
8	8	0	-1.856181	-0.443951	-0.566884
9	1	0	-2.642352	0.092951	-0.349168
10	8	0	-3.456434	-2.570752	0.386089
11	1	0	-3.647241	-3.251348	-0.269520
12	1	0	-2.687585	-2.086319	0.039340
13	8	0	-4.519010	0.028714	0.114666
14	1	0	-4.421666	-0.937772	0.206302
15	1	0	-5.134069	0.158350	-0.616187
16	1	0	0.752388	3.108312	0.550421
17	1	0	0.366580	-1.668113	-0.564835
18	6	0	-1.825395	2.394362	0.079017
19	1	0	-2.372881	2.358611	-0.867409
20	1	0	-2.516569	2.075111	0.864212
21	1	0	-1.556505	3.432743	0.272885
22	6	0	2.964498	-0.965792	-0.096132
23	1	0	2.631738	-1.967045	-0.384695
24	6	0	3.966727	-0.483334	-1.147487
25	1	0	4.818784	-1.165804	-1.203094
26	1	0	3.509574	-0.430689	-2.138473
27	1	0	4.349988	0.509793	-0.898656
28	6	0	3.631343	-1.071274	1.277871
29	1	0	2.933417	-1.444901	2.030938
30	1	0	4.483745	-1.754407	1.237234
31	1	0	3.998909	-0.097077	1.611179

Rotational constants (GHZ): 1.1520898 0.3538607 0.2912536

Carvacrolate
Electronic Energy -617.040399
Stoichiometry C10H17O3(1-)
Framework group C1[X(C10H17O3)]
Deg. of freedom 84
Full point group C1 NOp 1
Largest Abelian subgroup C1 NOp 1
Largest concise Abelian subgroup C1 NOp 1
Standard orientation:

Center Number	Atomic Number	Atomic Type	Coordinates (Angstroms) X	Y	Z
1	6	0	-0.858323	1.543109	-0.214997
2	6	0	-0.914722	0.134207	-0.343178
3	6	0	0.289862	-0.588573	-0.235027
4	6	0	1.516763	0.024214	-0.004263
5	6	0	1.552825	1.413209	0.123393
6	6	0	0.373970	2.144532	0.015105
7	1	0	2.490884	1.926212	0.302554
8	8	0	-2.064066	-0.485700	-0.566812
9	8	0	-4.099175	-0.346591	1.115575
10	1	0	-4.386767	0.569268	1.189736
11	1	0	-3.323865	-0.326394	0.489902
12	8	0	-2.249665	-3.110290	-0.436805

13	1	0	-2.144075	-2.115756	-0.480163
14	1	0	-3.136454	-3.255360	-0.090867
15	1	0	0.243873	-1.668692	-0.338717
16	1	0	0.411610	3.226063	0.110236
17	6	0	-2.114087	2.359826	-0.337886
18	1	0	-2.634447	2.156920	-1.278507
19	1	0	-2.823600	2.139904	0.465518
20	1	0	-1.886734	3.426756	-0.299665
21	6	0	2.779947	-0.811297	0.100885
22	1	0	2.483030	-1.856483	-0.027877
23	6	0	3.437598	-0.679453	1.476997
24	1	0	2.743332	-0.948309	2.276885
25	1	0	4.308879	-1.335998	1.550411
26	1	0	3.775878	0.344985	1.654195
27	6	0	3.780296	-0.471019	-1.006989
28	1	0	4.654024	-1.125960	-0.949954
29	1	0	3.332803	-0.589929	-1.996809
30	1	0	4.130109	0.560896	-0.916770

Rotational constants (GHZ): 0.9945305 0.4163322 0.3281564

2,3-dimethylphenol
Electronic Energy -538.906764
Stoichiometry C8H14O3
Framework group C1[X(C8H14O3)]
Deg. of freedom 69
Full point group C1 NOp 1
Largest Abelian subgroup C1 NOp 1
Largest concise Abelian subgroup C1 NOp 1
Standard orientation:

Center Number	Atomic Number	Atomic Type	Coordinates (Angstroms) X	Y	Z
1	6	0	0.819750	0.566863	-0.110668
2	6	0	-0.013762	-0.540401	-0.292785
3	6	0	0.476550	-1.839821	-0.264785
4	6	0	1.826082	-2.049744	-0.039510
5	6	0	2.672209	-0.967252	0.159324
6	6	0	2.184297	0.338238	0.127730
7	1	0	2.217986	-3.059779	-0.014216
8	1	0	3.727620	-1.132244	0.342938
9	8	0	-1.361087	-0.404661	-0.523849
10	1	0	-1.695446	0.503022	-0.393363
11	8	0	-3.836265	-1.101435	0.673615
12	1	0	-4.422361	-1.634929	0.124575
13	1	0	-2.952387	-1.200866	0.281032
14	8	0	-3.257979	1.572318	0.014414
15	1	0	-3.711903	0.739158	0.242813
16	1	0	-3.734992	1.926214	-0.744864
17	1	0	-0.208047	-2.665800	-0.415944
18	6	0	0.238139	1.953747	-0.167404
19	1	0	-0.415903	2.074820	-1.034947
20	1	0	-0.359425	2.173717	0.722560
21	1	0	1.014113	2.712359	-0.237399
22	6	0	3.134849	1.484893	0.351789
23	1	0	3.185264	2.142881	-0.519622
24	1	0	2.829280	2.100403	1.201499
25	1	0	4.140170	1.112133	0.549343

Rotational constants (GHZ): 1.5855516 0.5720926 0.4354852

2,3-dimethylphenolate
Electronic Energy -538.441423
Stoichiometry C8H13O3(1-)
Framework group C1[X(C8H13O3)]
Deg. of freedom 66
Full point group C1 NOp 1
Largest Abelian subgroup C1 NOp 1
Largest concise Abelian subgroup C1 NOp 1

Standard orientation:

Center Number	Atomic Number	Atomic Type	Coordinates (Angstroms) X	Y	Z
1	6	0	0.152829	1.667832	-0.056207
2	6	0	0.338462	0.299978	0.226755
3	6	0	-0.802641	-0.543209	0.243332
4	6	0	-2.067740	-0.014725	-0.042078
5	6	0	-2.216196	1.344269	-0.326976
6	6	0	-1.105081	2.178434	-0.328055
7	1	0	1.022738	2.316210	-0.061234
8	1	0	-3.201111	1.740906	-0.546564
9	8	0	1.550632	-0.175377	0.473711
10	8	0	3.682881	1.340339	0.272240
11	1	0	3.407092	2.261246	0.219835
12	1	0	2.832734	0.814167	0.347945
13	8	0	2.524184	-2.234555	-0.855159
14	1	0	2.123671	-1.467907	-0.356763
15	1	0	3.266226	-1.859704	-1.340639
16	1	0	-1.220751	3.235088	-0.546157
17	6	0	-0.635938	-2.005470	0.560446
18	1	0	-0.598437	-2.617787	-0.347373
19	1	0	0.290035	-2.181206	1.106351
20	1	0	-1.463293	-2.382477	1.164481
21	6	0	-3.282786	-0.906134	-0.047670
22	1	0	-3.163485	-1.744943	-0.738855
23	1	0	-3.471549	-1.335784	0.940394
24	1	0	-4.170898	-0.346922	-0.345048

Rotational constants (GHZ): 1.3746703 0.6761481 0.4800811

2,4-dimethylphenol

Electronic Energy -538.907743

Stoichiometry C8H14O3

Framework group C1[X(C8H14O3)]

Deg. of freedom 69

Full point group C1 NOp 1

Largest Abelian subgroup C1 NOp 1

Largest concise Abelian subgroup C1 NOp 1

Standard orientation:

Center Number	Atomic Number	Atomic Type	Coordinates (Angstroms) X	Y	Z
1	6	0	0.535176	0.957472	-0.073725
2	6	0	-0.079400	-0.275041	-0.308953
3	6	0	0.666905	-1.443507	-0.348757
4	6	0	2.039280	-1.401137	-0.146857
5	6	0	2.687143	-0.194394	0.101514
6	6	0	1.913363	0.964310	0.128196
7	1	0	2.611027	-2.322046	-0.183191
8	8	0	-1.430984	-0.389442	-0.535628
9	1	0	-1.931207	0.425257	-0.338341
10	8	0	-3.705379	-1.640877	0.589181
11	1	0	-4.186204	-2.238607	0.005243
12	1	0	-2.829973	-1.527525	0.180941
13	8	0	-3.657215	1.145655	0.157791
14	1	0	-3.953072	0.230420	0.320381
15	1	0	-4.202328	1.467603	-0.569289
16	1	0	2.398731	1.918608	0.308906
17	1	0	0.162279	-2.383102	-0.541873
18	6	0	-0.261883	2.231367	-0.048330
19	1	0	-0.809997	2.379917	-0.983267
20	1	0	-0.997818	2.228826	0.760622
21	1	0	0.394544	3.088884	0.098091
22	6	0	4.169387	-0.140781	0.357212
23	1	0	4.608552	0.775073	-0.042931
24	1	0	4.385195	-0.163794	1.429663
25	1	0	4.679072	-0.991943	-0.097510

Rotational constants (GHZ): 1.9065578 0.4928341 0.4040927

2,4-dimethylphenolate
Electronic Energy -538.441994
Stoichiometry C8H13O3(1-)
Framework group C1[X(C8H13O3)]
Deg. of freedom 66
Full point group C1 NOp 1
Largest Abelian subgroup C1 NOp 1
Largest concise Abelian subgroup C1 NOp 1
Standard orientation:

Center Number	Atomic Number	Atomic Type	Coordinates (Angstroms) X	Y	Z
1	6	0	0.232082	1.318657	0.162554
2	6	0	-0.394301	0.115388	-0.197258
3	6	0	0.431412	-1.022598	-0.381506
4	6	0	1.803704	-0.908541	-0.200908
5	6	0	2.426830	0.290121	0.158248
6	6	0	1.610411	1.401037	0.334652
7	1	0	-0.384920	2.199697	0.307983
8	1	0	2.417112	-1.794027	-0.348886
9	8	0	-1.709170	0.035973	-0.364570
10	8	0	-3.120570	2.232360	-0.600363
11	1	0	-2.649558	2.939131	-0.146995
12	1	0	-2.543497	1.418966	-0.489377
13	8	0	-3.118306	-1.491664	1.265062
14	1	0	-2.568952	-0.924346	0.655022
15	1	0	-3.628948	-0.872715	1.797094
16	1	0	2.054217	2.352049	0.613696
17	6	0	-0.192354	-2.330787	-0.777702
18	1	0	-0.902905	-2.683437	-0.025091
19	1	0	-0.749989	-2.239405	-1.714528
20	1	0	0.569993	-3.100431	-0.910097
21	6	0	3.919860	0.361824	0.346784
22	1	0	4.256411	-0.296107	1.153362
23	1	0	4.455547	0.059330	-0.557502
24	1	0	4.233993	1.377340	0.595112

Rotational constants (GHZ): 1.4141500 0.5965428 0.4634363

2,6-diisopropylphenol
Electronic Energy -696.099286
Stoichiometry C12H22O3
Framework group C1[X(C12H22O3)]
Deg. of freedom 105
Full point group C1 NOp 1
Largest Abelian subgroup C1 NOp 1
Largest concise Abelian subgroup C1 NOp 1
Standard orientation:

Center Number	Atomic Number	Atomic Type	Coordinates (Angstroms) X	Y	Z
1	6	0	1.117396	-1.021888	-0.079110
2	6	0	-0.059336	-0.266160	-0.133653
3	6	0	-1.329336	-0.855185	-0.117149
4	6	0	-1.401326	-2.240179	-0.035297
5	6	0	-0.253509	-3.015761	0.023384
6	6	0	0.989566	-2.405927	-0.001396
7	1	0	-2.369126	-2.724927	-0.026571
8	1	0	-0.328227	-4.094759	0.082251
9	8	0	-0.028055	1.102016	-0.199083
10	1	0	0.856668	1.476925	-0.359194
11	8	0	-0.845787	3.742564	-0.106490
12	1	0	-1.323768	4.110059	0.640522
13	1	0	-0.937047	2.776469	-0.053148
14	8	0	1.790018	3.110565	-0.526348
15	1	0	0.979678	3.636725	-0.389644
16	1	0	2.242963	3.489411	-1.283117
17	1	0	1.879523	-3.020594	0.037492
18	6	0	-2.562322	0.028687	-0.162134
19	1	0	-2.332222	0.856323	-0.839066

20	6	0	-2.850961	0.624183	1.223221
21	1	0	-1.977828	1.140753	1.625810
22	1	0	-3.682729	1.332694	1.176710
23	1	0	-3.121409	-0.165945	1.928736
24	6	0	-3.805197	-0.671816	-0.708622
25	1	0	-4.617238	0.049475	-0.825344
26	1	0	-3.615120	-1.126653	-1.682882
27	1	0	-4.162477	-1.452961	-0.032885
28	6	0	2.473554	-0.336215	-0.057428
29	1	0	2.429318	0.529117	-0.727970
30	6	0	2.794371	0.187419	1.349601
31	1	0	3.736090	0.741235	1.350524
32	1	0	2.015256	0.854872	1.720588
33	1	0	2.885139	-0.647045	2.049844
34	6	0	3.614746	-1.212547	-0.573334
35	1	0	3.393276	-1.624325	-1.560020
36	1	0	4.529934	-0.621354	-0.649150
37	1	0	3.824061	-2.044314	0.103391

Rotational constants (GHZ): 0.5782226 0.4700602 0.2833303

2,6-diisopropylphenolate

Electronic Energy -695.632393

Stoichiometry C12H21O3(1-)

Framework group C1[X(C12H21O3)]

Deg. of freedom 102

Full point group C1 NOp 1

Largest Abelian subgroup C1 NOp 1

Largest concise Abelian subgroup C1 NOp 1

Standard orientation:

Center Number	Atomic Number	Atomic Type	Coordinates (Angstroms) X	Y	Z
1	6	0	1.399820	-0.600415	-0.013362
2	6	0	0.082038	-0.064138	0.017880
3	6	0	-1.021801	-0.944442	-0.124690
4	6	0	-0.792860	-2.308204	-0.277359
5	6	0	0.493385	-2.833560	-0.297048
6	6	0	1.575810	-1.971865	-0.167329
7	1	0	-1.635671	-2.980795	-0.388259
8	1	0	0.649718	-3.899392	-0.418974
9	8	0	-0.097854	1.234347	0.187804
10	8	0	0.590990	2.917223	-1.772933
11	1	0	-0.172067	3.499436	-1.865021
12	1	0	0.334654	2.290981	-1.043292
13	8	0	-2.004239	3.061329	0.472367
14	1	0	-1.359802	2.306497	0.411868
15	1	0	-2.726751	2.738923	1.029931
16	1	0	2.579346	-2.381849	-0.186826
17	6	0	-2.425800	-0.370712	-0.074077
18	1	0	-2.383424	0.611007	-0.551782
19	6	0	-3.469279	-1.196564	-0.824747
20	1	0	-3.642863	-2.165404	-0.349439
21	1	0	-4.424437	-0.664666	-0.836351
22	1	0	-3.173000	-1.377116	-1.861040
23	6	0	-2.875284	-0.157771	1.378406
24	1	0	-2.155879	0.443701	1.937663
25	1	0	-3.844347	0.348892	1.418662
26	1	0	-2.979063	-1.120445	1.888808
27	6	0	2.581009	0.335554	0.175328
28	1	0	2.310749	1.286785	-0.287956
29	6	0	3.870909	-0.144380	-0.487205
30	1	0	4.638714	0.630097	-0.410821
31	1	0	4.267739	-1.042829	-0.007682
32	1	0	3.720754	-0.365315	-1.547092
33	6	0	2.820536	0.608652	1.666084
34	1	0	3.618305	1.343924	1.807402
35	1	0	1.919412	0.991961	2.148884
36	1	0	3.115839	-0.310516	2.182139

Rotational constants (GHZ): 0.6370146 0.4360955 0.3096368

2-methyl-4-*terz*-butylphenol
Electronic Energy -656.802064
Stoichiometry C11H20O3
Framework group C1[X(C11H20O3)]
Deg. of freedom 96
Full point group C1 NOp 1
Largest Abelian subgroup C1 NOp 1
Largest concise Abelian subgroup C1 NOp 1
Standard orientation:

Center Number	Atomic Number	Atomic Type	Coordinates (Angstroms) X	Y	Z
1	6	0	0.990122	-1.380127	-0.304095
2	6	0	-0.389534	-1.429986	-0.467179
3	6	0	-1.145936	-0.273619	-0.385728
4	6	0	-0.532753	0.959124	-0.143676
5	6	0	0.848504	0.972817	0.018135
6	6	0	1.641974	-0.175734	-0.054362
7	1	0	1.546643	-2.304953	-0.373950
8	1	0	-0.888599	-2.372966	-0.658695
9	1	0	1.316749	1.932745	0.206672
10	8	0	-2.502825	-0.396019	-0.571712
11	1	0	-3.003116	0.412395	-0.350452
12	8	0	-4.724068	1.109132	0.208009
13	1	0	-5.305495	1.398726	-0.504344
14	1	0	-4.989400	0.191196	0.403746
15	8	0	-4.679814	-1.670238	0.711090
16	1	0	-3.827045	-1.542107	0.261221
17	1	0	-5.167787	-2.294381	0.161717
18	6	0	3.158347	-0.069084	0.138714
19	6	0	3.748940	0.870541	-0.924586
20	1	0	3.324329	1.874154	-0.856688
21	1	0	4.831170	0.954892	-0.792333
22	1	0	3.559384	0.489068	-1.931401
23	6	0	3.459466	0.499907	1.534456
24	1	0	3.058532	-0.150909	2.316023
25	1	0	4.539844	0.581662	1.682719
26	1	0	3.026973	1.493786	1.666185
27	6	0	3.852431	-1.427951	0.015403
28	1	0	3.498290	-2.135309	0.769157
29	1	0	3.699773	-1.874447	-0.970150
30	1	0	4.927798	-1.299229	0.159475
31	6	0	-1.338758	2.225486	-0.065739
32	1	0	-1.918142	2.388539	-0.979192
33	1	0	-2.048172	2.200264	0.766251
34	1	0	-0.684884	3.085633	0.076900

Rotational constants (GHZ): 1.3473956 0.2736855 0.2449341

2-methyl-4-*terz*-butylphenolate
Electronic Energy -656.336205
Stoichiometry C11H19O3(1-)
Framework group C1[X(C11H19O3)]
Deg. of freedom 93
Full point group C1 NOp 1
Largest Abelian subgroup C1 NOp 1
Largest concise Abelian subgroup C1 NOp 1
Standard orientation:

Center Number	Atomic Number	Atomic Type	Coordinates (Angstroms) X	Y	Z
1	6	0	-0.759257	1.037085	0.238464
2	6	0	0.618102	1.106572	0.395219
3	6	0	1.390266	-0.080605	0.329295
4	6	0	0.701369	-1.280897	0.104921
5	6	0	-0.682182	-1.317526	-0.047801
6	6	0	-1.451800	-0.159840	0.012989
7	1	0	-1.315867	1.967594	0.296943
8	1	0	1.271708	-2.202930	0.051586
9	1	0	-1.150882	-2.278860	-0.218004

10	8	0	2.708732	-0.046550	0.479140
11	8	0	4.132363	-2.245467	0.311679
12	1	0	3.546947	-2.964695	0.052872
13	1	0	3.542153	-1.436845	0.373193
14	8	0	4.216650	1.218949	-1.285527
15	1	0	3.623630	0.763322	-0.625909
16	1	0	4.815316	0.533066	-1.599030
17	6	0	-2.975940	-0.148480	-0.148707
18	6	0	-3.630353	0.416801	1.122472
19	1	0	-3.299766	1.437636	1.325259
20	1	0	-4.718893	0.432645	1.014810
21	1	0	-3.385212	-0.197202	1.993292
22	6	0	-3.366231	0.735768	-1.344183
23	1	0	-2.930377	0.352901	-2.270939
24	1	0	-4.453436	0.754992	-1.463986
25	1	0	-3.026277	1.764974	-1.211323
26	6	0	-3.540393	-1.551133	-0.390474
27	1	0	-3.137173	-1.999923	-1.301568
28	1	0	-3.327104	-2.224247	0.443481
29	1	0	-4.626114	-1.492654	-0.501008
30	6	0	1.303620	2.421377	0.640909
31	1	0	1.872663	2.406797	1.575233
32	1	0	2.016158	2.659322	-0.153995
33	1	0	0.577361	3.233930	0.698124

Rotational constants (GHZ): 1.1197465 0.3119300 0.2710460

4-bromothymol

Electronic Energy -3191.151201

Stoichiometry C10H17BrO3

Framework group C1[X(C10H17BrO3)]

Deg. of freedom 87

Full point group C1 NOp 1

Largest Abelian subgroup C1 NOp 1

Largest concise Abelian subgroup C1 NOp 1

Standard orientation:

Center Number	Atomic Number	Atomic Type	Coordinates (Angstroms) X	Y	Z
1	6	0	-0.467162	-1.409828	-0.371235
2	6	0	-1.198622	-0.230875	-0.312842
3	6	0	-0.570411	1.000055	-0.089699
4	6	0	0.810343	0.986107	0.064824
5	6	0	1.531301	-0.197437	0.000810
6	6	0	0.917611	-1.426236	-0.217659
7	1	0	-0.989562	-2.344684	-0.542727
8	1	0	1.336562	1.914539	0.240530
9	8	0	-2.554620	-0.239417	-0.471170
10	1	0	-2.897647	-1.153052	-0.590717
11	6	0	-1.384759	2.279094	-0.060333
12	1	0	-2.337472	2.035595	0.417798
13	6	0	-1.690199	2.752777	-1.486751
14	1	0	-2.189083	1.977199	-2.070811
15	1	0	-2.338920	3.632378	-1.467339
16	1	0	-0.765875	3.025775	-2.003895
17	6	0	-0.737203	3.402351	0.745530
18	1	0	-1.427614	4.245599	0.819991
19	1	0	-0.486382	3.081335	1.759296
20	1	0	0.175161	3.770065	0.269154
21	6	0	1.669788	-2.722821	-0.289706
22	1	0	2.210882	-2.920637	0.638455
23	1	0	0.983754	-3.550564	-0.468027
24	1	0	2.407326	-2.709605	-1.095497
25	8	0	-4.706642	-0.692025	1.516195
26	1	0	-3.939942	-0.221121	1.154912
27	1	0	-4.504280	-0.832791	2.448543
28	8	0	-3.940549	-2.587283	-0.387446
29	1	0	-4.590240	-2.735724	-1.084465
30	1	0	-4.417854	-2.122140	0.326421
31	35	0	3.431473	-0.101185	0.231146

Rotational constants (GHZ): 0.6272509 0.2499845 0.1938929

4-bromothymolate
Electronic Energy -3190.685396
Stoichiometry C10H16BrO3(1-)
Framework group C1[X(C10H16BrO3)]
Deg. of freedom 84
Full point group C1 NOp 1
Largest Abelian subgroup C1 NOp 1
Largest concise Abelian subgroup C1 NOp 1
Standard orientation:

Center Number	Atomic Number	Atomic Type	Coordinates (Angstroms) X	Y	Z
1	6	0	1.711932	1.915457	-0.157654
2	1	0	2.556138	1.599423	-0.775886
3	6	0	0.729630	0.760895	-0.141535
4	6	0	-0.640866	0.967805	-0.073935
5	6	0	1.217178	-0.574071	-0.159529
6	6	0	-1.537182	-0.091542	-0.024700
7	1	0	-1.026411	1.979175	-0.061177
8	6	0	0.272488	-1.618038	-0.115162
9	6	0	-1.102643	-1.412950	-0.046280
10	1	0	0.644389	-2.637541	-0.137126
11	6	0	2.249604	2.186372	1.253263
12	1	0	3.031579	2.950671	1.232338
13	1	0	1.445461	2.544565	1.903273
14	1	0	2.670301	1.284863	1.702441
15	6	0	1.154596	3.201324	-0.765049
16	1	0	1.947736	3.949207	-0.842356
17	1	0	0.751729	3.033547	-1.766980
18	1	0	0.360966	3.632783	-0.149315
19	6	0	-2.041209	-2.585355	-0.003624
20	1	0	-2.732623	-2.574056	-0.849752
21	1	0	-1.482636	-3.521126	-0.032754
22	1	0	-2.648427	-2.578485	0.904818
23	8	0	2.504822	-0.845603	-0.223343
24	8	0	3.439457	-3.279685	0.120881
25	1	0	2.710208	-3.904229	0.049375
26	1	0	3.026182	-2.378519	-0.014766
27	8	0	4.892027	0.320376	-0.129314
28	1	0	3.965313	-0.033277	-0.152421
29	1	0	5.453893	-0.457722	-0.051755
30	35	0	-3.412726	0.315732	0.065757

Rotational constants (GHZ): 0.6895737 0.2564481 0.1930398

4-chlorothymol
Electronic Energy -1077.133037
Stoichiometry C10H17ClO3
Framework group C1[X(C10H17ClO3)]
Deg. of freedom 87
Full point group C1 NOp 1
Largest Abelian subgroup C1 NOp 1
Largest concise Abelian subgroup C1 NOp 1
Standard orientation:

Center Number	Atomic Number	Atomic Type	Coordinates (Angstroms) X	Y	Z
1	6	0	0.058158	-1.426434	-0.334392
2	6	0	-0.650727	-0.232568	-0.299024
3	6	0	-0.005852	0.986136	-0.056485
4	6	0	1.368819	0.945871	0.140679
5	6	0	2.065090	-0.252000	0.098900
6	6	0	1.436205	-1.468701	-0.137548
7	1	0	-0.476800	-2.350991	-0.521875
8	1	0	1.913421	1.860563	0.333073
9	8	0	-2.001473	-0.214288	-0.499698
10	1	0	-2.358165	-1.121579	-0.625570
11	6	0	-0.796081	2.280376	-0.051625

12	1	0	-1.767751	2.055023	0.396269
13	6	0	-1.047732	2.759868	-1.486607
14	1	0	-1.543088	1.993855	-2.086110
15	1	0	-1.679605	3.651858	-1.486850
16	1	0	-0.102674	3.014874	-1.974616
17	6	0	-0.152472	3.390630	0.775092
18	1	0	-0.828660	4.246862	0.829159
19	1	0	0.060653	3.064278	1.795736
20	1	0	0.781002	3.741219	0.327567
21	6	0	2.176547	-2.772905	-0.182571
22	1	0	2.686800	-2.971939	0.762809
23	1	0	1.488088	-3.594339	-0.379436
24	1	0	2.938721	-2.768856	-0.965392
25	8	0	-4.199541	-0.615666	1.444133
26	1	0	-3.416779	-0.161293	1.096194
27	1	0	-4.014253	-0.764892	2.378727
28	8	0	-3.440012	-2.533058	-0.437955
29	1	0	-4.077822	-2.666888	-1.148743
30	1	0	-3.920021	-2.050409	0.262528
31	17	0	3.807848	-0.207935	0.362128

Rotational constants (GHZ): 0.6307636 0.3539315 0.2509656

4-chlorothymolate

Electronic Energy -1076.667182

Stoichiometry C10H16ClO3(1-)

Framework group C1[X(C10H16ClO3)]

Deg. of freedom 84

Full point group C1 NOp 1

Largest Abelian subgroup C1 NOp 1

Largest concise Abelian subgroup C1 NOp 1

Standard orientation:

Center Number	Atomic Number	Atomic Type	Coordinates (Angstroms) X	Y	Z
1	6	0	0.392473	-1.535816	-0.126366
2	6	0	-0.627560	-0.565854	-0.186524
3	6	0	-0.244536	0.802339	-0.150893
4	6	0	1.104147	1.113727	-0.050481
5	6	0	2.074467	0.124412	0.013653
6	6	0	1.744982	-1.225672	-0.024550
7	1	0	0.099787	-2.580390	-0.161779
8	1	0	1.416706	2.150079	-0.021339
9	8	0	-1.889395	-0.934257	-0.280363
10	6	0	-1.310650	1.879396	-0.182161
11	1	0	-2.130053	1.491052	-0.792325
12	6	0	-0.850305	3.192505	-0.812598
13	1	0	-0.439165	3.037407	-1.813199
14	1	0	-1.695672	3.879762	-0.898892
15	1	0	-0.088180	3.690386	-0.207378
16	6	0	-1.863739	2.132461	1.226022
17	1	0	-2.700648	2.835867	1.195694
18	1	0	-2.215019	1.209285	1.690769
19	1	0	-1.087177	2.559734	1.867746
20	6	0	2.779377	-2.313172	0.036580
21	1	0	2.304698	-3.293387	-0.009857
22	1	0	3.487478	-2.236982	-0.792338
23	1	0	3.361040	-2.258837	0.960140
24	8	0	-2.635100	-3.429411	0.076723
25	1	0	-1.904350	-4.007595	-0.165618
26	1	0	-2.292762	-2.500064	-0.067712
27	8	0	-4.367374	-0.011433	-0.000914
28	1	0	-3.411820	-0.263773	-0.083406
29	1	0	-4.835190	-0.847220	0.099184
30	17	0	3.769021	0.618208	0.140038

Rotational constants (GHZ): 0.6955452 0.3651764 0.2497751

4-methoxyphenol

Electronic Energy -574.815819

Stoichiometry C7H12O4

Framework group C1[X(C7H12O4)]
Deg. of freedom 63
Full point group C1 NOp 1
Largest Abelian subgroup C1 NOp 1
Largest concise Abelian subgroup C1 NOp 1
Standard orientation:

Center Number	Atomic Number	Atomic Type	Coordinates (Angstroms) X	Y	Z
1	6	0	1.979785	1.084762	-0.581321
2	6	0	0.755870	1.723665	-0.450266
3	6	0	-0.362542	1.009375	-0.030180
4	6	0	-0.249658	-0.348699	0.254825
5	6	0	0.978323	-0.983970	0.122805
6	6	0	2.089625	-0.268577	-0.294927
7	1	0	2.853648	1.634403	-0.910824
8	1	0	0.657067	2.778961	-0.674804
9	1	0	-1.120867	-0.906728	0.576237
10	1	0	1.073327	-2.041427	0.339938
11	8	0	-1.546775	1.683439	0.080941
12	1	0	-2.271362	1.096877	0.409425
13	8	0	-3.611058	0.244743	1.027204
14	1	0	-4.315388	0.855146	1.274779
15	1	0	-4.015087	-0.380096	0.386633
16	8	0	-4.725277	-1.505583	-0.773318
17	1	0	-4.082654	-1.870917	-1.393704
18	1	0	-5.125714	-2.273279	-0.347246
19	8	0	3.307795	-0.915996	-0.463653
20	6	0	4.127457	-0.913828	0.708370
21	1	0	5.043596	-1.444956	0.454450
22	1	0	4.369089	0.109619	1.007790
23	1	0	3.623702	-1.426796	1.532103

Rotational constants (GHZ): 1.8994457 0.3857899 0.3441344
Standard basis: 6-311+G(d,p) (5D, 7F)

4-methoxyphenolate
Electronic Energy -574.349421
Stoichiometry C7H11O4(1-)
Framework group C1[X(C7H11O4)]
Deg. of freedom 60
Full point group C1 NOp 1
Largest Abelian subgroup C1 NOp 1
Largest concise Abelian subgroup C1 NOp 1
Standard orientation:

Center Number	Atomic Number	Atomic Type	Coordinates (Angstroms) X	Y	Z
1	6	0	1.295493	-1.203381	-0.028668
2	6	0	-0.084971	-1.193704	0.108703
3	6	0	-0.830309	-0.004713	-0.030264
4	6	0	-0.107581	1.171237	-0.316021
5	6	0	1.274076	1.157921	-0.448268
6	6	0	1.979024	-0.028118	-0.306829
7	1	0	1.849242	-2.130111	0.077300
8	1	0	-0.612809	-2.116057	0.324363
9	1	0	-0.652334	2.101126	-0.435794
10	1	0	1.812041	2.073334	-0.670113
11	8	0	-2.146612	0.004724	0.099216
12	8	0	-3.551325	2.225260	0.214473
13	1	0	-3.797456	2.333445	1.138784
14	1	0	-2.982963	1.402788	0.183457
15	8	0	-3.586318	-2.194661	0.020565
16	1	0	-3.006299	-1.379977	0.031858
17	1	0	-3.881818	-2.287247	-0.890865
18	8	0	3.363301	-0.042661	-0.481941
19	6	0	4.098410	0.064912	0.737577
20	1	0	3.870502	-0.771637	1.404060
21	1	0	5.155783	0.041631	0.476180
22	1	0	3.868904	1.006486	1.244881

Rotational constants (GHZ): 1.8043916 0.4620680 0.3803542

4-hydroxybenzonitrile
Electronic Energy -552.540660
Stoichiometry C7H9NO3
Framework group C1[X(C7H9NO3)]
Deg. of freedom 54
Full point group C1 NOp 1
Largest Abelian subgroup C1 NOp 1
Largest concise Abelian subgroup C1 NOp 1
Standard orientation:

Center Number	Atomic Number	Atomic Type	Coordinates (Angstroms) X	Y	Z
1	6	0	-2.144856	1.073756	0.457987
2	6	0	-0.903539	1.674283	0.458246
3	6	0	0.213892	0.971693	0.001947
4	6	0	0.075777	-0.340043	-0.458334
5	6	0	-1.167555	-0.941210	-0.458014
6	6	0	-2.285868	-0.240172	-0.000178
7	1	0	-3.011865	1.617118	0.811948
8	1	0	-0.780294	2.690813	0.810181
9	1	0	0.944708	-0.880415	-0.811934
10	1	0	-1.277100	-1.958400	-0.812466
11	8	0	1.405725	1.604842	0.027766
12	1	0	2.143287	1.037897	-0.321628
13	8	0	3.468775	0.228121	-0.928543
14	1	0	4.162600	0.850398	-1.176687
15	1	0	3.882269	-0.389406	-0.285963
16	8	0	4.607469	-1.495744	0.874725
17	1	0	3.970993	-1.862170	1.500797
18	1	0	5.013038	-2.262920	0.452563
19	6	0	-3.566889	-0.862048	0.001305
20	7	0	-4.604165	-1.365462	0.003407

Rotational constants (GHZ): 2.0054793 0.3916555 0.3469310

4-cyanophenolate
Electronic Energy -552.079872
Stoichiometry C7H8NO3(1-)
Framework group C1[X(C7H8NO3)]
Deg. of freedom 51
Full point group C1 NOp 1
Largest Abelian subgroup C1 NOp 1
Largest concise Abelian subgroup C1 NOp 1
Standard orientation:

Center Number	Atomic Number	Atomic Type	Coordinates (Angstroms) X	Y	Z
1	6	0	1.443045	1.207033	-0.075779
2	6	0	0.067449	1.209225	-0.099650
3	6	0	-0.674995	0.001337	-0.045049
4	6	0	0.063361	-1.207639	0.034858
5	6	0	1.438950	-1.207484	0.057703
6	6	0	2.151882	-0.000774	0.002955
7	1	0	1.987708	2.143000	-0.118771
8	1	0	-0.473943	2.145885	-0.162034
9	1	0	-0.481248	-2.143512	0.078022
10	1	0	1.980406	-2.144321	0.118527
11	8	0	-1.975363	0.002309	-0.068295
12	8	0	-3.433498	-2.222423	0.060192
13	1	0	-4.015075	-2.190927	-0.706394
14	1	0	-2.862814	-1.411844	-0.007751
15	8	0	-3.434164	2.227576	0.036500
16	1	0	-2.862039	1.415580	0.015865
17	1	0	-3.958423	2.151131	0.840335
18	6	0	3.569560	-0.002086	0.025866
19	7	0	4.724874	-0.003195	0.044228

Rotational constants (GHZ): 1.9275122 0.4705500 0.3791892

4-nitrophenol
Electronic Energy -664.816544
Stoichiometry C6H9NO5
Framework group C1[X(C6H9NO5)]
Deg. of freedom 57
Full point group C1 NOp 1
Largest Abelian subgroup C1 NOp 1
Largest concise Abelian subgroup C1 NOp 1
Standard orientation:

Center Number	Atomic Number	Atomic Type	Coordinates (Angstroms) X	Y	Z
1	6	0	-1.686175	1.329628	-0.020169
2	6	0	-0.356170	1.670889	0.073217
3	6	0	0.620776	0.672847	0.164219
4	6	0	0.247072	-0.676525	0.160519
5	6	0	-1.083939	-1.020933	0.068136
6	6	0	-2.041695	-0.016512	-0.022217
7	1	0	-2.446467	2.094462	-0.091458
8	1	0	-0.050549	2.709172	0.077384
9	1	0	1.005685	-1.445788	0.229452
10	1	0	-1.381708	-2.059814	0.064326
11	8	0	1.902412	1.060420	0.252303
12	1	0	2.530902	0.288452	0.311243
13	8	0	3.664201	-0.896115	0.451611
14	1	0	3.382793	-1.693567	-0.012436
15	1	0	4.538474	-0.656580	0.071318
16	8	0	6.099436	-0.191015	-0.584685
17	1	0	6.194069	0.765903	-0.666932
18	1	0	6.831939	-0.467704	-0.020526
19	7	0	-3.434979	-0.378478	-0.121809
20	8	0	-4.272078	0.512266	-0.209634
21	8	0	-3.738908	-1.565751	-0.116087

Rotational constants (GHZ): 2.6655742 0.2812469 0.2575819

4-nitrophenolate
Electronic Energy -664.359307
Stoichiometry C6H8NO5(1-)
Framework group C1[X(C6H8NO5)]
Deg. of freedom 54
Full point group C1 NOp 1
Largest Abelian subgroup C1 NOp 1
Largest concise Abelian subgroup C1 NOp 1
Standard orientation:

Center Number	Atomic Number	Atomic Type	Coordinates (Angstroms) X	Y	Z
1	6	0	1.034805	1.216112	-0.051315
2	6	0	-0.333265	1.216570	-0.051873
3	6	0	-1.077238	-0.000001	-0.000747
4	6	0	-0.333305	-1.216582	0.050721
5	6	0	1.034765	-1.216140	0.050820
6	6	0	1.732020	-0.000019	-0.000077
7	1	0	1.583246	2.147252	-0.090802
8	1	0	-0.879760	2.150865	-0.091976
9	1	0	-0.879832	-2.150870	0.090554
10	1	0	1.583175	-2.147287	0.090556
11	8	0	-2.362682	0.000013	-0.001047
12	8	0	-3.848863	-2.240742	0.013147
13	1	0	-4.401111	-2.186819	-0.773692
14	1	0	-3.275326	-1.435400	-0.013281
15	8	0	-3.848689	2.240908	-0.011546
16	1	0	-3.275332	1.435400	0.013613
17	1	0	-4.400184	2.186427	0.775780
18	7	0	3.145358	-0.000027	0.000275
19	8	0	3.753941	1.075668	-0.040649
20	8	0	3.753909	-1.075725	0.041612

Rotational constants (GHZ): 1.6706719 0.3892465 0.3162489

4-nitrothymol
Electronic Energy -822.012637
Stoichiometry C10H17NO5
Framework group C1[X(C10H17NO5)]
Deg. of freedom 93
Full point group C1 NOp 1
Largest Abelian subgroup C1 NOp 1
Largest concise Abelian subgroup C1 NOp 1
Standard orientation:

Center Number	Atomic Number	Atomic Type	Coordinates (Angstroms) X	Y	Z
1	6	0	-1.218002	0.958446	-0.067063
2	6	0	0.133892	1.008263	-0.322872
3	6	0	0.799634	-0.225446	-0.455180
4	6	0	0.115006	-1.431385	-0.327555
5	6	0	-1.249169	-1.491375	-0.080801
6	6	0	-1.900625	-0.255302	0.045296
7	1	0	-1.784040	1.872162	0.037220
8	1	0	0.671790	-2.355754	-0.423149
9	8	0	2.119293	-0.199809	-0.712388
10	1	0	2.517688	-1.105175	-0.741720
11	8	0	3.551471	-2.461816	-0.536261
12	1	0	4.115526	-2.646412	-1.296327
13	1	0	4.112570	-1.976565	0.103388
14	8	0	4.640413	-0.635780	1.208611
15	1	0	3.875870	-0.057677	1.081857
16	1	0	4.669654	-0.813768	2.156259
17	7	0	-3.318273	-0.170963	0.303353
18	8	0	-3.779793	0.883630	0.733046
19	8	0	-4.027972	-1.145032	0.083122
20	6	0	0.908888	2.305758	-0.432648
21	1	0	1.649359	2.165545	-1.224508
22	6	0	1.668975	2.587376	0.869853
23	1	0	2.325964	1.759223	1.143914
24	1	0	2.283074	3.485230	0.764662
25	1	0	0.967196	2.751705	1.692354
26	6	0	0.042814	3.503381	-0.812514
27	1	0	-0.526529	3.317813	-1.726285
28	1	0	-0.661563	3.762815	-0.018014
29	1	0	0.678163	4.375353	-0.982166
30	6	0	-1.890387	-2.842034	0.056036
31	1	0	-2.499121	-2.910992	0.958250
32	1	0	-2.546044	-3.055794	-0.789279
33	1	0	-1.115093	-3.606619	0.095720

Rotational constants (GHZ): 0.6177707 0.3091843 0.2224958

4-nitrothymolate
Electronic Energy -821.555067
Stoichiometry C10H16NO5(1-)
Framework group C1[X(C10H16NO5)]
Deg. of freedom 90
Full point group C1 NOp 1
Largest Abelian subgroup C1 NOp 1
Largest concise Abelian subgroup C1 NOp 1
Standard orientation:

Center Number	Atomic Number	Atomic Type	Coordinates (Angstroms) X	Y	Z
1	6	0	-0.956370	1.036747	-0.067584
2	6	0	0.388969	0.801333	-0.098718
3	6	0	0.838399	-0.564586	-0.086580
4	6	0	-0.146730	-1.587980	-0.039747
5	6	0	-1.503028	-1.357808	-0.004032
6	6	0	-1.912378	0.000499	-0.018380
7	1	0	-1.323470	2.052095	-0.081537

8	1	0	0.210475	-2.611721	-0.034701
9	8	0	2.086084	-0.866607	-0.122533
10	8	0	3.029545	-3.367321	0.074227
11	1	0	2.293932	-3.984884	0.004138
12	1	0	2.619502	-2.469064	-0.000876
13	8	0	4.579299	0.170081	-0.309701
14	1	0	3.638535	-0.110511	-0.242184
15	1	0	5.074831	-0.651234	-0.397509
16	7	0	-3.269169	0.385314	0.009773
17	8	0	-3.565235	1.591991	-0.015997
18	8	0	-4.160755	-0.471575	0.061018
19	6	0	1.416142	1.913358	-0.117818
20	1	0	2.225678	1.580137	-0.772926
21	6	0	2.010147	2.122273	1.281199
22	1	0	2.406546	1.193379	1.694605
23	1	0	2.822914	2.852692	1.249471
24	1	0	1.244135	2.498872	1.965473
25	6	0	0.887886	3.234615	-0.670102
26	1	0	0.450455	3.111974	-1.663872
27	1	0	0.128846	3.673610	-0.017084
28	1	0	1.705462	3.955080	-0.748251
29	6	0	-2.421865	-2.545429	0.041734
30	1	0	-3.043197	-2.537673	0.938223
31	1	0	-3.100586	-2.560987	-0.811985
32	1	0	-1.828405	-3.459647	0.034651

Rotational constants (GHZ): 0.6518717 0.3224676 0.2236825

- 2H_2O/CAM-B3LYP 6-311G+dp/PCM

H_2O

Electronic Energy -229.329930
Stoichiometry H6O3
Framework group C1[X(H6O3)]
Deg. of freedom 21
Full point group C1 NOp 1
Largest Abelian subgroup C1 NOp 1
Largest concise Abelian subgroup C1 NOp 1
Standard orientation:

Center Number	Atomic Number	Atomic Type	Coordinates (Angstroms) X	Y	Z
1	8	0	0.000027	0.771636	-0.000040
2	1	0	0.000772	1.353128	0.768172
3	1	0	-0.001205	1.353830	-0.767718
4	8	0	-2.657232	-0.369073	-0.000223
5	1	0	-2.720716	-1.326416	0.001483
6	1	0	-1.713112	-0.161121	0.000730
7	8	0	2.657268	-0.369069	0.000209
8	1	0	2.720574	-1.326425	-0.001593
9	1	0	1.713184	-0.160946	-0.000632

Rotational constants (GHZ): 22.6022816 2.0386116 1.8865469

OH^-

Electronic Energy -152.393647
Stoichiometry H3O2(1-)
Framework group C1[X(H3O2)]
Deg. of freedom 9
Full point group C1 NOp 1
Largest Abelian subgroup C1 NOp 1
Largest concise Abelian subgroup C1 NOp 1
Standard orientation:

Center Number	Atomic Number	Atomic Type	Coordinates (Angstroms) X	Y	Z
1	8	0	1.225669	0.098967	-0.058420
2	1	0	1.577600	-0.615783	0.477436

3	8	0	-1.224967	-0.103198	-0.046754
4	1	0	-1.479939	0.685116	0.436534
5	1	0	-0.103272	-0.035486	-0.072574

Rotational constants (GHZ): 314.8359149 9.4768812 9.3693157

Phenol

Electronic Energy -460.298191

Stoichiometry C6H10O3

Framework group C1[X(C6H10O3)]

Deg. of freedom 51

Full point group C1 NOp 1

Largest Abelian subgroup C1 NOp 1

Largest concise Abelian subgroup C1 NOp 1

Standard orientation:

Center Number	Atomic Number	Atomic Type	Coordinates (Angstroms) X	Y	Z
1	6	0	2.579260	-1.016676	-0.040705
2	6	0	1.253485	-1.237462	-0.379970
3	6	0	0.346478	-0.185920	-0.328551
4	6	0	0.765128	1.081408	0.061626
5	6	0	2.096200	1.289930	0.395107
6	6	0	3.009913	0.246168	0.347385
7	1	0	3.283009	-1.839665	-0.083224
8	1	0	0.907538	-2.215265	-0.691722
9	1	0	0.046779	1.892161	0.109070
10	1	0	2.417832	2.279297	0.698418
11	1	0	4.046814	0.414137	0.609927
12	8	0	-0.950101	-0.442795	-0.665179
13	1	0	-1.485870	0.376792	-0.670092
14	8	0	-2.896718	1.394032	-0.243515
15	1	0	-3.524160	1.828118	-0.825832
16	1	0	-3.367944	0.667279	0.203110
17	8	0	-3.222605	-1.107456	0.798023
18	1	0	-2.363787	-1.283333	0.378914
19	1	0	-3.707608	-1.934460	0.827446

Rotational constants (GHZ): 2.8958025 0.7313665 0.6242864

Phenolate

Electronic Energy -459.831866

Stoichiometry C6H9O3(1-)

Framework group C1[X(C6H9O3)]

Deg. of freedom 48

Full point group C1 NOp 1

Largest Abelian subgroup C1 NOp 1

Largest concise Abelian subgroup C1 NOp 1

Standard orientation:

Center Number	Atomic Number	Atomic Type	Coordinates (Angstroms) X	Y	Z
1	6	0	2.239783	-1.191366	0.089534
2	6	0	0.855874	-1.200725	0.090661
3	6	0	0.103276	-0.000014	0.000051
4	6	0	0.855928	1.200660	-0.090622
5	6	0	2.239837	1.191230	-0.089575
6	6	0	2.957691	-0.000086	-0.000040
7	1	0	2.773761	-2.135488	0.160346
8	1	0	0.307361	-2.134174	0.159615
9	1	0	0.307455	2.134133	-0.159578
10	1	0	2.773857	2.135324	-0.160435
11	1	0	4.041828	-0.000113	-0.000074
12	8	0	-1.191149	0.000017	0.000105
13	8	0	-2.824764	2.092553	0.032464
14	1	0	-3.337591	1.846389	0.804705
15	1	0	-2.156604	1.355543	-0.039445
16	8	0	-2.825152	-2.092399	-0.032578
17	1	0	-2.156952	-1.355406	0.038921
18	1	0	-3.338927	-1.845773	-0.804040

Rotational constants (GHZ): 2.1100979 0.8611089 0.6142565

Thymol

Electronic Energy -617.499633
Stoichiometry C10H18O3
Framework group C1[X(C10H18O3)]
Deg. of freedom 87
Full point group C1 NOp 1
Largest Abelian subgroup C1 NOp 1
Largest concise Abelian subgroup C1 NOp 1

Standard orientation:

Center Number	Atomic Number	Atomic Type	Coordinates (Angstroms) X	Y	Z
1	6	0	-0.158235	2.186971	-0.296881
2	1	0	-0.954110	1.966721	-1.013079
3	6	0	0.659901	0.918621	-0.151881
4	6	0	2.022711	0.916996	0.116129
5	6	0	0.035212	-0.329051	-0.256104
6	6	0	2.743388	-0.261066	0.272971
7	1	0	2.546027	1.861523	0.201744
8	6	0	0.746618	-1.510650	-0.102352
9	6	0	2.112986	-1.493599	0.161881
10	1	0	3.806330	-0.216292	0.481160
11	1	0	0.218718	-2.455973	-0.185447
12	6	0	0.638913	3.371957	-0.840523
13	1	0	-0.028266	4.215831	-1.030955
14	1	0	1.396041	3.713844	-0.130238
15	1	0	1.141948	3.120420	-1.776404
16	6	0	-0.824424	2.555201	1.035730
17	1	0	-1.485725	3.417699	0.917184
18	1	0	-1.413819	1.724340	1.428764
19	1	0	-0.069398	2.806814	1.785497
20	6	0	2.877844	-2.782596	0.301248
21	1	0	2.335678	-3.501958	0.918543
22	1	0	3.042434	-3.247875	-0.674687
23	1	0	3.854635	-2.614253	0.756614
24	8	0	-1.307457	-0.353020	-0.514643
25	1	0	-1.628633	-1.267484	-0.650891
26	8	0	-3.855804	-0.307367	0.617610
27	1	0	-2.982706	0.057343	0.395515
28	1	0	-4.086027	0.019953	1.490038
29	8	0	-2.840471	-2.590422	-0.492679
30	1	0	-3.317975	-3.019810	-1.206359
31	1	0	-3.474773	-2.011088	-0.030611

Rotational constants (GHZ): 0.6896704 0.5733312 0.3373845

Thymolate

Electronic Energy -617.037656
Stoichiometry C10H17O3(1-)
Framework group C1[X(C10H17O3)]
Deg. of freedom 84
Full point group C1 NOp 1
Largest Abelian subgroup C1 NOp 1
Largest concise Abelian subgroup C1 NOp 1

Standard orientation:

Center Number	Atomic Number	Atomic Type	Coordinates (Angstroms) X	Y	Z
1	6	0	-1.509154	-1.429087	-0.162357
2	1	0	-2.062035	-0.684207	-0.741028
3	6	0	-0.068396	-0.958252	-0.100830
4	6	0	1.003470	-1.840502	-0.138587
5	6	0	0.204367	0.432195	0.034743
6	6	0	2.328943	-1.417316	-0.049766
7	1	0	0.808856	-2.902389	-0.240386
8	6	0	1.550460	0.837524	0.119323
9	6	0	2.612351	-0.060731	0.077829

10	1	0	3.134860	-2.142432	-0.081762
11	1	0	1.751585	1.899791	0.224055
12	6	0	-2.129570	-1.472300	1.240286
13	1	0	-3.196248	-1.709020	1.188929
14	1	0	-1.642970	-2.242627	1.846520
15	1	0	-2.020540	-0.517521	1.757746
16	6	0	-1.698036	-2.775886	-0.858027
17	1	0	-2.764024	-2.997531	-0.954282
18	1	0	-1.262699	-2.778586	-1.860479
19	1	0	-1.246090	-3.594428	-0.291723
20	6	0	4.033448	0.434346	0.144720
21	1	0	4.351191	0.847871	-0.817201
22	1	0	4.145518	1.226100	0.888727
23	1	0	4.721799	-0.373691	0.398771
24	8	0	-0.763856	1.330041	0.081953
25	8	0	-0.382076	3.907055	-0.229604
26	1	0	0.032666	4.047945	-1.086903
27	1	0	-0.461632	2.913423	-0.129809
28	8	0	-3.399098	1.582521	-0.084703
29	1	0	-2.425100	1.397558	-0.024046
30	1	0	-3.472198	2.552860	-0.062300

Rotational constants (GHZ): 0.7435371 0.5832709 0.3473228

Carvacrol

Electronic Energy -617.500163

Stoichiometry C10H18O3

Framework group C1[X(C10H18O3)]

Deg. of freedom 87

Full point group C1 NOp 1

Largest Abelian subgroup C1 NOp 1

Largest concise Abelian subgroup C1 NOp 1

Standard orientation:

Center Number	Atomic Number	Atomic Type	Coordinates (Angstroms) X	Y	Z
1	6	0	-0.592706	1.517328	0.025200
2	6	0	-0.663353	0.146195	-0.225444
3	6	0	0.483202	-0.640551	-0.254854
4	6	0	1.739752	-0.091511	-0.031609
5	6	0	1.820967	1.277349	0.224687
6	6	0	0.672927	2.055094	0.250246
7	1	0	2.781978	1.744387	0.405354
8	8	0	-1.849404	-0.487241	-0.469724
9	1	0	-2.639655	0.067380	-0.313123
10	8	0	-3.585143	-2.533214	0.320034
11	1	0	-3.749081	-3.306691	-0.228103
12	1	0	-2.749300	-2.147902	0.008812
13	8	0	-4.436575	0.084092	0.116617
14	1	0	-4.484734	-0.888665	0.173723
15	1	0	-5.127580	0.360158	-0.493146
16	1	0	0.754893	3.118524	0.448742
17	1	0	0.374164	-1.700767	-0.455983
18	6	0	-1.828893	2.374453	0.041192
19	1	0	-2.337360	2.366125	-0.927655
20	1	0	-2.553042	2.035640	0.786664
21	1	0	-1.572803	3.408362	0.271316
22	6	0	2.977020	-0.968259	-0.067330
23	1	0	2.640558	-1.986556	-0.284379
24	6	0	3.937488	-0.548809	-1.184724
25	1	0	4.790634	-1.230139	-1.231143
26	1	0	3.441247	-0.558554	-2.157585
27	1	0	4.324546	0.459027	-1.013946
28	6	0	3.693097	-0.994970	1.286720
29	1	0	3.021730	-1.323419	2.083077
30	1	0	4.544358	-1.679489	1.255390
31	1	0	4.071406	-0.004434	1.552064

Rotational constants (GHZ): 1.1705685 0.3522548 0.2904456

Carvacrolate
Electronic Energy -617.031742
Stoichiometry C10H17O3(1-)
Framework group C1[X(C10H17O3)]
Deg. of freedom 84
Full point group C1 NOp 1
Largest Abelian subgroup C1 NOp 1
Largest concise Abelian subgroup C1 NOp 1
Standard orientation:

Center Number	Atomic Number	Atomic Type	Coordinates (Angstroms) X	Y	Z
1	6	0	-0.724978	1.583626	-0.104295
2	6	0	-0.842648	0.185508	-0.319697
3	6	0	0.341011	-0.583415	-0.269269
4	6	0	1.592109	-0.031647	-0.018486
5	6	0	1.683575	1.344646	0.192133
6	6	0	0.530195	2.123397	0.144739
7	1	0	2.641007	1.812981	0.390113
8	8	0	-2.004989	-0.369451	-0.566245
9	8	0	-4.269226	-0.373224	0.774481
10	1	0	-4.902699	0.250877	0.410960
11	1	0	-3.419508	-0.225472	0.275979
12	8	0	-2.814719	-2.867462	-0.219986
13	1	0	-2.374029	-1.995064	-0.403057
14	1	0	-3.634795	-2.592791	0.205486
15	1	0	0.250916	-1.653091	-0.438035
16	1	0	0.610774	3.195064	0.306719
17	6	0	-1.954791	2.446242	-0.158728
18	1	0	-2.462521	2.356993	-1.123754
19	1	0	-2.685336	2.157201	0.602236
20	1	0	-1.702050	3.496869	-0.002895
21	6	0	2.823097	-0.920397	0.020866
22	1	0	2.483880	-1.943309	-0.171167
23	6	0	3.495369	-0.907643	1.397482
24	1	0	2.793991	-1.199693	2.182371
25	1	0	4.339918	-1.601600	1.420984
26	1	0	3.875109	0.088256	1.640051
27	6	0	3.829096	-0.554177	-1.075134
28	1	0	4.675762	-1.245918	-1.068825
29	1	0	3.367041	-0.592125	-2.064263
30	1	0	4.221816	0.455071	-0.926579

Rotational constants (GHZ): 1.0622442 0.3937437 0.3138211

2,3-dimethylphenol
Electronic Energy -538.900010
Stoichiometry C8H14O3
Framework group C1[X(C8H14O3)]
Deg. of freedom 69
Full point group C1 NOp 1
Largest Abelian subgroup C1 NOp 1
Largest concise Abelian subgroup C1 NOp 1
Standard orientation:

Center Number	Atomic Number	Atomic Type	Coordinates (Angstroms) X	Y	Z
1	6	0	-0.827063	0.559985	0.092173
2	6	0	0.024940	-0.540189	0.230755
3	6	0	-0.452761	-1.844044	0.188185
4	6	0	-1.805899	-2.064316	-0.001198
5	6	0	-2.670023	-0.987901	-0.146518
6	6	0	-2.196234	0.322255	-0.101606
7	1	0	-2.187827	-3.077742	-0.036964
8	1	0	-3.728992	-1.163073	-0.296228
9	8	0	1.367982	-0.389265	0.434905
10	1	0	1.696368	0.523809	0.317619
11	8	0	3.923424	-1.062831	-0.478340
12	1	0	4.506404	-1.644385	0.019102
13	1	0	3.024684	-1.224627	-0.146315

14	8	0	3.173583	1.568015	-0.101308
15	1	0	3.756907	0.795436	-0.220867
16	1	0	3.614010	2.153859	0.521755
17	1	0	0.245100	-2.664516	0.302845
18	6	0	-0.249504	1.949383	0.167409
19	1	0	0.198894	2.138848	1.147760
20	1	0	0.535314	2.101701	-0.578249
21	1	0	-1.006198	2.711944	0.003407
22	6	0	-3.171221	1.461821	-0.258519
23	1	0	-3.175455	2.113796	0.618349
24	1	0	-2.929292	2.084744	-1.123099
25	1	0	-4.183231	1.080889	-0.395260

Rotational constants (GHZ): 1.6252953 0.5727865 0.4334124

2,3-dimethylphenolate
Electronic Energy -538.431915
Stoichiometry C8H13O3(1-)
Framework group C1[X(C8H13O3)]
Deg. of freedom 66
Full point group C1 NOp 1
Largest Abelian subgroup C1 NOp 1
Largest concise Abelian subgroup C1 NOp 1
Standard orientation:

Center Number	Atomic Number	Atomic Type	Coordinates (Angstroms) X	Y	Z
1	6	0	-0.116716	1.735461	0.136368
2	6	0	0.216018	0.372146	0.291149
3	6	0	-0.832424	-0.583910	0.184864
4	6	0	-2.139334	-0.170066	-0.090074
5	6	0	-2.429943	1.188041	-0.247361
6	6	0	-1.416423	2.131414	-0.129218
7	1	0	0.677728	2.469257	0.225104
8	1	0	-3.446360	1.498877	-0.461963
9	8	0	1.451048	0.000835	0.529306
10	8	0	3.660742	1.197529	-0.196068
11	1	0	4.040780	1.698905	0.529984
12	1	0	2.774613	0.863295	0.128850
13	8	0	3.136808	-1.763352	-0.523683
14	1	0	2.378617	-1.257257	-0.137356
15	1	0	3.802092	-1.071614	-0.620909
16	1	0	-1.643227	3.186206	-0.248644
17	6	0	-0.487720	-2.039109	0.362078
18	1	0	-0.192911	-2.506398	-0.584865
19	1	0	0.357083	-2.146553	1.042939
20	1	0	-1.325907	-2.614367	0.756760
21	6	0	-3.254343	-1.178339	-0.227480
22	1	0	-3.030360	-1.927843	-0.991090
23	1	0	-3.427964	-1.720322	0.706586
24	1	0	-4.187652	-0.686108	-0.503796

Rotational constants (GHZ): 1.5704121 0.6208995 0.4604893

2,4-dimethylphenol
Electronic Energy -538.901268
Stoichiometry C8H14O3
Framework group C1[X(C8H14O3)]
Deg. of freedom 69
Full point group C1 NOp 1
Largest Abelian subgroup C1 NOp 1
Largest concise Abelian subgroup C1 NOp 1
Standard orientation:

Center Number	Atomic Number	Atomic Type	Coordinates (Angstroms) X	Y	Z
1	6	0	0.534353	0.949192	-0.087337
2	6	0	-0.087926	-0.287430	-0.275675
3	6	0	0.657474	-1.458063	-0.288332
4	6	0	2.031642	-1.412640	-0.104948

5	6	0	2.686023	-0.200719	0.097870
6	6	0	1.914718	0.958964	0.098445
7	1	0	2.599896	-2.336340	-0.120303
8	8	0	-1.436091	-0.402231	-0.480354
9	1	0	-1.938058	0.417701	-0.301802
10	8	0	-3.779150	-1.595147	0.468060
11	1	0	-4.263651	-2.245491	-0.049607
12	1	0	-2.880016	-1.565495	0.100371
13	8	0	-3.553882	1.145068	0.223394
14	1	0	-3.974292	0.269652	0.316726
15	1	0	-4.120377	1.663568	-0.356071
16	1	0	2.404315	1.917028	0.244098
17	1	0	0.147984	-2.401458	-0.445603
18	6	0	-0.260069	2.226802	-0.100502
19	1	0	-0.759892	2.377713	-1.062038
20	1	0	-1.035879	2.233685	0.669433
21	1	0	0.390605	3.083275	0.073440
22	6	0	4.172815	-0.143938	0.333915
23	1	0	4.600987	0.783051	-0.051420
24	1	0	4.405073	-0.191613	1.401860
25	1	0	4.682116	-0.979811	-0.148488

Rotational constants (GHZ): 1.9418747 0.4959590 0.4051853
Standard basis: 6-311+G(d,p) (5D, 7F)

2,4-dimethylphenolate
Electronic Energy -538.432575
Stoichiometry C8H13O3(1-)
Framework group C1[X(C8H13O3)]
Deg. of freedom 66
Full point group C1 NOp 1
Largest Abelian subgroup C1 NOp 1
Largest concise Abelian subgroup C1 NOp 1
Standard orientation:

Center Number	Atomic Number	Atomic Type	Coordinates (Angstroms) X	Y	Z
1	6	0	-0.422160	-1.374535	-0.213073
2	6	0	0.263806	-0.149389	-0.306981
3	6	0	-0.512139	1.035138	-0.178094
4	6	0	-1.880981	0.942658	0.031780
5	6	0	-2.557667	-0.277102	0.127795
6	6	0	-1.795480	-1.432472	-0.000816
7	1	0	0.150746	-2.290937	-0.314344
8	1	0	-2.450469	1.864948	0.124357
9	8	0	1.559316	-0.090733	-0.514097
10	8	0	3.370021	-1.850134	0.157500
11	1	0	3.618701	-2.393031	-0.595071
12	1	0	2.606266	-1.278792	-0.150334
13	8	0	3.621479	1.128127	0.638387
14	1	0	2.766603	0.850202	0.222969
15	1	0	4.088478	0.286677	0.704206
16	1	0	-2.280446	-2.402523	0.063956
17	6	0	0.172789	2.369390	-0.278529
18	1	0	0.925639	2.493815	0.505235
19	1	0	0.700128	2.475124	-1.231037
20	1	0	-0.545485	3.186736	-0.191683
21	6	0	-4.045508	-0.325764	0.368995
22	1	0	-4.310523	0.069415	1.354468
23	1	0	-4.595175	0.263752	-0.370158
24	1	0	-4.416940	-1.351011	0.316657

Rotational constants (GHZ): 1.7782764 0.5428769 0.4307223

2,6-diisopropylphenol
Electronic Energy -696.094172
Stoichiometry C12H22O3
Framework group C1[X(C12H22O3)]
Deg. of freedom 105
Full point group C1 NOp 1
Largest Abelian subgroup C1 NOp 1

Largest concise Abelian subgroup C1 NOp 1
Standard orientation:

Center Number	Atomic Number	Atomic Type	Coordinates (Angstroms) X	Y	Z
1	6	0	1.117396	-1.021888	-0.079110
2	6	0	-0.059336	-0.266160	-0.133653
3	6	0	-1.329336	-0.855185	-0.117149
4	6	0	-1.401326	-2.240179	-0.035297
5	6	0	-0.253509	-3.015761	0.023384
6	6	0	0.989566	-2.405927	-0.001396
7	1	0	-2.369126	-2.724927	-0.026571
8	1	0	-0.328227	-4.094759	0.082251
9	8	0	-0.028055	1.102016	-0.199083
10	1	0	0.856668	1.476925	-0.359194
11	8	0	-0.845787	3.742564	-0.106490
12	1	0	-1.323768	4.110059	0.640522
13	1	0	-0.937047	2.776469	-0.053148
14	8	0	1.790018	3.110565	-0.526348
15	1	0	0.979678	3.636725	-0.389644
16	1	0	2.242963	3.489411	-1.283117
17	1	0	1.879523	-3.020594	0.037492
18	6	0	-2.562322	0.028687	-0.162134
19	1	0	-2.332222	0.856323	-0.839066
20	6	0	-2.850961	0.624183	1.223221
21	1	0	-1.977828	1.140753	1.625810
22	1	0	-3.682729	1.332694	1.176710
23	1	0	-3.121409	-0.165945	1.928736
24	6	0	-3.805197	-0.671816	-0.708622
25	1	0	-4.617238	0.049475	-0.825344
26	1	0	-3.615120	-1.126653	-1.682882
27	1	0	-4.162477	-1.452961	-0.032885
28	6	0	2.473554	-0.336215	-0.057428
29	1	0	2.429318	0.529117	-0.727970
30	6	0	2.794371	0.187419	1.349601
31	1	0	3.736090	0.741235	1.350524
32	1	0	2.015256	0.854872	1.720588
33	1	0	2.885139	-0.647045	2.049844
34	6	0	3.614746	-1.212547	-0.573334
35	1	0	3.393276	-1.624325	-1.560020
36	1	0	4.529934	-0.621354	-0.649150
37	1	0	3.824061	-2.044314	0.103391

Rotational constants (GHZ): 0.5782226 0.4700602 0.2833303

2,6-diisopropylphenolate
Electronic Energy -695.624897
Stoichiometry C12H21O3(1-)
Framework group C1[X(C12H21O3)]
Deg. of freedom 102
Full point group C1 NOp 1
Largest Abelian subgroup C1 NOp 1
Largest concise Abelian subgroup C1 NOp 1
Standard orientation:

Center Number	Atomic Number	Atomic Type	Coordinates (Angstroms) X	Y	Z
1	6	0	1.358986	-0.756285	-0.046465
2	6	0	0.069902	-0.140274	-0.060458
3	6	0	-1.075140	-0.989154	-0.116270
4	6	0	-0.913596	-2.367445	-0.131883
5	6	0	0.343743	-2.959020	-0.105504
6	6	0	1.466768	-2.138370	-0.066545
7	1	0	-1.791396	-3.005336	-0.173193
8	1	0	0.448086	-4.038291	-0.124417
9	8	0	-0.037165	1.153091	-0.028665
10	8	0	0.475970	3.387682	-1.575367
11	1	0	-0.276447	3.771058	-1.108414
12	1	0	0.496613	2.494700	-1.174380
13	8	0	-1.556722	3.193460	0.641465

14	1	0	-1.095562	2.337698	0.420597
15	1	0	-1.091015	3.512232	1.417104
16	1	0	2.450004	-2.597917	-0.057373
17	6	0	-2.442748	-0.336507	-0.121060
18	1	0	-2.314434	0.637401	-0.599861
19	6	0	-3.500267	-1.109528	-0.910583
20	1	0	-3.747593	-2.064541	-0.436667
21	1	0	-4.425839	-0.528872	-0.971796
22	1	0	-3.160930	-1.318845	-1.927934
23	6	0	-2.930358	-0.075407	1.309879
24	1	0	-2.195516	0.509126	1.864194
25	1	0	-3.872849	0.481806	1.311803
26	1	0	-3.086065	-1.022011	1.838593
27	6	0	2.564827	0.159850	0.019909
28	1	0	2.342548	1.010051	-0.631513
29	6	0	3.867579	-0.475265	-0.463948
30	1	0	4.664041	0.274433	-0.491037
31	1	0	4.200172	-1.278790	0.200896
32	1	0	3.760883	-0.893502	-1.468011
33	6	0	2.739383	0.727475	1.434553
34	1	0	3.553397	1.460086	1.467975
35	1	0	1.819589	1.219667	1.750628
36	1	0	2.971170	-0.074433	2.143591

Rotational constants (GHZ): 0.5861723 0.4602902 0.2994736

2-methyl-4-*terz*-butylphenol

Electronic Energy -656.796670

Stoichiometry C11H20O3

Framework group C1[X(C11H20O3)]

Deg. of freedom 96

Full point group C1 NOp 1

Largest Abelian subgroup C1 NOp 1

Largest concise Abelian subgroup C1 NOp 1

Standard orientation:

Center Number	Atomic Number	Atomic Type	Coordinates (Angstroms) X	Y	Z
1	6	0	0.984831	-1.396329	-0.211543
2	6	0	-0.397022	-1.450005	-0.348754
3	6	0	-1.150543	-0.288779	-0.316262
4	6	0	-0.526975	0.951807	-0.150763
5	6	0	0.856153	0.968640	-0.012079
6	6	0	1.645799	-0.183905	-0.037194
7	1	0	1.537021	-2.325698	-0.241519
8	1	0	-0.902206	-2.399614	-0.481070
9	1	0	1.328967	1.935941	0.117691
10	8	0	-2.503937	-0.410321	-0.472398
11	1	0	-3.003610	0.406956	-0.276429
12	8	0	-4.611026	1.120203	0.295212
13	1	0	-5.193481	1.644167	-0.263071
14	1	0	-5.025299	0.241635	0.386316
15	8	0	-4.821655	-1.624544	0.507724
16	1	0	-3.930025	-1.587072	0.122971
17	1	0	-5.314836	-2.267960	-0.010362
18	6	0	3.166427	-0.073622	0.121534
19	6	0	3.741508	0.795492	-1.010042
20	1	0	3.322885	1.803521	-0.998471
21	1	0	4.826322	0.882927	-0.905373
22	1	0	3.528982	0.352880	-1.986340
23	6	0	3.498025	0.575895	1.476034
24	1	0	3.108526	-0.025420	2.301233
25	1	0	4.581075	0.660975	1.599189
26	1	0	3.073070	1.577908	1.559022
27	6	0	3.852822	-1.442347	0.067906
28	1	0	3.508803	-2.100749	0.868897
29	1	0	3.681706	-1.945373	-0.886615
30	1	0	4.931061	-1.313106	0.185500
31	6	0	-1.328954	2.224826	-0.134889
32	1	0	-1.866397	2.371209	-1.076620
33	1	0	-2.074532	2.228431	0.664451

34	1	0	-0.677522	3.085699	0.012616

Rotational constants (GHZ): 1.3749188 0.2733416 0.2437086

2-methyl-4-*terz*-butylphenolate
Electronic Energy -656.327999
Stoichiometry C11H19O3(1-)
Framework group C1[X(C11H19O3)]
Deg. of freedom 93
Full point group C1 NOp 1
Largest Abelian subgroup C1 NOp 1
Largest concise Abelian subgroup C1 NOp 1
Standard orientation:

Center Number	Atomic Number	Atomic Type	Coordinates (Angstroms) X	Y	Z
1	6	0	-0.808052	1.025357	0.084874
2	6	0	0.570056	1.071601	0.233095
3	6	0	1.309991	-0.137017	0.342400
4	6	0	0.574136	-1.333517	0.289654
5	6	0	-0.809354	-1.344989	0.138180
6	6	0	-1.542612	-0.166995	0.031598
7	1	0	-1.332700	1.973470	0.007375
8	1	0	1.114856	-2.271282	0.372713
9	1	0	-1.309431	-2.305311	0.105793
10	8	0	2.614079	-0.123658	0.492951
11	8	0	4.350336	-1.939404	-0.224267
12	1	0	4.599694	-2.490792	0.521890
13	1	0	3.612724	-1.344904	0.102316
14	8	0	4.690519	1.043111	-0.683710
15	1	0	3.834573	0.788566	-0.255665
16	1	0	5.128343	0.187819	-0.767810
17	6	0	-3.066163	-0.128392	-0.132837
18	6	0	-3.699986	0.632878	1.044683
19	1	0	-3.331291	1.658742	1.105009
20	1	0	-4.787579	0.673994	0.933591
21	1	0	-3.473778	0.137950	1.992620
22	6	0	-3.433498	0.587276	-1.444549
23	1	0	-3.013211	0.059718	-2.304618
24	1	0	-4.519804	0.627122	-1.567863
25	1	0	-3.057633	1.612116	-1.462520
26	6	0	-3.678203	-1.532643	-0.172937
27	1	0	-3.291980	-2.118952	-1.010048
28	1	0	-3.483482	-2.085858	0.748841
29	1	0	-4.761830	-1.456993	-0.292246
30	6	0	1.303881	2.382646	0.284765
31	1	0	1.873230	2.483625	1.213270
32	1	0	2.027451	2.469952	-0.530955
33	1	0	0.611185	3.223398	0.214962

Rotational constants (GHZ): 1.2874803 0.2915888 0.2560299

4-bromothymol
Electronic Energy -3191.144679
Stoichiometry C10H17BrO3
Framework group C1[X(C10H17BrO3)]
Deg. of freedom 87
Full point group C1 NOp 1
Largest Abelian subgroup C1 NOp 1
Largest concise Abelian subgroup C1 NOp 1
Standard orientation:

Center Number	Atomic Number	Atomic Type	Coordinates (Angstroms) X	Y	Z
1	6	0	-1.413955	2.156170	-0.299924
2	1	0	-2.257457	1.912828	-0.951041
3	6	0	-0.543058	0.916588	-0.236902
4	6	0	0.838119	0.974934	-0.104127
5	6	0	-1.126544	-0.354954	-0.277834
6	6	0	1.604158	-0.178073	-0.014708

7	1	0	1.332430	1.936215	-0.072311
8	6	0	-0.348963	-1.502970	-0.191225
9	6	0	1.036481	-1.446926	-0.056360
10	1	0	-0.835314	-2.471765	-0.230189
11	6	0	-0.707501	3.374403	-0.892434
12	1	0	-1.421162	4.191962	-1.014375
13	1	0	0.092089	3.737856	-0.242055
14	1	0	-0.276099	3.153469	-1.871145
15	6	0	-1.981174	2.483343	1.088147
16	1	0	-2.679254	3.322144	1.030971
17	1	0	-2.509376	1.629255	1.516048
18	1	0	-1.175995	2.758913	1.774818
19	6	0	1.841640	-2.711478	0.034358
20	1	0	1.191557	-3.584252	-0.017551
21	1	0	2.568375	-2.776058	-0.778358
22	1	0	2.402216	-2.756304	0.970584
23	8	0	-2.477872	-0.434930	-0.411292
24	1	0	-2.791981	-1.365910	-0.433634
25	8	0	-5.167463	-0.321713	0.512151
26	1	0	-4.295888	0.085945	0.396944
27	1	0	-5.499309	-0.015678	1.362012
28	8	0	-3.937411	-2.679731	-0.184042
29	1	0	-4.293850	-3.196497	-0.913450
30	1	0	-4.643720	-2.069559	0.101806
31	35	0	3.501700	0.020920	0.164070

Rotational constants (GHZ): 0.6973444 0.2396645

4-bromothymolate

Electronic Energy -3190.677936

Stoichiometry C10H16BrO3(1-)

Framework group C1[X(C10H16BrO3)]

Deg. of freedom 84

Full point group C1 NOp 1

Largest Abelian subgroup C1 NOp 1

Largest concise Abelian subgroup C1 NOp 1

Standard orientation:

Center Number	Atomic Number	Atomic Type	Coordinates (Angstroms) X	Y	Z
1	6	0	1.569883	1.998840	-0.224633
2	1	0	2.410230	1.703475	-0.859757
3	6	0	0.635129	0.807091	-0.180669
4	6	0	-0.740405	0.949497	-0.090180
5	6	0	1.191904	-0.505418	-0.203992
6	6	0	-1.584161	-0.152573	-0.019821
7	1	0	-1.177167	1.940061	-0.075579
8	6	0	0.293613	-1.592732	-0.136417
9	6	0	-1.087332	-1.451929	-0.042260
10	1	0	0.716626	-2.592157	-0.160095
11	6	0	2.137622	2.296001	1.170002
12	1	0	2.885766	3.092779	1.126276
13	1	0	1.339922	2.619175	1.845571
14	1	0	2.609189	1.410537	1.598806
15	6	0	0.950895	3.260035	-0.826039
16	1	0	1.711549	4.037837	-0.928514
17	1	0	0.527641	3.069181	-1.815027
18	1	0	0.157798	3.666685	-0.192584
19	6	0	-1.971620	-2.666537	0.026922
20	1	0	-2.679078	-2.692435	-0.805351
21	1	0	-1.373198	-3.576980	-0.005969
22	1	0	-2.561320	-2.678327	0.946668
23	8	0	2.477235	-0.700662	-0.293642
24	8	0	4.047917	-2.763200	0.066201
25	1	0	4.041213	-3.352200	-0.692869
26	1	0	3.338926	-2.079917	-0.104408
27	8	0	4.996676	0.064785	0.170310
28	1	0	4.024368	0.028906	0.020067
29	1	0	5.220431	-0.874722	0.182382
30	35	0	-3.478306	0.166202	0.101141

Rotational constants (GHZ): 0.7297818 0.2430774 0.1882574

4-chlorothymol
Electronic Energy -1077.127149
Stoichiometry C10H17ClO3
Framework group C1[X(C10H17ClO3)]
Deg. of freedom 87
Full point group C1 NOp 1
Largest Abelian subgroup C1 NOp 1
Largest concise Abelian subgroup C1 NOp 1
Standard orientation:

Center Number	Atomic Number	Atomic Type	Coordinates (Angstroms) X	Y	Z
1	6	0	0.114163	-1.447452	-0.271486
2	6	0	-0.628238	-0.273697	-0.240127
3	6	0	-0.011991	0.966412	-0.034688
4	6	0	1.366618	0.967142	0.132386
5	6	0	2.097332	-0.210407	0.094062
6	6	0	1.497125	-1.447454	-0.107163
7	1	0	-0.399429	-2.390049	-0.426755
8	1	0	1.890110	1.898699	0.298929
9	8	0	-1.979801	-0.296309	-0.406105
10	1	0	-2.311194	-1.212030	-0.538864
11	6	0	-0.840602	2.236509	-0.033585
12	1	0	-1.782997	1.996729	0.466684
13	6	0	-1.179054	2.658311	-1.469856
14	1	0	-1.679835	1.854107	-2.010604
15	1	0	-1.837770	3.530386	-1.467953
16	1	0	-0.268622	2.923356	-2.014552
17	6	0	-0.194743	3.392219	0.728114
18	1	0	-0.891927	4.230644	0.786085
19	1	0	0.073867	3.105386	1.747179
20	1	0	0.706649	3.755168	0.227652
21	6	0	2.275738	-2.730919	-0.145757
22	1	0	2.815388	-2.893616	0.789782
23	1	0	1.610686	-3.578173	-0.309438
24	1	0	3.018829	-2.717916	-0.946156
25	8	0	-4.346373	-0.544104	1.195535
26	1	0	-3.532425	-0.083249	0.940339
27	1	0	-4.419455	-0.455730	2.151056
28	8	0	-3.353798	-2.611723	-0.315839
29	1	0	-3.885884	-2.981278	-1.027473
30	1	0	-3.963230	-2.126638	0.272330
31	17	0	3.842290	-0.111923	0.315390

Rotational constants (GHZ): 0.6526065 0.3495687 0.2473178

4-chlorothymolate
Electronic Energy -1076.659978
Stoichiometry C10H16ClO3(1-)
Framework group C1[X(C10H16ClO3)]
Deg. of freedom 84
Full point group C1 NOp 1
Largest Abelian subgroup C1 NOp 1
Largest concise Abelian subgroup C1 NOp 1
Standard orientation:

Center Number	Atomic Number	Atomic Type	Coordinates (Angstroms) X	Y	Z
1	6	0	0.307575	-1.545590	-0.129715
2	6	0	-0.622766	-0.487030	-0.215296
3	6	0	-0.108808	0.842174	-0.178082
4	6	0	1.259441	1.028545	-0.057442
5	6	0	2.134372	-0.045912	0.029409
6	6	0	1.680410	-1.359431	-0.005231
7	1	0	-0.082002	-2.558202	-0.163202
8	1	0	1.668356	2.031055	-0.031409
9	8	0	-1.900397	-0.722624	-0.333693
10	6	0	-1.079395	2.003916	-0.239420

11	1	0	-1.906485	1.675596	-0.875463
12	6	0	-0.495029	3.277092	-0.850459
13	1	0	-0.060144	3.088552	-1.834832
14	1	0	-1.277888	4.030749	-0.965714
15	1	0	0.281710	3.713967	-0.216876
16	6	0	-1.663813	2.297652	1.149033
17	1	0	-2.435480	3.070937	1.093469
18	1	0	-2.110783	1.402588	1.584430
19	1	0	-0.880257	2.651134	1.825997
20	6	0	2.611071	-2.537429	0.082802
21	1	0	2.050928	-3.471391	0.037942
22	1	0	3.336349	-2.534218	-0.734499
23	1	0	3.181951	-2.525548	1.014428
24	8	0	-3.361018	-2.871412	-0.034340
25	1	0	-3.358366	-3.411843	-0.828740
26	1	0	-2.690924	-2.144931	-0.188067
27	8	0	-4.429020	-0.113188	0.288949
28	1	0	-3.464634	-0.090790	0.092070
29	1	0	-4.605750	-1.062260	0.254308
30	17	0	3.866974	0.291086	0.179721

Rotational constants (GHZ): 0.7266745 0.3455481 0.2444736

4-methoxyphenol
Electronic Energy -574.807648
Stoichiometry C7H12O4
Framework group C1[X(C7H12O4)]
Deg. of freedom 63
Full point group C1 NOp 1
Largest Abelian subgroup C1 NOp 1
Largest concise Abelian subgroup C1 NOp 1
Standard orientation:

Center Number	Atomic Number	Atomic Type	Coordinates (Angstroms) X	Y	Z
1	6	0	1.997292	1.079941	-0.533056
2	6	0	0.760395	1.674488	-0.336983
3	6	0	-0.329027	0.909290	0.074707
4	6	0	-0.165375	-0.458008	0.287717
5	6	0	1.077585	-1.047652	0.093801
6	6	0	2.159063	-0.283018	-0.316375
7	1	0	2.842299	1.672631	-0.863537
8	1	0	0.623777	2.735495	-0.507699
9	1	0	-1.012791	-1.054167	0.604246
10	1	0	1.210797	-2.110899	0.254877
11	8	0	-1.519044	1.536896	0.248661
12	1	0	-2.218518	0.906767	0.535758
13	8	0	-3.461603	-0.153117	1.039999
14	1	0	-3.940566	0.060476	1.846651
15	1	0	-4.130193	-0.448274	0.387436
16	8	0	-5.307896	-0.988280	-0.805992
17	1	0	-5.419586	-0.467187	-1.608422
18	1	0	-5.284775	-1.907927	-1.091596
19	8	0	3.377359	-0.889990	-0.551777
20	6	0	4.318512	-0.751396	0.509155
21	1	0	5.227777	-1.260290	0.194151
22	1	0	4.540836	0.301873	0.702039
23	1	0	3.939749	-1.214425	1.425180

Rotational constants (GHZ): 2.2281291 0.3515143 0.3252443

4-methoxyphenolate
Electronic Energy -574.339591
Stoichiometry C7H11O4(1-)
Framework group C1[X(C7H11O4)]
Deg. of freedom 60
Full point group C1 NOp 1
Largest Abelian subgroup C1 NOp 1
Largest concise Abelian subgroup C1 NOp 1
Standard orientation:

Center Number	Atomic Number	Atomic Type	Coordinates (Angstroms) X	Y	Z
1	6	0	1.288567	-1.200996	-0.244265
2	6	0	-0.094854	-1.202345	-0.138756
3	6	0	-0.836339	-0.000410	-0.085550
4	6	0	-0.092268	1.199512	-0.148816
5	6	0	1.291001	1.194313	-0.255390
6	6	0	1.990103	-0.004335	-0.303067
7	1	0	1.834628	-2.137543	-0.288824
8	1	0	-0.632907	-2.143131	-0.098805
9	1	0	-0.628329	2.141796	-0.118542
10	1	0	1.838833	2.129370	-0.308861
11	8	0	-2.144864	0.001350	0.013286
12	8	0	-3.617764	2.169691	0.074262
13	1	0	-3.451203	2.609300	0.911993
14	1	0	-3.018449	1.367737	0.065364
15	8	0	-3.606947	-2.160226	0.281758
16	1	0	-3.014926	-1.362349	0.160357
17	1	0	-3.581356	-2.632063	-0.554621
18	8	0	3.370794	-0.006757	-0.450790
19	6	0	4.086204	0.007675	0.778429
20	1	0	3.851330	-0.877190	1.378197
21	1	0	5.147371	0.005288	0.532308
22	1	0	3.850770	0.905847	1.357807

Rotational constants (GHZ): 1.8247603 0.4606836 0.3790182

4-hydroxybenzonitrile
Electronic Energy -552.532032
Stoichiometry C7H9NO3
Framework group C1[X(C7H9NO3)]
Deg. of freedom 54
Full point group C1 NOp 1
Largest Abelian subgroup C1 NOp 1
Largest concise Abelian subgroup C1 NOp 1
Standard orientation:

Center Number	Atomic Number	Atomic Type	Coordinates (Angstroms) X	Y	Z
1	6	0	1.760719	-1.233786	-0.233659
2	6	0	0.410802	-1.379265	-0.477235
3	6	0	-0.429988	-0.266471	-0.431992
4	6	0	0.092629	0.994505	-0.138720
5	6	0	1.444747	1.138891	0.104307
6	6	0	2.289410	0.027444	0.059009
7	1	0	2.412635	-2.097126	-0.269129
8	1	0	-0.010881	-2.349605	-0.705633
9	1	0	-0.565219	1.854117	-0.101794
10	1	0	1.850287	2.116492	0.331222
11	8	0	-1.739966	-0.457321	-0.677920
12	1	0	-2.255192	0.382853	-0.613263
13	8	0	-3.462163	1.500797	-0.144653
14	1	0	-4.036489	1.929152	-0.787331
15	1	0	-4.000224	0.831614	0.320538
16	8	0	-4.173394	-0.923308	0.903977
17	1	0	-3.316865	-1.269228	0.616522
18	1	0	-4.290112	-1.215233	1.813730
19	6	0	3.686303	0.178409	0.310336
20	7	0	4.812644	0.299607	0.513092

Rotational constants (GHZ): 2.7056010 0.4048195 0.3713120

4-cyanophenolate
Electronic Energy -552.073689
Stoichiometry C7H8NO3(1-)
Framework group C1[X(C7H8NO3)]
Deg. of freedom 51
Full point group C1 NOp 1
Largest Abelian subgroup C1 NOp 1
Largest concise Abelian subgroup C1 NOp 1

Standard orientation:

Center Number	Atomic Number	Atomic Type	Coordinates (Angstroms) X	Y	Z
1	6	0	-1.447784	1.203839	0.099918
2	6	0	-0.072665	1.206143	0.100448
3	6	0	0.674485	-0.000002	0.000006
4	6	0	-0.072664	-1.206147	-0.100431
5	6	0	-1.447783	-1.203845	-0.099896
6	6	0	-2.162673	-0.000003	0.000013
7	1	0	-1.988483	2.140024	0.178015
8	1	0	0.470674	2.140474	0.178987
9	1	0	0.470675	-2.140477	-0.178974
10	1	0	-1.988482	-2.140030	-0.177990
11	8	0	1.965846	-0.000001	0.000005
12	8	0	3.471582	-2.194191	0.014082
13	1	0	3.906521	-2.206092	0.870666
14	1	0	2.886358	-1.392139	0.022082
15	8	0	3.471470	2.194265	-0.014187
16	1	0	2.886307	1.392168	-0.022139
17	1	0	3.907088	2.205648	-0.870434
18	6	0	-3.582309	-0.000004	0.000017
19	7	0	-4.736213	-0.000007	0.000020

Rotational constants (GHZ): 1.9585189 0.4658555 0.3775323

4-nitrophenol
Electronic Energy -664.810429
Stoichiometry C6H9NO5
Framework group C1[X(C6H9NO5)]
Deg. of freedom 57
Full point group C1 NOp 1
Largest Abelian subgroup C1 NOp 1
Largest concise Abelian subgroup C1 NOp 1
Standard orientation:

Center Number	Atomic Number	Atomic Type	Coordinates (Angstroms) X	Y	Z
1	6	0	1.724438	-1.283635	0.286206
2	6	0	0.447555	-1.791147	0.194445
3	6	0	-0.619675	-0.955155	-0.155303
4	6	0	-0.387099	0.402115	-0.412919
5	6	0	0.891277	0.911432	-0.321777
6	6	0	1.938512	0.066718	0.027044
7	1	0	2.555551	-1.919473	0.555481
8	1	0	0.251499	-2.837283	0.390018
9	1	0	-1.217060	1.041997	-0.683289
10	1	0	1.084389	1.956366	-0.517987
11	8	0	-1.837755	-1.498645	-0.229715
12	1	0	-2.529050	-0.834413	-0.484182
13	8	0	-3.682371	0.274100	-0.905431
14	1	0	-4.149299	0.149820	-1.737703
15	1	0	-4.353332	0.539222	-0.240605
16	8	0	-5.543663	1.006126	0.950793
17	1	0	-5.797169	0.366093	1.624621
18	1	0	-5.433181	1.844319	1.412405
19	7	0	3.281595	0.603627	0.123056
20	8	0	4.191072	-0.152498	0.433713
21	8	0	3.453523	1.791667	-0.110150

Rotational constants (GHZ): 1.9550780 0.3073942 0.2791405

4-nitrophenolate
Electronic Energy -664.352792
Stoichiometry C6H8NO5(1-)
Framework group C1[X(C6H8NO5)]
Deg. of freedom 54
Full point group C1 NOp 1
Largest Abelian subgroup C1 NOp 1
Largest concise Abelian subgroup C1 NOp 1

Standard orientation:

Center Number	Atomic Number	Atomic Type	Coordinates (Angstroms) X	Y	Z
1	6	0	1.042199	1.211853	-0.086639
2	6	0	-0.327398	1.213290	-0.086555
3	6	0	-1.076321	-0.000006	0.000033
4	6	0	-0.327392	-1.213300	0.086604
5	6	0	1.042205	-1.211859	0.086658
6	6	0	1.740065	-0.000002	0.000001
7	1	0	1.595558	2.138485	-0.153612
8	1	0	-0.873546	2.146392	-0.153700
9	1	0	-0.873535	-2.146404	0.153761
10	1	0	1.595568	-2.138489	0.153617
11	8	0	-2.357634	-0.000009	0.000049
12	8	0	-3.880908	-2.207964	-0.039455
13	1	0	-4.387198	-2.158815	-0.854627
14	1	0	-3.300244	-1.407304	-0.038281
15	8	0	-3.880907	2.207950	0.039359
16	1	0	-3.300236	1.407295	0.038260
17	1	0	-4.386844	2.159128	0.854769
18	7	0	3.162258	0.000001	-0.000014
19	8	0	3.761880	1.074001	-0.078747
20	8	0	3.761885	-1.073998	0.078705

Rotational constants (GHZ): 1.6987616 0.3855470

4-nitrothymol
Electronic Energy -822.006861
Stoichiometry C10H17NO5
Framework group C1[X(C10H17NO5)]
Deg. of freedom 93
Full point group C1 NOp 1
Largest Abelian subgroup C1 NOp 1
Largest concise Abelian subgroup C1 NOp 1

Standard orientation:

Center Number	Atomic Number	Atomic Type	Coordinates (Angstroms) X	Y	Z
1	6	0	-1.196078	0.970338	-0.077155
2	6	0	0.174029	0.930770	-0.220839
3	6	0	0.770524	-0.342051	-0.244527
4	6	0	0.003725	-1.498623	-0.130043
5	6	0	-1.378456	-1.467962	0.001512
6	6	0	-1.959029	-0.193012	0.025332
7	1	0	-1.713959	1.917540	-0.053847
8	1	0	0.507945	-2.458035	-0.140396
9	8	0	2.106270	-0.408944	-0.384815
10	1	0	2.439190	-1.338998	-0.392839
11	8	0	3.510106	-2.653708	-0.172860
12	1	0	3.797132	-3.199347	-0.912049
13	1	0	4.263393	-2.080280	0.067806
14	8	0	4.915800	-0.379753	0.414522
15	1	0	4.098410	0.132438	0.345718
16	1	0	5.344841	-0.092131	1.226884
17	7	0	-3.393743	-0.011599	0.158846
18	8	0	-3.812590	1.080659	0.521068
19	8	0	-4.135090	-0.948019	-0.101480
20	6	0	1.033806	2.174539	-0.318880
21	1	0	1.854380	1.934604	-0.999978
22	6	0	1.648604	2.508154	1.047350
23	1	0	2.198402	1.659412	1.457759
24	1	0	2.338211	3.351146	0.961117
25	1	0	0.866964	2.781224	1.761456
26	6	0	0.294681	3.384442	-0.886830
27	1	0	-0.168627	3.158760	-1.849709
28	1	0	-0.484536	3.740269	-0.208059
29	1	0	0.995785	4.208383	-1.034049
30	6	0	-2.115483	-2.771821	0.131060
31	1	0	-2.800468	-2.766979	0.978947

32	1	0	-2.714874	-2.974640	-0.756747
33	1	0	-1.399887	-3.582688	0.262711

Rotational constants (GHZ): 0.6612248 0.2995492 0.2153042

4-nitrothymolate
Electronic Energy -821.549751
Stoichiometry C10H16NO5(1-)
Framework group C1[X(C10H16NO5)]
Deg. of freedom 90
Full point group C1 NOp 1
Largest Abelian subgroup C1 NOp 1
Largest concise Abelian subgroup C1 NOp 1
Standard orientation:

Center Number	Atomic Number	Atomic Type	Coordinates (Angstroms) X	Y	Z
1	6	0	-1.088155	0.974404	-0.080034
2	6	0	0.270244	0.825059	-0.108675
3	6	0	0.811962	-0.508651	-0.076357
4	6	0	-0.110591	-1.590356	-0.013019
5	6	0	-1.479629	-1.445079	0.022422
6	6	0	-1.976120	-0.118230	-0.012058
7	1	0	-1.526341	1.960976	-0.110629
8	1	0	0.312334	-2.588671	0.006083
9	8	0	2.071487	-0.719355	-0.108448
10	8	0	3.775688	-2.763307	-0.179252
11	1	0	3.694592	-3.357460	0.571810
12	1	0	3.012463	-2.136619	-0.119912
13	8	0	4.694794	0.022887	-0.135720
14	1	0	3.717430	0.040857	-0.120894
15	1	0	4.866753	-0.928943	-0.128069
16	7	0	-3.362971	0.182324	0.012156
17	8	0	-3.727929	1.363505	-0.051781
18	8	0	-4.188664	-0.731216	0.098345
19	6	0	1.229414	1.995549	-0.150134
20	1	0	2.053104	1.695559	-0.805290
21	6	0	1.824823	2.257237	1.240289
22	1	0	2.280864	1.355203	1.650479
23	1	0	2.591021	3.035783	1.193732
24	1	0	1.045278	2.591362	1.931083
25	6	0	0.623643	3.276390	-0.720074
26	1	0	0.186278	3.111747	-1.707484
27	1	0	-0.155024	3.681836	-0.068321
28	1	0	1.396014	4.042855	-0.816091
29	6	0	-2.323928	-2.688831	0.087602
30	1	0	-2.942413	-2.706260	0.985289
31	1	0	-3.008073	-2.753780	-0.758873
32	1	0	-1.676463	-3.565784	0.087067

Rotational constants (GHZ): 0.6940206 0.3012712 0.2174272

- $2H_2O$/CAM-B3LYP 6-311G+dp/CPCM

H_2O
Electronic Energy -229.330057
Stoichiometry H6O3
Framework group C1[X(H6O3)]
Deg. of freedom 21
Full point group C1 NOp 1
Largest Abelian subgroup C1 NOp 1
Largest concise Abelian subgroup C1 NOp 1
Standard orientation:

Center Number	Atomic Number	Atomic Type	Coordinates (Angstroms) X	Y	Z
1	8	0	0.000027	0.771636	-0.000040
2	1	0	0.000772	1.353128	0.768172

3	1	0	-0.001205	1.353830	-0.767718
4	8	0	-2.657232	-0.369073	-0.000223
5	1	0	-2.720716	-1.326416	0.001483
6	1	0	-1.713112	-0.161121	0.000730
7	8	0	2.657268	-0.369069	0.000209
8	1	0	2.720574	-1.326425	-0.001593
9	1	0	1.713184	-0.160946	-0.000632

Rotational constants (GHZ): 22.6022816 2.0386116 1.8865469

OH^-
Electronic Energy -152.393735
Stoichiometry H3O2(1-)
Framework group C1[X(H3O2)]
Deg. of freedom 9
Full point group C1 NOp 1
Largest Abelian subgroup C1 NOp 1
Largest concise Abelian subgroup C1 NOp 1
Standard orientation:

Center Number	Atomic Number	Atomic Type	Coordinates (Angstroms) X	Y	Z
1	8	0	1.248218	0.055511	-0.095601
2	1	0	1.684853	-0.281910	0.690602
3	8	0	-1.239815	-0.107854	-0.024696
4	1	0	-1.567990	0.737123	0.291814
5	1	0	-0.184086	-0.036471	-0.020040

Rotational constants (GHZ): 337.1039073 9.0856433 9.0651418

Phenol
Electronic Energy -460.298289
Stoichiometry C6H10O3
Framework group C1[X(C6H10O3)]
Deg. of freedom 51
Full point group C1 NOp 1
Largest Abelian subgroup C1 NOp 1
Largest concise Abelian subgroup C1 NOp 1
Standard orientation:

Center Number	Atomic Number	Atomic Type	Coordinates (Angstroms) X	Y	Z
1	6	0	2.579260	-1.016676	-0.040705
2	6	0	1.253485	-1.237462	-0.379970
3	6	0	0.346478	-0.185920	-0.328551
4	6	0	0.765128	1.081408	0.061626
5	6	0	2.096200	1.289930	0.395107
6	6	0	3.009913	0.246168	0.347385
7	1	0	3.283009	-1.839665	-0.083224
8	1	0	0.907538	-2.215265	-0.691722
9	1	0	0.046779	1.892161	0.109070
10	1	0	2.417832	2.279297	0.698418
11	1	0	4.046814	0.414137	0.609927
12	8	0	-0.950101	-0.442795	-0.665179
13	1	0	-1.485870	0.376792	-0.670092
14	8	0	-2.896718	1.394032	-0.243515
15	1	0	-3.524160	1.828118	-0.825832
16	1	0	-3.367944	0.667279	0.203110
17	8	0	-3.222605	-1.107456	0.798023
18	1	0	-2.363787	-1.283333	0.378914
19	1	0	-3.707608	-1.934460	0.827446

Rotational constants (GHZ): 2.8958025 0.7313665 0.6242864

Phenolate
Electronic Energy -459.832127
Stoichiometry C6H9O3(1-)
Framework group C1[X(C6H9O3)]
Deg. of freedom 48

Full point group C1 NOp 1
Largest Abelian subgroup C1 NOp 1
Largest concise Abelian subgroup C1 NOp 1
Standard orientation:

Center Number	Atomic Number	Atomic Type	Coordinates (Angstroms) X	Y	Z
1	6	0	2.239783	-1.191366	0.089534
2	6	0	0.855874	-1.200725	0.090661
3	6	0	0.103276	-0.000014	0.000051
4	6	0	0.855928	1.200660	-0.090622
5	6	0	2.239837	1.191230	-0.089575
6	6	0	2.957691	-0.000086	-0.000040
7	1	0	2.773761	-2.135488	0.160346
8	1	0	0.307361	-2.134174	0.159615
9	1	0	0.307455	2.134133	-0.159578
10	1	0	2.773857	2.135324	-0.160435
11	1	0	4.041828	-0.000113	-0.000074
12	8	0	-1.191149	0.000017	0.000105
13	8	0	-2.824764	2.092553	0.032464
14	1	0	-3.337591	1.846389	0.804705
15	1	0	-2.156604	1.355543	-0.039445
16	8	0	-2.825152	-2.092399	-0.032578
17	1	0	-2.156952	-1.355406	0.038921
18	1	0	-3.338927	-1.845773	-0.804040

Rotational constants (GHZ): 2.1100979 0.8611089 0.6142565

Thymol

Electronic Energy -617.499726
Stoichiometry C10H18O3
Framework group C1[X(C10H18O3)]
Deg. of freedom 87
Full point group C1 NOp 1
Largest Abelian subgroup C1 NOp 1
Largest concise Abelian subgroup C1 NOp 1
Standard orientation:

Center Number	Atomic Number	Atomic Type	Coordinates (Angstroms) X	Y	Z
1	6	0	-0.158235	2.186971	-0.296881
2	1	0	-0.954110	1.966721	-1.013079
3	6	0	0.659901	0.918621	-0.151881
4	6	0	2.022711	0.916996	0.116129
5	6	0	0.035212	-0.329051	-0.256104
6	6	0	2.743388	-0.261066	0.272971
7	1	0	2.546027	1.861523	0.201744
8	6	0	0.746618	-1.510650	-0.102352
9	6	0	2.112986	-1.493599	0.161881
10	1	0	3.806330	-0.216292	0.481160
11	1	0	0.218718	-2.455973	-0.185447
12	6	0	0.638913	3.371957	-0.840523
13	1	0	-0.028266	4.215831	-1.030955
14	1	0	1.396041	3.713844	-0.130238
15	1	0	1.141948	3.120420	-1.776404
16	6	0	-0.824424	2.555201	1.035730
17	1	0	-1.485725	3.417699	0.917184
18	1	0	-1.413819	1.724340	1.428764
19	1	0	-0.069398	2.806814	1.785497
20	6	0	2.877844	-2.782596	0.301248
21	1	0	2.335678	-3.501958	0.918543
22	1	0	3.042434	-3.247875	-0.674687
23	1	0	3.854635	-2.614253	0.756614
24	8	0	-1.307457	-0.353020	-0.514643
25	1	0	-1.628633	-1.267484	-0.650891
26	8	0	-3.855804	-0.307367	0.617610
27	1	0	-2.982706	0.057343	0.395515
28	1	0	-4.086027	0.019953	1.490038
29	8	0	-2.840471	-2.590422	-0.492679
30	1	0	-3.317975	-3.019810	-1.206359

31	1	0	-3.474773	-2.011088	-0.030611

Rotational constants (GHZ): 0.6896704 0.5733312 0.3373845

Thymolate
Electronic Energy -617.030020
Stoichiometry C10H17O3(1-)
Framework group C1[X(C10H17O3)]
Deg. of freedom 84
Full point group C1 NOp 1
Largest Abelian subgroup C1 NOp 1
Largest concise Abelian subgroup C1 NOp 1
Standard orientation:

Center Number	Atomic Number	Atomic Type	Coordinates (Angstroms) X	Y	Z
1	6	0	0.862433	1.904108	-0.159855
2	1	0	1.602774	1.399736	-0.789027
3	6	0	-0.339018	0.984134	-0.102912
4	6	0	-1.645907	1.437860	-0.126542
5	6	0	-0.089182	-0.422321	0.007852
6	6	0	-2.741878	0.576563	-0.045521
7	1	0	-1.835727	2.503481	-0.212284
8	6	0	-1.219973	-1.270677	0.086089
9	6	0	-2.522922	-0.793665	0.060583
10	1	0	-3.752210	0.972521	-0.065383
11	1	0	-1.031815	-2.336753	0.179402
12	6	0	1.495593	2.060354	1.229205
13	1	0	2.421198	2.643685	1.178046
14	1	0	0.804027	2.571649	1.907392
15	1	0	1.732330	1.081647	1.646784
16	6	0	0.578715	3.274348	-0.773460
17	1	0	1.509196	3.840301	-0.878333
18	1	0	0.121468	3.184532	-1.762275
19	1	0	-0.093354	3.868385	-0.145988
20	6	0	-3.683726	-1.756595	0.119084
21	1	0	-3.845731	-2.242323	-0.848921
22	1	0	-3.507232	-2.547560	0.852647
23	1	0	-4.609972	-1.243627	0.389883
24	8	0	1.116022	-0.889059	0.033874
25	8	0	2.044314	-3.334390	-0.139312
26	1	0	1.852010	-3.564171	-1.050608
27	1	0	1.587865	-2.449875	-0.013080
28	8	0	3.865582	-0.921312	-0.140614
29	1	0	2.940080	-0.611510	-0.090940
30	1	0	3.732937	-1.876693	-0.096032

Rotational constants (GHZ): 0.7430395 0.5610521 0.3376507

Carvacrol
Electronic Energy -617.500272
Stoichiometry C10H18O3
Framework group C1[X(C10H18O3)]
Deg. of freedom 87
Full point group C1 NOp 1
Largest Abelian subgroup C1 NOp 1
Largest concise Abelian subgroup C1 NOp 1
Standard orientation:

Center Number	Atomic Number	Atomic Type	Coordinates (Angstroms) X	Y	Z
1	6	0	-0.591979	1.517782	0.024320
2	6	0	-0.663210	0.146155	-0.223540
3	6	0	0.483106	-0.641015	-0.252171
4	6	0	1.739986	-0.091885	-0.030927
5	6	0	1.821796	1.277491	0.222554
6	6	0	0.673987	2.055637	0.247374
7	1	0	2.783082	1.744620	0.401518
8	8	0	-1.849588	-0.487381	-0.465683
9	1	0	-2.639644	0.067751	-0.309808

10	8	0	-3.587989	-2.533687	0.317161
11	1	0	-3.753565	-3.304974	-0.233593
12	1	0	-2.751428	-2.149084	0.007030
13	8	0	-4.436774	0.085013	0.118619
14	1	0	-4.485696	-0.887908	0.172467
15	1	0	-5.125672	0.363353	-0.492517
16	1	0	0.756413	3.119445	0.443631
17	1	0	0.373592	-1.701589	-0.451144
18	6	0	-1.827916	2.375284	0.039543
19	1	0	-2.337819	2.364220	-0.928509
20	1	0	-2.551061	2.038854	0.787084
21	1	0	-1.571234	3.409788	0.266282
22	6	0	2.976998	-0.969025	-0.066023
23	1	0	2.640018	-1.987800	-0.279959
24	6	0	3.935958	-0.552651	-1.185870
25	1	0	4.788952	-1.234209	-1.231643
26	1	0	3.438368	-0.565004	-2.158019
27	1	0	4.323336	0.455601	-1.018299
28	6	0	3.695011	-0.992365	1.287060
29	1	0	3.024768	-1.318776	2.085213
30	1	0	4.546117	-1.677090	1.256215
31	1	0	4.073861	-0.001220	1.549346

Rotational constants (GHZ): 1.1703831 0.3521736 0.2903455

Carvacrolate
Electronic Energy -617.031943
Stoichiometry C10H17O3(1-)
Framework group C1[X(C10H17O3)]
Deg. of freedom 84
Full point group C1 NOp 1
Largest Abelian subgroup C1 NOp 1
Largest concise Abelian subgroup C1 NOp 1
Standard orientation:

Center Number	Atomic Number	Atomic Type	Coordinates (Angstroms) X	Y	Z
1	6	0	-0.725771	1.583241	-0.105543
2	6	0	-0.842803	0.185257	-0.322100
3	6	0	0.340987	-0.583492	-0.270756
4	6	0	1.591726	-0.031685	-0.018227
5	6	0	1.682670	1.344569	0.193109
6	6	0	0.529138	2.123059	0.145085
7	1	0	2.639773	1.813042	0.392300
8	8	0	-2.004727	-0.370043	-0.570422
9	8	0	-4.263744	-0.374236	0.778912
10	1	0	-4.895321	0.260337	0.430535
11	1	0	-3.416660	-0.225313	0.276293
12	8	0	-2.816938	-2.867050	-0.220166
13	1	0	-2.375495	-1.995257	-0.403881
14	1	0	-3.636594	-2.591778	0.205735
15	1	0	0.251260	-1.653082	-0.440265
16	1	0	0.609334	3.194616	0.307886
17	6	0	-1.955420	2.446157	-0.160587
18	1	0	-2.460044	2.361529	-1.127663
19	1	0	-2.688642	2.154189	0.596616
20	1	0	-1.703016	3.496061	0.000479
21	6	0	2.822782	-0.920305	0.021936
22	1	0	2.483601	-1.943480	-0.168744
23	6	0	3.495524	-0.905853	1.398302
24	1	0	2.794433	-1.196986	2.183827
25	1	0	4.340035	-1.599808	1.422305
26	1	0	3.875432	0.090293	1.639574
27	6	0	3.828359	-0.555324	-1.074897
28	1	0	4.675250	-1.246760	-1.067750
29	1	0	3.366040	-0.595010	-2.063853
30	1	0	4.220725	0.454300	-0.927918

Rotational constants (GHZ): 1.0618176 0.3938853 0.3139844

2,3-dimethylphenol
Electronic Energy -538.900113
Stoichiometry C8H14O3
Framework group C1[X(C8H14O3)]
Deg. of freedom 69
Full point group C1 NOp 1
Largest Abelian subgroup C1 NOp 1
Largest concise Abelian subgroup C1 NOp 1
Standard orientation:

Center Number	Atomic Number	Atomic Type	Coordinates (Angstroms) X	Y	Z
1	6	0	0.828924	0.559614	-0.086449
2	6	0	-0.023217	-0.541199	-0.219368
3	6	0	0.455558	-1.844796	-0.179673
4	6	0	1.810189	-2.064117	0.000310
5	6	0	2.674719	-0.987081	0.138756
6	6	0	2.199723	0.322804	0.096880
7	1	0	2.192958	-3.077307	0.033679
8	1	0	3.734910	-1.161463	0.280514
9	8	0	-1.367754	-0.391449	-0.413541
10	1	0	-1.694121	0.523529	-0.307047
11	8	0	-3.935569	-1.061377	0.462042
12	1	0	-4.514793	-1.636808	-0.046942
13	1	0	-3.033313	-1.226284	0.141311
14	8	0	-3.180007	1.568372	0.096303
15	1	0	-3.760904	0.792436	0.207193
16	1	0	-3.613779	2.150694	-0.534768
17	1	0	-0.242506	-2.665785	-0.289345
18	6	0	0.249994	1.948711	-0.156677
19	1	0	-0.194355	2.143378	-1.137875
20	1	0	-0.538057	2.095970	0.586550
21	1	0	1.005027	2.711223	0.015019
22	6	0	3.175099	1.463090	0.245823
23	1	0	3.169864	2.116374	-0.630031
24	1	0	2.941010	2.084560	1.113618
25	1	0	4.188763	1.082968	0.372088

Rotational constants (GHZ): 1.6299101 0.5710850 0.4321183

2,3-dimethylphenolate
Electronic Energy -538.432082
Stoichiometry C8H13O3(1-)
Framework group C1[X(C8H13O3)]
Deg. of freedom 66
Full point group C1 NOp 1
Largest Abelian subgroup C1 NOp 1
Largest concise Abelian subgroup C1 NOp 1
Standard orientation:

Center Number	Atomic Number	Atomic Type	Coordinates (Angstroms) X	Y	Z
1	6	0	-0.119726	1.737411	0.138363
2	6	0	0.215543	0.374584	0.291781
3	6	0	-0.830866	-0.583561	0.184358
4	6	0	-2.138579	-0.172101	-0.090506
5	6	0	-2.431710	1.185632	-0.246642
6	6	0	-1.420170	2.130991	-0.127238
7	1	0	0.673186	2.472741	0.228153
8	1	0	-3.448670	1.494692	-0.461212
9	8	0	1.451246	0.005231	0.529683
10	8	0	3.661471	1.197171	-0.202264
11	1	0	4.037919	1.712138	0.516091
12	1	0	2.775402	0.865842	0.125413
13	8	0	3.133687	-1.765610	-0.517362
14	1	0	2.377372	-1.255839	-0.132009
15	1	0	3.801505	-1.076806	-0.617600
16	1	0	-1.649113	3.185416	-0.245721
17	6	0	-0.482373	-2.037999	0.360555
18	1	0	-0.174393	-2.500672	-0.584454

19	1	0	0.355143	-2.144595	1.050594
20	1	0	-1.322517	-2.618070	0.743563
21	6	0	-3.251992	-1.182060	-0.228963
22	1	0	-3.026783	-1.930698	-0.993033
23	1	0	-3.425117	-1.724914	0.704675
24	1	0	-4.185923	-0.690961	-0.505169

Rotational constants (GHZ): 1.5696057 0.6212491 0.4605379

2,4-dimethylphenol
Electronic Energy -538.901371
Stoichiometry C8H14O3
Framework group C1[X(C8H14O3)]
Deg. of freedom 69
Full point group C1 NOp 1
Largest Abelian subgroup C1 NOp 1
Largest concise Abelian subgroup C1 NOp 1
Standard orientation:

Center Number	Atomic Number	Atomic Type	Coordinates (Angstroms) X	Y	Z
1	6	0	0.534371	0.948985	-0.086103
2	6	0	-0.087732	-0.287731	-0.274805
3	6	0	0.657996	-1.458155	-0.288465
4	6	0	2.032267	-1.412458	-0.105779
5	6	0	2.686450	-0.200481	0.097294
6	6	0	1.914826	0.959030	0.098886
7	1	0	2.600745	-2.336005	-0.121968
8	8	0	-1.435921	-0.402876	-0.478576
9	1	0	-1.937907	0.417481	-0.302066
10	8	0	-3.779820	-1.594613	0.469676
11	1	0	-4.264906	-2.245125	-0.047236
12	1	0	-2.880701	-1.566189	0.101903
13	8	0	-3.554970	1.145346	0.219248
14	1	0	-3.975176	0.269858	0.312782
15	1	0	-4.119259	1.661995	-0.364014
16	1	0	2.404279	1.917138	0.244737
17	1	0	0.148693	-2.401598	-0.446067
18	6	0	-0.260436	2.226374	-0.098062
19	1	0	-0.760551	2.377955	-1.059339
20	1	0	-1.036077	2.232424	0.672057
21	1	0	0.390009	3.082910	0.076422
22	6	0	4.173358	-0.143416	0.332515
23	1	0	4.601106	0.783762	-0.052838
24	1	0	4.406257	-0.191280	1.400314
25	1	0	4.682575	-0.979067	-0.150365

Rotational constants (GHZ): 1.9426732 0.4958120 0.4050856

2,4-dimethylphenolate
Electronic Energy -538.432738
Stoichiometry C8H13O3(1-)
Framework group C1[X(C8H13O3)]
Deg. of freedom 66
Full point group C1 NOp 1
Largest Abelian subgroup C1 NOp 1
Largest concise Abelian subgroup C1 NOp 1
Standard orientation:

Center Number	Atomic Number	Atomic Type	Coordinates (Angstroms) X	Y	Z
1	6	0	0.421840	-1.374726	0.215435
2	6	0	-0.264140	-0.149590	0.309207
3	6	0	0.511523	1.034979	0.179145
4	6	0	1.880142	0.942538	-0.032344
5	6	0	2.556823	-0.277216	-0.128589
6	6	0	1.794956	-1.432631	0.001659
7	1	0	-0.150767	-2.291166	0.318113
8	1	0	2.449411	1.864840	-0.125976
9	8	0	-1.559621	-0.091026	0.517241

10	8	0	-3.370308	-1.849168	-0.158951
11	1	0	-3.615886	-2.398933	0.589651
12	1	0	-2.606814	-1.278983	0.151301
13	8	0	-3.617154	1.128899	-0.642086
14	1	0	-2.763998	0.848961	-0.224403
15	1	0	-4.087070	0.289090	-0.707712
16	1	0	2.279980	-2.402648	-0.063009
17	6	0	-0.173150	2.369319	0.280484
18	1	0	-0.929492	2.492488	-0.500074
19	1	0	-0.696188	2.476602	1.235245
20	1	0	0.544664	3.186573	0.189302
21	6	0	4.044343	-0.325795	-0.371821
22	1	0	4.307830	0.068389	-1.358100
23	1	0	4.594865	0.264763	0.365848
24	1	0	4.416112	-1.350880	-0.318872

Rotational constants (GHZ): 1.7768559 0.5431962 0.4310042

2,6-diisopropylphenol

Electronic Energy -696.094271
Stoichiometry C12H22O3
Framework group C1[X(C12H22O3)]
Deg. of freedom 105
Full point group C1 NOp 1
Largest Abelian subgroup C1 NOp 1
Largest concise Abelian subgroup C1 NOp 1
Standard orientation:

Center Number	Atomic Number	Atomic Type	Coordinates (Angstroms) X	Y	Z
1	6	0	1.117396	-1.021888	-0.079110
2	6	0	-0.059336	-0.266160	-0.133653
3	6	0	-1.329336	-0.855185	-0.117149
4	6	0	-1.401326	-2.240179	-0.035297
5	6	0	-0.253509	-3.015761	0.023384
6	6	0	0.989566	-2.405927	-0.001396
7	1	0	-2.369126	-2.724927	-0.026571
8	1	0	-0.328227	-4.094759	0.082251
9	8	0	-0.028055	1.102016	-0.199083
10	1	0	0.856668	1.476925	-0.359194
11	8	0	-0.845787	3.742564	-0.106490
12	1	0	-1.323768	4.110059	0.640522
13	1	0	-0.937047	2.776469	-0.053148
14	8	0	1.790018	3.110565	-0.526348
15	1	0	0.979678	3.636725	-0.389644
16	1	0	2.242963	3.489411	-1.283117
17	1	0	1.879523	-3.020594	0.037492
18	6	0	-2.562322	0.028687	-0.162134
19	1	0	-2.332222	0.856323	-0.839066
20	6	0	-2.850961	0.624183	1.223221
21	1	0	-1.977828	1.140753	1.625810
22	1	0	-3.682729	1.332694	1.176710
23	1	0	-3.121409	-0.165945	1.928736
24	6	0	-3.805197	-0.671816	-0.708622
25	1	0	-4.617238	0.049475	-0.825344
26	1	0	-3.615120	-1.126653	-1.682882
27	1	0	-4.162477	-1.452961	-0.032885
28	6	0	2.473554	-0.336215	-0.057428
29	1	0	2.429318	0.529117	-0.727970
30	6	0	2.794371	0.187419	1.349601
31	1	0	3.736090	0.741235	1.350524
32	1	0	2.015256	0.854872	1.720588
33	1	0	2.885139	-0.647045	2.049844
34	6	0	3.614746	-1.212547	-0.573334
35	1	0	3.393276	-1.624325	-1.560020
36	1	0	4.529934	-0.621354	-0.649150
37	1	0	3.824061	-2.044314	0.103391

Rotational constants (GHZ): 0.5782226 0.4700602 0.2833303

2,6-diisopropylphenolate
Electronic Energy -695.625109
Stoichiometry C12H21O3(1-)
Framework group C1[X(C12H21O3)]
Deg. of freedom 102
Full point group C1 NOp 1
Largest Abelian subgroup C1 NOp 1
Largest concise Abelian subgroup C1 NOp 1
Standard orientation:

Center Number	Atomic Number	Atomic Type	Coordinates (Angstroms) X	Y	Z
1	6	0	1.358986	-0.756285	-0.046465
2	6	0	0.069902	-0.140274	-0.060458
3	6	0	-1.075140	-0.989154	-0.116270
4	6	0	-0.913596	-2.367445	-0.131883
5	6	0	0.343743	-2.959020	-0.105504
6	6	0	1.466768	-2.138370	-0.066545
7	1	0	-1.791396	-3.005336	-0.173193
8	1	0	0.448086	-4.038291	-0.124417
9	8	0	-0.037165	1.153091	-0.028665
10	8	0	0.475970	3.387682	-1.575367
11	1	0	-0.276447	3.771058	-1.108414
12	1	0	0.496613	2.494700	-1.174380
13	8	0	-1.556722	3.193460	0.641465
14	1	0	-1.095562	2.337698	0.420597
15	1	0	-1.091015	3.512232	1.417104
16	1	0	2.450004	-2.597917	-0.057373
17	6	0	-2.442748	-0.336507	-0.121060
18	1	0	-2.314434	0.637401	-0.599861
19	6	0	-3.500267	-1.109528	-0.910583
20	1	0	-3.747593	-2.064541	-0.436667
21	1	0	-4.425839	-0.528872	-0.971796
22	1	0	-3.160930	-1.318845	-1.927934
23	6	0	-2.930358	-0.075407	1.309879
24	1	0	-2.195516	0.509126	1.864194
25	1	0	-3.872849	0.481806	1.311803
26	1	0	-3.086065	-1.022011	1.838593
27	6	0	2.564827	0.159850	0.019909
28	1	0	2.342548	1.010051	-0.631513
29	6	0	3.867579	-0.475265	-0.463948
30	1	0	4.664041	0.274433	-0.491037
31	1	0	4.200172	-1.278790	0.200896
32	1	0	3.760883	-0.893502	-1.468011
33	6	0	2.739383	0.727475	1.434553
34	1	0	3.553397	1.460086	1.467975
35	1	0	1.819589	1.219667	1.750628
36	1	0	2.971170	-0.074433	2.143591

Rotational constants (GHZ): 0.5861723 0.4602902 0.2994736

2-methyl-4-*terz*-butylphenol
Electronic Energy -656.796779
Stoichiometry C11H20O3
Framework group C1[X(C11H20O3)]
Deg. of freedom 96
Full point group C1 NOp 1
Largest Abelian subgroup C1 NOp 1
Largest concise Abelian subgroup C1 NOp 1
Standard orientation:

Center Number	Atomic Number	Atomic Type	Coordinates (Angstroms) X	Y	Z
1	6	0	0.984974	-1.396293	-0.212136
2	6	0	-0.396893	-1.450016	-0.349260
3	6	0	-1.150512	-0.288837	-0.316159
4	6	0	-0.527041	0.951737	-0.150108
5	6	0	0.856132	0.968622	-0.011581
6	6	0	1.645864	-0.183872	-0.037279
7	1	0	1.537229	-2.325605	-0.242637

8	1	0	-0.902025	-2.399587	-0.482067
9	1	0	1.328889	1.935902	0.118544
10	8	0	-2.503884	-0.410538	-0.472101
11	1	0	-3.003678	0.406914	-0.277129
12	8	0	-4.611574	1.120304	0.293096
13	1	0	-5.192453	1.643126	-0.267925
14	1	0	-5.025677	0.241617	0.383924
15	8	0	-4.821485	-1.624368	0.509252
16	1	0	-3.930225	-1.587614	0.123592
17	1	0	-5.315525	-2.267691	-0.008161
18	6	0	3.166499	-0.073563	0.121399
19	6	0	3.741448	0.796127	-1.009811
20	1	0	3.322697	1.804097	-0.997790
21	1	0	4.826249	0.883638	-0.905101
22	1	0	3.529010	0.353867	-1.986293
23	6	0	3.498130	0.575371	1.476176
24	1	0	3.108859	-0.026435	2.301138
25	1	0	4.581178	0.660543	1.599237
26	1	0	3.073041	1.577286	1.559670
27	6	0	3.852983	-1.442216	0.067110
28	1	0	3.509122	-2.100993	0.867864
29	1	0	3.681771	-1.944874	-0.887588
30	1	0	4.931220	-1.312912	0.184619
31	6	0	-1.329104	2.224706	-0.133522
32	1	0	-1.866243	2.371817	-1.075309
33	1	0	-2.074995	2.227626	0.665533
34	1	0	-0.677784	3.085494	0.014934

Rotational constants (GHZ): 1.3749873 0.2733280 0.2436996

2-methyl-4-*terz*-butylphenolate
Electronic Energy -656.328185
Stoichiometry C11H19O3(1-)
Framework group C1[X(C11H19O3)]
Deg. of freedom 93
Full point group C1 NOp 1
Largest Abelian subgroup C1 NOp 1
Largest concise Abelian subgroup C1 NOp 1
Standard orientation:

Center Number	Atomic Number	Atomic Type	Coordinates (Angstroms) X	Y	Z
1	6	0	-0.807627	1.025580	0.086940
2	6	0	0.570327	1.072407	0.236687
3	6	0	1.310660	-0.135982	0.345768
4	6	0	0.575425	-1.332812	0.292213
5	6	0	-0.807955	-1.344862	0.139541
6	6	0	-1.541588	-0.167128	0.032539
7	1	0	-1.332629	1.973472	0.009294
8	1	0	1.116433	-2.270373	0.375711
9	1	0	-1.307599	-2.305379	0.106545
10	8	0	2.614765	-0.122162	0.497152
11	8	0	4.350057	-1.938726	-0.221469
12	1	0	4.596441	-2.495454	0.521710
13	1	0	3.613143	-1.344494	0.106825
14	8	0	4.682556	1.041832	-0.697101
15	1	0	3.830205	0.786341	-0.262312
16	1	0	5.124040	0.188199	-0.778641
17	6	0	-3.064892	-0.129142	-0.134443
18	6	0	-3.700982	0.633782	1.040762
19	1	0	-3.332448	1.659732	1.100463
20	1	0	-4.788329	0.674737	0.927385
21	1	0	-3.476818	0.140064	1.989811
22	6	0	-3.430021	0.584502	-1.447903
23	1	0	-3.008136	0.055722	-2.306463
24	1	0	-4.516105	0.623898	-1.573210
25	1	0	-3.054354	1.609411	-1.466657
26	6	0	-3.676542	-1.533592	-0.173477
27	1	0	-3.289298	-2.120890	-1.009420
28	1	0	-3.482715	-2.085611	0.749211
29	1	0	-4.760038	-1.458293	-0.294108

30	6	0	1.303223	2.383917	0.290883
31	1	0	1.867793	2.486082	1.222232
32	1	0	2.030843	2.471075	-0.521206
33	1	0	0.610380	3.224187	0.217117

Rotational constants (GHZ): 1.2856518 0.2918145 0.2562683

4-bromothymol

Electronic Energy -3191.144763
Stoichiometry C10H17BrO3
Framework group C1[X(C10H17BrO3)]
Deg. of freedom 87
Full point group C1 NOp 1
Largest Abelian subgroup C1 NOp 1
Largest concise Abelian subgroup C1 NOp 1

Standard orientation:

Center Number	Atomic Number	Atomic Type	Coordinates (Angstroms) X	Y	Z
1	6	0	-1.414478	2.155394	-0.298382
2	1	0	-2.259062	1.911554	-0.947887
3	6	0	-0.543283	0.915975	-0.235994
4	6	0	0.837976	0.974774	-0.103782
5	6	0	-1.126362	-0.355740	-0.276675
6	6	0	1.604419	-0.177968	-0.014781
7	1	0	1.331928	1.936245	-0.072032
8	6	0	-0.348322	-1.503534	-0.190529
9	6	0	1.037144	-1.447052	-0.056272
10	1	0	-0.834354	-2.472490	-0.229454
11	6	0	-0.709127	3.373265	-0.892996
12	1	0	-1.423110	4.190615	-1.014441
13	1	0	0.091470	3.737342	-0.244217
14	1	0	-0.279277	3.151584	-1.872227
15	6	0	-1.979435	2.483424	1.090413
16	1	0	-2.677232	3.322499	1.033853
17	1	0	-2.507508	1.629813	1.519440
18	1	0	-1.173094	2.759006	1.775721
19	6	0	1.842784	-2.711330	0.033982
20	1	0	1.193011	-3.584332	-0.017895
21	1	0	2.569300	-2.775507	-0.778960
22	1	0	2.403641	-2.756118	0.970045
23	8	0	-2.477711	-0.436240	-0.409377
24	1	0	-2.791678	-1.367329	-0.430201
25	8	0	-5.169899	-0.319857	0.504931
26	1	0	-4.298015	0.087608	0.391387
27	1	0	-5.504517	-0.011103	1.352737
28	8	0	-3.937691	-2.680254	-0.179578
29	1	0	-4.291909	-3.199594	-0.908257
30	1	0	-4.644864	-2.069081	0.101979
31	35	0	3.501994	0.021624	0.163220

Rotational constants (GHZ): 0.6975886 0.2396062 0.1852995

4-bromothymolate

Electronic Energy -3190.678091
Stoichiometry C10H16BrO3(1-)
Framework group C1[X(C10H16BrO3)]
Deg. of freedom 84
Full point group C1 NOp 1
Largest Abelian subgroup C1 NOp 1
Largest concise Abelian subgroup C1 NOp 1

Standard orientation:

Center Number	Atomic Number	Atomic Type	Coordinates (Angstroms) X	Y	Z
1	6	0	1.569554	2.000982	-0.229044
2	1	0	2.412434	1.704658	-0.860239
3	6	0	0.635787	0.808458	-0.185622
4	6	0	-0.739576	0.950018	-0.090617
5	6	0	1.193115	-0.503635	-0.213355

6	6	0	-1.582570	-0.152597	-0.020160
7	1	0	-1.176684	1.940388	-0.072340
8	6	0	0.295593	-1.591582	-0.145646
9	6	0	-1.085144	-1.451689	-0.047027
10	1	0	0.718956	-2.590768	-0.172975
11	6	0	2.131749	2.301942	1.167022
12	1	0	2.879535	3.099118	1.124329
13	1	0	1.331296	2.626097	1.838849
14	1	0	2.602272	1.417829	1.599865
15	6	0	0.951452	3.260009	-0.835991
16	1	0	1.711580	4.038450	-0.937567
17	1	0	0.532265	3.066020	-1.826111
18	1	0	0.155432	3.667394	-0.206732
19	6	0	-1.968607	-2.666899	0.022036
20	1	0	-2.679107	-2.690807	-0.807691
21	1	0	-1.369902	-3.576965	-0.015587
22	1	0	-2.554930	-2.681508	0.943904
23	8	0	2.478463	-0.698197	-0.307046
24	8	0	4.040636	-2.765211	0.067123
25	1	0	4.037266	-3.357313	-0.689563
26	1	0	3.335338	-2.080072	-0.111042
27	8	0	4.992522	0.059711	0.197743
28	1	0	4.022205	0.025207	0.034681
29	1	0	5.216497	-0.879774	0.204860
30	35	0	-3.476444	0.164903	0.107464

Rotational constants (GHZ): 0.7283951 0.2433800 0.1884423

4-chlorothymol

Electronic Energy -1077.127229

Stoichiometry C10H17ClO3

Framework group C1[X(C10H17ClO3)]

Deg. of freedom 87

Full point group C1 NOp 1

Largest Abelian subgroup C1 NOp 1

Largest concise Abelian subgroup C1 NOp 1

Standard orientation:

Center Number	Atomic Number	Atomic Type	Coordinates (Angstroms) X	Y	Z
1	6	0	0.116745	-1.448408	-0.272339
2	6	0	-0.627412	-0.275749	-0.241060
3	6	0	-0.013073	0.965250	-0.035235
4	6	0	1.365491	0.968016	0.132269
5	6	0	2.097948	-0.208459	0.094091
6	6	0	1.499650	-1.446389	-0.107479
7	1	0	-0.395332	-2.391750	-0.428069
8	1	0	1.887519	1.900342	0.299118
9	8	0	-1.978846	-0.300304	-0.407673
10	1	0	-2.309372	-1.216751	-0.537606
11	6	0	-0.843561	2.234115	-0.034045
12	1	0	-1.785963	1.992638	0.465381
13	6	0	-1.181487	2.656296	-1.470328
14	1	0	-1.680511	1.851654	-2.012074
15	1	0	-1.841604	3.527306	-1.468416
16	1	0	-0.271026	2.923176	-2.014084
17	6	0	-0.200008	3.390314	0.728865
18	1	0	-0.898476	4.227675	0.786729
19	1	0	0.068112	3.103240	1.747988
20	1	0	0.701295	3.754836	0.229394
21	6	0	2.280189	-2.728691	-0.145926
22	1	0	2.819317	-2.890921	0.790000
23	1	0	1.616529	-3.576865	-0.310448
24	1	0	3.023900	-2.714301	-0.945723
25	8	0	-4.347132	-0.541065	1.191309
26	1	0	-3.532215	-0.081817	0.936262
27	1	0	-4.422496	-0.448625	2.146281
28	8	0	-3.352299	-2.615521	-0.309456
29	1	0	-3.883362	-2.988661	-1.019996
30	1	0	-3.962621	-2.127749	0.275560
31	17	0	3.842685	-0.107416	0.316082

Rotational constants (GHZ): 0.6530656 0.3494788 0.2472950

4-chlorothymolate

Electronic Energy -1076.660134
Stoichiometry C10H16ClO3(1-)
Framework group C1[X(C10H16ClO3)]
Deg. of freedom 84
Full point group C1 NOp 1
Largest Abelian subgroup C1 NOp 1
Largest concise Abelian subgroup C1 NOp 1
Standard orientation:

Center Number	Atomic Number	Atomic Type	Coordinates (Angstroms) X	Y	Z
1	6	0	0.307827	-1.545699	-0.140446
2	6	0	-0.623050	-0.487534	-0.225263
3	6	0	-0.110340	0.841872	-0.181345
4	6	0	1.257307	1.028934	-0.054568
5	6	0	2.132781	-0.045106	0.031536
6	6	0	1.680046	-1.358892	-0.010052
7	1	0	-0.080683	-2.558520	-0.179692
8	1	0	1.665186	2.031720	-0.022967
9	8	0	-1.900235	-0.723854	-0.348999
10	6	0	-1.081257	2.003421	-0.241570
11	1	0	-1.909809	1.674169	-0.875124
12	6	0	-0.498375	3.275778	-0.855854
13	1	0	-0.066274	3.085714	-1.841169
14	1	0	-1.281412	4.029425	-0.969993
15	1	0	0.280193	3.713400	-0.225082
16	6	0	-1.662128	2.298783	1.148019
17	1	0	-2.433539	3.072412	1.093643
18	1	0	-2.108617	1.404425	1.585473
19	1	0	-0.876808	2.652479	1.822813
20	6	0	2.611400	-2.536443	0.076548
21	1	0	2.052371	-3.470690	0.024478
22	1	0	3.340466	-2.528503	-0.737335
23	1	0	3.177948	-2.528618	1.010867
24	8	0	-3.357283	-2.872086	-0.023224
25	1	0	-3.354626	-3.424497	-0.809346
26	1	0	-2.689061	-2.146611	-0.188513
27	8	0	-4.421865	-0.113705	0.301856
28	1	0	-3.459419	-0.093590	0.094982
29	1	0	-4.601693	-1.062227	0.269111
30	17	0	3.864446	0.292705	0.189927

Rotational constants (GHZ): 0.7259885 0.3459574 0.2447423

4-methoxyphenol

Electronic Energy -574.807744
Stoichiometry C7H12O4
Framework group C1[X(C7H12O4)]
Deg. of freedom 63
Full point group C1 NOp 1
Largest Abelian subgroup C1 NOp 1
Largest concise Abelian subgroup C1 NOp 1
Standard orientation:

Center Number	Atomic Number	Atomic Type	Coordinates (Angstroms) X	Y	Z
1	6	0	1.998538	1.078159	-0.532957
2	6	0	0.761992	1.673956	-0.338591
3	6	0	-0.328659	0.910020	0.072269
4	6	0	-0.166538	-0.457281	0.286248
5	6	0	1.076136	-1.048192	0.094215
6	6	0	2.158795	-0.284851	-0.315222
7	1	0	2.844372	1.670002	-0.862830
8	1	0	0.626613	2.735012	-0.510031
9	1	0	-1.014770	-1.052558	0.602247
10	1	0	1.208192	-2.111429	0.256275

11	8	0	-1.518299	1.538891	0.244370
12	1	0	-2.218274	0.909990	0.532762
13	8	0	-3.462662	-0.147660	1.040470
14	1	0	-3.942502	0.070192	1.845475
15	1	0	-4.130573	-0.446212	0.388833
16	8	0	-5.307738	-0.992370	-0.803193
17	1	0	-5.420981	-0.474162	-1.607273
18	1	0	-5.284519	-1.912961	-1.085756
19	8	0	3.376596	-0.893443	-0.548918
20	6	0	4.319312	-0.749680	0.510041
21	1	0	5.227881	-1.260659	0.196441
22	1	0	4.542446	0.304441	0.697094
23	1	0	3.941487	-1.207783	1.428910

Rotational constants (GHZ): 2.2273278 0.3515120 0.3251593

4-methoxyphenolate

Electronic Energy -574.339791

Stoichiometry C7H11O4(1-)

Framework group C1[X(C7H11O4)]

Deg. of freedom 60

Full point group C1 NOp 1

Largest Abelian subgroup C1 NOp 1

Largest concise Abelian subgroup C1 NOp 1

Standard orientation:

Center Number	Atomic Number	Atomic Type	Coordinates (Angstroms) X	Y	Z
1	6	0	1.288625	-1.200115	-0.251506
2	6	0	-0.094862	-1.202231	-0.146444
3	6	0	-0.836437	-0.000702	-0.086279
4	6	0	-0.092468	1.199678	-0.141733
5	6	0	1.290829	1.195281	-0.247947
6	6	0	1.990038	-0.003034	-0.302859
7	1	0	1.834766	-2.136328	-0.301528
8	1	0	-0.632643	-2.143419	-0.112543
9	1	0	-0.628447	2.141800	-0.105463
10	1	0	1.838522	2.130740	-0.295324
11	8	0	-2.145056	0.000383	0.011895
12	8	0	-3.619995	2.167709	0.069207
13	1	0	-3.430007	2.628815	0.890250
14	1	0	-3.019021	1.367089	0.062569
15	8	0	-3.609891	-2.157905	0.288194
16	1	0	-3.015288	-1.362591	0.162615
17	1	0	-3.553986	-2.656291	-0.531152
18	8	0	3.370699	-0.004516	-0.450590
19	6	0	4.086102	0.004939	0.778735
20	1	0	3.850924	-0.882248	1.374926
21	1	0	5.147274	0.003223	0.532657
22	1	0	3.850891	0.900939	1.361538

Rotational constants (GHZ): 1.8252678 0.4605878 0.3789666

4-hydroxybenzonitrile

Electronic Energy -552.532119

Stoichiometry C7H9NO3

Framework group C1[X(C7H9NO3)]

Deg. of freedom 54

Full point group C1 NOp 1

Largest Abelian subgroup C1 NOp 1

Largest concise Abelian subgroup C1 NOp 1

Standard orientation:

Center Number	Atomic Number	Atomic Type	Coordinates (Angstroms) X	Y	Z
1	6	0	1.759995	-1.233893	-0.232874
2	6	0	0.409843	-1.378216	-0.475867
3	6	0	-0.429821	-0.264564	-0.431004
4	6	0	0.094098	0.996088	-0.138719
5	6	0	1.446446	1.139330	0.103743

6	6	0	2.289996	0.027020	0.058814
7	1	0	2.411043	-2.097895	-0.267975
8	1	0	-0.012873	-2.348318	-0.703361
9	1	0	-0.562847	1.856406	-0.102179
10	1	0	1.853004	2.116681	0.329887
11	8	0	-1.740066	-0.454254	-0.676528
12	1	0	-2.254838	0.386072	-0.610490
13	8	0	-3.463509	1.501746	-0.139855
14	1	0	-4.036548	1.932880	-0.781832
15	1	0	-4.002447	0.830490	0.321318
16	8	0	-4.174836	-0.927361	0.897974
17	1	0	-3.316700	-1.270012	0.611282
18	1	0	-4.292257	-1.221905	1.806803
19	6	0	3.687127	0.176790	0.309520
20	7	0	4.813665	0.297033	0.511734

Rotational constants (GHZ): 2.7083813 0.4047179 0.3710987

4-cyanophenolate
Electronic Energy -552.073802
Stoichiometry C7H8NO3(1-)
Framework group C1[X(C7H8NO3)]
Deg. of freedom 51
Full point group C1 NOp 1
Largest Abelian subgroup C1 NOp 1
Largest concise Abelian subgroup C1 NOp 1
Standard orientation:

Center Number	Atomic Number	Atomic Type	Coordinates (Angstroms) X	Y	Z
1	6	0	1.447016	-1.204387	0.093114
2	6	0	0.071867	-1.206699	0.093612
3	6	0	-0.675111	0.000019	0.000032
4	6	0	0.071867	1.206736	-0.093561
5	6	0	1.447016	1.204421	-0.093098
6	6	0	2.161821	0.000016	0.000000
7	1	0	1.987748	-2.140984	0.165912
8	1	0	-0.471543	-2.141407	0.166769
9	1	0	-0.471545	2.141443	-0.166702
10	1	0	1.987749	2.141016	-0.165906
11	8	0	-1.966708	0.000024	0.000046
12	8	0	-3.471564	2.194549	0.005319
13	1	0	-3.883854	2.227486	0.872516
14	1	0	-2.885971	1.392695	0.017955
15	8	0	-3.471313	-2.194689	-0.005406
16	1	0	-2.886030	-1.392607	-0.017900
17	1	0	-3.883738	-2.227517	-0.872544
18	6	0	3.581524	0.000014	-0.000019
19	7	0	4.735409	0.000012	-0.000035

Rotational constants (GHZ): 1.9565927 0.4661451 0.3776052

4-nitrophenol
Electronic Energy -664.810518
Stoichiometry C6H9NO5
Framework group C1[X(C6H9NO5)]
Deg. of freedom 57
Full point group C1 NOp 1
Largest Abelian subgroup C1 NOp 1
Largest concise Abelian subgroup C1 NOp 1
Standard orientation:

Center Number	Atomic Number	Atomic Type	Coordinates (Angstroms) X	Y	Z
1	6	0	1.722766	-1.282841	0.290377
2	6	0	0.446113	-1.790939	0.198724
3	6	0	-0.620756	-0.956366	-0.155473
4	6	0	-0.388138	0.400046	-0.417561
5	6	0	0.889996	0.909955	-0.326560
6	6	0	1.936899	0.066626	0.026638

7	1	0	2.553629	-1.917552	0.563073
8	1	0	0.250042	-2.836409	0.397814
9	1	0	-1.217854	1.038875	-0.691176
10	1	0	1.083135	1.954238	-0.526189
11	8	0	-1.838671	-1.500242	-0.229717
12	1	0	-2.529702	-0.836777	-0.487081
13	8	0	-3.686341	0.267383	-0.909321
14	1	0	-4.152938	0.141603	-1.741503
15	1	0	-4.357514	0.531206	-0.244184
16	8	0	-5.531005	1.015734	0.957263
17	1	0	-5.788023	0.379408	1.633306
18	1	0	-5.399765	1.851083	1.418666
19	7	0	3.279768	0.604067	0.122454
20	8	0	4.188929	-0.150695	0.437380
21	8	0	3.452002	1.791192	-0.115201

Rotational constants (GHZ): 1.9486458 0.3078253 0.2795524

4-nitrophenolate
Electronic Energy -664.352895
Stoichiometry C6H8NO5(1-)
Framework group C1[X(C6H8NO5)]
Deg. of freedom 54
Full point group C1 NOp 1
Largest Abelian subgroup C1 NOp 1
Largest concise Abelian subgroup C1 NOp 1
Standard orientation:

Center Number	Atomic Number	Atomic Type	Coordinates (Angstroms) X	Y	Z
1	6	0	1.041300	1.212285	-0.080860
2	6	0	-0.328324	1.213733	-0.080669
3	6	0	-1.077179	0.000054	0.000103
4	6	0	-0.328328	-1.213632	0.080829
5	6	0	1.041295	-1.212195	0.080912
6	6	0	1.739138	0.000042	-0.000004
7	1	0	1.594664	2.139226	-0.143370
8	1	0	-0.874504	2.147105	-0.143321
9	1	0	-0.874513	-2.146999	0.143518
10	1	0	1.594657	-2.139140	0.143381
11	8	0	-2.358667	0.000057	0.000145
12	8	0	-3.886762	-2.204377	-0.022720
13	1	0	-4.302690	-2.228133	-0.888549
14	1	0	-3.301855	-1.406794	-0.030015
15	8	0	-3.887872	2.203705	0.022319
16	1	0	-3.302381	1.406557	0.029905
17	1	0	-4.299573	2.230603	0.890074
18	7	0	3.161354	0.000035	-0.000056
19	8	0	3.760984	1.074400	-0.073463
20	8	0	3.760979	-1.074335	0.073331

Rotational constants (GHZ): 1.6979030 0.3855860 0.3150706

4-nitrothymol
Electronic Energy -822.006976
Stoichiometry C10H17NO5
Framework group C1[X(C10H17NO5)]
Deg. of freedom 93
Full point group C1 NOp 1
Largest Abelian subgroup C1 NOp 1
Largest concise Abelian subgroup C1 NOp 1
Standard orientation:

Center Number	Atomic Number	Atomic Type	Coordinates (Angstroms) X	Y	Z
1	6	0	-1.203704	0.969686	-0.080492
2	6	0	0.170896	0.922042	-0.166109
3	6	0	0.761906	-0.353707	-0.143441
4	6	0	-0.014991	-1.504887	-0.041525
5	6	0	-1.401234	-1.466220	0.031355

6	6	0	-1.976075	-0.188700	0.009171
7	1	0	-1.717604	1.919204	-0.094775
8	1	0	0.484170	-2.466658	-0.014445
9	8	0	2.101046	-0.428240	-0.226289
10	1	0	2.431486	-1.359019	-0.215593
11	8	0	3.534962	-2.657455	-0.064279
12	1	0	3.697644	-3.262432	-0.795040
13	1	0	4.310369	-2.065712	-0.007170
14	8	0	5.009694	-0.358498	0.076192
15	1	0	4.207191	0.178844	0.118891
16	1	0	5.595504	-0.020960	0.761392
17	7	0	-3.413966	0.000645	0.078043
18	8	0	-3.842875	1.099493	0.407335
19	8	0	-4.148083	-0.935860	-0.202089
20	6	0	1.040673	2.159810	-0.251748
21	1	0	1.877531	1.908771	-0.908930
22	6	0	1.622297	2.501629	1.126954
23	1	0	2.162057	1.654892	1.553943
24	1	0	2.314460	3.343633	1.051423
25	1	0	0.823788	2.780617	1.819828
26	6	0	0.324722	3.369040	-0.849763
27	1	0	-0.113665	3.138291	-1.823091
28	1	0	-0.470507	3.735175	-0.195416
29	1	0	1.034698	4.187471	-0.984570
30	6	0	-2.149667	-2.764406	0.152137
31	1	0	-2.872747	-2.740348	0.967418
32	1	0	-2.708692	-2.982066	-0.758231
33	1	0	-1.444815	-3.575455	0.331877

Rotational constants (GHZ): 0.6696075 0.2953642 0.2127204

4-nitrothymolate
Electronic Energy -821.549876
Stoichiometry C10H16NO5(1-)
Framework group C1[X(C10H16NO5)]
Deg. of freedom 90
Full point group C1 NOp 1
Largest Abelian subgroup C1 NOp 1
Largest concise Abelian subgroup C1 NOp 1
Standard orientation:

Center Number	Atomic Number	Atomic Type	Coordinates (Angstroms) X	Y	Z
1	6	0	-1.088087	0.974478	-0.079347
2	6	0	0.270252	0.824852	-0.110198
3	6	0	0.811658	-0.509022	-0.080436
4	6	0	-0.110981	-1.590614	-0.017031
5	6	0	-1.479965	-1.445083	0.020269
6	6	0	-1.976179	-0.118080	-0.011834
7	1	0	-1.526079	1.961198	-0.107969
8	1	0	0.311727	-2.589047	0.000645
9	8	0	2.071142	-0.720008	-0.114775
10	8	0	3.776872	-2.763354	-0.170500
11	1	0	3.691415	-3.357329	0.580238
12	1	0	3.012950	-2.137142	-0.116290
13	8	0	4.693776	0.024127	-0.135378
14	1	0	3.716302	0.040285	-0.123850
15	1	0	4.867840	-0.927232	-0.121333
16	7	0	-3.362934	0.182767	0.014570
17	8	0	-3.727487	1.364433	-0.042193
18	8	0	-4.188965	-0.730955	0.095425
19	6	0	1.229625	1.995196	-0.151435
20	1	0	2.052768	1.695591	-0.807454
21	6	0	1.826139	2.255604	1.238771
22	1	0	2.282341	1.353157	1.647926
23	1	0	2.592431	3.034066	1.192273
24	1	0	1.047202	2.589307	1.930445
25	6	0	0.623676	3.276618	-0.719894
26	1	0	0.185567	3.112839	-1.707124
27	1	0	-0.154425	3.681681	-0.067252
28	1	0	1.396115	4.043017	-0.815839

29	6	0	-2.324426	-2.688698	0.085463
30	1	0	-2.943553	-2.705609	0.982715
31	1	0	-3.007940	-2.753864	-0.761498
32	1	0	-1.677092	-3.565736	0.085777

Rotational constants (GHZ): 0.6941591 0.3012779 0.2174264

- $2H_2O$/ B3LYP 6-311G+dp/SMD

H_2O

Electronic Energy -229.353562
Stoichiometry H6O3
Framework group C1[X(H6O3)]
Deg. of freedom 21
Full point group C1 NOp 1
Largest Abelian subgroup C1 NOp 1
Largest concise Abelian subgroup C1 NOp 1
Standard orientation:

Center Number	Atomic Number	Atomic Type	Coordinates (Angstroms) X	Y	Z
1	8	0	2.735801	-0.325766	0.000189
2	1	0	1.777385	-0.167268	-0.000788
3	1	0	2.854479	-1.282620	-0.000811
4	8	0	0.000035	0.694272	-0.000145
5	1	0	0.000379	1.277840	0.771765
6	1	0	-0.000039	1.279670	-0.770667
7	8	0	-2.735859	-0.325731	0.000100
8	1	0	-2.854569	-1.282580	-0.000258
9	1	0	-1.777449	-0.167245	-0.000393

Rotational constants (GHZ): 26.6419672 1.9184767 1.8049327

OH^-

Electronic Energy -228.874555
Stoichiometry H5O3(1-)
Framework group C1[X(H5O3)]
Deg. of freedom 18
Full point group C1 NOp 1
Largest Abelian subgroup C1 NOp 1
Largest concise Abelian subgroup C1 NOp 1
Standard orientation:

Center Number	Atomic Number	Atomic Type	Coordinates (Angstroms) X	Y	Z
1	8	0	0.002573	0.774056	0.059619
2	1	0	-0.001673	1.586363	-0.461383
3	8	0	-2.299048	-0.359957	0.050835
4	1	0	-2.085911	-1.166115	-0.433319
5	1	0	-1.385258	0.138503	0.080533
6	8	0	2.250581	-0.436666	-0.101112
7	1	0	1.327618	0.052367	-0.087303
8	1	0	2.512382	-0.430583	0.826732

Rotational constants (GHZ): 25.0953866 2.7863934 2.5409504

Phenol

Electronic Energy -460.411219
Stoichiometry C6H10O3
Framework group C1[X(C6H10O3)]
Deg. of freedom 51
Full point group C1 NOp 1
Largest Abelian subgroup C1 NOp 1
Largest concise Abelian subgroup C1 NOp 1
Standard orientation:

Center Number	Atomic Number	Atomic Type	Coordinates (Angstroms) X	Y	Z

1	6	0	2.964200	-0.390659	0.530711
2	6	0	1.810158	-1.172913	0.449199
3	6	0	0.627468	-0.619687	-0.061591
4	6	0	0.606148	0.715701	-0.489894
5	6	0	1.769039	1.487575	-0.402824
6	6	0	2.953086	0.943639	0.106250
7	1	0	3.875890	-0.828007	0.928313
8	1	0	1.813119	-2.208476	0.776642
9	1	0	-0.310113	1.142478	-0.886028
10	1	0	1.744116	2.521233	-0.736822
11	1	0	3.852461	1.548424	0.170832
12	8	0	-0.480716	-1.430466	-0.122085
13	1	0	-1.269853	-0.952600	-0.490963
14	8	0	-2.742693	-0.299175	-1.099414
15	1	0	-3.283657	-1.007738	-1.477859
16	1	0	-3.278052	0.072541	-0.358634
17	8	0	-4.129475	0.838829	1.015128
18	1	0	-3.678972	0.584287	1.834252
19	1	0	-5.022454	0.472408	1.100127

Rotational constants (GHZ): 2.7044459 0.5956508 0.5483634

Phenolate
Electronic Energy -459.942619
Stoichiometry C6H9O3(1-)
Framework group C1[X(C6H9O3)]
Deg. of freedom 48
Full point group C1 NOp 1
Largest Abelian subgroup C1 NOp 1
Largest concise Abelian subgroup C1 NOp 1
Standard orientation:

Center Number	Atomic Number	Atomic Type	Coordinates (Angstroms) X	Y	Z
1	6	0	-2.253191	-1.199371	-0.094639
2	6	0	-0.859724	-1.208477	-0.096867
3	6	0	-0.101532	-0.000162	-0.001151
4	6	0	-0.859465	1.208197	0.095724
5	6	0	-2.252956	1.199182	0.095533
6	6	0	-2.975542	-0.000052	0.000962
7	1	0	-2.788653	-2.145917	-0.169196
8	1	0	-0.312317	-2.145183	-0.170476
9	1	0	-0.311939	2.144890	0.168570
10	1	0	-2.788203	2.145786	0.170885
11	1	0	-4.062832	-0.000018	0.001751
12	8	0	1.201996	-0.000265	-0.002171
13	8	0	2.836666	2.116821	-0.037236
14	1	0	3.365086	1.868616	-0.804494
15	1	0	2.165962	1.375378	0.030204
16	8	0	2.838341	-2.116195	0.039205
17	1	0	2.167163	-1.375504	-0.030878
18	1	0	3.364165	-1.866836	0.807871

Rotational constants (GHZ): 2.0697234 0.8518192 0.6062176

Thymol
Electronic Energy -617.680335
Stoichiometry C10H18O3
Framework group C1[X(C10H18O3)]
Deg. of freedom 87
Full point group C1 NOp 1
Largest Abelian subgroup C1 NOp 1
Largest concise Abelian subgroup C1 NOp 1
Standard orientation:

Center Number	Atomic Number	Atomic Type	Coordinates (Angstroms) X	Y	Z
1	6	0	-1.049580	-2.013902	-0.196597
2	1	0	-0.049476	-2.347969	-0.488169

3	6	0	-1.000795	-0.495535	-0.074709
4	6	0	-2.141864	0.254165	0.240651
5	6	0	0.190272	0.223007	-0.282770
6	6	0	-2.116637	1.645953	0.342342
7	1	0	-3.082955	-0.264964	0.403831
8	6	0	0.226551	1.619244	-0.181821
9	6	0	-0.924071	2.350740	0.131824
10	1	0	-3.028998	2.185677	0.582512
11	1	0	1.170341	2.134093	-0.348823
12	6	0	-2.022617	-2.470210	-1.301688
13	1	0	-1.993764	-3.560416	-1.412559
14	1	0	-3.055808	-2.187129	-1.069608
15	1	0	-1.761799	-2.022502	-2.266078
16	6	0	-1.391430	-2.684026	1.149436
17	1	0	-1.364178	-3.775889	1.055269
18	1	0	-0.681903	-2.391797	1.931179
19	1	0	-2.394415	-2.403717	1.491513
20	6	0	-0.866860	3.855110	0.269617
21	1	0	-0.585591	4.148322	1.289174
22	1	0	-0.128806	4.292581	-0.410209
23	1	0	-1.838279	4.311096	0.054814
24	8	0	1.332622	-0.482824	-0.590218
25	1	0	2.076442	0.124093	-0.800727
26	8	0	3.474478	-1.466621	1.000484
27	1	0	2.555491	-1.410398	0.675304
28	1	0	3.427811	-1.534078	1.961555
29	8	0	3.798169	0.733876	-0.644227
30	1	0	4.446545	0.686001	-1.356779
31	1	0	4.029384	0.034266	0.001773

Rotational constants (GHZ): 0.6449740 0.5728722 0.3422173

Thymolate

Electronic Energy -617.210138
Stoichiometry C10H17O3(1-)
Framework group C1[X(C10H17O3)]
Deg. of freedom 84
Full point group C1 NOp 1
Largest Abelian subgroup C1 NOp 1
Largest concise Abelian subgroup C1 NOp 1
Standard orientation:

Center Number	Atomic Number	Atomic Type	Coordinates (Angstroms) X	Y	Z
1	6	0	0.844972	1.937787	-0.155380
2	1	0	1.614279	1.423451	-0.745946
3	6	0	-0.346040	0.993276	-0.097665
4	6	0	-1.669608	1.431961	-0.120803
5	6	0	-0.076939	-0.419556	0.015968
6	6	0	-2.760463	0.551747	-0.039666
7	1	0	-1.875039	2.497877	-0.204106
8	6	0	-1.206026	-1.288345	0.092060
9	6	0	-2.523682	-0.826698	0.064877
10	1	0	-3.778142	0.937276	-0.059433
11	1	0	-1.004483	-2.354346	0.186416
12	6	0	1.446015	2.160242	1.249716
13	1	0	2.363880	2.762147	1.197143
14	1	0	0.728837	2.683784	1.896122
15	1	0	1.693310	1.200785	1.711680
16	6	0	0.557118	3.286053	-0.834542
17	1	0	1.484369	3.864064	-0.937450
18	1	0	0.128096	3.148903	-1.834054
19	1	0	-0.142945	3.898405	-0.251159
20	6	0	-3.678700	-1.807081	0.114443
21	1	0	-3.904500	-2.215267	-0.880920
22	1	0	-3.453562	-2.658219	0.767819
23	1	0	-4.593055	-1.328556	0.485332
24	8	0	1.141105	-0.877420	0.049496
25	8	0	2.038575	-3.343561	-0.155690
26	1	0	1.870612	-3.558995	-1.080473
27	1	0	1.587721	-2.449558	-0.025660

28	8	0	3.940915	-0.959349	-0.119130
29	1	0	3.026578	-0.611135	-0.065156
30	1	0	3.769397	-1.914290	-0.091614

Rotational constants (GHZ): 0.7247502 0.5537282 0.3322523

Carvacrol
Electronic Energy -617.820125
Stoichiometry C10H18O3
Framework group C1[X(C10H18O3)]
Deg. of freedom 87
Full point group C1 NOp 1
Largest Abelian subgroup C1 NOp 1
Largest concise Abelian subgroup C1 NOp 1
Standard orientation:

Center Number	Atomic Number	Atomic Type	Coordinates (Angstroms) X	Y	Z
1	6	0	-0.601130	1.503064	0.108323
2	6	0	-0.651752	0.153483	-0.269028
3	6	0	0.506202	-0.622778	-0.347645
4	6	0	1.760049	-0.084651	-0.052791
5	6	0	1.823654	1.263167	0.325979
6	6	0	0.663067	2.029581	0.400696
7	1	0	2.778417	1.719425	0.562674
8	8	0	-1.834470	-0.482475	-0.590818
9	1	0	-2.620200	0.084332	-0.477170
10	8	0	-3.542546	-2.469731	0.633214
11	1	0	-3.658614	-3.268532	0.103847
12	1	0	-2.747914	-2.041902	0.270599
13	8	0	-4.620369	0.043331	-0.210467
14	1	0	-4.498961	-0.892749	0.037880
15	1	0	-5.140192	0.023045	-1.023377
16	1	0	0.730730	3.072510	0.694419
17	1	0	0.413074	-1.662302	-0.645421
18	6	0	-1.844813	2.350133	0.196711
19	1	0	-2.383586	2.381893	-0.756518
20	1	0	-2.543064	1.968359	0.949064
21	1	0	-1.587716	3.374859	0.469510
22	6	0	3.008714	-0.949834	-0.145701
23	1	0	2.682752	-1.947120	-0.459368
24	6	0	3.989973	-0.424499	-1.207600
25	1	0	4.848372	-1.097093	-1.299515
26	1	0	3.511661	-0.350296	-2.188479
27	1	0	4.368636	0.566744	-0.940186
28	6	0	3.706479	-1.094503	1.217612
29	1	0	3.025635	-1.500702	1.971186
30	1	0	4.564071	-1.769618	1.137443
31	1	0	4.073325	-0.128822	1.578642

Rotational constants (GHZ): 1.1679865 0.3419093 0.2867066

Carvacrolate
Electronic Energy -617.353212
Stoichiometry C10H17O3(1-)
Framework group C1[X(C10H17O3)]
Deg. of freedom 84
Full point group C1 NOp 1
Largest Abelian subgroup C1 NOp 1
Largest concise Abelian subgroup C1 NOp 1
Standard orientation:

Center Number	Atomic Number	Atomic Type	Coordinates (Angstroms) X	Y	Z
1	6	0	0.857887	-1.552212	-0.234259
2	6	0	0.918941	-0.131993	-0.328789
3	6	0	-0.293404	0.590540	-0.206422
4	6	0	-1.528196	-0.027148	0.004813
5	6	0	-1.565909	-1.425337	0.098242
6	6	0	-0.382498	-2.158209	-0.023499

7	1	0	-2.504954	-1.943313	0.260603
8	8	0	2.071613	0.494594	-0.533614
9	8	0	4.142312	0.317477	1.150307
10	1	0	4.401350	-0.608645	1.220458
11	1	0	3.360817	0.314724	0.532555
12	8	0	2.276015	3.153086	-0.470280
13	1	0	2.161354	2.159527	-0.484301
14	1	0	3.166560	3.294618	-0.128942
15	1	0	-0.246311	1.673647	-0.282029
16	1	0	-0.423545	-3.242478	0.045066
17	6	0	2.113921	-2.374989	-0.369963
18	1	0	2.643486	-2.153327	-1.303077
19	1	0	2.822315	-2.179378	0.443112
20	1	0	1.881120	-3.442578	-0.358806
21	6	0	-2.793845	0.813502	0.124075
22	1	0	-2.488842	1.862010	0.037020
23	6	0	-3.476033	0.641247	1.492128
24	1	0	-2.789726	0.877030	2.310760
25	1	0	-4.342090	1.305636	1.575656
26	1	0	-3.828463	-0.385225	1.632680
27	6	0	-3.785692	0.523428	-1.015977
28	1	0	-4.655447	1.184673	-0.946621
29	1	0	-3.321712	0.677758	-1.994580
30	1	0	-4.146476	-0.508916	-0.972951

Rotational constants (GHZ): 0.9754935 0.4102434 0.3233110

2,3-dimethylphenol
Electronic Energy -539.171228
Stoichiometry C8H14O3
Framework group C1[X(C8H14O3)]
Deg. of freedom 69
Full point group C1 NOp 1
Largest Abelian subgroup C1 NOp 1
Largest concise Abelian subgroup C1 NOp 1
Standard orientation:

Center Number	Atomic Number	Atomic Type	Coordinates (Angstroms) X	Y	Z
1	6	0	0.836349	0.574545	-0.111473
2	6	0	-0.006242	-0.534418	-0.296627
3	6	0	0.480558	-1.841620	-0.269960
4	6	0	1.834095	-2.059548	-0.042514
5	6	0	2.688625	-0.977377	0.160123
6	6	0	2.206243	0.336755	0.130029
7	1	0	2.220814	-3.072324	-0.018307
8	1	0	3.743428	-1.148904	0.345108
9	8	0	-1.359923	-0.396979	-0.531087
10	1	0	-1.686725	0.512505	-0.397545
11	8	0	-3.872111	-1.132349	0.681172
12	1	0	-4.454342	-1.635904	0.098589
13	1	0	-2.987757	-1.212765	0.284162
14	8	0	-3.328320	1.594875	0.018982
15	1	0	-3.750164	0.743281	0.243233
16	1	0	-3.800876	1.910914	-0.761050
17	1	0	-0.207204	-2.665434	-0.422810
18	6	0	0.262836	1.970019	-0.167169
19	1	0	-0.406612	2.092834	-1.024139
20	1	0	-0.317074	2.203038	0.733065
21	1	0	1.044079	2.722802	-0.256263
22	6	0	3.167790	1.479630	0.358778
23	1	0	3.223848	2.142819	-0.510276
24	1	0	2.868135	2.095769	1.212059
25	1	0	4.171755	1.099078	0.554513

Rotational constants (GHZ): 1.5642966 0.5594339 0.4268884

2,3-dimethylphenolate
Electronic Energy -538.704374
Stoichiometry C8H13O3(1-)
Framework group C1[X(C8H13O3)]

Deg. of freedom 66
Full point group C1 NOp 1
Largest Abelian subgroup C1 NOp 1
Largest concise Abelian subgroup C1 NOp 1
Standard orientation:

Center Number	Atomic Number	Atomic Type	Coordinates (Angstroms) X	Y	Z
1	6	0	0.122245	-1.637449	0.396652
2	6	0	0.355411	-0.306969	-0.027855
3	6	0	-0.774905	0.532203	-0.265716
4	6	0	-2.075342	0.039108	-0.049854
5	6	0	-2.267369	-1.283100	0.377935
6	6	0	-1.168303	-2.114811	0.594112
7	1	0	0.979412	-2.279262	0.574202
8	1	0	-3.274581	-1.652405	0.540396
9	8	0	1.595606	0.138395	-0.197997
10	8	0	3.605322	-1.492182	-0.783774
11	1	0	3.689031	-1.456519	-1.743451
12	1	0	2.818726	-0.911390	-0.568538
13	8	0	2.541764	2.237813	1.143459
14	1	0	2.159235	1.458247	0.652231
15	1	0	3.140010	1.851932	1.793457
16	1	0	-1.319315	-3.138353	0.924076
17	6	0	-0.551973	1.949688	-0.736965
18	1	0	-0.522733	2.660089	0.099137
19	1	0	0.398843	2.039610	-1.264564
20	1	0	-1.345724	2.282651	-1.409406
21	6	0	-3.283054	0.922214	-0.271433
22	1	0	-3.235594	1.834000	0.332918
23	1	0	-3.367625	1.241133	-1.315906
24	1	0	-4.201482	0.392755	-0.009309

Rotational constants (GHZ): 1.2911874 0.6504205 0.4833657

2,4-dimethylphenol
Electronic Energy -539.172676
Stoichiometry C8H14O3
Framework group C1[X(C8H14O3)]
Deg. of freedom 69
Full point group C1 NOp 1
Largest Abelian subgroup C1 NOp 1
Largest concise Abelian subgroup C1 NOp 1
Standard orientation:

Center Number	Atomic Number	Atomic Type	Coordinates (Angstroms) X	Y	Z
1	6	0	0.541981	0.968894	-0.071812
2	6	0	-0.075518	-0.264959	-0.331351
3	6	0	0.673489	-1.438978	-0.381888
4	6	0	2.049162	-1.402610	-0.166517
5	6	0	2.700835	-0.195751	0.106548
6	6	0	1.924943	0.968273	0.143272
7	1	0	2.618370	-2.325447	-0.211370
8	8	0	-1.431176	-0.381232	-0.573873
9	1	0	-1.929631	0.431882	-0.367950
10	8	0	-3.693600	-1.672957	0.640052
11	1	0	-4.176863	-2.261205	0.046526
12	1	0	-2.828271	-1.540884	0.214811
13	8	0	-3.733284	1.148433	0.146235
14	1	0	-3.977515	0.220625	0.326400
15	1	0	-4.272664	1.406615	-0.611399
16	1	0	2.410352	1.919509	0.342458
17	1	0	0.169766	-2.375828	-0.593248
18	6	0	-0.249683	2.251346	-0.034231
19	1	0	-0.788060	2.420640	-0.972916
20	1	0	-0.995510	2.242795	0.767568
21	1	0	0.411268	3.102933	0.133266
22	6	0	4.184945	-0.149748	0.378911
23	1	0	4.627859	0.781820	0.017691

24	1	0	4.392535	-0.212622	1.453208
25	1	0	4.701916	-0.983603	-0.101945

Rotational constants (GHZ): 1.8646861 0.4857021 0.3990726

2,4-dimethylphenolate
Electronic Energy -538.705545
Stoichiometry C8H13O3(1-)
Framework group C1[X(C8H13O3)]
Deg. of freedom 66
Full point group C1 NOp 1
Largest Abelian subgroup C1 NOp 1
Largest concise Abelian subgroup C1 NOp 1
Standard orientation:

Center Number	Atomic Number	Atomic Type	Coordinates (Angstroms) X	Y	Z
1	6	0	-0.215692	-1.309324	0.157490
2	6	0	0.390348	-0.089208	-0.212647
3	6	0	-0.466031	1.037067	-0.393876
4	6	0	-1.840595	0.894209	-0.201152
5	6	0	-2.440218	-0.320313	0.167761
6	6	0	-1.595508	-1.419218	0.341399
7	1	0	0.418799	-2.179269	0.300033
8	1	0	-2.473095	1.767480	-0.346770
9	8	0	1.704770	0.014421	-0.390867
10	8	0	3.201920	-2.157093	-0.669584
11	1	0	2.759617	-2.888687	-0.223930
12	1	0	2.593010	-1.371351	-0.539304
13	8	0	3.152524	1.418009	1.367529
14	1	0	2.596973	0.900873	0.721637
15	1	0	3.639855	0.751744	1.865568
16	1	0	-2.017583	-2.379211	0.626566
17	6	0	0.125671	2.362776	-0.798483
18	1	0	0.835865	2.735208	-0.052433
19	1	0	0.678819	2.285335	-1.741139
20	1	0	-0.655482	3.116015	-0.925295
21	6	0	-3.934247	-0.422055	0.370351
22	1	0	-4.278047	0.223973	1.185882
23	1	0	-4.486695	-0.122479	-0.526816
24	1	0	-4.228126	-1.445939	0.614323

Rotational constants (GHZ): 1.4073639 0.5803614 0.4590803

2,6-diisopropylphenol
Electronic Energy -696.094271
Stoichiometry C12H22O3
Framework group C1[X(C12H22O3)]
Deg. of freedom 105
Full point group C1 NOp 1
Largest Abelian subgroup C1 NOp 1
Largest concise Abelian subgroup C1 NOp 1
Standard orientation:

Center Number	Atomic Number	Atomic Type	Coordinates (Angstroms) X	Y	Z
1	6	0	1.117396	-1.021888	-0.079110
2	6	0	-0.059336	-0.266160	-0.133653
3	6	0	-1.329336	-0.855185	-0.117149
4	6	0	-1.401326	-2.240179	-0.035297
5	6	0	-0.253509	-3.015761	0.023384
6	6	0	0.989566	-2.405927	-0.001396
7	1	0	-2.369126	-2.724927	-0.026571
8	1	0	-0.328227	-4.094759	0.082251
9	8	0	-0.028055	1.102016	-0.199083
10	1	0	0.856668	1.476925	-0.359194
11	8	0	-0.845787	3.742564	-0.106490
12	1	0	-1.323768	4.110059	0.640522
13	1	0	-0.937047	2.776469	-0.053148
14	8	0	1.790018	3.110565	-0.526348

15	1	0	0.979678	3.636725	-0.389644
16	1	0	2.242963	3.489411	-1.283117
17	1	0	1.879523	-3.020594	0.037492
18	6	0	-2.562322	0.028687	-0.162134
19	1	0	-2.332222	0.856323	-0.839066
20	6	0	-2.850961	0.624183	1.223221
21	1	0	-1.977828	1.140753	1.625810
22	1	0	-3.682729	1.332694	1.176710
23	1	0	-3.121409	-0.165945	1.928736
24	6	0	-3.805197	-0.671816	-0.708622
25	1	0	-4.617238	0.049475	-0.825344
26	1	0	-3.615120	-1.126653	-1.682882
27	1	0	-4.162477	-1.452961	-0.032885
28	6	0	2.473554	-0.336215	-0.057428
29	1	0	2.429318	0.529117	-0.727970
30	6	0	2.794371	0.187419	1.349601
31	1	0	3.736090	0.741235	1.350524
32	1	0	2.015256	0.854872	1.720588
33	1	0	2.885139	-0.647045	2.049844
34	6	0	3.614746	-1.212547	-0.573334
35	1	0	3.393276	-1.624325	-1.560020
36	1	0	4.529934	-0.621354	-0.649150
37	1	0	3.824061	-2.044314	0.103391

Rotational constants (GHZ): 0.5782226 0.4700602 0.2833303

2,6-diisopropylphenolate
Electronic Energy -695.625109
Stoichiometry C12H21O3(1-)
Framework group C1[X(C12H21O3)]
Deg. of freedom 102
Full point group C1 NOp 1
Largest Abelian subgroup C1 NOp 1
Largest concise Abelian subgroup C1 NOp 1
Standard orientation:

Center Number	Atomic Number	Atomic Type	Coordinates (Angstroms) X	Y	Z
1	6	0	1.358986	-0.756285	-0.046465
2	6	0	0.069902	-0.140274	-0.060458
3	6	0	-1.075140	-0.989154	-0.116270
4	6	0	-0.913596	-2.367445	-0.131883
5	6	0	0.343743	-2.959020	-0.105504
6	6	0	1.466768	-2.138370	-0.066545
7	1	0	-1.791396	-3.005336	-0.173193
8	1	0	0.448086	-4.038291	-0.124417
9	8	0	-0.037165	1.153091	-0.028665
10	8	0	0.475970	3.387682	-1.575367
11	1	0	-0.276447	3.771058	-1.108414
12	1	0	0.496613	2.494700	-1.174380
13	8	0	-1.556722	3.193460	0.641465
14	1	0	-1.095562	2.337698	0.420597
15	1	0	-1.091015	3.512232	1.417104
16	1	0	2.450004	-2.597917	-0.057373
17	6	0	-2.442748	-0.336507	-0.121060
18	1	0	-2.314434	0.637401	-0.599861
19	6	0	-3.500267	-1.109528	-0.910583
20	1	0	-3.747593	-2.064541	-0.436667
21	1	0	-4.425839	-0.528872	-0.971796
22	1	0	-3.160930	-1.318845	-1.927934
23	6	0	-2.930358	-0.075407	1.309879
24	1	0	-2.195516	0.509126	1.864194
25	1	0	-3.872849	0.481806	1.311803
26	1	0	-3.086065	-1.022011	1.838593
27	6	0	2.564827	0.159850	0.019909
28	1	0	2.342548	1.010051	-0.631513
29	6	0	3.867579	-0.475265	-0.463948
30	1	0	4.664041	0.274433	-0.491037
31	1	0	4.200172	-1.278790	0.200896
32	1	0	3.760883	-0.893502	-1.468011
33	6	0	2.739383	0.727475	1.434553

34	1	0	3.553397	1.460086	1.467975
35	1	0	1.819589	1.219667	1.750628
36	1	0	2.971170	-0.074433	2.143591

Rotational constants (GHZ): 0.5861723 0.4602902 0.2994736

2-methyl-4-*terz*-butylphenol
Electronic Energy -657.139992
Stoichiometry C11H20O3
Framework group C1[X(C11H20O3)]
Deg. of freedom 96
Full point group C1 NOp 1
Largest Abelian subgroup C1 NOp 1
Largest concise Abelian subgroup C1 NOp 1
Standard orientation:

Center Number	Atomic Number	Atomic Type	Coordinates (Angstroms) X	Y	Z
1	6	0	1.011335	-1.329020	-0.310999
2	6	0	-0.366608	-1.397576	-0.479987
3	6	0	-1.144737	-0.243495	-0.390352
4	6	0	-0.546974	0.996550	-0.135486
5	6	0	0.845407	1.028335	0.033314
6	6	0	1.654779	-0.109960	-0.045784
7	1	0	1.586300	-2.244882	-0.386035
8	1	0	-0.852801	-2.345932	-0.681532
9	1	0	1.291304	1.995428	0.230817
10	8	0	-2.504603	-0.393274	-0.583932
11	1	0	-3.014594	0.408570	-0.362385
12	8	0	-4.814791	1.091376	0.201447
13	1	0	-5.381258	1.332259	-0.541906
14	1	0	-5.034126	0.160216	0.395731
15	8	0	-4.697816	-1.722300	0.725095
16	1	0	-3.847238	-1.577001	0.275377
17	1	0	-5.182283	-2.329829	0.152318
18	6	0	3.182241	-0.067727	0.140933
19	6	0	3.697558	1.351518	0.435269
20	1	0	3.266243	1.759773	1.353769
21	1	0	4.783489	1.323933	0.562445
22	1	0	3.477102	2.043565	-0.382476
23	6	0	3.583199	-0.980156	1.322115
24	1	0	3.286058	-2.017823	1.150719
25	1	0	4.668752	-0.961426	1.462051
26	1	0	3.116047	-0.643065	2.252512
27	6	0	3.873585	-0.574682	-1.144949
28	1	0	3.582187	-1.600909	-1.382653
29	1	0	3.618318	0.057169	-2.001171
30	1	0	4.961187	-0.555182	-1.021747
31	6	0	-1.364653	2.260714	-0.047861
32	1	0	-1.952288	2.425302	-0.957424
33	1	0	-2.069792	2.230161	0.789565
34	1	0	-0.715726	3.126249	0.093861

Rotational constants (GHZ): 1.3255978 0.2688096 0.2406409

2-methyl-4-*terz*-butylphenolate
Electronic Energy -656.672780
Stoichiometry C11H19O3(1-)
Framework group C1[X(C11H19O3)]
Deg. of freedom 93
Full point group C1 NOp 1
Largest Abelian subgroup C1 NOp 1
Largest concise Abelian subgroup C1 NOp 1
Standard orientation:

Center Number	Atomic Number	Atomic Type	Coordinates (Angstroms) X	Y	Z
1	6	0	0.672512	-1.295363	0.274298
2	6	0	-0.720058	-1.258871	0.167192
3	6	0	-1.410736	-0.088208	-0.206731

4	6	0	-0.623595	1.069695	-0.474924
5	6	0	0.764412	1.000927	-0.356467
6	6	0	1.456519	-0.166128	0.016227
7	1	0	1.136128	-2.230100	0.566985
8	1	0	-1.297251	-2.154407	0.377684
9	1	0	1.328440	1.904942	-0.568728
10	8	0	-2.737289	-0.056197	-0.309305
11	8	0	-4.131366	1.290908	1.534332
12	1	0	-4.400333	0.623621	2.176063
13	1	0	-3.592155	0.795032	0.858552
14	8	0	-4.173015	-2.271645	-0.575048
15	1	0	-3.596170	-1.457565	-0.482742
16	1	0	-4.495340	-2.249429	-1.483295
17	6	0	2.992122	-0.155504	0.124816
18	6	0	3.433980	0.874434	1.189513
19	1	0	4.525938	0.894993	1.271384
20	1	0	3.098540	1.883668	0.936907
21	1	0	3.026004	0.619166	2.172504
22	6	0	3.556255	-1.529339	0.527411
23	1	0	3.304498	-2.303096	-0.203689
24	1	0	4.647113	-1.472924	0.588455
25	1	0	3.185865	-1.851918	1.504745
26	6	0	3.612537	0.235579	-1.235914
27	1	0	4.705372	0.252070	-1.166325
28	1	0	3.334211	-0.483143	-2.012996
29	1	0	3.283802	1.226423	-1.559841
30	6	0	-1.301661	2.351334	-0.888683
31	1	0	-1.895719	2.216707	-1.799543
32	1	0	-1.992274	2.712415	-0.119000
33	1	0	-0.567033	3.137696	-1.077383

Rotational constants (GHZ): 1.0743958 0.3051678 0.2706838

4-bromothymol
Electronic Energy -3191.363864
Stoichiometry C10H17BrO3
Framework group C1[X(C10H17BrO3)]
Deg. of freedom 87
Full point group C1 NOp 1
Largest Abelian subgroup C1 NOp 1
Largest concise Abelian subgroup C1 NOp 1
Standard orientation:

Center Number	Atomic Number	Atomic Type	Coordinates (Angstroms) X	Y	Z
1	6	0	-0.479437	-1.410239	-0.376846
2	6	0	-1.205760	-0.220334	-0.322939
3	6	0	-0.564934	1.011386	-0.098614
4	6	0	0.822263	0.984554	0.059878
5	6	0	1.534470	-0.209872	-0.000227
6	6	0	0.910843	-1.440918	-0.218799
7	1	0	-1.008628	-2.341748	-0.548827
8	1	0	1.355428	1.909527	0.235026
9	8	0	-2.567552	-0.218189	-0.488983
10	1	0	-2.915518	-1.131274	-0.599066
11	6	0	-1.363876	2.307183	-0.074760
12	1	0	-2.342293	2.067251	0.352337
13	6	0	-1.600703	2.823854	-1.507828
14	1	0	-2.085912	2.069627	-2.132352
15	1	0	-2.238733	3.712889	-1.493348
16	1	0	-0.651106	3.095828	-1.979886
17	6	0	-0.739134	3.404365	0.796301
18	1	0	-1.420420	4.257718	0.856964
19	1	0	-0.548774	3.052400	1.814204
20	1	0	0.204368	3.769896	0.380366
21	6	0	1.651456	-2.749364	-0.286971
22	1	0	2.185515	-2.955081	0.645304
23	1	0	0.957254	-3.570626	-0.470201
24	1	0	2.395681	-2.745223	-1.088681
25	8	0	-4.778145	-0.691494	1.540629
26	1	0	-4.003610	-0.226252	1.187558

27	1	0	-4.550969	-0.891751	2.457549
28	8	0	-4.008515	-2.584261	-0.418313
29	1	0	-4.653257	-2.676990	-1.131110
30	1	0	-4.479955	-2.113957	0.297254
31	35	0	3.452671	-0.123839	0.238145

Rotational constants (GHZ): 0.6155415 0.2459348 0.1910200

4-bromothymolate
Electronic Energy -3190.896942
Stoichiometry C10H16BrO3(1-)
Framework group C1[X(C10H16BrO3)]
Deg. of freedom 84
Full point group C1 NOp 1
Largest Abelian subgroup C1 NOp 1
Largest concise Abelian subgroup C1 NOp 1
Standard orientation:

Center Number	Atomic Number	Atomic Type	Coordinates (Angstroms) X	Y	Z
1	6	0	1.666010	2.096049	-0.169124
2	1	0	2.596673	1.837263	0.345167
3	6	0	0.764060	0.873029	-0.074813
4	6	0	-0.625039	0.988365	-0.026105
5	6	0	1.342397	-0.433193	-0.078120
6	6	0	-1.448922	-0.134707	0.021554
7	1	0	-1.079262	1.971434	-0.025629
8	6	0	0.465694	-1.541592	-0.020175
9	6	0	-0.927981	-1.431297	0.027260
10	1	0	0.905905	-2.534524	-0.014059
11	6	0	1.098268	3.350164	0.508644
12	1	0	1.845206	4.149614	0.497470
13	1	0	0.210235	3.728970	-0.006676
14	1	0	0.827807	3.157789	1.551117
15	6	0	2.027893	2.397043	-1.637278
16	1	0	2.743283	3.223824	-1.698942
17	1	0	2.475259	1.527251	-2.125346
18	1	0	1.134372	2.680930	-2.203433
19	6	0	-1.786036	-2.668791	0.083343
20	1	0	-2.467626	-2.723936	-0.770926
21	1	0	-1.163199	-3.564962	0.078913
22	1	0	-2.404702	-2.688644	0.985676
23	8	0	2.653753	-0.612049	-0.131886
24	8	0	3.648804	-2.836545	-1.203505
25	1	0	3.052478	-3.561530	-0.983837
26	1	0	3.231590	-2.029013	-0.787015
27	8	0	4.054694	-0.289343	2.135449
28	1	0	3.518949	-0.411076	1.306531
29	1	0	4.110035	-1.168808	2.526592
30	35	0	-3.367231	0.152528	0.088937

Rotational constants (GHZ): 0.6043836 0.2616458

4-chlorothymol
Electronic Energy -1077.443594
Stoichiometry C10H17ClO3
Framework group C1[X(C10H17ClO3)]
Deg. of freedom 87
Full point group C1 NOp 1
Largest Abelian subgroup C1 NOp 1
Largest concise Abelian subgroup C1 NOp 1
Standard orientation:

Center Number	Atomic Number	Atomic Type	Coordinates (Angstroms) X	Y	Z
1	6	0	0.050211	-1.435408	-0.316608
2	6	0	-0.648695	-0.227972	-0.290553
3	6	0	0.014490	0.990607	-0.059005
4	6	0	1.396132	0.933699	0.134295
5	6	0	2.079534	-0.277415	0.101055

6	6	0	1.434180	-1.495443	-0.122834
7	1	0	-0.495914	-2.355798	-0.494770
8	1	0	1.951685	1.844462	0.315898
9	8	0	-2.005736	-0.195831	-0.492281
10	1	0	-2.370077	-1.101577	-0.607859
11	6	0	-0.754832	2.304208	-0.064193
12	1	0	-1.750785	2.089041	0.334444
13	6	0	-0.936917	2.819518	-1.505734
14	1	0	-1.421070	2.073793	-2.141224
15	1	0	-1.553979	3.723308	-1.513739
16	1	0	0.032342	3.066915	-1.950539
17	6	0	-0.130691	3.390556	0.820844
18	1	0	-0.794046	4.259207	0.859221
19	1	0	0.022532	3.038260	1.844866
20	1	0	0.832228	3.733302	0.430895
21	6	0	2.157400	-2.814579	-0.158888
22	1	0	2.672736	-3.012488	0.785643
23	1	0	1.455423	-3.629143	-0.342131
24	1	0	2.915290	-2.831779	-0.947747
25	8	0	-4.338118	-0.567866	1.412351
26	1	0	-3.532077	-0.131619	1.094307
27	1	0	-4.164999	-0.761365	2.342479
28	8	0	-3.514799	-2.522188	-0.464323
29	1	0	-4.114017	-2.606984	-1.216603
30	1	0	-4.014749	-2.019231	0.208280
31	17	0	3.837284	-0.248109	0.360138

Rotational constants (GHZ): 0.6230492 0.3454761 0.2

4-chlorothymolate

Electronic Energy -1076.976267

Stoichiometry C10H16ClO3(1-)

Framework group C1[X(C10H16ClO3)]

Deg. of freedom 84

Full point group C1 NOp 1

Largest Abelian subgroup C1 NOp 1

Largest concise Abelian subgroup C1 NOp 1

Standard orientation:

Center Number	Atomic Number	Atomic Type	Coordinates (Angstroms) X	Y	Z
1	6	0	0.209459	-1.552469	-0.027995
2	6	0	-0.754366	-0.523551	-0.166382
3	6	0	-0.284230	0.823807	-0.181329
4	6	0	1.086603	1.057740	-0.061082
5	6	0	1.994357	0.012798	0.070931
6	6	0	1.582179	-1.322923	0.090938
7	1	0	-0.145780	-2.578207	-0.013629
8	1	0	1.457119	2.076870	-0.071725
9	8	0	-2.041522	-0.820293	-0.277854
10	6	0	-1.248003	1.992673	-0.333932
11	1	0	-2.251481	1.566521	-0.396872
12	6	0	-0.997063	2.779007	-1.633090
13	1	0	-1.058150	2.127078	-2.509542
14	1	0	-1.740408	3.574079	-1.751665
15	1	0	-0.006902	3.246085	-1.629765
16	6	0	-1.218513	2.932140	0.884386
17	1	0	-1.963905	3.726614	0.775279
18	1	0	-1.437154	2.389834	1.808770
19	1	0	-0.239059	3.408166	0.996665
20	6	0	2.545495	-2.472720	0.233522
21	1	0	2.008922	-3.422836	0.223649
22	1	0	3.277276	-2.486029	-0.580066
23	1	0	3.109930	-2.410027	1.168998
24	8	0	-2.923820	-3.261849	-0.864129
25	1	0	-2.913456	-3.327536	-1.825815
26	1	0	-2.543057	-2.360185	-0.659864
27	8	0	-3.718775	-0.121284	1.686395
28	1	0	-3.078650	-0.368481	0.964690
29	1	0	-3.847427	-0.932354	2.191361
30	17	0	3.726448	0.414048	0.217554

Rotational constants (GHZ): 0.6121752 0.3739397 0.2644884

4-methoxyphenol
Electronic Energy -575.077262
Stoichiometry C7H12O4
Framework group C1[X(C7H12O4)]
Deg. of freedom 63
Full point group C1 NOp 1
Largest Abelian subgroup C1 NOp 1
Largest concise Abelian subgroup C1 NOp 1
Standard orientation:

Center Number	Atomic Number	Atomic Type	Coordinates (Angstroms) X	Y	Z
1	6	0	1.700563	-0.975283	-0.326105
2	6	0	0.354255	-1.204437	-0.621329
3	6	0	-0.581712	-0.181596	-0.496896
4	6	0	-0.166065	1.085211	-0.074538
5	6	0	1.172795	1.316264	0.220543
6	6	0	2.113304	0.289057	0.097997
7	1	0	2.406229	-1.788098	-0.431993
8	1	0	0.032301	-2.185928	-0.950404
9	1	0	-0.888897	1.887468	0.023178
10	1	0	1.498578	2.296599	0.549119
11	8	0	-1.897946	-0.463281	-0.800496
12	1	0	-2.469593	0.323622	-0.663771
13	8	0	-3.915410	1.354927	-0.160704
14	1	0	-4.548051	1.466346	-0.881471
15	1	0	-4.241380	0.587520	0.349049
16	8	0	-4.076982	-1.141612	1.170384
17	1	0	-3.323009	-1.333969	0.590664
18	1	0	-4.748814	-1.792046	0.929971
19	8	0	3.416225	0.617234	0.416857
20	6	0	4.412747	-0.404901	0.304866
21	1	0	5.350488	0.062412	0.601561
22	1	0	4.195307	-1.243014	0.973294
23	1	0	4.494419	-0.764937	-0.724743

Rotational constants (GHZ): 2.4601649 0.3868185 0.3589148

4-methoxyphenolate
Electronic Energy -574.607639
Stoichiometry C7H11O4(1-)
Framework group C1[X(C7H11O4)]
Deg. of freedom 60
Full point group C1 NOp 1
Largest Abelian subgroup C1 NOp 1
Largest concise Abelian subgroup C1 NOp 1
Standard orientation:

Center Number	Atomic Number	Atomic Type	Coordinates (Angstroms) X	Y	Z
1	6	0	1.312966	-1.207380	-0.019803
2	6	0	-0.073098	-1.199022	0.118225
3	6	0	-0.826955	-0.006480	-0.025175
4	6	0	-0.099046	1.174235	-0.317702
5	6	0	1.288139	1.162144	-0.450432
6	6	0	1.999172	-0.027302	-0.303509
7	1	0	1.866519	-2.134650	0.089295
8	1	0	-0.597663	-2.123368	0.337270
9	1	0	-0.643173	2.104756	-0.442143
10	1	0	1.823639	2.078737	-0.676750
11	8	0	-2.145746	0.001758	0.105874
12	8	0	-3.604029	2.225582	0.219458
13	1	0	-3.837337	2.333808	1.148493
14	1	0	-3.018136	1.416480	0.190338
15	8	0	-3.626233	-2.209072	-0.005192
16	1	0	-3.040296	-1.400214	0.019455
17	1	0	-4.140562	-2.122044	-0.815800

18	8	0	3.388171	-0.041235	-0.488358
19	6	0	4.141225	0.063765	0.731508
20	1	0	5.195648	0.039400	0.454242
21	1	0	3.919115	1.005869	1.243248
22	1	0	3.920520	-0.774794	1.399425

Rotational constants (GHZ): 1.7985083 0.4523952 0.3735018

4-hydroxybenzonitrile
Electronic Energy -552.797296
Stoichiometry C7H9NO3
Framework group C1[X(C7H9NO3)]
Deg. of freedom 54
Full point group C1 NOp 1
Largest Abelian subgroup C1 NOp 1
Largest concise Abelian subgroup C1 NOp 1
Standard orientation:

Center Number	Atomic Number	Atomic Type	Coordinates (Angstroms) X	Y	Z
1	6	0	-2.135175	1.263629	-0.000970
2	6	0	-0.788467	1.582372	-0.011507
3	6	0	0.176308	0.563909	-0.014877
4	6	0	-0.224290	-0.781104	-0.009000
5	6	0	-1.572613	-1.100957	0.001980
6	6	0	-2.540777	-0.082950	0.006216
7	1	0	-2.879867	2.050240	0.002251
8	1	0	-0.465865	2.616524	-0.016524
9	1	0	0.522349	-1.566009	-0.012045
10	1	0	-1.882595	-2.138723	0.007254
11	8	0	1.480599	0.933452	-0.024851
12	1	0	2.092072	0.149000	-0.016501
13	8	0	3.267177	-1.066093	0.045857
14	1	0	3.198371	-1.659911	-0.712496
15	1	0	4.176822	-0.695412	0.005750
16	8	0	5.834283	-0.012394	-0.007754
17	1	0	6.162463	0.105820	0.893286
18	1	0	5.844376	0.874670	-0.390540
19	6	0	-3.924047	-0.412835	0.018024
20	7	0	-5.052889	-0.681185	0.027765

Rotational constants (GHZ): 3.6246217 0.3198568

4-cyanophenolate
Electronic Energy -552.335086
Stoichiometry C7H8NO3(1-)
Framework group C1[X(C7H8NO3)]
Deg. of freedom 51
Full point group C1 NOp 1
Largest Abelian subgroup C1 NOp 1
Largest concise Abelian subgroup C1 NOp 1
Standard orientation:

Center Number	Atomic Number	Atomic Type	Coordinates (Angstroms) X	Y	Z
1	6	0	-1.453836	1.208022	0.127941
2	6	0	-0.073291	1.208249	0.127448
3	6	0	0.673999	-0.000188	-0.000906
4	6	0	-0.073559	-1.208511	-0.128896
5	6	0	-1.454091	-1.208087	-0.128462
6	6	0	-2.171316	0.000022	-0.000009
7	1	0	-1.996411	2.141520	0.227895
8	1	0	0.468088	2.142446	0.227695
9	1	0	0.467643	-2.142772	-0.229553
10	1	0	-1.996892	-2.141489	-0.228104
11	8	0	1.977375	-0.000326	-0.001170
12	8	0	3.499806	-2.226017	0.031851
13	1	0	3.764372	-2.347256	0.950971
14	1	0	2.901044	-1.434111	0.038929
15	8	0	3.499567	2.226184	-0.029918

16	1	0	2.900731	1.434392	-0.038380
17	1	0	3.762868	2.349818	-0.949095
18	6	0	-3.586878	0.000151	0.000565
19	7	0	-4.750229	0.000110	0.001064

Rotational constants (GHZ): 1.9055870 0.4620672 0.3734441

4-nitrophenol
Electronic Energy -665.095584
Stoichiometry C6H9NO5
Framework group C1[X(C6H9NO5)]
Deg. of freedom 57
Full point group C1 NOp 1
Largest Abelian subgroup C1 NOp 1
Largest concise Abelian subgroup C1 NOp 1
Standard orientation:

Center Number	Atomic Number	Atomic Type	Coordinates (Angstroms) X	Y	Z
1	6	0	-1.663101	1.324062	0.098003
2	6	0	-0.342194	1.690227	-0.063005
3	6	0	0.641928	0.709749	-0.284841
4	6	0	0.279643	-0.648725	-0.343547
5	6	0	-1.042197	-1.018127	-0.181948
6	6	0	-2.009656	-0.031591	0.038155
7	1	0	-2.423415	2.073367	0.268512
8	1	0	-0.048628	2.731729	-0.020472
9	1	0	1.039660	-1.400627	-0.514199
10	1	0	-1.327763	-2.059915	-0.225406
11	8	0	1.912185	1.127376	-0.434384
12	1	0	2.555553	0.377892	-0.580219
13	8	0	3.767455	-0.747001	-0.810983
14	1	0	4.182588	-0.635845	-1.675764
15	1	0	4.490674	-0.625141	-0.156087
16	8	0	5.754779	-0.449589	1.105350
17	1	0	5.435792	0.096542	1.835984
18	1	0	6.526750	0.022299	0.766248
19	7	0	-3.387861	-0.417854	0.205636
20	8	0	-3.684408	-1.618432	0.154829
21	8	0	-4.237850	0.461532	0.395817

Rotational constants (GHZ): 2.2440105 0.2882818 0.2682157

4-nitrophenolate
Electronic Energy -664.638571
Stoichiometry C6H8NO5(1-)
Framework group C1[X(C6H8NO5)]
Deg. of freedom 54
Full point group C1 NOp 1
Largest Abelian subgroup C1 NOp 1
Largest concise Abelian subgroup C1 NOp 1
Standard orientation:

Center Number	Atomic Number	Atomic Type	Coordinates (Angstroms) X	Y	Z
1	6	0	1.050540	1.221205	-0.066392
2	6	0	-0.321353	1.221551	-0.066386
3	6	0	-1.072375	0.000008	-0.000098
4	6	0	-0.321364	-1.221539	0.066237
5	6	0	1.050529	-1.221199	0.066336
6	6	0	1.754877	0.000001	-0.000003
7	1	0	1.598519	2.152480	-0.117712
8	1	0	-0.866880	2.156615	-0.117826
9	1	0	-0.866899	-2.156600	0.117639
10	1	0	1.598499	-2.152478	0.117691
11	8	0	-2.358641	0.000011	-0.000141
12	8	0	-3.913440	-2.245515	-0.009920
13	1	0	-4.449508	-2.171078	-0.807875
14	1	0	-3.319517	-1.456586	-0.027192
15	8	0	-3.913477	2.245507	0.010126

16	1	0	-3.319515	1.456604	0.027222
17	1	0	-4.449423	2.170953	0.808151
18	7	0	3.165496	-0.000003	0.000047
19	8	0	3.784720	-1.085824	0.056967
20	8	0	3.784729	1.085814	-0.056856

Rotational constants (GHZ): 1.6580401 0.3809862 0.3104356

4-nitrothymol
Electronic Energy -822.391872
Stoichiometry C10H17NO5
Framework group C1[X(C10H17NO5)]
Deg. of freedom 93
Full point group C1 NOp 1
Largest Abelian subgroup C1 NOp 1
Largest concise Abelian subgroup C1 NOp 1
Standard orientation:

Center Number	Atomic Number	Atomic Type	Coordinates (Angstroms) X	Y	Z
1	6	0	-1.522861	0.767993	-0.007563
2	6	0	-0.218548	1.174921	-0.212015
3	6	0	0.748414	0.153654	-0.382125
4	6	0	0.381975	-1.197778	-0.342943
5	6	0	-0.925726	-1.617280	-0.119388
6	6	0	-1.881548	-0.589233	0.049543
7	1	0	-2.301820	1.503646	0.128831
8	1	0	1.151052	-1.945291	-0.496678
9	8	0	2.027557	0.524705	-0.588677
10	1	0	2.642373	-0.252129	-0.693137
11	8	0	3.851884	-1.412632	-0.883421
12	1	0	4.286402	-1.305392	-1.739086
13	1	0	4.560815	-1.288119	-0.213783
14	8	0	5.774570	-1.095702	1.092873
15	1	0	5.778932	-0.190416	1.430237
16	1	0	6.673785	-1.240708	0.770484
17	7	0	-3.275669	-0.875252	0.278647
18	8	0	-3.618185	-2.015583	0.616685
19	8	0	-4.107608	0.035870	0.138608
20	6	0	0.197571	2.637710	-0.221190
21	1	0	1.010663	2.729943	-0.947274
22	6	0	0.757921	3.048086	1.155295
23	1	0	1.582331	2.401142	1.464694
24	1	0	1.128481	4.076999	1.122954
25	1	0	-0.023834	2.992520	1.919456
26	6	0	-0.921156	3.592326	-0.654809
27	1	0	-1.340271	3.308871	-1.624376
28	1	0	-1.736944	3.623818	0.073393
29	1	0	-0.523366	4.606979	-0.743097
30	6	0	-1.207728	-3.096135	-0.112434
31	1	0	-1.494399	-3.441661	0.883439
32	1	0	-2.027168	-3.355243	-0.786133
33	1	0	-0.312974	-3.637044	-0.423207

Rotational constants (GHZ): 0.5649846 0.2765779 0.1987812

4-nitrothymolate
Electronic Energy -822.391872
Stoichiometry C10H16NO5(1-)
Framework group C1[X(C10H16NO5)]
Deg. of freedom 90
Full point group C1 NOp 1
Largest Abelian subgroup C1 NOp 1
Largest concise Abelian subgroup C1 NOp 1
Standard orientation:

Center Number	Atomic Number	Atomic Type	Coordinates (Angstroms) X	Y	Z
1	6	0	0.977393	-1.031607	-0.066050
2	6	0	-0.376600	-0.814091	-0.090788

3	6	0	-0.847452	0.553952	-0.069907
4	6	0	0.133720	1.592593	-0.025739
5	6	0	1.497174	1.380869	0.001272
6	6	0	1.928279	0.019030	-0.018942
7	1	0	1.357909	-2.042265	-0.084526
8	1	0	-0.236446	2.612216	-0.014023
9	8	0	-2.099892	0.844742	-0.094993
10	8	0	-3.121853	3.362708	0.058771
11	1	0	-2.403697	4.001485	-0.018661
12	1	0	-2.680505	2.479645	0.000105
13	8	0	-4.648261	-0.174613	-0.315159
14	1	0	-3.704830	0.089714	-0.239527
15	1	0	-5.125360	0.659333	-0.399603
16	7	0	3.288434	-0.351513	0.001786
17	8	0	4.185595	0.520969	0.035685
18	8	0	3.602808	-1.568854	-0.011061
19	6	0	-1.388749	-1.947069	-0.108011
20	1	0	-2.226982	-1.607719	-0.724182
21	6	0	-1.936911	-2.207063	1.309928
22	1	0	-2.338122	-1.296661	1.761213
23	1	0	-2.738223	-2.951833	1.281261
24	1	0	-1.143681	-2.587782	1.961636
25	6	0	-0.860227	-3.248445	-0.723267
26	1	0	-0.462922	-3.087220	-1.729522
27	1	0	-0.069866	-3.697781	-0.114343
28	1	0	-1.671076	-3.978954	-0.794613
29	6	0	2.402371	2.584724	0.047729
30	1	0	3.035836	2.579568	0.937430
31	1	0	3.071773	2.620010	-0.814529
32	1	0	1.795999	3.491858	0.056091

Rotational constants (GHZ): 0.6409108 0.3169995 0.2201699

- $2H_2O$/B3LYP 6-311G+dp/PCM

H_2O

Electronic Energy -229.411585
Stoichiometry H6O3
Framework group C1[X(H6O3)]
Deg. of freedom 21
Full point group C1 NOp 1
Largest Abelian subgroup C1 NOp 1
Largest concise Abelian subgroup C1 NOp 1

Standard orientation:

Center Number	Atomic Number	Atomic Type	Coordinates (Angstroms) X	Y	Z
1	8	0	2.252207	-0.617414	-0.000217
2	1	0	1.885750	-1.507876	-0.001218
3	1	0	1.478583	-0.027246	-0.000007
4	8	0	-0.002259	1.183651	0.000256
5	1	0	-0.006374	1.769886	-0.767289
6	1	0	-0.006471	1.770013	0.767703
7	8	0	-2.118421	-0.770095	0.001452
8	1	0	-1.442815	-0.069801	0.000397
9	1	0	-2.960887	-0.304115	-0.011519

Rotational constants (GHZ): 10.5713991 2.9583424 2.3368711

OH^-

Electronic Energy -228.930353
Stoichiometry H5O3(1-)
Framework group C1[X(H5O3)]
Deg. of freedom 18
Full point group C1 NOp 1
Largest Abelian subgroup C1 NOp 1
Largest concise Abelian subgroup C1 NOp 1

Standard orientation:

Center Number	Atomic Number	Atomic Type	Coordinates (Angstroms) X	Y	Z
1	8	0	-2.206177	-0.500235	0.079535
2	1	0	-1.337317	0.044429	0.037315
3	1	0	-2.740320	-0.191497	-0.658468
4	8	0	-0.002751	0.823110	-0.042352
5	1	0	-0.001224	1.616774	0.502239
6	8	0	2.263495	-0.395997	-0.099074
7	1	0	1.365492	0.097713	-0.049248
8	1	0	2.276828	-0.982437	0.663289

Rotational constants (GHZ): 22.6190704 2.8439268 2.5611181

Phenol
Electronic Energy -460.513121
Stoichiometry C6H10O3
Framework group C1[X(C6H10O3)]
Deg. of freedom 51
Full point group C1 NOp 1
Largest Abelian subgroup C1 NOp 1
Largest concise Abelian subgroup C1 NOp 1
Standard orientation:

Center Number	Atomic Number	Atomic Type	Coordinates (Angstroms) X	Y	Z
1	6	0	0.841640	1.155245	0.089364
2	6	0	0.151589	-0.042189	-0.124440
3	6	0	0.852919	-1.247448	-0.219956
4	6	0	2.240842	-1.250346	-0.100747
5	6	0	2.939472	-0.060417	0.113063
6	6	0	2.231434	1.137811	0.206095
7	1	0	0.292544	2.087162	0.161383
8	1	0	2.777776	-2.189414	-0.176244
9	1	0	4.019077	-0.067623	0.204967
10	8	0	-1.211126	-0.082095	-0.248574
11	1	0	-1.610516	0.813840	-0.170591
12	8	0	-2.973442	-2.245386	0.369748
13	1	0	-3.088698	-2.711632	-0.464985
14	1	0	-2.367509	-1.511372	0.163660
15	8	0	-2.407424	2.371527	-0.052743
16	1	0	-2.910987	2.548041	0.751030
17	1	0	-2.989395	2.620753	-0.780904
18	1	0	2.759998	2.070157	0.371555
19	1	0	0.306280	-2.168210	-0.387586

Rotational constants (GHZ): 1.7519272 0.8822399 0.5959856

Phenolate
Electronic Energy -460.046389
Stoichiometry C6H9O3(1-)
Framework group C1[X(C6H9O3)]
Deg. of freedom 48
Full point group C1 NOp 1
Largest Abelian subgroup C1 NOp 1
Largest concise Abelian subgroup C1 NOp 1
Standard orientation:

Center Number	Atomic Number	Atomic Type	Coordinates (Angstroms) X	Y	Z
1	6	0	0.814211	1.206499	-0.061315
2	6	0	0.062810	0.000031	-0.000449
3	6	0	0.814091	-1.206493	0.060834
4	6	0	2.206263	-1.199452	0.060512
5	6	0	2.923320	-0.000075	0.000380
6	6	0	2.206384	1.199352	-0.060182
7	1	0	0.274887	2.147526	-0.109400

8	1	0	2.740158	-2.144344	0.108754
9	1	0	4.007316	-0.000111	0.000698
10	8	0	-1.249989	0.000068	-0.000837
11	8	0	-2.784421	2.159751	0.169682
12	1	0	-2.659733	2.671430	-0.635627
13	1	0	-2.164336	1.379920	0.085047
14	8	0	-2.784463	-2.159853	-0.168944
15	1	0	-2.164424	-1.379909	-0.085195
16	1	0	-2.660392	-2.670129	0.637351
17	1	0	0.274659	-2.147475	0.108603
18	1	0	2.740376	2.144204	-0.108118

Rotational constants (GHZ): 1.9631148 0.8937955 0.6170292

Thymol

Electronic Energy -617.812865
Stoichiometry C10H18O3
Framework group C1[X(C10H18O3)]
Deg. of freedom 87
Full point group C1 NOp 1
Largest Abelian subgroup C1 NOp 1
Largest concise Abelian subgroup C1 NOp 1
Standard orientation:

Center Number	Atomic Number	Atomic Type	Coordinates (Angstroms) X	Y	Z
1	6	0	1.656500	0.274600	-0.007448
2	6	0	0.265766	0.291450	0.103174
3	6	0	-0.491084	-0.893491	0.024609
4	6	0	0.220396	-2.079623	-0.168940
5	6	0	1.612572	-2.105609	-0.276762
6	6	0	2.352302	-0.924572	-0.195785
7	1	0	2.198167	1.213213	0.056202
8	1	0	-0.321421	-3.015800	-0.236641
9	1	0	2.121015	-3.052374	-0.425416
10	8	0	-0.407410	1.477509	0.287738
11	1	0	0.219761	2.214175	0.460382
12	6	0	-2.003972	-0.858751	0.200633
13	1	0	-2.355709	0.090547	-0.212495
14	6	0	-2.377067	-0.869309	1.697667
15	1	0	-1.897128	-0.045989	2.231847
16	1	0	-3.459692	-0.772894	1.825923
17	1	0	-2.062890	-1.807721	2.165678
18	6	0	-2.740784	-1.978669	-0.548435
19	1	0	-3.821016	-1.826307	-0.472752
20	1	0	-2.475556	-1.995310	-1.609069
21	1	0	-2.522489	-2.964948	-0.128706
22	6	0	3.857685	-0.923713	-0.317932
23	1	0	4.182562	-0.371823	-1.205695
24	1	0	4.326236	-0.445261	0.547286
25	1	0	4.247779	-1.940437	-0.394335
26	8	0	-2.114616	2.617173	-1.727486
27	1	0	-1.580511	2.197391	-1.029787
28	1	0	-2.973816	2.774129	-1.321872
29	8	0	1.236370	3.628738	0.786017
30	1	0	1.242912	3.966027	1.689963
31	1	0	1.103170	4.402146	0.224656

Rotational constants (GHZ): 0.6494772 0.5774567 0.3599864

Thymolate

Electronic Energy -617.342935
Stoichiometry C10H17O3(1-)
Framework group C1[X(C10H17O3)]
Deg. of freedom 84
Full point group C1 NOp 1
Largest Abelian subgroup C1 NOp 1
Largest concise Abelian subgroup C1 NOp 1

Standard orientation:

Center Number	Atomic Number	Atomic Type	Coordinates (Angstroms) X	Y	Z
1	6	0	-1.564293	0.826235	-0.020035
2	6	0	-0.196255	0.443050	-0.062908
3	6	0	0.091875	-0.961435	-0.078845
4	6	0	-0.972505	-1.862564	-0.033524
5	6	0	-2.309664	-1.456435	0.018348
6	6	0	-2.614727	-0.090400	0.026347
7	1	0	-1.784886	1.890567	-0.019968
8	1	0	-0.760233	-2.927731	-0.044801
9	1	0	-3.103397	-2.196446	0.046155
10	8	0	0.748838	1.356811	-0.092854
11	6	0	1.538415	-1.434825	-0.107169
12	1	0	2.124324	-0.621430	-0.542000
13	6	0	1.771100	-2.679866	-0.979071
14	1	0	1.400597	-2.528447	-1.997122
15	1	0	2.840666	-2.904754	-1.039318
16	1	0	1.276575	-3.566495	-0.570603
17	6	0	2.076582	-1.669787	1.319332
18	1	0	3.140905	-1.928572	1.301539
19	1	0	1.956057	-0.777336	1.938918
20	1	0	1.538185	-2.491297	1.804174
21	6	0	-4.048450	0.385393	0.113532
22	1	0	-4.350757	0.545094	1.155031
23	1	0	-4.188810	1.333600	-0.412112
24	1	0	-4.737922	-0.346767	-0.314791
25	8	0	0.331809	3.977584	-0.009183
26	1	0	-0.180855	4.224666	-0.785117
27	1	0	0.423660	2.981828	-0.059184
28	8	0	3.405239	1.688898	-0.086186
29	1	0	2.433472	1.481708	-0.083433
30	1	0	3.452871	2.649275	-0.047628

Rotational constants (GHZ): 0.7163425 0.5788882 0.3417463

- $2H_2O$/ B3LYP 6-311G+dp/CPCM

H_2O

Electronic Energy -229.411687
Stoichiometry H6O3
Framework group C1[X(H6O3)]
Deg. of freedom 21
Full point group C1 NOp 1
Largest Abelian subgroup C1 NOp 1
Largest concise Abelian subgroup C1 NOp 1
Standard orientation:

Center Number	Atomic Number	Atomic Type	Coordinates (Angstroms) X	Y	Z
1	8	0	2.252693	-0.617041	-0.000269
2	1	0	1.886759	-1.507732	-0.001290
3	1	0	1.478717	-0.027309	-0.000070
4	8	0	-0.002426	1.183267	0.000232
5	1	0	-0.006791	1.769541	-0.767287
6	1	0	-0.006839	1.769704	0.767626
7	8	0	-2.118803	-0.770052	0.001581
8	1	0	-1.442680	-0.070235	0.000435
9	1	0	-2.960879	-0.303355	-0.011772

Rotational constants (GHZ): 10.5772216 2.9572214 2.3364534

OH^-

Electronic Energy -228.930472
Stoichiometry H5O3(1-)
Framework group C1[X(H5O3)]
Deg. of freedom 18

Full point group C1 NOp 1
Largest Abelian subgroup C1 NOp 1
Largest concise Abelian subgroup C1 NOp 1
Standard orientation:

Center Number	Atomic Number	Atomic Type	Coordinates (Angstroms) X	Y	Z
1	8	0	-2.199979	-0.503518	0.079539
2	1	0	-1.339922	0.054683	0.034583
3	1	0	-2.743830	-0.199186	-0.653209
4	8	0	-0.002584	0.828923	-0.042875
5	1	0	0.000754	1.622460	0.501907
6	8	0	2.258546	-0.399490	-0.099219
7	1	0	1.363133	0.098680	-0.047518
8	1	0	2.272001	-0.983956	0.664681

Rotational constants (GHZ): 22.3472549 2.8568217 2.5678934

Phenol
Electronic Energy -460.513184
Stoichiometry C6H10O3
Framework group C1[X(C6H10O3)]
Deg. of freedom 51
Full point group C1 NOp 1
Largest Abelian subgroup C1 NOp 1
Largest concise Abelian subgroup C1 NOp 1
Standard orientation:

Center Number	Atomic Number	Atomic Type	Coordinates (Angstroms) X	Y	Z
1	6	0	0.841640	1.155245	0.089364
2	6	0	0.151589	-0.042189	-0.124440
3	6	0	0.852919	-1.247448	-0.219956
4	6	0	2.240842	-1.250346	-0.100747
5	6	0	2.939472	-0.060417	0.113063
6	6	0	2.231434	1.137811	0.206095
7	1	0	0.292544	2.087162	0.161383
8	1	0	2.777776	-2.189414	-0.176244
9	1	0	4.019077	-0.067623	0.204967
10	8	0	-1.211126	-0.082095	-0.248574
11	1	0	-1.610516	0.813840	-0.170591
12	8	0	-2.973442	-2.245386	0.369748
13	1	0	-3.088698	-2.711632	-0.464985
14	1	0	-2.367509	-1.511372	0.163660
15	8	0	-2.407424	2.371527	-0.052743
16	1	0	-2.910987	2.548041	0.751030
17	1	0	-2.989395	2.620753	-0.780904
18	1	0	2.759998	2.070157	0.371555
19	1	0	0.306280	-2.168210	-0.387586

Rotational constants (GHZ): 1.7519272 0.8822399 0.5959856

Phenolate
Electronic Energy -460.046575
Stoichiometry C6H9O3(1-)
Framework group C1[X(C6H9O3)]
Deg. of freedom 48
Full point group C1 NOp 1
Largest Abelian subgroup C1 NOp 1
Largest concise Abelian subgroup C1 NOp 1
Standard orientation:

Center Number	Atomic Number	Atomic Type	Coordinates (Angstroms) X	Y	Z
1	6	0	0.814211	1.206499	-0.061315
2	6	0	0.062810	0.000031	-0.000449
3	6	0	0.814091	-1.206493	0.060834
4	6	0	2.206263	-1.199452	0.060512

5	6	0	2.923320	-0.000075	0.000380
6	6	0	2.206384	1.199352	-0.060182
7	1	0	0.274887	2.147526	-0.109400
8	1	0	2.740158	-2.144344	0.108754
9	1	0	4.007316	-0.000111	0.000698
10	8	0	-1.249989	0.000068	-0.000837
11	8	0	-2.784421	2.159751	0.169682
12	1	0	-2.659733	2.671430	-0.635627
13	1	0	-2.164336	1.379920	0.085047
14	8	0	-2.784463	-2.159853	-0.168944
15	1	0	-2.164424	-1.379909	-0.085195
16	1	0	-2.660392	-2.670129	0.637351
17	1	0	0.274659	-2.147475	0.108603
18	1	0	2.740376	2.144204	-0.108118

Rotational constants (GHZ): 1.9631148 0.8937955 0.6170292

Thymol
Electronic Energy -617.812974
Stoichiometry C10H18O3
Framework group C1[X(C10H18O3)]
Deg. of freedom 87
Full point group C1 NOp 1
Largest Abelian subgroup C1 NOp 1
Largest concise Abelian subgroup C1 NOp 1
Standard orientation:

Center Number	Atomic Number	Atomic Type	Coordinates (Angstroms) X	Y	Z
1	6	0	1.657090	0.272018	-0.006869
2	6	0	0.266394	0.291497	0.104070
3	6	0	-0.492725	-0.891993	0.025011
4	6	0	0.216454	-2.079392	-0.169322
5	6	0	1.608563	-2.107991	-0.277490
6	6	0	2.350565	-0.928390	-0.196021
7	1	0	2.200599	1.209535	0.057171
8	1	0	-0.327171	-3.014510	-0.237299
9	1	0	2.115157	-3.055652	-0.426732
10	8	0	-0.404474	1.478696	0.289425
11	1	0	0.224034	2.214254	0.462074
12	6	0	-2.005486	-0.854686	0.201626
13	1	0	-2.355519	0.096476	-0.208577
14	6	0	-2.378004	-0.868844	1.698772
15	1	0	-1.896198	-0.048034	2.235162
16	1	0	-3.460370	-0.770577	1.827732
17	1	0	-2.065545	-1.809244	2.163939
18	6	0	-2.744720	-1.971034	-0.550422
19	1	0	-3.824627	-1.816804	-0.473899
20	1	0	-2.479910	-1.985089	-1.611211
21	1	0	-2.528154	-2.958963	-0.133706
22	6	0	3.855948	-0.930356	-0.318164
23	1	0	4.182052	-0.376332	-1.204135
24	1	0	4.325460	-0.455603	0.548578
25	1	0	4.243872	-1.947663	-0.397695
26	8	0	-2.110670	2.616277	-1.727816
27	1	0	-1.576846	2.199628	-1.028034
28	1	0	-2.966109	2.786312	-1.319505
29	8	0	1.243174	3.627812	0.783854
30	1	0	1.256376	3.961987	1.688898
31	1	0	1.104193	4.403014	0.226374

Rotational constants (GHZ): 0.6496469 0.5774292 0.3600687

Thymolate
Electronic Energy -617.343125
Stoichiometry C10H17O3(1-)
Framework group C1[X(C10H17O3)]
Deg. of freedom 84
Full point group C1 NOp 1
Largest Abelian subgroup C1 NOp 1
Largest concise Abelian subgroup C1 NOp 1

Standard orientation:

Center Number	Atomic Number	Atomic Type	Coordinates (Angstroms) X	Y	Z
1	6	0	-1.564293	0.826235	-0.020035
2	6	0	-0.196255	0.443050	-0.062908
3	6	0	0.091875	-0.961435	-0.078845
4	6	0	-0.972505	-1.862564	-0.033524
5	6	0	-2.309664	-1.456435	0.018348
6	6	0	-2.614727	-0.090400	0.026347
7	1	0	-1.784886	1.890567	-0.019968
8	1	0	-0.760233	-2.927731	-0.044801
9	1	0	-3.103397	-2.196446	0.046155
10	8	0	0.748838	1.356811	-0.092854
11	6	0	1.538415	-1.434825	-0.107169
12	1	0	2.124324	-0.621430	-0.542000
13	6	0	1.771100	-2.679866	-0.979071
14	1	0	1.400597	-2.528447	-1.997122
15	1	0	2.840666	-2.904754	-1.039318
16	1	0	1.276575	-3.566495	-0.570603
17	6	0	2.076582	-1.669787	1.319332
18	1	0	3.140905	-1.928572	1.301539
19	1	0	1.956057	-0.777336	1.938918
20	1	0	1.538185	-2.491297	1.804174
21	6	0	-4.048450	0.385393	0.113532
22	1	0	-4.350757	0.545094	1.155031
23	1	0	-4.188810	1.333600	-0.412112
24	1	0	-4.737922	-0.346767	-0.314791
25	8	0	0.331809	3.977584	-0.009183
26	1	0	-0.180855	4.224666	-0.785117
27	1	0	0.423660	2.981828	-0.059184
28	8	0	3.405239	1.688898	-0.086186
29	1	0	2.433472	1.481708	-0.083433
30	1	0	3.452871	2.649275	-0.047628

Rotational constants (GHZ): 0.7163425 0.5788882 0.3417463

- $2H_2O$/ B3PW91 6-311G+dp/SMD

H_2O

Electronic Energy -229.332264
Stoichiometry H6O3
Framework group C1[X(H6O3)]
Deg. of freedom 21
Full point group C1 NOp 1
Largest Abelian subgroup C1 NOp 1
Largest concise Abelian subgroup C1 NOp 1

Standard orientation:

Center Number	Atomic Number	Atomic Type	Coordinates (Angstroms) X	Y	Z
1	8	0	-2.494935	-0.448565	-0.004972
2	1	0	-2.295456	-1.386131	0.083572
3	1	0	-1.621942	-0.012346	-0.014804
4	8	0	0.001211	0.893130	-0.008879
5	1	0	0.011465	1.416860	0.801604
6	1	0	0.016473	1.545600	-0.720069
7	8	0	2.394287	-0.627847	-0.027832
8	1	0	1.585637	-0.081611	-0.024298
9	1	0	3.099321	-0.016113	0.207454

Rotational constants (GHZ): 16.9597454 2.3759513 2.1053937

OH^-

Electronic Energy -228.850093
Stoichiometry H5O3(1-)
Framework group C1[X(H5O3)]
Deg. of freedom 18

Full point group C1 NOp 1
Largest Abelian subgroup C1 NOp 1
Largest concise Abelian subgroup C1 NOp 1
Standard orientation:

Center Number	Atomic Number	Atomic Type	Coordinates (Angstroms) X	Y	Z
1	8	0	-2.185914	-0.510229	0.071110
2	1	0	-1.328366	0.047024	0.017619
3	1	0	-2.774519	-0.127146	-0.586148
4	8	0	-0.001640	0.840823	-0.059223
5	1	0	0.002555	1.494137	0.647085
6	8	0	2.252213	-0.391012	-0.094332
7	1	0	1.351543	0.095095	-0.046993
8	1	0	2.231512	-1.025763	0.627995

Rotational constants (GHZ): 22.3431386 2.8811704 2.5882475

Phenol
Electronic Energy -460.335848
Stoichiometry C6H10O3
Framework group C1[X(C6H10O3)]
Deg. of freedom 51
Full point group C1 NOp 1
Largest Abelian subgroup C1 NOp 1
Largest concise Abelian subgroup C1 NOp 1
Standard orientation:

Center Number	Atomic Number	Atomic Type	Coordinates (Angstroms) X	Y	Z
1	6	0	-0.691700	-1.163850	0.255906
2	6	0	-0.144793	0.004326	-0.278666
3	6	0	-0.966429	1.099723	-0.551430
4	6	0	-2.330127	1.022059	-0.289458
5	6	0	-2.886421	-0.140506	0.242313
6	6	0	-2.059143	-1.228507	0.511270
7	1	0	-0.050165	-2.012625	0.468345
8	1	0	-2.961659	1.878177	-0.505432
9	1	0	-3.950721	-0.197291	0.443930
10	8	0	1.188214	0.128245	-0.551042
11	1	0	1.683029	-0.706778	-0.359852
12	8	0	2.590492	2.310714	0.643253
13	1	0	1.929181	2.965753	0.888966
14	1	0	2.077980	1.589268	0.236445
15	8	0	2.735904	-2.054161	-0.139990
16	1	0	3.190812	-1.999801	0.708859
17	1	0	3.441983	-2.014740	-0.796144
18	1	0	-2.477189	-2.140572	0.925779
19	1	0	-0.528448	2.000757	-0.968269

Rotational constants (GHZ): 1.8201443 0.8808976 0.6331429

Phenolate
Electronic Energy -459.867467
Stoichiometry C6H9O3(1-)
Framework group C1[X(C6H9O3)]
Deg. of freedom 48
Full point group C1 NOp 1
Largest Abelian subgroup C1 NOp 1
Largest concise Abelian subgroup C1 NOp 1
Standard orientation:

Center Number	Atomic Number	Atomic Type	Coordinates (Angstroms) X	Y	Z
1	6	0	0.768244	1.205074	-0.032149
2	6	0	0.024168	-0.000678	-0.072008
3	6	0	0.767819	-1.207033	-0.045169
4	6	0	2.156803	-1.200806	0.018530

5	6	0	2.870907	-0.001818	0.057028
6	6	0	2.157205	1.197763	0.031409
7	1	0	0.228913	2.148366	-0.052865
8	1	0	2.690209	-2.147800	0.036743
9	1	0	3.954804	-0.002262	0.105659
10	8	0	-1.291461	0.000045	-0.134688
11	8	0	-2.708571	2.226574	0.090207
12	1	0	-2.121480	2.967596	-0.089040
13	1	0	-2.128153	1.418126	-0.001308
14	8	0	-2.719498	-2.224197	0.043470
15	1	0	-2.133321	-1.417526	-0.022318
16	1	0	-2.124872	-2.935267	0.301713
17	1	0	0.228385	-2.149958	-0.076382
18	1	0	2.690882	2.144347	0.060039

Rotational constants (GHZ): 1.8802259 0.9420649 0.6287416

Thymol

Electronic Energy -617.577213
Stoichiometry C10H18O3
Framework group C1[X(C10H18O3)]
Deg. of freedom 87
Full point group C1 NOp 1
Largest Abelian subgroup C1 NOp 1
Largest concise Abelian subgroup C1 NOp 1

Standard orientation:

Center Number	Atomic Number	Atomic Type	Coordinates (Angstroms) X	Y	Z
1	6	0	1.699738	0.126285	0.054921
2	6	0	0.326228	0.308443	0.230810
3	6	0	-0.565397	-0.771418	0.134581
4	6	0	-0.013186	-2.023511	-0.146249
5	6	0	1.353828	-2.212393	-0.324746
6	6	0	2.233647	-1.131407	-0.226565
7	1	0	2.358901	0.985617	0.143108
8	1	0	-0.676274	-2.880799	-0.221879
9	1	0	1.739450	-3.205138	-0.537519
10	8	0	-0.181226	1.551710	0.507831
11	1	0	0.538376	2.225240	0.581177
12	6	0	-2.059896	-0.603931	0.340223
13	1	0	-2.248650	0.452569	0.545148
14	6	0	-2.552367	-1.403633	1.551905
15	1	0	-2.018574	-1.115346	2.462660
16	1	0	-3.620696	-1.229207	1.716934
17	1	0	-2.408881	-2.478861	1.402552
18	6	0	-2.849590	-0.979937	-0.918706
19	1	0	-3.918582	-0.796512	-0.768662
20	1	0	-2.524250	-0.395275	-1.784978
21	1	0	-2.724934	-2.040102	-1.163203
22	6	0	3.711177	-1.303911	-0.445348
23	1	0	3.981372	-1.077359	-1.483293
24	1	0	4.291662	-0.633528	0.194010
25	1	0	4.024146	-2.331142	-0.243084
26	8	0	-1.977946	2.702440	-1.390660
27	1	0	-1.388764	2.281129	-0.738641
28	1	0	-2.585131	1.999454	-1.645041
29	8	0	1.584332	3.593490	0.781525
30	1	0	1.577799	4.140220	-0.013151
31	1	0	2.496648	3.290390	0.859320

Rotational constants (GHZ): 0.6738635 0.5826735 0.3638672

Thymolate

Electronic Energy- -617.105470
Stoichiometry C10H17O3(1-)
Framework group C1[X(C10H17O3)]
Deg. of freedom 84
Full point group C1 NOp 1
Largest Abelian subgroup C1 NOp 1
Largest concise Abelian subgroup C1 NOp 1

Standard orientation:

Center Number	Atomic Number	Atomic Type	Coordinates (Angstroms) X	Y	Z
1	6	0	-1.570625	0.786966	-0.064020
2	6	0	-0.203986	0.426879	-0.129765
3	6	0	0.117318	-0.965593	-0.099225
4	6	0	-0.922979	-1.885912	0.007957
5	6	0	-2.264759	-1.503946	0.081250
6	6	0	-2.600104	-0.148668	0.040894
7	1	0	-1.814590	1.847053	-0.094524
8	1	0	-0.686231	-2.946273	0.036788
9	1	0	-3.042856	-2.257416	0.165461
10	8	0	0.725847	1.357810	-0.219647
11	6	0	1.570478	-1.398419	-0.136220
12	1	0	2.108766	-0.644239	-0.720212
13	6	0	1.801150	-2.750286	-0.812970
14	1	0	1.373486	-2.775670	-1.820340
15	1	0	2.874539	-2.948306	-0.899458
16	1	0	1.364989	-3.575607	-0.240724
17	6	0	2.173891	-1.400164	1.276280
18	1	0	3.246828	-1.620089	1.245965
19	1	0	2.042054	-0.433648	1.770835
20	1	0	1.691449	-2.163631	1.897279
21	6	0	-4.037497	0.298886	0.084814
22	1	0	-4.174013	1.145007	0.765216
23	1	0	-4.381511	0.624112	-0.903927
24	1	0	-4.695179	-0.510667	0.411784
25	8	0	0.193006	3.934603	0.011093
26	1	0	-0.762281	4.049255	0.024214
27	1	0	0.327871	2.945737	-0.073554
28	8	0	3.374678	1.743593	-0.139970
29	1	0	2.410616	1.512475	-0.157454
30	1	0	3.390496	2.705403	-0.113134

Rotational constants (GHZ): 0.7330949 0.5838275 0.3445364

- $2H_2O$/ B3PW91 6-311G+dp/PCM

H_2O

Electronic Energy -229.318876
Stoichiometry H6O3
Framework group C1[X(H6O3)]
Deg. of freedom 21
Full point group C1 NOp 1
Largest Abelian subgroup C1 NOp 1
Largest concise Abelian subgroup C1 NOp 1

Standard orientation:

Center Number	Atomic Number	Atomic Type	Coordinates (Angstroms) X	Y	Z
1	8	0	-2.494935	-0.448565	-0.004972
2	1	0	-2.295456	-1.386131	0.083572
3	1	0	-1.621942	-0.012346	-0.014804
4	8	0	0.001211	0.893130	-0.008879
5	1	0	0.011465	1.416860	0.801604
6	1	0	0.016473	1.545600	-0.720069
7	8	0	2.394287	-0.627847	-0.027832
8	1	0	1.585637	-0.081611	-0.024298
9	1	0	3.099321	-0.016113	0.207454

Rotational constants (GHZ): 16.9597454 2.3759513 2.1053937

OH^-

Electronic Energy -228.834438
Stoichiometry H5O3(1-)
Framework group C1[X(H5O3)]
Deg. of freedom 18

Full point group C1 NOp 1
Largest Abelian subgroup C1 NOp 1
Largest concise Abelian subgroup C1 NOp 1
Standard orientation:

Center Number	Atomic Number	Atomic Type	Coordinates (Angstroms) X	Y	Z
1	8	0	-2.185914	-0.510229	0.071110
2	1	0	-1.328366	0.047024	0.017619
3	1	0	-2.774519	-0.127146	-0.586148
4	8	0	-0.001640	0.840823	-0.059223
5	1	0	0.002555	1.494137	0.647085
6	8	0	2.252213	-0.391012	-0.094332
7	1	0	1.351543	0.095095	-0.046993
8	1	0	2.231512	-1.025763	0.627995

Rotational constants (GHZ): 22.3431386 2.8811704 2.5882475

Phenol
Electronic Energy -460.326711
Stoichiometry C6H10O3
Framework group C1[X(C6H10O3)]
Deg. of freedom 51
Full point group C1 NOp 1
Largest Abelian subgroup C1 NOp 1
Largest concise Abelian subgroup C1 NOp 1
Standard orientation:

Center Number	Atomic Number	Atomic Type	Coordinates (Angstroms) X	Y	Z
1	6	0	-0.691700	-1.163850	0.255906
2	6	0	-0.144793	0.004326	-0.278666
3	6	0	-0.966429	1.099723	-0.551430
4	6	0	-2.330127	1.022059	-0.289458
5	6	0	-2.886421	-0.140506	0.242313
6	6	0	-2.059143	-1.228507	0.511270
7	1	0	-0.050165	-2.012625	0.468345
8	1	0	-2.961659	1.878177	-0.505432
9	1	0	-3.950721	-0.197291	0.443930
10	8	0	1.188214	0.128245	-0.551042
11	1	0	1.683029	-0.706778	-0.359852
12	8	0	2.590492	2.310714	0.643253
13	1	0	1.929181	2.965753	0.888966
14	1	0	2.077980	1.589268	0.236445
15	8	0	2.735904	-2.054161	-0.139990
16	1	0	3.190812	-1.999801	0.708859
17	1	0	3.441983	-2.014740	-0.796144
18	1	0	-2.477189	-2.140572	0.925779
19	1	0	-0.528448	2.000757	-0.968269

Rotational constants (GHZ): 1.8201443 0.8808976 0.6331429

Phenolate
Electronic Energy -459.858007
Stoichiometry C6H9O3(1-)
Framework group C1[X(C6H9O3)]
Deg. of freedom 48
Full point group C1 NOp 1
Largest Abelian subgroup C1 NOp 1
Largest concise Abelian subgroup C1 NOp 1
Standard orientation:

Center Number	Atomic Number	Atomic Type	Coordinates (Angstroms) X	Y	Z
1	6	0	-0.807930	-1.204678	-0.047769
2	6	0	-0.056705	-0.000004	-0.000036
3	6	0	-0.807930	1.204668	0.047732
4	6	0	-2.197922	1.197712	0.047175

5	6	0	-2.914369	-0.000006	0.000030
6	6	0	-2.197923	-1.197723	-0.047148
7	1	0	-0.268408	-2.147227	-0.085361
8	1	0	-2.731774	2.144292	0.084953
9	1	0	-3.999345	-0.000007	0.000056
10	8	0	1.251023	-0.000002	-0.000069
11	8	0	2.768464	-2.156645	0.155698
12	1	0	2.644215	-2.652039	-0.657539
13	1	0	2.152402	-1.373001	0.078373
14	8	0	2.768422	2.156694	-0.155625
15	1	0	2.152394	1.372999	-0.078536
16	1	0	2.644099	2.651887	0.657724
17	1	0	-0.268407	2.147218	0.085296
18	1	0	-2.731775	-2.144305	-0.084901

Rotational constants (GHZ): 1.9711824 0.9020585 0.6214956

Thymol

Electronic Energy -617.569276

Stoichiometry C10H18O3

Framework group C1[X(C10H18O3)]

Deg. of freedom 87

Full point group C1 NOp 1

Largest Abelian subgroup C1 NOp 1

Largest concise Abelian subgroup C1 NOp 1

Standard orientation:

Center Number	Atomic Number	Atomic Type	Coordinates (Angstroms) X	Y	Z
1	6	0	1.651783	0.289156	0.006594
2	6	0	0.262124	0.296732	0.113453
3	6	0	-0.483004	-0.892687	0.024502
4	6	0	0.236179	-2.070243	-0.176014
5	6	0	1.626258	-2.085946	-0.280916
6	6	0	2.355781	-0.901363	-0.189582
7	1	0	2.187269	1.231785	0.080322
8	1	0	-0.300973	-3.010087	-0.251090
9	1	0	2.143060	-3.028547	-0.434823
10	8	0	-0.417634	1.468592	0.303644
11	1	0	0.204932	2.211830	0.470709
12	6	0	-1.990411	-0.871254	0.199215
13	1	0	-2.348736	0.079693	-0.207678
14	6	0	-2.358732	-0.895822	1.690833
15	1	0	-1.884179	-0.072494	2.230877
16	1	0	-3.442198	-0.809909	1.823348
17	1	0	-2.035154	-1.835567	2.150888
18	6	0	-2.713074	-1.989043	-0.554315
19	1	0	-3.795364	-1.846722	-0.481662
20	1	0	-2.444329	-1.999957	-1.614665
21	1	0	-2.488516	-2.975729	-0.137141
22	6	0	3.855325	-0.888373	-0.316918
23	1	0	4.167294	-0.418822	-1.256071
24	1	0	4.319660	-0.321696	0.495572
25	1	0	4.261986	-1.901854	-0.300651
26	8	0	-2.127729	2.585559	-1.727480
27	1	0	-1.589976	2.176783	-1.028338
28	1	0	-2.969759	2.777655	-1.305756
29	8	0	1.190522	3.623320	0.781934
30	1	0	1.096640	4.031220	1.649237
31	1	0	1.103702	4.345692	0.151021

Rotational constants (GHZ): 0.6540042 0.5811268 0.3626310

Thymolate

Electronic Energy- -617.098204

Stoichiometry C10H17O3(1-)

Framework group C1[X(C10H17O3)]

Deg. of freedom 84

Full point group C1 NOp 1

Largest Abelian subgroup C1 NOp 1

Largest concise Abelian subgroup C1 NOp 1

Standard orientation:

Center Number	Atomic Number	Atomic Type	Coordinates (Angstroms) X	Y	Z
1	6	0	1.626243	0.482127	-0.118661
2	6	0	0.219895	0.437627	0.058958
3	6	0	-0.393927	-0.851560	0.100021
4	6	0	0.409836	-1.981922	-0.037324
5	6	0	1.792053	-1.909315	-0.211526
6	6	0	2.416032	-0.658005	-0.248807
7	1	0	2.093695	1.463998	-0.156825
8	1	0	-0.058327	-2.964182	-0.009013
9	1	0	2.378495	-2.817801	-0.318428
10	8	0	-0.476422	1.544683	0.181604
11	6	0	-1.891859	-0.978238	0.304333
12	1	0	-2.281442	0.043342	0.343961
13	6	0	-2.231106	-1.654871	1.639338
14	1	0	-1.781682	-1.117951	2.480404
15	1	0	-3.314781	-1.685763	1.798396
16	1	0	-1.861813	-2.685994	1.666161
17	6	0	-2.582769	-1.699932	-0.859284
18	1	0	-3.668702	-1.720615	-0.715500
19	1	0	-2.377821	-1.202631	-1.812077
20	1	0	-2.241531	-2.737427	-0.944572
21	6	0	3.911865	-0.543684	-0.398254
22	1	0	4.194760	0.384062	-0.903107
23	1	0	4.408485	-0.544893	0.579381
24	1	0	4.321496	-1.381549	-0.969276
25	8	0	0.520168	3.771504	1.183307
26	1	0	1.034955	3.527339	1.956340
27	1	0	0.191849	2.903911	0.805924
28	8	0	-2.219192	2.385835	-1.625536
29	1	0	-1.569696	2.036219	-0.954513
30	1	0	-1.721955	3.030404	-2.135018

Rotational constants (GHZ): 0.6592475 0.5874810 0.3753732

- $2H_2O$/ B3PW91 6-311G+dp/CPCM

H_2O
Electronic -229.318978
Stoichiometry H6O3
Framework group C1[X(H6O3)]
Deg. of freedom 21
Full point group C1 NOp 1
Largest Abelian subgroup C1 NOp 1
Largest concise Abelian subgroup C1 NOp 1
Standard orientation:

Center Number	Atomic Number	Atomic Type	Coordinates (Angstroms) X	Y	Z
1	8	0	-2.296878	-0.593422	-0.000255
2	1	0	-1.952339	-1.490517	-0.000004
3	1	0	-1.508045	-0.025837	-0.000169
4	8	0	0.002110	1.143281	0.000013
5	1	0	0.007888	1.729571	0.765109
6	1	0	0.008090	1.729559	-0.765091
7	8	0	2.168610	-0.751604	0.000222
8	1	0	1.466189	-0.080055	0.000153
9	1	0	2.987486	-0.248760	0.000165

Rotational constants (GHZ): 11.2277243 2.8376767 2.2893942

OH^-
Electronic Energy -228.834586
Stoichiometry H5O3(1-)
Framework group C1[X(H5O3)]
Deg. of freedom 18

Full point group C1 NOp 1
Largest Abelian subgroup C1 NOp 1
Largest concise Abelian subgroup C1 NOp 1

Standard orientation:

Center Number	Atomic Number	Atomic Type	Coordinates (Angstroms) X	Y	Z
1	8	0	2.347649	-0.359291	-0.108003
2	1	0	1.408846	0.059358	-0.053254
3	1	0	2.607740	-0.514981	0.802915
4	8	0	-0.000168	0.634046	-0.006311
5	1	0	0.000681	1.593152	0.049920
6	8	0	-2.344661	-0.365915	0.108226
7	1	0	-1.407508	0.056137	0.051504
8	1	0	-2.632319	-0.464388	-0.802386

Rotational constants (GHZ): 33.0898352 2.5837218 2.4334453

Phenol
Electronic Energy -460.326830
Stoichiometry C6H10O3
Framework group C1[X(C6H10O3)]
Deg. of freedom 51
Full point group C1 NOp 1
Largest Abelian subgroup C1 NOp 1
Largest concise Abelian subgroup C1 NOp 1

Standard orientation:

Center Number	Atomic Number	Atomic Type	Coordinates (Angstroms) X	Y	Z
1	6	0	0.824771	1.146992	0.130214
2	6	0	0.153042	-0.042805	-0.160487
3	6	0	0.870558	-1.232867	-0.296290
4	6	0	2.252542	-1.228022	-0.140898
5	6	0	2.931663	-0.045569	0.148807
6	6	0	2.208778	1.137454	0.281858
7	1	0	0.262969	2.069445	0.234612
8	1	0	2.801718	-2.158038	-0.248786
9	1	0	4.009559	-0.046722	0.268492
10	8	0	-1.197977	-0.089922	-0.321782
11	1	0	-1.606474	0.800932	-0.222457
12	8	0	-2.959282	-2.202736	0.456433
13	1	0	-3.094520	-2.745662	-0.324885
14	1	0	-2.340245	-1.509932	0.170588
15	8	0	-2.418584	2.321105	-0.066497
16	1	0	-3.046305	2.396850	0.659884
17	1	0	-2.889520	2.638225	-0.844360
18	1	0	2.722632	2.066691	0.507089
19	1	0	0.338808	-2.150463	-0.524636

Rotational constants (GHZ): 1.7860070 0.8802264 0.6038646

Phenolate
Electronic Energy -459.858194
Stoichiometry C6H9O3(1-)
Framework group C1[X(C6H9O3)]
Deg. of freedom 48
Full point group C1 NOp 1
Largest Abelian subgroup C1 NOp 1
Largest concise Abelian subgroup C1 NOp 1

Standard orientation:

Center Number	Atomic Number	Atomic Type	Coordinates (Angstroms) X	Y	Z
1	6	0	-0.807930	-1.204678	-0.047769
2	6	0	-0.056705	-0.000004	-0.000036
3	6	0	-0.807930	1.204668	0.047732
4	6	0	-2.197922	1.197712	0.047175

5	6	0	-2.914369	-0.000006	0.000030
6	6	0	-2.197923	-1.197723	-0.047148
7	1	0	-0.268408	-2.147227	-0.085361
8	1	0	-2.731774	2.144292	0.084953
9	1	0	-3.999345	-0.000007	0.000056
10	8	0	1.251023	-0.000002	-0.000069
11	8	0	2.768464	-2.156645	0.155698
12	1	0	2.644215	-2.652039	-0.657539
13	1	0	2.152402	-1.373001	0.078373
14	8	0	2.768422	2.156694	-0.155625
15	1	0	2.152394	1.372999	-0.078536
16	1	0	2.644099	2.651887	0.657724
17	1	0	-0.268407	2.147218	0.085296
18	1	0	-2.731775	-2.144305	-0.084901

Rotational constants (GHZ): 1.9711824 0.9020585 0.6214956

Thymol
Electronic Energy -617.569391
Stoichiometry C10H18O3
Framework group C1[X(C10H18O3)]
Deg. of freedom 87
Full point group C1 NOp 1
Largest Abelian subgroup C1 NOp 1
Largest concise Abelian subgroup C1 NOp 1
Standard orientation:

Center Number	Atomic Number	Atomic Type	Coordinates (Angstroms) X	Y	Z
1	6	0	1.651783	0.289156	0.006594
2	6	0	0.262124	0.296732	0.113453
3	6	0	-0.483004	-0.892687	0.024502
4	6	0	0.236179	-2.070243	-0.176014
5	6	0	1.626258	-2.085946	-0.280916
6	6	0	2.355781	-0.901363	-0.189582
7	1	0	2.187269	1.231785	0.080322
8	1	0	-0.300973	-3.010087	-0.251090
9	1	0	2.143060	-3.028547	-0.434823
10	8	0	-0.417634	1.468592	0.303644
11	1	0	0.204932	2.211830	0.470709
12	6	0	-1.990411	-0.871254	0.199215
13	1	0	-2.348736	0.079693	-0.207678
14	6	0	-2.358732	-0.895822	1.690833
15	1	0	-1.884179	-0.072494	2.230877
16	1	0	-3.442198	-0.809909	1.823348
17	1	0	-2.035154	-1.835567	2.150888
18	6	0	-2.713074	-1.989043	-0.554315
19	1	0	-3.795364	-1.846722	-0.481662
20	1	0	-2.444329	-1.999957	-1.614665
21	1	0	-2.488516	-2.975729	-0.137141
22	6	0	3.855325	-0.888373	-0.316918
23	1	0	4.167294	-0.418822	-1.256071
24	1	0	4.319660	-0.321696	0.495572
25	1	0	4.261986	-1.901854	-0.300651
26	8	0	-2.127729	2.585559	-1.727480
27	1	0	-1.589976	2.176783	-1.028338
28	1	0	-2.969759	2.777655	-1.305756
29	8	0	1.190522	3.623320	0.781934
30	1	0	1.096640	4.031220	1.649237
31	1	0	1.103702	4.345692	0.151021

Rotational constants (GHZ): 0.6540042 0.5811268 0.3626310

Thymolate
Electronic Energy -617.098415
Stoichiometry C10H17O3(1-)
Framework group C1[X(C10H17O3)]
Deg. of freedom 84
Full point group C1 NOp 1
Largest Abelian subgroup C1 NOp 1
Largest concise Abelian subgroup C1 NOp 1

Standard orientation:

Center Number	Atomic Number	Atomic Type	Coordinates (Angstroms) X	Y	Z
1	6	0	1.626243	0.482127	-0.118661
2	6	0	0.219895	0.437627	0.058958
3	6	0	-0.393927	-0.851560	0.100021
4	6	0	0.409836	-1.981922	-0.037324
5	6	0	1.792053	-1.909315	-0.211526
6	6	0	2.416032	-0.658005	-0.248807
7	1	0	2.093695	1.463998	-0.156825
8	1	0	-0.058327	-2.964182	-0.009013
9	1	0	2.378495	-2.817801	-0.318428
10	8	0	-0.476422	1.544683	0.181604
11	6	0	-1.891859	-0.978238	0.304333
12	1	0	-2.281442	0.043342	0.343961
13	6	0	-2.231106	-1.654871	1.639338
14	1	0	-1.781682	-1.117951	2.480404
15	1	0	-3.314781	-1.685763	1.798396
16	1	0	-1.861813	-2.685994	1.666161
17	6	0	-2.582769	-1.699932	-0.859284
18	1	0	-3.668702	-1.720615	-0.715500
19	1	0	-2.377821	-1.202631	-1.812077
20	1	0	-2.241531	-2.737427	-0.944572
21	6	0	3.911865	-0.543684	-0.398254
22	1	0	4.194760	0.384062	-0.903107
23	1	0	4.408485	-0.544893	0.579381
24	1	0	4.321496	-1.381549	-0.969276
25	8	0	0.520168	3.771504	1.183307
26	1	0	1.034955	3.527339	1.956340
27	1	0	0.191849	2.903911	0.805924
28	8	0	-2.219192	2.385835	-1.625536
29	1	0	-1.569696	2.036219	-0.954513
30	1	0	-1.721955	3.030404	-2.135018

Rotational constants (GHZ): 0.6592475 0.5874810 0.3753732

- $2H_2O$/ W97XD 6-311G+dp/SMD

H_2O

Electronic Energy -229.348806
Stoichiometry H6O3
Framework group C1[X(H6O3)]
Deg. of freedom 21
Full point group C1 NOp 1
Largest Abelian subgroup C1 NOp 1
Largest concise Abelian subgroup C1 NOp 1
Standard orientation:

Center Number	Atomic Number	Atomic Type	Coordinates (Angstroms) X	Y	Z
1	8	0	-1.965536	-0.700126	-0.055753
2	1	0	-1.582981	-1.443920	0.416645
3	1	0	-1.294017	0.002533	-0.010773
4	8	0	0.002934	1.302634	0.008698
5	1	0	0.015580	1.826341	0.815938
6	1	0	0.021081	1.937068	-0.714988
7	8	0	1.827005	-0.829366	-0.013775
8	1	0	1.220503	-0.069017	-0.018204
9	1	0	2.704609	-0.438140	-0.001983

Rotational constants (GHZ): 8.9042224 3.8973181 2.7522536

OH^-

Electronic Energy -228.864447
Stoichiometry H5O3(1-)
Framework group C1[X(H5O3)]
Deg. of freedom 18

Full point group C1 NOp 1
Largest Abelian subgroup C1 NOp 1
Largest concise Abelian subgroup C1 NOp 1
Standard orientation:

Center Number	Atomic Number	Atomic Type	Coordinates (Angstroms) X	Y	Z
1	8	0	-2.203038	-0.502190	0.073485
2	1	0	-1.344472	0.045347	0.022335
3	1	0	-2.783304	-0.139629	-0.603202
4	8	0	-0.001605	0.821737	-0.051405
5	1	0	0.002120	1.546942	0.580644
6	8	0	2.265709	-0.386666	-0.096212
7	1	0	1.366447	0.092099	-0.045469
8	1	0	2.270689	-1.007805	0.638745

Rotational constants (GHZ): 22.9681042 2.8416324 2.5629840

Phenol
Electronic Energy -460.361903
Stoichiometry C6H10O3
Framework group C1[X(C6H10O3)]
Deg. of freedom 51
Full point group C1 NOp 1
Largest Abelian subgroup C1 NOp 1
Largest concise Abelian subgroup C1 NOp 1
Standard orientation:

Center Number	Atomic Number	Atomic Type	Coordinates (Angstroms) X	Y	Z
1	6	0	-0.749993	-1.195204	0.134636
2	6	0	-0.132570	0.004299	-0.205507
3	6	0	-0.886805	1.163843	-0.354539
4	6	0	-2.260290	1.118706	-0.163153
5	6	0	-2.889035	-0.074629	0.175678
6	6	0	-2.126196	-1.226579	0.322260
7	1	0	-0.155413	-2.093637	0.248689
8	1	0	-2.842709	2.025132	-0.280410
9	1	0	-3.961370	-0.105608	0.324109
10	8	0	1.217640	0.093760	-0.406637
11	1	0	1.676579	-0.768340	-0.259601
12	8	0	2.613743	2.345298	0.516097
13	1	0	2.370700	3.073820	-0.065512
14	1	0	2.113269	1.578617	0.180170
15	8	0	2.625076	-2.180506	-0.061852
16	1	0	3.093538	-2.194250	0.782076
17	1	0	3.314773	-2.252411	-0.733563
18	1	0	-2.602805	-2.163584	0.585959
19	1	0	-0.388894	2.089220	-0.619045

Rotational constants (GHZ): 1.7779602 0.9132646 0.6231768

Phenolate
Electronic Energy -459.891878
Stoichiometry C6H9O3(1-)
Framework group C1[X(C6H9O3)]
Deg. of freedom 48
Full point group C1 NOp 1
Largest Abelian subgroup C1 NOp 1
Largest concise Abelian subgroup C1 NOp 1
Standard orientation:

Center Number	Atomic Number	Atomic Type	Coordinates (Angstroms) X	Y	Z
1	6	0	0.782663	1.179975	-0.022191
2	6	0	0.036633	-0.016717	-0.082545
3	6	0	0.764346	-1.225667	-0.063561
4	6	0	2.149395	-1.230641	0.011167

5	6	0	2.869554	-0.040525	0.069350
6	6	0	2.167686	1.161626	0.052125
7	1	0	0.249849	2.125043	-0.036394
8	1	0	2.675056	-2.179869	0.022440
9	1	0	3.951229	-0.049723	0.126246
10	8	0	-1.281488	-0.004925	-0.157929
11	8	0	-2.622618	2.251650	0.070168
12	1	0	-1.995339	2.974778	-0.033559
13	1	0	-2.076061	1.419111	-0.020777
14	8	0	-2.746556	-2.187903	-0.001705
15	1	0	-2.155607	-1.382544	-0.037944
16	1	0	-2.989746	-2.286874	0.924445
17	1	0	0.216700	-2.160695	-0.110728
18	1	0	2.707549	2.101894	0.095925

Rotational constants (GHZ): 1.9069249 0.9428061 0.6333924

Thymol
Electronic Energy -617.618699
Stoichiometry C10H18O3
Framework group C1[X(C10H18O3)]
Deg. of freedom 87
Full point group C1 NOp 1
Largest Abelian subgroup C1 NOp 1
Largest concise Abelian subgroup C1 NOp 1
Standard orientation:

Center Number	Atomic Number	Atomic Type	Coordinates (Angstroms) X	Y	Z
1	6	0	1.665616	0.158736	0.041080
2	6	0	0.286711	0.287203	0.141467
3	6	0	-0.561579	-0.824316	0.037304
4	6	0	0.045990	-2.057729	-0.168821
5	6	0	1.427511	-2.196515	-0.266965
6	6	0	2.257221	-1.086523	-0.163075
7	1	0	2.284401	1.046277	0.123516
8	1	0	-0.571132	-2.943659	-0.255285
9	1	0	1.859189	-3.178211	-0.426824
10	8	0	-0.288008	1.517452	0.340649
11	1	0	0.393749	2.216888	0.480167
12	6	0	-2.062478	-0.661285	0.194945
13	1	0	-2.335413	0.292605	-0.263522
14	6	0	-2.441587	-0.584925	1.679171
15	1	0	-1.902188	0.213933	2.191793
16	1	0	-3.512718	-0.397873	1.793455
17	1	0	-2.208033	-1.528128	2.181515
18	6	0	-2.874140	-1.750001	-0.503582
19	1	0	-3.938535	-1.510655	-0.444357
20	1	0	-2.607134	-1.839412	-1.559304
21	1	0	-2.733530	-2.726755	-0.033932
22	6	0	3.752085	-1.206768	-0.278810
23	1	0	4.119106	-0.693948	-1.171959
24	1	0	4.252611	-0.755411	0.581169
25	1	0	4.058192	-2.251777	-0.341649
26	8	0	-1.791200	2.581486	-1.794162
27	1	0	-1.296881	2.184247	-1.052955
28	1	0	-2.583813	2.039854	-1.876270
29	8	0	1.438209	3.551248	0.792814
30	1	0	0.952282	4.335449	1.076898
31	1	0	1.925751	3.827822	0.006854

Rotational constants (GHZ): 0.6675340 0.5885093 0.3711919

Thymolate
Electronic Energy -617.144926
Stoichiometry C10H17O3(1-)
Framework group C1[X(C10H17O3)]
Deg. of freedom 84
Full point group C1 NOp 1
Largest Abelian subgroup C1 NOp 1
Largest concise Abelian subgroup C1 NOp 1

Standard orientation:

Center Number	Atomic Number	Atomic Type	Coordinates (Angstroms) X	Y	Z
1	6	0	-1.548924	0.842635	0.091914
2	6	0	-0.204408	0.432598	0.011707
3	6	0	0.064382	-0.961051	-0.100770
4	6	0	-1.009386	-1.841062	-0.118963
5	6	0	-2.333854	-1.412879	-0.033629
6	6	0	-2.613364	-0.053820	0.070513
7	1	0	-1.747323	1.907254	0.176329
8	1	0	-0.817588	-2.905041	-0.203166
9	1	0	-3.141767	-2.136211	-0.050916
10	8	0	0.766711	1.328000	0.040331
11	6	0	1.503475	-1.436978	-0.160200
12	1	0	2.058575	-0.696962	-0.742837
13	6	0	1.687176	-2.788216	-0.848776
14	1	0	1.247027	-2.796014	-1.849089
15	1	0	2.752331	-3.012212	-0.948738
16	1	0	1.236496	-3.602689	-0.275622
17	6	0	2.123902	-1.474111	1.242512
18	1	0	3.188082	-1.722271	1.193083
19	1	0	2.024917	-0.513367	1.750972
20	1	0	1.629543	-2.233611	1.856050
21	6	0	-4.032239	0.447312	0.140478
22	1	0	-4.160531	1.168525	0.951151
23	1	0	-4.318142	0.951851	-0.787090
24	1	0	-4.733315	-0.373517	0.301096
25	8	0	0.391646	3.915384	-0.182111
26	1	0	0.070527	4.089603	-1.072742
27	1	0	0.469981	2.918819	-0.115766
28	8	0	3.405548	1.570374	-0.087435
29	1	0	2.430055	1.388827	-0.045704
30	1	0	3.479335	2.530381	-0.072009

Rotational constants (GHZ): 0.7419549 0.5837649 0.3467626

- $2H_2O$/ W97XD 6-311G+dp/PCM

H_2O

Electronic Energy -229.335123
Stoichiometry H6O3
Framework group C1[X(H6O3)]
Deg. of freedom 21
Full point group C1 NOp 1
Largest Abelian subgroup C1 NOp 1
Largest concise Abelian subgroup C1 NOp 1

Standard orientation:

Center Number	Atomic Number	Atomic Type	Coordinates (Angstroms) X	Y	Z
1	8	0	-2.089611	-0.678902	0.000039
2	1	0	-1.625799	-1.517928	-0.000040
3	1	0	-1.389917	-0.008410	-0.000006
4	8	0	-0.002943	1.272775	-0.000022
5	1	0	0.007357	1.853483	0.765859
6	1	0	0.007302	1.853586	-0.765825
7	8	0	1.948936	-0.807808	-0.000112
8	1	0	1.330103	-0.061968	-0.000065
9	1	0	2.819892	-0.407289	0.000840

Rotational constants (GHZ): 9.3158328 3.4572268 2.5515749

OH^-

Electronic Energy -228.848407
Stoichiometry H5O3(1-)
Framework group C1[X(H5O3)]
Deg. of freedom 18

Full point group C1 NOp 1
Largest Abelian subgroup C1 NOp 1
Largest concise Abelian subgroup C1 NOp 1
Standard orientation:

Center Number	Atomic Number	Atomic Type	Coordinates (Angstroms) X	Y	Z
1	8	0	2.340848	-0.383085	-0.107284
2	1	0	1.426195	0.057161	-0.057513
3	1	0	2.598618	-0.534647	0.802565
4	8	0	-0.000377	0.681282	-0.005006
5	1	0	0.000424	1.638630	0.037913
6	8	0	-2.338284	-0.387959	0.107623
7	1	0	-1.425067	0.055430	0.056126
8	1	0	-2.617669	-0.498480	-0.801756

Rotational constants (GHZ): 29.7077647 2.5972631 2.4249258

Phenol
Electronic Energy -460.352300
Stoichiometry C6H10O3
Framework group C1[X(C6H10O3)]
Deg. of freedom 51
Full point group C1 NOp 1
Largest Abelian subgroup C1 NOp 1
Largest concise Abelian subgroup C1 NOp 1
Standard orientation:

Center Number	Atomic Number	Atomic Type	Coordinates (Angstroms) X	Y	Z
1	6	0	-0.743471	-1.172240	0.125483
2	6	0	-0.107250	0.025220	-0.199710
3	6	0	-0.848918	1.197166	-0.338159
4	6	0	-2.225130	1.166941	-0.151189
5	6	0	-2.871020	-0.023324	0.173058
6	6	0	-2.122182	-1.187829	0.308893
7	1	0	-0.158958	-2.079418	0.232006
8	1	0	-2.795990	2.082408	-0.260879
9	1	0	-3.944760	-0.042309	0.317553
10	8	0	1.237468	0.099332	-0.392875
11	1	0	1.666337	-0.772732	-0.264913
12	8	0	2.666016	2.365333	0.506968
13	1	0	2.714464	2.960612	-0.242939
14	1	0	2.186798	1.588730	0.177920
15	8	0	2.449636	-2.303453	-0.032910
16	1	0	3.065524	-2.372225	0.700908
17	1	0	2.901165	-2.688465	-0.788154
18	1	0	-2.611588	-2.122157	0.561099
19	1	0	-0.340127	2.120256	-0.592311

Rotational constants (GHZ): 1.7034982 0.9391133 0.6241319

Phenolate
Electronic Energy -459.881628
Stoichiometry C6H9O3(1-)
Framework group C1[X(C6H9O3)]
Deg. of freedom 48
Full point group C1 NOp 1
Largest Abelian subgroup C1 NOp 1
Largest concise Abelian subgroup C1 NOp 1
Standard orientation:

Center Number	Atomic Number	Atomic Type	Coordinates (Angstroms) X	Y	Z
1	6	0	0.775999	1.204878	-0.014645
2	6	0	0.027996	-0.000017	-0.000066
3	6	0	0.775988	-1.204918	0.014576
4	6	0	2.164501	-1.197593	0.014330

5	6	0	2.879423	-0.000028	0.000053
6	6	0	2.164512	1.197542	-0.014284
7	1	0	0.235312	2.146815	-0.025348
8	1	0	2.698504	-2.143621	0.025854
9	1	0	3.963487	-0.000033	0.000098
10	8	0	-1.277689	-0.000011	-0.000122
11	8	0	-2.696518	2.231214	0.114499
12	1	0	-2.463228	2.743758	-0.660651
13	1	0	-2.133805	1.413049	0.062477
14	8	0	-2.696713	-2.231119	-0.114365
15	1	0	-2.133824	-1.413052	-0.062680
16	1	0	-2.463407	-2.743481	0.660902
17	1	0	0.235292	-2.146850	0.025232
18	1	0	2.698524	2.143565	-0.025764

Rotational constants (GHZ): 1.8887015 0.9386221 0.6289976

Thymol

Electronic Energy -617.609391
Stoichiometry C10H18O3
Framework group C1[X(C10H18O3)]
Deg. of freedom 87
Full point group C1 NOp 1
Largest Abelian subgroup C1 NOp 1
Largest concise Abelian subgroup C1 NOp 1

Standard orientation:

Center Number	Atomic Number	Atomic Type	Coordinates (Angstroms) X	Y	Z
1	6	0	1.660848	0.281385	0.014929
2	6	0	0.273168	0.304126	0.107032
3	6	0	-0.484858	-0.873889	0.015307
4	6	0	0.218550	-2.060111	-0.171310
5	6	0	1.608391	-2.092589	-0.261615
6	6	0	2.348751	-0.918343	-0.168990
7	1	0	2.207910	1.216597	0.090294
8	1	0	-0.327804	-2.993337	-0.248525
9	1	0	2.114856	-3.041448	-0.405374
10	8	0	-0.395218	1.481909	0.287310
11	1	0	0.232625	2.221219	0.422766
12	6	0	-1.994320	-0.824608	0.171945
13	1	0	-2.345519	0.075817	-0.340426
14	6	0	-2.372016	-0.689434	1.654413
15	1	0	-1.897840	0.185493	2.104401
16	1	0	-3.455542	-0.589256	1.768532
17	1	0	-2.052278	-1.577537	2.209319
18	6	0	-2.717906	-2.014378	-0.459354
19	1	0	-3.798291	-1.852779	-0.418147
20	1	0	-2.434097	-2.149768	-1.506514
21	1	0	-2.508167	-2.946355	0.074538
22	6	0	3.851286	-0.921307	-0.282181
23	1	0	4.171635	-0.469713	-1.225947
24	1	0	4.309221	-0.345439	0.526185
25	1	0	4.247785	-1.937721	-0.245423
26	8	0	-2.219273	2.435508	-1.667425
27	1	0	-1.617920	2.095604	-0.986038
28	1	0	-3.062536	2.536285	-1.222346
29	8	0	1.286633	3.594579	0.653535
30	1	0	1.246593	4.041990	1.502408
31	1	0	1.240878	4.289272	-0.008121

Rotational constants (GHZ): 0.6818712 0.5812152 0.3649818

Thymolate

Electronic Energy- -617.136181
Stoichiometry C10H17O3(1-)
Framework group C1[X(C10H17O3)]
Deg. of freedom 84
Full point group C1 NOp 1
Largest Abelian subgroup C1 NOp 1
Largest concise Abelian subgroup C1 NOp 1

Standard orientation:

Center Number	Atomic Number	Atomic Type	Coordinates (Angstroms) X	Y	Z
1	6	0	-1.570857	0.794648	0.015013
2	6	0	-0.205957	0.430455	-0.057900
3	6	0	0.100739	-0.964758	-0.104461
4	6	0	-0.943112	-1.880658	-0.064789
5	6	0	-2.282603	-1.493822	0.013713
6	6	0	-2.604162	-0.138145	0.050807
7	1	0	-1.806486	1.856028	0.049787
8	1	0	-0.715832	-2.942265	-0.096963
9	1	0	-3.066247	-2.244772	0.043277
10	8	0	0.728518	1.343539	-0.082613
11	6	0	1.555681	-1.390405	-0.149818
12	1	0	2.077545	-0.663184	-0.780379
13	6	0	1.787526	-2.775330	-0.756119
14	1	0	1.324221	-2.865851	-1.742838
15	1	0	2.860096	-2.960662	-0.866183
16	1	0	1.385239	-3.570378	-0.119831
17	6	0	2.183859	-1.310007	1.249301
18	1	0	3.256541	-1.527031	1.212424
19	1	0	2.052174	-0.313696	1.676934
20	1	0	1.712046	-2.038302	1.918249
21	6	0	-4.041871	0.319154	0.099367
22	1	0	-4.163859	1.180193	0.761867
23	1	0	-4.390553	0.621494	-0.893724
24	1	0	-4.700482	-0.478619	0.450996
25	8	0	0.212645	3.933658	-0.013898
26	1	0	-0.375909	4.120353	-0.746528
27	1	0	0.360561	2.949299	-0.045324
28	8	0	3.371978	1.642731	-0.153836
29	1	0	2.407755	1.426385	-0.113974
30	1	0	3.402603	2.544793	-0.475694

Rotational constants (GHZ): 0.7450336 0.5849983 0.3463810

- $2H_2O$/ W97XD 6-311G+dp/CPCM

H_2O

Electronic Energy -229.335224
Stoichiometry H6O3
Framework group C1[X(H6O3)]
Deg. of freedom 21
Full point group C1 NOp 1
Largest Abelian subgroup C1 NOp 1
Largest concise Abelian subgroup C1 NOp 1

Standard orientation:

Center Number	Atomic Number	Atomic Type	Coordinates (Angstroms) X	Y	Z
1	8	0	-2.089611	-0.678902	0.000039
2	1	0	-1.625799	-1.517928	-0.000040
3	1	0	-1.389917	-0.008410	-0.000006
4	8	0	-0.002943	1.272775	-0.000022
5	1	0	0.007357	1.853483	0.765859
6	1	0	0.007302	1.853586	-0.765825
7	8	0	1.948936	-0.807808	-0.000112
8	1	0	1.330103	-0.061968	-0.000065
9	1	0	2.819892	-0.407289	0.000840

Rotational constants (GHZ): 9.3158328 3.4572268 2.5515749

OH^-

Electronic Energy -228.848526
Stoichiometry H5O3(1-)
Framework group C1[X(H5O3)]
Deg. of freedom 18

Full point group C1 NOp 1
Largest Abelian subgroup C1 NOp 1
Largest concise Abelian subgroup C1 NOp 1
Standard orientation:

Center Number	Atomic Number	Atomic Type	Coordinates (Angstroms) X	Y	Z
1	8	0	2.340848	-0.383085	-0.107284
2	1	0	1.426195	0.057161	-0.057513
3	1	0	2.598618	-0.534647	0.802565
4	8	0	-0.000377	0.681282	-0.005006
5	1	0	0.000424	1.638630	0.037913
6	8	0	-2.338284	-0.387959	0.107623
7	1	0	-1.425067	0.055430	0.056126
8	1	0	-2.617669	-0.498480	-0.801756

Rotational constants (GHZ): 29.7077647 2.5972631 2.4249258

Phenol
Electronic Energy -460.352403
Stoichiometry C6H10O3
Framework group C1[X(C6H10O3)]
Deg. of freedom 51
Full point group C1 NOp 1
Largest Abelian subgroup C1 NOp 1
Largest concise Abelian subgroup C1 NOp 1
Standard orientation:

Center Number	Atomic Number	Atomic Type	Coordinates (Angstroms) X	Y	Z
1	6	0	-0.743471	-1.172240	0.125483
2	6	0	-0.107250	0.025220	-0.199710
3	6	0	-0.848918	1.197166	-0.338159
4	6	0	-2.225130	1.166941	-0.151189
5	6	0	-2.871020	-0.023324	0.173058
6	6	0	-2.122182	-1.187829	0.308893
7	1	0	-0.158958	-2.079418	0.232006
8	1	0	-2.795990	2.082408	-0.260879
9	1	0	-3.944760	-0.042309	0.317553
10	8	0	1.237468	0.099332	-0.392875
11	1	0	1.666337	-0.772732	-0.264913
12	8	0	2.666016	2.365333	0.506968
13	1	0	2.714464	2.960612	-0.242939
14	1	0	2.186798	1.588730	0.177920
15	8	0	2.449636	-2.303453	-0.032910
16	1	0	3.065524	-2.372225	0.700908
17	1	0	2.901165	-2.688465	-0.788154
18	1	0	-2.611588	-2.122157	0.561099
19	1	0	-0.340127	2.120256	-0.592311

Rotational constants (GHZ): 1.7034982 0.9391133 0.6241319

Phenolate
Electronic Energy -459.881822
Stoichiometry C6H9O3(1-)
Framework group C1[X(C6H9O3)]
Deg. of freedom 48
Full point group C1 NOp 1
Largest Abelian subgroup C1 NOp 1
Largest concise Abelian subgroup C1 NOp 1
Standard orientation:

Center Number	Atomic Number	Atomic Type	Coordinates (Angstroms) X	Y	Z
1	6	0	0.775999	1.204878	-0.014645
2	6	0	0.027996	-0.000017	-0.000066
3	6	0	0.775988	-1.204918	0.014576
4	6	0	2.164501	-1.197593	0.014330

5	6	0	2.879423	-0.000028	0.000053
6	6	0	2.164512	1.197542	-0.014284
7	1	0	0.235312	2.146815	-0.025348
8	1	0	2.698504	-2.143621	0.025854
9	1	0	3.963487	-0.000033	0.000098
10	8	0	-1.277689	-0.000011	-0.000122
11	8	0	-2.696518	2.231214	0.114499
12	1	0	-2.463228	2.743758	-0.660651
13	1	0	-2.133805	1.413049	0.062477
14	8	0	-2.696713	-2.231119	-0.114365
15	1	0	-2.133824	-1.413052	-0.062680
16	1	0	-2.463407	-2.743481	0.660902
17	1	0	0.235292	-2.146850	0.025232
18	1	0	2.698524	2.143565	-0.025764

Rotational constants (GHZ): 1.8887015 0.9386221 0.6289976

Thymol
Electronic Energy -617.609504
Stoichiometry C10H18O3
Framework group C1[X(C10H18O3)]
Deg. of freedom 87
Full point group C1 NOp 1
Largest Abelian subgroup C1 NOp 1
Largest concise Abelian subgroup C1 NOp 1
Standard orientation:

Center Number	Atomic Number	Atomic Type	Coordinates (Angstroms) X	Y	Z
1	6	0	1.660848	0.281385	0.014929
2	6	0	0.273168	0.304126	0.107032
3	6	0	-0.484858	-0.873889	0.015307
4	6	0	0.218550	-2.060111	-0.171310
5	6	0	1.608391	-2.092589	-0.261615
6	6	0	2.348751	-0.918343	-0.168990
7	1	0	2.207910	1.216597	0.090294
8	1	0	-0.327804	-2.993337	-0.248525
9	1	0	2.114856	-3.041448	-0.405374
10	8	0	-0.395218	1.481909	0.287310
11	1	0	0.232625	2.221219	0.422766
12	6	0	-1.994320	-0.824608	0.171945
13	1	0	-2.345519	0.075817	-0.340426
14	6	0	-2.372016	-0.689434	1.654413
15	1	0	-1.897840	0.185493	2.104401
16	1	0	-3.455542	-0.589256	1.768532
17	1	0	-2.052278	-1.577537	2.209319
18	6	0	-2.717906	-2.014378	-0.459354
19	1	0	-3.798291	-1.852779	-0.418147
20	1	0	-2.434097	-2.149768	-1.506514
21	1	0	-2.508167	-2.946355	0.074538
22	6	0	3.851286	-0.921307	-0.282181
23	1	0	4.171635	-0.469713	-1.225947
24	1	0	4.309221	-0.345439	0.526185
25	1	0	4.247785	-1.937721	-0.245423
26	8	0	-2.219273	2.435508	-1.667425
27	1	0	-1.617920	2.095604	-0.986038
28	1	0	-3.062536	2.536285	-1.222346
29	8	0	1.286633	3.594579	0.653535
30	1	0	1.246593	4.041990	1.502408
31	1	0	1.240878	4.289272	-0.008121

Rotational constants (GHZ): 0.6818712 0.5812152 0.3649818

Thymolate
Electronic Energy- -617.136385
Stoichiometry C10H17O3(1-)
Framework group C1[X(C10H17O3)]
Deg. of freedom 84
Full point group C1 NOp 1
Largest Abelian subgroup C1 NOp 1
Largest concise Abelian subgroup C1 NOp 1

Standard orientation:

Center Number	Atomic Number	Atomic Type	Coordinates (Angstroms) X	Y	Z
1	6	0	-1.572364	0.791675	0.010703
2	6	0	-0.206687	0.430340	-0.061406
3	6	0	0.103136	-0.964324	-0.104532
4	6	0	-0.938678	-1.882432	-0.062008
5	6	0	-2.279032	-1.498383	0.015871
6	6	0	-2.603635	-0.143351	0.049322
7	1	0	-1.810570	1.852577	0.042496
8	1	0	-0.709067	-2.943611	-0.091285
9	1	0	-3.060943	-2.251034	0.047689
10	8	0	0.725768	1.345624	-0.088158
11	6	0	1.558963	-1.387060	-0.148999
12	1	0	2.079442	-0.660166	-0.781080
13	6	0	1.793659	-2.772843	-0.752247
14	1	0	1.330382	-2.866548	-1.738687
15	1	0	2.866607	-2.956085	-0.862113
16	1	0	1.393250	-3.567378	-0.114166
17	6	0	2.186820	-1.302451	1.250037
18	1	0	3.259877	-1.517726	1.213855
19	1	0	2.053258	-0.305531	1.675724
20	1	0	1.716254	-2.030207	1.920455
21	6	0	-4.042341	0.310893	0.096860
22	1	0	-4.165829	1.174747	0.755389
23	1	0	-4.392425	0.607798	-0.897372
24	1	0	-4.698863	-0.486761	0.452615
25	8	0	0.202476	3.933944	-0.004486
26	1	0	-0.400431	4.122304	-0.724924
27	1	0	0.352927	2.950246	-0.043068
28	8	0	3.369124	1.648761	-0.153119
29	1	0	2.404991	1.431498	-0.115784
30	1	0	3.403155	2.526862	-0.535253

Rotational constants (GHZ): 0.7452069 0.5850133 0.3464057

References

1. Sabuzi, F.; Churakova, E.; Galloni, P.; Wever, R.; Hollmann, F.; Floris, B.; Conte, V. Thymol Bromination – A Comparison between Enzymatic and Chemical Catalysis. *Eur. J. Inorg. Chem.* **2015**, 3519-3525.
2. Piombino, C.; Lange, H.; Sabuzi, F.; Galloni, P.; Conte, V.; Crestini, C. Lignosulfonate Microcapsules for Delivery and Controlled Release of Thymol and Derivatives. *Molecules* **2020**, *25*, 866.
3. Joshi, A.V.; Baidoosi, M.; Mukhopadhyay, S.; Sasson, Y. Nitration of Phenol and Substituted Phenols with Dilute Nitric Acid Using Phase-Transfer Catalysts. *Org. Proc. Res. Dev.* **2003**, *7*, 95–97.
